# Suppression of Dexter transfer by covalent encapsulation for efficient matrix-free narrowband deep blue hyperfluorescent OLEDs

**DOI:** 10.1038/s41563-024-01812-4

**Published:** 2024-03-13

**Authors:** Hwan-Hee Cho, Daniel G. Congrave, Alexander J. Gillett, Stephanie Montanaro, Haydn E. Francis, Víctor Riesgo-Gonzalez, Junzhi Ye, Rituparno Chowdury, Weixuan Zeng, Marc K. Etherington, Jeroen Royakkers, Oliver Millington, Andrew D. Bond, Felix Plasser, Jarvist M. Frost, Clare P. Grey, Akshay Rao, Richard H. Friend, Neil C. Greenham, Hugo Bronstein

**Affiliations:** 1 Cavendish Laboratory, University of Cambridge, Cambridge, UK; 2 Yusuf Hamied Department of Chemistry, University of Cambridge, Cambridge, UK; 3 The Faraday Institution, Quad One, Harwell Science and Innovation Campus, Didcot, UK; 4 Department of Mathematics, Physics and Electrical Engineering, Northumbria University, Ellison Place, Newcastle upon Tyne, UK; 5 Department of Chemistry, Loughborough University, Loughborough, UK; 6 Department of Physics, Imperial College London, London, UK

**Keywords:** Optical materials, Organic LEDs, Energy transfer

## Abstract

Hyperfluorescence shows great promise for the next generation of commercially feasible blue organic light-emitting diodes, for which eliminating the Dexter transfer to terminal emitter triplet states is key to efficiency and stability. Current devices rely on high-gap matrices to prevent Dexter transfer, which unfortunately leads to overly complex devices from a fabrication standpoint. Here we introduce a molecular design where ultranarrowband blue emitters are covalently encapsulated by insulating alkylene straps. Organic light-emitting diodes with simple emissive layers consisting of pristine thermally activated delayed fluorescence hosts doped with encapsulated terminal emitters exhibit negligible external quantum efficiency drops compared with non-doped devices, enabling a maximum external quantum efficiency of 21.5%. To explain the high efficiency in the absence of high-gap matrices, we turn to transient absorption spectroscopy. It is directly observed that Dexter transfer from a pristine thermally activated delayed fluorescence sensitizer host can be substantially reduced by an encapsulated terminal emitter, opening the door to highly efficient ‘matrix-free’ blue hyperfluorescence.

## Abstract (web summary)

Suppressed Dexter transfer is needed to achieve efficient and stable hyperfluorescence, but complex matrices must be involved. A molecular design strategy has been proposed where Dexter transfer can be substantially reduced by an encapsulated terminal emitter, leading to ‘matrix-free’ hyperfluorescence.

## Main

The ‘blue OLED problem’—the quest for a stable and efficient blue organic light-emitting diode (OLED), has plagued academic and industrial researchers alike for over 20 years^1,2^. Thermally activated delayed fluorescence (TADF)-sensitized fluorescent OLEDs (coined as Hyperfluorescent in other work^3,4^) are considered one of the most promising contemporary solutions, combining the high efficiency of a TADF donor with the stability and colour purity of a conventional fluorescent acceptor^2,5,6^.

In current hyperfluorescent OLEDs, a high triplet energy matrix is implemented alongside the TADF sensitizer and emitter molecules, affording a three-component emissive layer (EML) (or even four in the case of exciplex/mixed matrices^7^)—a device structure that we term matrix-containing hyperfluorescence (MCHF)^2^. Progress in blue MCHF has been swift, with efficient devices now approaching the Commission Internationale de l'Éclairage (CIE_*xy*_) colour coordinates for display requirements, owing to the concurrent development of efficient TADF sensitizers and narrowband terminal emitters^2,7–15^. However, compared with the typical two-component host–guest EML already adopted by industry, the additional matrices required in MCHF devices unavoidably increases the complexity of device fabrication. Power efficiency is also reduced due to higher turn-on and driving voltages. A simple and efficient matrix-free hyperfluorescent (MFHF)^16^ device (first introduced in another work^17^) with a two-component EML solely consisting of a TADF sensitizer (hereafter referred to as the host) alongside a narrowband terminal emitter, would afford a new paradigm in blue OLED technology from a device fabrication perspective.

The underlying obstacle to MFHF is maintaining high device performance and simplifying the device structure. Dilution into a high triplet energy matrix has so far been inescapable to suppress Dexter transfer to long-lived terminal emitter triplets. Although Dexter transfer to terminal emitter triplets has been challenging to directly observe, it has been hypothesized to be highly disadvantageous in hyperfluorescent devices from both efficiency and stability perspectives^18–20^. First, due to their non-radiative nature, the population of terminal emitter triplet states is an efficiency loss pathway. Second, it has been suggested that the operational stability of hyperfluorescent OLEDs is adversely affected by the population of terminal emitter triplets due to a substantial increase in the exciton residence time^21^. Multiresonant (MR) TADF materials are a popular class of terminal emitter that could, in principle, alleviate the efficiency loss associated with Dexter transfer through their ability to triplet harvest. However, to the best of our knowledge, such a process has not been unequivocally observed^1,22^. The problem of an elongated exciton residence time through the Dexter population of terminal emitter triplets also persists, as MR TADF reverse intersystem crossing rates are typically orders of magnitude slower than for conventional donor–acceptor TADF materials^11,23^.

In this work, molecular design provides a new approach to suppress loss through the population of terminal emitter triplet states—the encapsulated ultranarrowband blue emitter. When doped into a pristine TADF host with no additional matrix, transient absorption (TA) studies directly indicate that Dexter transfer can be substantially curtailed by decorating a terminal emitter with insulating alkylene straps. Owing to Dexter transfer suppression, negligible efficiency loss is observed in MFHF OLEDs compared with non-doped TADF devices, with external quantum efficiencies (EQEs) as high as 21.5% and narrow true-blue electroluminescence (EL) (14–15 nm full-width at half-maximum (FWHM)). Hence, simply structured MFHF devices with narrowband blue emission and high efficiency have been realized for the first time.

## Molecular design

**Fig. 1:**
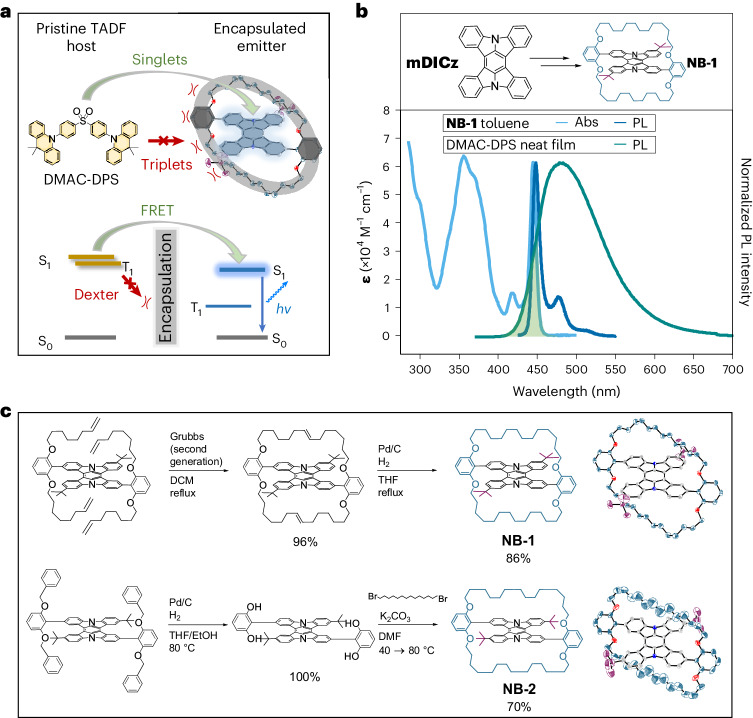
Molecular design. **a**, Schematic and Jablonski diagram for the suppression of Dexter triplet transfer from a TADF host via emitter encapsulation. **b**, Structures of the **mDICz** and encapsulated **NB-1** luminophores; relevant absorption and PL spectra for **NB-1** and DMAC-DPS. **c**, Synthesis schemes and structures of **NB-1** and **NB-2** with X-ray single-crystal structures (H atoms are omitted for clarity).

The design and syntheses of new terminal emitters **NB-1** and **NB-2** are illustrated in Fig. 1. In hyperfluorescence, the working mechanism is an energy cascade from a triplet-harvesting TADF donor (our host) to a fluorescent terminal emitter. To achieve efficient blue MFHF OLEDs, we sought to design emitters with structural and photophysical properties that promote singlet transfer via Förster resonant energy transfer (FRET)^18^ while suppressing Dexter transfer (Fig. 1a)^24,25^.

FRET proceeds via dipole–dipole coupling, with its efficiency linked to the spectral overlap between the host photoluminescence (PL) and the emitter absorption. As the S_0_ ↔ S_1_ transitions of the TADF host and fluorescent emitter are spin allowed, the desirable singlet energy transfer is accessible via FRET. FRET follows an *R*^−6^ distance dependence, making it efficient over rather long distances—on the molecular scale—of up to around 10 nm (ref. ^18^). Conversely, as the S_0_ → T_*n*_ transitions of fluorescent emitters are quantum mechanically forbidden, energy transfer to their triplet states is restricted to a Dexter transfer mechanism. Dexter transfer proceeds via direct host–emitter orbital overlap and is therefore restricted to the Ångstrom length scale, with its efficiency dropping off exponentially with distance^24,25^. Increasing the host–emitter distance in a system with good spectral overlap is, therefore, a plausible strategy to suppress Dexter transfer while preserving efficient FRET, which would prevent triplets accumulating on the terminal emitter to simultaneously improve the device efficiency and stability^18–20^.

When designing **NB-1** and **NB-2**, we first sought to maximize singlet transfer via FRET. A highly rigid and planar indolocarbazole heterocycle (**mDICz**)^26–28^ was selected as our blue luminophore (Fig. 1b), which displays intense 0–0 absorption (*ɛ*, *~*60,000 M^−1^ cm^−1^), a narrow Stokes shift (2–3 nm), an extremely small PL FWHM (*~*10 nm), unity fluorescence quantum yield (*Φ*_PL_) and high photostability (Supplementary Fig. 66). Computations suggest that the narrow emission can be understood in view of its strongly distorted central benzene ring (Supplementary Note 3). The distortion suppresses the Franck–Condon activity of the aromatic breathing vibration, inducing a weak vibronic progression.

The solution absorption and PL spectra of **NB-1** are shown in Fig. 1b alongside the neat film PL of DMAC-DPS—an established blue TADF material with high reported non-doped efficiency (*Φ*_PL_ = *~*100%, EQE = *~*20%), which functions as one of our host materials^29^. The photophysical properties of **NB-1** are clearly beneficial for maximizing the spectral overlap with the TADF host to promote efficient FRET while preserving pure-blue emission. Our design also allows a subtle tuning of the emitter bandgap for matching with different host materials. Modifying the position of the peripheral *tert*-butyl groups of **NB-1** affords the regioisomer **NB-2** (Fig. 1c), for which the solution absorption and emission spectra are redshifted by 6–7 nm (Supplementary Figs. 59 and 60). Supplementary Note 5 presents a full photophysical characterization of the emitters.

Second, we focused on suppressing the Dexter transfer. Previous evidence indicates that alkylene encapsulation can suppress the intermolecular interactions leading to concentration quenching and aggregation^30–35^. We designed **NB-1** and **NB-2** hypothesizing that the encapsulating straps would similarly shield the luminophore core from the short-range coupling required for Dexter transfer (Fig. 1a)^36–38^. The alkylene straps were installed by either Grubbs metathesis (**NB-1**) or nucleophilic substitution chemistry (**NB-2**) (Fig. 1c). Both routes crucially proceed via intermediates that can be obtained in high purity using conventional laboratory techniques. This was achieved by employing bulky alkene (**NB-1**) or benzyl (**NB-2**) groups to circumvent the poor solubility of the indolocarbazole core. The luminophore cores of **NB-1** and **NB-2** are completely enveloped by a continuous alkylene macrocycle (Supplementary Note 2), in contrast to designs that decorate emitters with peripheral steric bulk^39,40^. Despite high molecular weights (MW = 1,122), **NB-1** and **NB-2** remain sufficiently volatile for processing via thermal evaporation (240–260 °C at 10^−7^ mbar; Supplementary Fig. 58). This suggests a suppression of intermolecular interactions, in line with calculations indicating that the non-conjugated straps of **NB-1** contribute to over half its molecular volume (Supplementary Table 2). Additionally, although the shielding of emitters with side groups could lead to redshifted and broadened emission^1^, we note that our design has a negligible effect on the **mDICz** core photophysics (Supplementary Table 4).

## Organic light-emitting devices

**Fig. 2:**
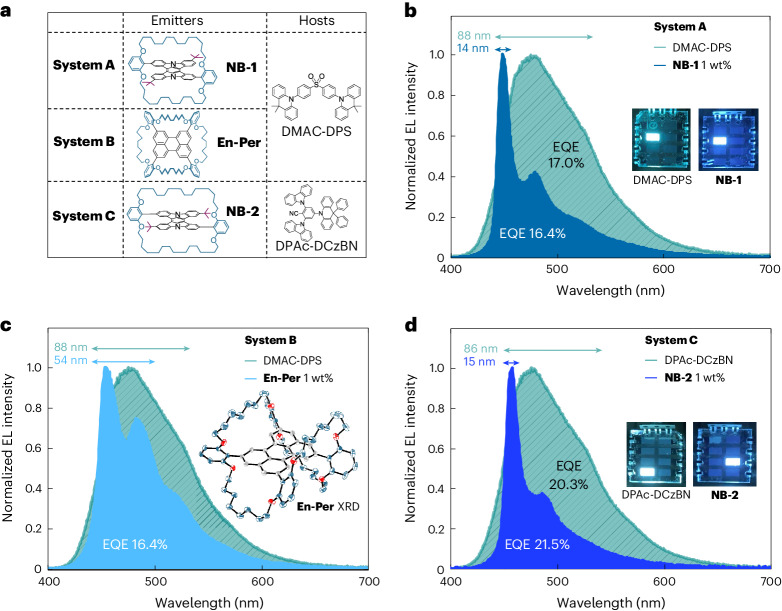
Organic light-emitting devices. **a**, Material combinations for matrix-free **Systems** **A**, **B** and **C**. **b**, EL spectra of **System A**, with photographs of OLED emission shown in the insets. **c**, EL spectra of **System B**, with **En-Per** single-crystal X-ray structure shown in the inset. XRD, X-ray diffraction. **d**, EL spectra of **System C**, with photographs of OLED emission shown in the insets.

OLEDs were fabricated to explore the effect of incorporating encapsulated emitters into MFHF devices (Fig. 2). The EL spectrum of an OLED where the EML consists of a DMAC-DPS host doped with 1 wt% **NB-1** is shown in Fig. 2b, alongside that for a non-doped reference device (**System A**; Fig. 2a). Pertinent device metrics are listed in Table 1. The non-doped DMAC-DPS OLED demonstrates peak EL at 476 nm with a broad FWHM of 88 nm. Owing to singlet energy transfer via FRET, the 1 wt% **NB-1** MFHF device displays a far superior EL spectrum, with a desirable 449 nm peak wavelength and an extremely narrow FWHM of 14 nm. Harmful high-energy emission of <450 nm is also reduced. We note that the incorporation of 1 wt% **NB-1** is accompanied by only a slight decrease in EQE compared with the non-doped device (16.4% versus 17.0%).

**Table 1:** Pertinent device parameters for matrix-free OLEDs

System	EML	EQE_Max_ (%)	EQE_100_^a^ (%)	*λ*_EL_ (nm)	FWHM (nm)	CIE (*x*, *y*)
**A**	DMAC-DPS	17.0	15.8	476	88.3	0.185, 0.315
	DMAC-DPS/**NB-1** 1 wt%	16.4	14.6	449	13.9	0.168, 0.180
**B**	DMAC-DPS/**En-Per** 1 wt%	16.4	15.7	454	54.2	0.164, 0.231
**C**	DPAc-DCzBN	20.3	18.7	476	86.3	0.186, 0.313
	DPAc-DCzBN/**NB-2** 1 wt%	21.5	17.8	458	15.4	0.161, 0.186

^a^Value at 100 cd m^−2^

Perylene, the archetypal blue terminal emitter in hyperfluorescent systems, was also encapsulated to afford **En-Per** (**System B**; Fig. 2a). In an identical fashion to the results for **System A**, the 1 wt% **En-Per** in the DMAC-DPS device affords a maximum EQE of 16.4%, suggesting that encapsulation may be generally applicable to obtain high-MFHF EQEs from different luminophore cores. This efficiency is comparable with the best previously reported values for organic hyperfluorescent systems incorporating perylene terminal emitters, even when matrices are included^3,41–43^.

In **System C** (Fig. 2a), the slightly redshifted emitter **NB-2** exhibits good spectral overlap with the TADF material DPAc-DCzBN (Supplementary Fig. 76), a high-performing emitter in non-doped devices^44^. In analogy to **System A**, the incorporation of 1 wt% **NB-2** into DPAc-DCzBN in **System C** affords a large decrease in FWHM compared with the host-only device (86 → 15 nm) (Fig. 2d). The 1 wt% **NB-2** device also crosses the 20.0% EQE threshold, affording a high maximum EQE of 21.5% comparable with the non-doped device (maximum EQE = 20.3%). This is a 1.5 times improvement over the best industry-published triplet–triplet annihilation (TTA) blue OLEDs^45^. **Systems A** and **C** are the first examples of blue narrowband MFHF OLEDs, which—to the best of our knowledge—display the highest EQEs and lowest CIE_*xy*_ coordinates yet reported for matrix-free systems^36,42^. For reference, an MFHF device based on the DMAC-DPS host and an archetypal bis(diphenylamino)pyrene fluorescent emitter affords a maximum EQE of merely 7% in sky blue (Supplementary Fig. 112)^46,47^. Additionally, even compared with contemporary MCHF and TTA devices, our 14–15 nm FWHM values are record equalling^12,27^. Supplementary Note 7 presents further detailed device characterization results.

An important observation is that for all the three MFHF systems, there is a negligible difference in the OLED EQEs between doped and non-doped devices. The same trend is additionally seen for a further encapsulated structure based on 9,10-diphenylanthracene (Supplementary Table 21). The lack of any substantial decrease in performance implies that the emitters introduce minimal loss pathways to MFHF OLEDs; for example, quenching via Dexter transfer to the terminal emitter may not be substantial, which would validate the encapsulated design. This prompted us to study the energy transfer dynamics of MFHF blends in further detail.

## PL study

**Fig. 3:**
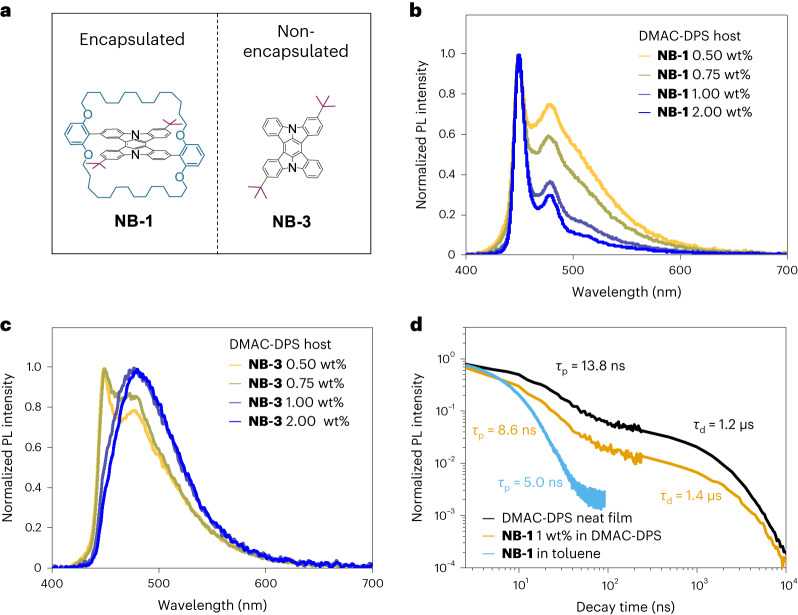
PL. **a**, Molecular structures of **NB-1** and **NB-3**. **b**,**c**, PL spectra of the concentration series of **NB-1** (**b**) and **NB-3** (**c**) in DMAC-DPS. **d**, PL decays for **NB-1** and DMAC-DPS.

To determine the effect of an encapsulated acceptor structure on the energy transfer processes in a TADF host, non-encapsulated analogue **NB-3** (Fig. 3a) was synthesized for comparison with **NB-1** in photophysical studies on **System A**. The PL spectra for the concentration series of **NB-1** and **NB-3** evaporated into DMAC-DPS are shown in Fig. 3, obtained on excitation of the host at 330 nm. Aggregation can plague narrowband emitters, leading to broadened emission and quenched luminescence even in dilute evaporated films^14,27,40,48–51^. For **NB-3**, broad unstructured PL is recorded on increasing the doping concentration (Fig. 3c). In contrast, for **NB-1**, PL in DMAC-DPS progresses towards its narrow-structured solution spectrum (Fig. 3b), indicating that alkylene encapsulation effectively suppresses the aggregation of **mDICz**.

Next, *Φ*_PL_ and time-resolved PL experiments were conducted. Data for the films of **NB-1** and **NB-3** doped into DMAC-DPS are presented in Table 2. In solution, the emitters display only prompt fluorescence with a lifetime (*τ*) of around 5.0 ns (Fig. 3d and Supplementary Fig. 63), in agreement with previous reports for **mDICz** (ref. ^27^), and expectedly shorter than the prompt fluorescence lifetime of pristine DMAC-DPS (13.8 ns). In contrast, when doped into DMAC-DPS, clear biphasic decay is observed, as singlet excitons from the TADF host are transferred to the terminal emitters on both prompt and delayed timescales.

**Table 2:** *Φ*_PL_ and time-resolved PL data with relevant energy transfer parameters

Host	Dopant (1 wt%)	*Φ*_PL_ (±0.05)	*Φ*_Prompt_	*Φ*_Delayed_	*τ*_Prompt_ (ns)	*τ*_Delayed_ (μs)	*k*_FRET_ (10^7 ^s^−1^)	*R*_0_ (nm)	*R*_Average_ (nm)	*k*_DET_ (10^6 ^s^−1^)
DMAC-DPS	**NB-1**	0.63	0.14	0.49	8.57	1.43	1.84	2.88	2.62	2.61
DMAC-DPS	**NB-3**	0.35	0.11	0.24	13.60	1.15	13.77	2.73	1.77	8.25

Although the prompt component of *Φ*_PL_ (*Φ*_Prompt_) for both terminal emitters is similar, the overall *Φ*_PL_ is substantially smaller for **NB-3** compared with **NB-1** due to a large reduction in the delayed PL (*Φ*_Delayed_), as also evidenced by a shorter delayed PL lifetime (*τ*_Delayed_). As triplets are only present in a PL experiment for TADF hosts on the delayed timescale after intersystem crossing, the selective decrease in delayed PL for **NB-3** compared with **NB-1** supports the hypothesis of a triplet-mediated pathway such as Dexter transfer, which is suppressed by the encapsulated structure of **NB-1**. Metrics describing FRET and Dexter transfer were next calculated from the time-resolved PL data (Supplementary Note 5). Crucially, in DMAC-DPS, the rate of Dexter transfer (*k*_DET_) for **NB-1** is reduced by over a factor of three (3.2) compared with **NB-3** at the same wt% doping, supporting the fact that encapsulation can suppress Dexter transfer. Parameters such as FRET rate (*k*_FRET_), Förster radius (*R*_0_) and average donor–acceptor distance (*R*_Average_) also suggest that FRET is more efficient for **NB-3** than **NB-1**, in line with the encapsulated structure of **NB-1** increasing the average intermolecular distances.

## TA study

**Fig. 4:**
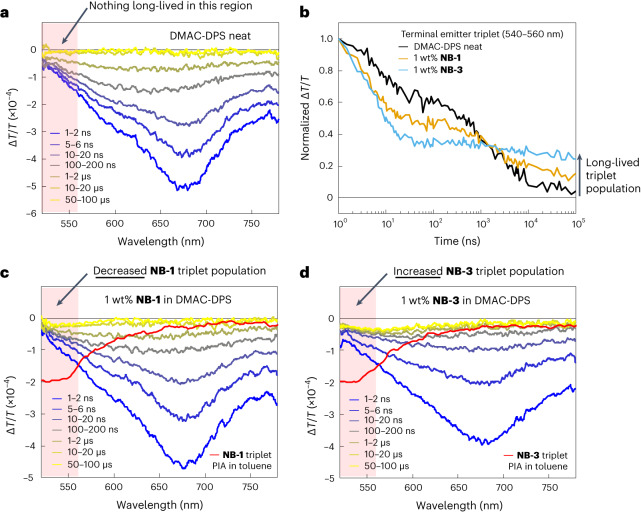
TA. **a**, Long-time TA spectra for DMAC-DPS. **b**, Normalized decays in the PIA region of terminal emitter triplets for doped films and neat DMAC-DPS. **c**, Long-time TA spectra for DMAC-DPS:1 wt% **NB-1**. **d**, Long-time TA spectra for DMAC-DPS:1 wt% **NB-3**.

Although supportive of the hypothesis that the encapsulated structure of **NB-1** can suppress Dexter transfer to a terminal emitter from a TADF host, calculations based on time-resolved PL data invariably require assumptions stemming from the dark nature of organic triplet states. TA spectroscopy is not restricted to bright states, and therefore, we turned to this technique in the hope of directly observing Dexter transfer to further understand the energy transfer dynamics in DMAC-DPS-hosted films. Experiments were pumped at 355 or 400 nm. The TA spectra of the diluted emitters and a neat film of DMAC-DPS were first assigned (Fig. 4a and Supplementary Figs. 92–97). In a toluene solution, **NB-1** and **NB-3** display very similar spectra: photoinduced absorption (PIA) centred at around 480 nm, which decays with a lifetime of around 10 ns, is assigned to S_1_ → S_*n*_, whereas a slower-rising PIA centred between 540 and 560 nm with a lifetime of tens of microseconds and clear oxygen quenching is assigned to T_1_ → T_*n*_ (Supplementary Figs. 92–97). A neat film of DMAC-DPS displays a broad PIA between 600 and 700 nm when probed on timescales between 0.2 ps and 100 μs (Fig. 4a and Supplementary Fig. 101). The kinetics roughly fit biphasic prompt-delayed behaviour, as previously reported for TADF materials^52^ (Fig. 4b and Supplementary Fig. 104). Fortunately, the PIA of DMAC-DPS has poor overlap with the T_1_ PIA of the emitters, displaying essentially no long-lived signal of ≤550 nm, which we envisaged should make it possible to directly observe Dexter transfer in doped films.

Next, 1 wt% **NB-1** and **NB-3** films in DMAC-DPS were investigated. Owing to the low concentration of the dopants, essentially an exclusive excitation of the DMAC-DPS host is expected. Short-time experiments (<2 ns) indicate a faster initial decay of the DMAC-DPS PIA for the **NB-3**-doped film compared with **NB-1**, in agreement with the larger *k*_FRET_ calculated for **NB-3** above (Supplementary Fig. 102). Microsecond-timescale experiments were next performed to probe delayed processes (Fig. 4c,d). Normalized decays of the 540–560 nm region for the doped films are shown in Fig. 4b, with neat DMAC-DPS for comparison. For the **NB-3**-doped film, the prompt-delayed biphasic kinetics of DMAC-DPS are substantially quenched when probed at either 540–560 nm or 600–700 nm (Fig. 4b and Supplementary Fig. 108), suggesting that delayed fluorescence, and perhaps T → S reverse intersystem crossing, are suppressed (in agreement with the relatively low *Φ*_Delayed_ value of the **NB-3**-doped film). Meanwhile, the 540–560 nm PIA levels out within *~*200 ns and persists onto the microsecond timescale at an intensity appreciably larger than that for neat DMAC-DPS, consistent with terminal emitter triplets. Although it is plausible to simply ascribe delayed fluorescence quenching to FRET^20^, this mechanism cannot explain the concomitant population of the terminal emitter triplet state, implying a different quenching pathway. Such a result is consistent with what would be expected for Dexter transfer, which—to the best of our knowledge—is being directly observed for the first time in a hyperfluorescent system.

After validating the potential of TA for directly observing Dexter transfer with **NB-3**-doped films, DMAC-DPS films doped with the encapsulated **NB-1** emitter were investigated. For **NB-1**-doped films, the prompt-delayed biphasic kinetics of DMAC-DPS are more strongly retained than for **NB-3** (Fig. 4b and Supplementary Fig. 106), in agreement with the higher *Φ*_Delayed_ mentioned above. The more appreciable delayed fluorescence of **NB-1**-doped films is accompanied by a weaker long-lived 540–560 nm PIA compared with **NB-3**, suggesting a lower population of terminal emitter triplets. Hence, TA provides direct evidence that the encapsulated molecular structure of **NB-1** can appreciably suppress loss via Dexter transfer to terminal emitter triplets in a pristine TADF host, supporting the minimal OLED EQE drop encountered for the MFHF OLED.

## Outlook

Current blue hyperfluorescence technology requires OLEDs with complex EMLs of at least three components—a TADF sensitizer, narrowband emitter guest and wide-gap matrix (matrices). High triplet energy matrices have been considered essential to suppress Dexter transfer to the terminal emitter towards high device efficiency and stability.

Here we developed ultranarrowband blue emitters encapsulated by bulky alkylene straps. The ideal spectral properties of the emitters facilitates efficient FRET of singlets from pristine blue TADF hosts. Through comparison with a non-encapsulated analogue using TA spectroscopy, the population of terminal emitter triplets via a Dexter mechanism was directly observed in a hyperfluorescent system for the first time, enabling us to reveal that encapsulating straps can efficiently suppress the Dexter loss pathway to terminal emitter triplets.

Consequently, the new molecular design circumvents the need for a matrix, facilitating efficient blue MFHF OLEDs, with EMLs consisting of TADF sensitizer hosts and encapsulated terminal emitters only. Owing to the efficient suppression of Dexter transfer, MFHF OLEDs demonstrated negligible drops in EQE compared with non-doped reference devices, with a maximum EQE of 21.5%. Simultaneously, the EL FWHM of devices was decreased by a factor of six compared with the non-doped devices from >80 nm to ultranarrow values of 14–15 nm, with desirable deep blue peak wavelengths (449 and 458 nm). Non-doped deep blue TADF research is steadily progressing^53–55^, and with improved TADF hosts, we envisage that this encapsulated ultranarrowband emitter design should enable efficient MFHF OLEDs satisfying BT.2020, similar to the CIE_*y*_ ≤ 0.05 that can be obtained in TTA-sensitized devices with the **NB-1** emitter (Supplementary Table 16). More broadly, it has been suggested that the unsatisfactory stability of current blue MCHF OLEDs is directly linked to the population of terminal emitter triplets^21^, making the new strategy outlined here for suppressing Dexter transfer highly relevant.

## Methods

### Molecular synthesis

Supplementary Note 1 discusses the synthesis routes.

**S1**. This synthesis was inspired by procedures in the literature^26,56^. 5-Bromoindole (4.07 g, 20.7 mmol, 1.00 eq.), 2-bromo-4-*tert*-butylbenzaldehyde (5 g, 20.7 mmol, 1.00 eq.) and HI (57 wt% in water, 0.56 ml, 4.23 mmol, 0.20 eq.) were heated overnight to reflux under air in MeCN (150 ml). The reaction mixture was cooled in a −20 °C freezer for 6 h and the precipitate was isolated by filtration and washed with −20 °C MeCN (3 × 20 ml). The isolated solid was suspended in MeCN (150 ml) with iodine (1.95 g, 7.69 mmol, 0.37 eq.), and the resulting mixture heated overnight again to reflux under air. The reaction mixture was cooled in a −20 °C freezer for 6 h and the presumed indolocarbazole intermediate was isolated as a yellow-white powder by filtration after washing with −20 °C MeCN (3 × 20 ml) and drying under suction (3.74 g, 4.47 mmol, 43%). The presumed indolocarbazole intermediate was suspended in dry DMF (150 ml) with TBAOH (40 wt% in methanol, 6.50 g, 11.00 mmol, 2.46 eq.) under argon. The resulting mixture was degassed for 10 min. CuI (852 mg, 4.47 mmol, 1.00 eq.) was added and the mixture was immersed in a 120 °C preheated oil bath overnight. The reaction mixture was cooled to room temperature. The yellow precipitate was isolated via filtration, washed with MeCN (3 × 20 ml) and then suspended in benzonitrile (100 ml). The suspension was refluxed for 10 min and then cooled to room temperature. Filtration afforded **S1** as a yellow powder sufficiently pure for the next step (870 mg, 1.29 mmol, 12% overall yield). This compound is too insoluble to obtain the nuclear magnetic resonance (NMR) spectra in common organic solvents. An analytical sample was obtained as fluffy yellow needles after recrystallization from benzonitrile (reflux → room temperature, hot filtered through paper under gravity). The ultraviolet spectra of the crude and recrystallized samples are essentially identical. Anal. Calcd. for C_38_H_30_Br_2_N_2_: C, 67.67; H, 4.48; N, 4.15. Calcd. for C_38_H_30_Br_2_N_2_·0.2PhCN: C, 68.08; H, 4.50; N, 4.43. Found: C, 68.09; H, 4.49; N, 4.39 (average of two runs); HRMS (ASAP): *m*/*z* 673.0832 [M–H^+^]. Calcd. for C_38_H_31_Br^79^_2_N_2_^+^: 673.0854.

**S2**. 2-Bromoresorcinol (2.90 g, 15.3 mmol, 1.00 eq.), K_2_CO_3_ (12.67 g, 91.7 mmol, 6.00 eq.) and KI (762 mg, 4.59 mmol, 0.30 eq.) were combined in dry DMF (50 ml) under argon and the resulting mixture was heated in a 60 °C oil bath. After 1 h, 8-bromo-1-octene (7.32 g, 38.3 mmol, 2.50 eq.) was added and the heating was continued overnight. The reaction mixture was cooled to room temperature and diluted with hexane (100 ml) and water (300 ml). The layers were separated, and the aqueous layer was extracted with further hexane (3 × 50 ml). The extracts were combined, washed with 2 M NaOH (3 × 50 ml), dried over MgSO_4_, filtered and the solvent was removed under reduced pressure. The residue was purified by flash chromatography on silica gel (eluent:gradient, 0:1–1:9 EtOAc/*n*-hexane v/v) to afford **S2** as a clear oil (5.84 g, 14.3 mmol, 93%). ^1^H NMR data were in accordance with those reported in the literature^57^. ^1^H NMR (500 MHz, CDCl_3_) δ (ppm) = 7.18 (t, *J* = 8.3 Hz, 1H), 6.55 (d, *J* = 8.3 Hz, 2H), 5.84 (ddt, *J* = 17.1, 10.2, 6.7 Hz, 2H), 5.03 (ddd, *J* = 17.1, 3.5, 1.7 Hz, 2H), 4.96 (ddt, *J* = 10.2, 2.2, 1.7 Hz, 2H), 4.04 (t, *J* = 6.5 Hz, 4H), 2.13–2.05 (m, 4H), 1.86 (dq, *J* = 13.0, 6.5 Hz, 4H), 1.58–1.50 (m, 5H), 1.47–1.39 (m, 8H).

**S3**. A solution of **S2** (5.81 g, 14.2 mmol, 1.00 eq.) in dry tetrahydrofuran (THF) (80 ml) was cooled to −78 °C in a dry ice–acetone bath under argon. *n*-BuLi (2.5 M in hexane, 7.37 ml, 18.4 mmol, 1.30 eq.) was added dropwise and the resulting mixture was stirred for 1 h. B(OMe)_3_ (5.54 ml, 49.7 mmol, 3.50 eq.) was added dropwise, and the resulting mixture was warmed overnight to room temperature. The reaction mixture was quenched with 1 M HCl (30 ml) and stirred for 1 h. The layers were separated and the aqueous layer was extracted with dichloromethane (DCM) (3 × 50 ml). The extracts were combined, dried over MgSO_4_, filtered and the solvent was removed under reduced pressure. The residue was purified by flash chromatography on silica gel (eluent:gradient, 0:1–1:0 DCM/*n*-hexane v/v) to afford **S3** as a clear oil that solidified on standing (4.52 g, 12.1 mmol, 85%). ^1^H NMR data were in accordance with those reported in the literature^57^. ^1^H NMR (500 MHz, CDCl_3_) δ (ppm) = 7.38–7.31 (m, 3H), 6.60 (d, *J* = 8.4 Hz, 2H), 5.80 (ddt, *J* = 17.1, 10.2, 6.7 Hz, 2H), 5.00 (ddd, *J* = 17.1, 3.6, 1.6 Hz, 2H), 4.94 (ddt, *J* = 10.2, 2.2, 1.2 Hz, 2H), 4.06 (t, *J* = 6.6 Hz, 4H), 2.06 (td, *J* = 6.8, 1.2 Hz, 4H), 1.85 (dq, *J* = 13.2, 6.6 Hz, 4H), 1.51–1.36 (m, 12H).

**S4**. **S1** (1.00 g, 1.48 mmol, 1.00 eq.), **S3** (3.33 g, 8.90 mmol, 6.00 eq.), Pd(OAc)_2_ (20 mg, 88.9 μmol, 6 mol%) and SPhos (73 mg, 178 μmol, 12 mol%) were combined in a 500 ml round-bottomed flask that was evacuated and backfilled with argon five times. Degassed toluene (250 ml, with five drops of Aliquat 336) and a degassed solution of K_3_PO_4_ (1.89 g, 8.90 mmol, 6.00 eq.) in water (32 ml) were sequentially added, and the resulting mixture was refluxed overnight in a preheated 130 °C oil bath. The reaction mixture was cooled to room temperature and acidified with 1 M HCl (100 ml). The layers were separated, and the aqueous layer was extracted with DCM (3 × 50 ml). The extracts were combined, dried over MgSO_4_, filtered and the solvent was removed under reduced pressure. The residue was purified by flash chromatography on silica gel (eluent:gradient, 0:1–35:65 DCM/*n*-hexane v/v) and then recrystallized (100 ml ethanol and minimal CHCl_3_, reflux → −20 °C) to afford **S4** as yellow needles (950 mg, 0.81 mmol, 55%). ^1^H NMR (400 MHz, CDCl_3_) δ (ppm) = 8.59 (s, 2H), 8.42 (d, *J* = 8.3 Hz, 2H), 8.13–8.05 (m, 4H), 7.67 (d, *J* = 8.3 Hz, 2H), 7.49 (d, *J* = 8.2 Hz, 2H), 7.34 (t, *J* = 8.3 Hz, 2H), 6.77 (d, *J* = 8.3 Hz, 4H), 5.52–5.39 (m, 4H), 4.73–4.62 (m, 8H), 4.00 (t, *J* = 6.3 Hz, 8H), 1.75–1.67 (m, 8H), 1.66–1.58 (m, 8H), 1.55 (s, 18H), 1.33–1.24 (m, 8H), 1.13–0.98 (m, 16H); ^13^C NMR (101 MHz, CDCl_3_) δ (ppm) = 157.6, 150.1, 142.5, 139.2, 138.8, 137.6, 129.6, 128.9, 128.5, 127.5, 127.4, 126.9, 124.0, 120.9, 119.1, 113.8, 112.4, 112.0, 111.1, 109.1, 105.8, 68.9, 35.4, 33.5, 31.8, 29.2, 28.7, 28.6, 25.9; Anal. Calcd. for C_82_H_96_N_2_O_4_: C, 83.92; H, 8.24; N, 2.39. Found: C, 83.97; H, 8.14; N, 2.34 (average of two runs); HRMS (ASAP): *m*/*z* 1,173.7407 [M–H^+^]. Calcd. for C_82_H_97_N_2_O_4_^+^: 1,173.7448.

**S5**. **S4** (800 mg, 0.68 mmol) and Grubbs second-generation catalyst (36 mg, 41 μmol, 6 mol%) were stirred overnight in degassed dry DCM (200 ml) under argon at reflux. The solvent was evaporated and the residue was purified by flash chromatography on silica gel (eluent:gradient, 0:1–35:65 DCM/*n*-hexane v/v, a single yellow band was collected) to afford **S5** as a yellow powder sufficiently pure for the next step after trituration with methanol and drying under suction (753 mg, 0.66 mmol, 96%), yielding a mixture of E and Z isomers. ^1^H NMR (400 MHz, CDCl_3_) δ (ppm) = 8.60 (s, 2H), 8.43 (d, *J* = 8.2 Hz, 2H), 8.18–8.08 (m, 4H), 7.76–7.67 (m, 2H), 7.50 (d, *J* = 8.2 Hz, 2H), 7.37 (t, *J* = 8.3 Hz, 2H), 6.80 (d, *J* = 8.3 Hz, 4H), 4.19–4.02 (m, 8H), 4.00–3.91 (m, 4H), 1.62–1.53 (m, 8H), 1.32–1.08 (m, 16H), 0.96–0.83 (m, 8H), 0.62–0.42 (m, 8H); ^13^C NMR (176 MHz, CDCl_3_) δ (ppm) 157.8, 150.2, 142.6, 139.4, 137.6, 129.9, 129.1, 128.9, 128.7, 127.6, 127.5, 127.1, 124.4, 121.4, 119.1, 112.3, 112.0, 111.3, 109.2, 106.0, 69.2, 35.5, 32.2, 32.0, 29.8, 29.6, 29.3, 26.6; Anal. Calcd. for C_78_H_88_N_2_O_4_: C, 83.83; H, 7.94; N, 2.51. Found: C, 83.69; H, 7.94; N, 2.49 (average of two runs); HRMS (ASAP): *m*/*z* 1,117.6803 [M–H^+^]. Calcd. for C_78_H_89_N_2_O_4_^+^: 1,117.6822. Shoulder peaks in the ^1^H NMR spectrum are assigned to a mixture of E and Z isomers, considering that no shoulder peaks are present after alkene reduction in the next step. Shoulders are also present in the ^13^C NMR spectrum. Only peaks for the major compound are listed.

**NB-1**. A mixture of **S5** (710 mg, 0.64 mmol) and Pd/C (10 wt% Pd, 150 mg) in THF (125 ml) was bubbled with H_2_ for 30 min at room temperature and then refluxed overnight under H_2_ (balloon). The warm reaction mixture was filtered through celite and the solvent was removed under reduced pressure. The residue was recrystallized (100 ml ethanol and minimal CHCl_3_, reflux → −20 °C) to obtain **NB-1** as a yellow powder (610 mg, 0.54 mmol, 86%). ^1^H NMR (400 MHz, CDCl_3_) δ (ppm) = 8.61 (s, 2H), 8.47–8.37 (m, 2H), 8.15–8.01 (m, 4H), 7.70 (d, *J* = 8.2 Hz, 2H), 7.47 (d, *J* = 8.1 Hz, 2H), 7.34 (t, *J* = 8.3 Hz, 2H), 6.78 (t, *J* = 8.3 Hz, 4H), 4.09–3.92 (m, 8H), 1.56 (s, 18H), 1.35–1.18 (m, 8H), 0.94–0.80 (m, 8H), 0.56–0.32 (m, 16H), 0.21 to −0.02 (m, 8H); ^13^C NMR (126 MHz, CDCl_3_) δ (ppm) = 157.8, 150.1, 142.5, 139.3, 137.5, 130.0, 128.9, 128.6, 127.6, 127.5, 127.1, 124.5, 120.9, 119.0, 112.3, 112.0, 111.2, 109.3, 105.7, 69.2, 35.5, 32.0, 29.9, 29.8, 29.8, 29.6, 29.3, 26.9; Anal. Calcd. for C_78_H_92_N_2_O_4_: C, 83.53; H, 8.27; N, 2.50. Found: C, 83.39; H, 8.26; N, 2.50 (average of two runs); HRMS (ASAP): *m*/*z* 1,121.7126 [M–H^+^]. Calcd. for C_78_H_93_N_2_O_4_^+^: 1,121.7135.

**S6**. This synthesis was inspired by procedures in the literature^26,56^. 5-Bromoindole (4.07 g, 20.7 mmol, 1.00 eq.), 2-bromo-5-*tert*-butylbenzaldehyde (5 g, 20.7 mmol, 1.00 eq.) and HI (57 wt% in water, 0.56 ml, 4.23 mmol, 0.20 eq.) were heated overnight to reflux under air in MeCN (50 ml). The reaction mixture was cooled in a −20 °C freezer for 6 h and the presumed indolocarbazole intermediate was isolated as a yellow-white powder by filtration after washing with −20 °C MeCN (3 × 20 ml) and drying under suction (3.29 g, 3.93 mmol, 38%). Aromatization with I_2_ was not carried out during the synthesis of **S6** as it caused the yield to drop by a factor of nearly five. The presumed indolocarbazole intermediate was suspended in dry DMF (100 ml) with TBAOH (40 wt% in methanol, 5.72 g, 9.68 mmol, 2.46 eq.) under argon. The resulting mixture was degassed for 10 min. CuI (750 mg, 3.93 mmol, 1.00 eq.) was added and the mixture was immersed overnight in a 120 °C preheated oil bath. The reaction mixture was cooled to room temperature. The yellow precipitate was isolated via filtration, washed with MeCN (3 × 20 ml) and then suspended in benzonitrile (50 ml). The suspension was refluxed for 10 min and then cooled to room temperature. Filtration afforded **S6** as a yellow powder sufficiently pure for the next step (730 mg, 1.08 mmol, 10% overall yield). This compound is too insoluble to obtain the NMR spectra in common organic solvents. HRMS (ASAP): *m*/*z* 673.0826 [M–H^+^]. Calcd. for C_38_H_31_Br^79^_2_N_2_^+^: 673.0854.

**S7**. 2-Bromoresorcinol (13.6 g, 71.6 mmol, 1.00 eq.), K_2_CO_3_ (21.8 g, 158 mmol, 2.20 eq.) and KI (1.36 mg, 8.16 mmol, 0.11 eq.) were combined in dry acetone (100 ml) under argon. Benzyl bromide (19.0 ml, 160 mmol, 2.23 eq.) was added and the resulting mixture was heated overnight to reflux. The reaction mixture was cooled to room temperature and filtered through celite eluting with additional acetone (500 ml). The solvent was removed under reduced pressure and the residue was recrystallized from hexane (700 ml, reflux → room temperature) to afford **S7** as colourless needles (21.3 g, 57.9 mmol, 81%). ^1^H NMR data were in accordance with those reported in the literature^58^. ^1^H NMR (400 MHz, CDCl_3_) δ (ppm) = 7.49 (d, *J* = 7.4 Hz, 4H), 7.41–7.36 (m, 4H), 7.34–7.30 (m, 2H), 7.15 (t, *J* = 8.3 Hz, 1H), 6.61 (d, *J* = 8.3 Hz, 2H), 5.17 (s, 4H).

**S8**. A solution of **S7** (9.44 g, 25.6 mmol, 1.00 eq.) in dry THF (200 ml) was cooled to −78 °C in a dry ice–acetone bath under argon. *n*-BuLi (2.5 M in hexane, 13.3 ml, 33.3 mmol, 1.30 eq.) was added dropwise and the resulting mixture was stirred for 1 h. The mixture set to a solid that could not be stirred. The mixture was transferred to an ice bath until full dissolution was observed, and then transferred back into a dry ice–acetone bath. B(OMe)_3_ (9.92 ml, 89.0 mmol, 3.48 eq.) was immediately added dropwise, and the resulting mixture was warmed overnight to room temperature. The reaction mixture was quenched with 1 M HCl (30 ml) and stirred for 1 h. The layers were separated and the aqueous layer was extracted with DCM (3 × 50 ml). The extracts were combined, dried over MgSO_4_, filtered and the solvent removed under reduced pressure. The residue was purified by flash chromatography on silica gel (eluent:gradient, 0:1–1:0 EtOAc/DCM v/v) to afford **S8** as an off-white solid (6.84 g, 20.5 mmol, 80%). ^1^H NMR data were in accordance with those reported in the literature^58^. ^1^H NMR (400 MHz, CDCl_3_) δ (ppm) = 7.45–7.33 (m, 11H), 7.20 (s, 2H), 6.71 (d, *J* = 8.4 Hz, 2H), 5.15 (s, 4H).

**S9**. **S6** (400 mg, 0.59 mmol, 1.00 eq.), **S8** (1.00 g, 3.00 mmol, 5.08 eq.), Pd(OAc)_2_ (12 mg, 54 μmol, 9 mol%) and SPhos (44 mg, 107 μmol, 18 mol%) were combined in a 250 ml round-bottomed flask that was evacuated and backfilled with argon five times. Degassed toluene (100 ml with three drops of Aliquat 336) and a degassed solution of K_3_PO_4_ (637 mg, 3.00 mmol, 5.08 eq.) in water (13 ml) were sequentially added, and the resulting mixture was refluxed overnight in a preheated 130 °C oil bath. The reaction mixture was cooled to room temperature and acidified with 1 M HCl (100 ml). The layers were separated, and the aqueous layer was extracted with DCM (3 × 50 ml). The extracts were combined, dried over MgSO_4_, filtered and the solvent was removed under reduced pressure. The residue was purified by flash chromatography on silica gel (eluent:gradient, 0:1−3:1 DCM/*n*-hexane v/v) and then recrystallized (100 ml ethanol and minimal CHCl_3_, reflux → room temperature) to afford **S9** as yellow crystals (360 mg, 0.33 mmol, 56%). ^1^H NMR (400 MHz, CDCl_3_) δ (ppm) = 8.82 (s, 2H), 8.53 (s, 2H), 8.11 (d, *J* = 8.2 Hz, 2H), 8.02 (d, *J* = 8.3 Hz, 2H), 7.83 (d, *J* = 8.3 Hz, 2H), 7.65 (d, *J* = 8.4 Hz, 2H), 7.34 (t, *J* = 6.8 Hz, 10H), 7.10–7.02 (m, 12H), 6.88 (d, *J* = 8.4 Hz, 4H), 5.17 (s, 4H), 1.40 (s, 18H); ^13^C NMR (126 MHz, CDCl_3_) δ (ppm) = 157.3, 144.6, 142.7, 137.8, 137.3, 136.9, 130.3, 129.2, 128.8, 128.7, 128.5, 127.8, 127.5, 127.1, 126.7, 124.2, 121.5, 121.2, 112.8, 112.7, 111.8, 111.4, 107.1, 70.9, 34.9, 31.9; Anal. Calcd. for C_78_H_64_N_2_O_4_: C, 85.68; H, 5.90; N, 2.56. Found: C, 85.55; H, 5.79; N, 2.95 (average of two runs); HRMS (ASAP): *m*/*z* 1,093.4896 [M–H^+^]. Calcd. for C_78_H_65_N_2_O_4_^+^: 1,093.4944.

**S10**. **S9** (250 mg, 229 μmol), Pd/C (10 wt% Pd, 78 mg), THF (30 ml) and ethanol (5 ml) were sealed in a 50 ml microwave vial with a crimp septum cap. The resulting mixture was bubbled with H_2_ gas via a balloon for *~*30 min. The balloon was removed and the vial was stirred overnight in a preheated 80 °C oil bath. The reaction mixture was cooled to room temperature, diluted with THF (50 ml) and filtered through a plug of silica gel (eluent, *~*100 ml THF). The solvent was removed and the residue was triturated with hexane and filtered to obtain **S10** as a yellow powder that was used as obtained in the next step after drying under a high vacuum (168 mg, 229 μmol, 100%). ^1^H NMR (400 MHz, THF) δ (ppm) = 8.81 (d, *J* = 1.1 Hz, 2H), 8.67 (d, *J* = 1.7 Hz, 2H), 8.23 (d, *J* = 8.4 Hz, 2H), 8.18 (d, *J* = 8.5 Hz, 2H), 7.95 (s, 4H), 7.81 (dd, *J* = 8.3, 1.5 Hz, 2H), 7.76 (dd, *J* = 8.5, 1.8 Hz, 2H), 6.99 (t, *J* = 8.1 Hz, 2H), 6.49 (d, *J* = 8.1 Hz, 4H); ^13^C NMR (176 MHz, THF) δ (ppm) = 158.4, 147.0, 144.6, 139.8, 139.3, 132.6, 131.5, 131.0, 130.4, 130.2, 129.8, 126.5, 123.2, 118.8, 114.8, 114.8, 114.0, 113.7, 36.9, 33.7; HRMS (ESI): *m*/*z* 733.3053 [M–H^+^]. Calcd. for C_50_H_41_N_2_O_4_^+^: 733.3066.

**NB-2**. A mixture of **S10** (151 mg, 206 μmol, 1.00 eq.) and 1,14-dibromotetradecane (147 mg, 412 206 μmol, 2.00 eq.) was heated to 40 °C in dry DMF (50 ml) under argon until full dissolution was observed (*~*30 min). K_2_CO_3_ (142 mg, 1.03 mmol, 5.00 eq.) was added, and the mixture was stirred at 40 °C for 24 h and then at 80 °C for 48 h. After evaporation of the reaction solvent, the residue was purified by flash chromatography on silica gel (eluent:gradient, 0:1−1:1 DCM/*n*-hexane v/v, the product is the first fluorescent band to elute and drags, use a small column) to afford **NB-2** as a yellow powder after trituration with methanol and drying under suction (162 mg, 144 μmol, 70%). ^1^H NMR (400 MHz, CDCl_3_) δ (ppm) = 8.66 (d, *J* = 1.2 Hz, 2H), 8.55 (d, *J* = 1.7 Hz, 2H), 8.07 (d, *J* = 8.3 Hz, 2H), 8.00 (d, *J* = 8.5 Hz, 2H), 7.75 (dd, *J* = 8.3, 1.5 Hz, 2H), 7.68 (dd, *J* = 8.5, 1.8 Hz, 2H), 7.32 (t, *J* = 8.3 Hz, 2H), 6.75 (d, *J* = 8.4 Hz, 4H), 4.08 (dt, *J* = 8.9, 4.5 Hz, 4H), 4.00–3.91 (m, 4H), 1.64–1.55 (m, 8H), 1.53 (s, 18H), 1.44–1.23 (m, 12H), 0.89 (dd, *J* = 13.6, 6.9 Hz, 8H), 0.48–0.37 (m, 12H), 0.28–0.18 (m, 4H), 0.06 (d, *J* = 13.0 Hz, 4H); ^13^C NMR (126 MHz, CDCl_3_) δ (ppm) = 157.6, 144.3, 142.3, 137.5, 136.9, 130.3, 129.5, 128.6, 128.5, 128.0, 127.2, 124.1, 121.5, 120.3, 112.7, 112.5, 111.8, 111.0, 105.3, 69.0, 35.0, 32.2, 30.1, 29.9, 29.8, 29.7, 29.3, 26.9; Anal. Calcd. for C_78_H_92_N_2_O_4_: C, 83.53; H, 8.27; N, 2.50. Found: C, 83.37; H, 8.30; N, 2.74; HRMS (ASAP): *m*/*z* 1,121.7108 [M–H^+^]. Calcd. for C_78_H_93_N_2_O_4_^+^: 1,121.7135.

**NB-3**. This synthesis was inspired by procedures in the literature^26,56^. Indole (1.94 g, 16.6 mmol, 1.00 eq.), 2-bromo-4-*tert*-butylbenzaldehyde (4.00 g, 16.6 mmol, 1.00 eq.) and HI (57 wt% in water, 0.45 ml, 3.40 mmol, 0.20 eq.) were heated overnight to reflux under air in MeCN (50 ml). The reaction mixture was cooled in a −20 °C freezer for 6 h and the precipitate was isolated by filtration and washed with −20 °C MeCN (3 × 20 ml). The isolated solid was suspended in MeCN (50 ml) with iodine (1.56 g, 6.15 mmol, 0.37 eq.) and the resulting mixture was heated overnight again to reflux under air. The reaction mixture was cooled in a −20 °C freezer for 6 h and the presumed indolocarbazole intermediate was isolated as an off-white powder by filtration after washing with −20 °C MeCN (3 × 20 ml) and drying under suction (890 mg, 1.31 mmol, 16%). The presumed indolocarbazole intermediate was suspended in dry DMF (20 ml) with TBAOH (40 wt% in methanol, 1.89 g, 3.20 mmol, 2.44 eq.) under argon. The resulting mixture was degassed for 10 min. CuI (280 mg, 1.47 mmol, 1.12 eq.) was added and the mixture immersed overnight in a 120 °C preheated oil bath. The reaction mixture was cooled to room temperature. The yellow precipitate was isolated via filtration, washed with MeCN (3 × 20 ml) and recrystallized (100 ml ethanol and minimal CHCl_3_, reflux → room temperature, hot filtered through paper under gravity) to afford **NB-3** as fluffy yellow needles (133 mg, 0.26 mmol, 2% overall yield). ^1^H NMR (400 MHz, TCE) δ (ppm) = 8.28 (d, *J* = 7.7 Hz, 2H), 8.23 (d, *J* = 8.3 Hz, 2H), 7.94–7.88 (m, 4H), 7.64 (td, *J* = 7.7, 1.2 Hz, 2H), 7.57 (dd, *J* = 8.3, 1.7 Hz, 2H), 7.48 (td, *J* = 7.6, 1.0 Hz, 2H), 1.62 (s, 18H); ^13^C NMR (176 MHz, TCE) δ (ppm) = 150.3, 141.5, 138.6, 138.1, 128.6, 126.2, 126.1, 124.1, 123.8, 121.5, 119.4, 112.2, 111.3, 111.2, 109.1, 35.2, 31.7; Anal. Calcd. for C_38_H_32_N_2_: C, 88.34; H, 6.24; N, 5.42. Calcd. for C_38_H_32_N_2_·0.15CHCl_3_: C, 85.80; H, 6.02; N, 5.40. Found: C, 85.71; H, 6.06; N, 5.24 (average of two runs); HRMS (ASAP): *m*/*z* 517.2625 [M–H^+^]. Calcd. for C_38_H_33_N_2_^+^: 517.2624.

**S11**. In an argon glovebox, perylene (5.00 g, 19.4 mmol, 1.00 eq.), bis(pinacolato)diboron (21.87 g, 85.3 mmol, 4.39 eq.), [Ir(COD)OMe]_2_ (306 mg, 0.46 mmol, 2.4 mol%) and 4,4′-di-*tert*-butyl-2,2′-bipyridine (260 mg, 0.92 mmol, 4.7 mol%) were sealed in a 120 ml pressure tube with a Young’s tap having a side port (FengTecEx, product no. P260006). Outside the glovebox, anhydrous degassed THF (60 ml) was added and the tube was heated overnight in an 80 °C oil bath. Initially, everything dissolved on heating before a yellow precipitate slowly formed, which eventually prevented stirring and set to a yellow mass overnight. After the mixture was cooled to room temperature, it was carefully slurried into *~*300 ml methanol (warning: H_2_ evolution). The precipitate was filtered and washed with further methanol (*~*300 ml) to afford **S11** as a yellow solid sufficiently pure for the next step after drying under suction (14.5 g, 19.2 mmol, 99%). ^1^H NMR data were in accordance with those reported in the literature^59^. ^1^H NMR (500 MHz, CDCl_3_) δ (ppm) = 8.62 (s, 4H), 8.24 (s, 4H), 1.43 (s, 48H).

**S12**. **S11** (2.00 g, 2.64 mmol, 1.00 eq.), **S7** (9.75 g, 26.4 mmol, 10.00 eq.), Pd(OAc)_2_ (60 mg, 0.27 mmol, 10 mol%) and SPhos (217 mg, 0.53 mmol, 20 mol%) were combined in a 250 ml round-bottomed flask that was evacuated and backfilled with argon five times. Degassed toluene (100 ml with five drops Aliquat 336) and a degassed solution of K_3_PO_4_ (4.50 g, 21.1 mmol, 8.00 eq.) in water (20 ml) were sequentially added, and the resulting mixture was refluxed overnight in a preheated 130 °C oil bath. The reaction mixture was cooled to room temperature and acidified with 1 M HCl (100 ml). The layers were separated, and the aqueous layer was extracted with toluene (1 × 50 ml). The extracts were combined, dried over Na_2_SO_4_, filtered and the solvent was removed under reduced pressure. The residue was purified by flash chromatography on silica gel (eluent:gradient, 0:1−70:30 DCM/*n*-hexane v/v). The fractions predominantly consisting of the product (the main fluorescent blue band observed in the crude reaction mixture) by thin-layer chromatography were combined and recrystallized (200 ml ethanol and minimal CHCl_3_, reflux → room temperature) to afford **S12** as shiny yellow flakes after drying under suction (1.43 g). The filtrate was combined with impure fractions from the first column and further purified similarly by flash chromatography (eluent:gradient, 0:1−70:30 DCM/*n*-hexane v/v) and recrystallization (200 ml ethanol and minimal CHCl_3_, reflux → room temperature) to afford a second crop of comparable purity (970 mg). Total yield (2.40 g, 1.71 mmol, 65%). ^1^H NMR (400 MHz, CDCl_3_) δ (ppm) = 8.37 (d, *J* = 1.5 Hz, 4H), 7.82 (d, *J* = 1.3 Hz, 4H), 7.26–7.21 (m, 20H), 7.05 (dd, *J* = 8.4, 6.9 Hz, 16H), 6.99–6.94 (m, 8H), 6.73 (d, *J* = 8.4 Hz, 8H), 5.02 (s, 16H); ^13^C NMR (101 MHz, CDCl_3_) δ (ppm) = 157.0, 137.3, 134.8, 132.1, 130.8, 130.0, 128.5, 128.3, 127.3, 127.3, 126.7, 123.7, 121.7, 106.9, 70.5; HRMS (ESI): *m*/*z* 1,405.5581 [M–H^+^]. Calcd. for C_100_H_77_O_8_^+^: 1,405.5618.

**S13. S12** (2.27 g, 1.61 mmol, 1.00 eq.), Pd/C (5 wt% Pd, 2.17 g), THF (100 ml) and ethanol (20 ml) were sealed in an Ace Glass 500 ml pressure flask (product no. 8415-25). The resulting mixture was bubbled with H_2_ gas via a balloon for *~*30 min. The balloon was removed and the flask was stirred overnight in a preheated 80 °C oil bath. The warm reaction mixture was filtered through celite eluting with additional THF. After evaporation of the solvent, analysis by ^1^H NMR indicated *~*75% debenzylation. The residue was hydrogenated overnight again with Pd/C (5 wt% Pd, 700 mg) in THF (50 ml) and ethanol (10 ml), and worked up similarly. Analysis by ^1^H NMR indicated complete debenzylation. The residue was purified by flash chromatography on silica gel (eluent:gradient, 0:1−1:1 EtOAc/DCM v/v) to obtain **S13** as a yellow powder after trituration with hexane and drying under suction (1.12 g, 1.60 mmol, 100%). ^1^H NMR (400 MHz, DMSO) δ (ppm) = 9.20 (s, 8H), 8.06 (d, *J* = 1.5 Hz, 4H), 7.65 (d, *J* = 1.3 Hz, 4H), 6.98 (t, *J* = 8.1 Hz, 4H), 6.46 (d, *J* = 8.2 Hz, 8H); ^13^C NMR (101 MHz, DMSO) δ (ppm) = 156.2, 134.8, 133.7, 130.0, 129.8, 128.7, 126.1, 123.7, 116.4, 107.3; HRMS (ESI): *m*/*z* 683.1729 [M–1]^−^. Calcd. for C_44_H_27_O_8_^−^: 683.1706.

**En-Per**. **S13** (1.10 g, 1.61 mmol, 1.00 eq.) and 1,8-dibromooctane (1.19 ml, 6.44 mmol, 4.00 eq.) were dissolved in dry DMF (900 ml) under argon at 40 °C. After 30 min, K_2_CO_3_ (4.17 g, 32.2 mmol, 20.0 eq.) was added and the resulting mixture was stirred at 80 °C for a further 48 h. The mixture was cooled to room temperature, filtered and the solvent was removed under reduced pressure. The residue was purified by flash chromatography on silica gel (eluent:gradient, 0:1–1:0 DCM/*n*-hexane v/v). All of the material that eluted was combined, affording a mixture presumably constituting the material that had been predominantly alkylated as a yellow solid (430 mg, 24% mass yield). Analysis by NMR suggested that **S14** constituted the majority of this material (≥60 mol% pure; the NMR spectrum is provided in the Supplementary Information). Then, 120 mg of the material was purified by preparative high-performance liquid chromatography (HPLC) (eluent: 1:1 DCM/*n*-hexane v/v) to afford **En-Per** as a yellow powder after trituration with methanol and drying under suction (74 mg, 0.07 mmol, 4%). Based on this, if all the DCM-mobile material was purified by preparative HPLC, a yield of *~*14% is anticipated. ^1^H NMR (400 MHz, CDCl_3_) δ (ppm) = 8.11 (d, *J* = 1.4 Hz, 4H), 7.52 (d, *J* = 1.3 Hz, 4H), 7.25 (t, *J* = 8.3 Hz, 4H), 6.72–6.64 (m, 8H), 3.98–3.85 (m, 16H), 1.56–1.41 (m, 16H), 1.20 (q, *J* = 7.3 Hz, 8H), 1.13–0.88 (m, 24H); ^13^C NMR (101 MHz, CDCl_3_) δ (ppm) = 157.7, 157.7, 134.7, 132.6, 130.8, 129.4, 128.4, 127.1, 123.1, 122.3, 106.9, 106.4, 70.1, 69.4, 30.1, 29.7, 29.1, 29.0, 27.1, 26.5; HRMS (ESI): *m*/*z* 1,125.6226 [M–H^+^]. Calcd. for C_76_H_85_O_8_^+^: 1,125.6244.

### X-ray crystallography

X-ray data were collected on a Bruker D8 QUEST diffractometer, equipped with an Incoatec IμS Cu microsource (*λ* = 1.5418 Å) and a PHOTON III detector operating in the shutterless mode. Crystals were mounted on a MiTeGen crystal mount using inert polyfluoroether oil and the analysis was carried out under an Oxford Cryosystems open-flow N_2_ Cryostream operating at 180(2) K. The control and processing software was Bruker APEX4 (v. 2022.1-1)^60^. The diffraction images were integrated using SAINT in APEX4, and a multiscan correction was applied using SADABS. The final unit-cell parameters were refined against all reflections. Structures were solved using SHELXT^61^ and refined using SHELX^62^. All of the crystal structures include CHCl_3_ solvent molecules, and the crystals were generally prone to solvent loss and degradation on removal from the mother liquour. In some cases, the SQUEEZE algorithm within PLATON was applied to complete the refinement^63^. Supplementary Table 1 provides summary details of the data collection and structure/refinement parameters.

### Computational details

Computations were performed using (time-dependent) density functional theory as implemented in ORCA 5.0 (ref. ^64^). Ground- and excited-state structure optimizations, Hessians and vibronic coupling parameters were computed at the ωB97X-D3/def2-SVP level of theory^65,66^. Vertical excitations are reported at the LC-BLYP/def2-TZVP level using a range separation parameter of *μ* = 0.1 a.u. chosen to experimentally reproduce the observed singlet and triplet excitation energies^67^. Absorption and emission spectra were computed using a path integral approach^68^ as implemented in the ORCA ESD module, employing ωB97X-D3/def2-SVP Hessians along with LC-BLYP/def2-TZVP excitation energies. A linear vibronic coupling model^69–71^ was used for interpreting the spectral lineshapes. Here the Huang–Rhys factor for normal mode *i* was computed as

$${S}_{i}=\frac{{\kappa }_{i}^{2}}{{\omega }_{i}^{2}},$$

where *κ*_*i*_ is the vibronic coupling parameter and *ω*_*i*_ is the wavenumber for mode *i*, both inserted in atomic units and computed with respect to the ground-state modes. These computations were carried out using the linear vibronic coupling functionality of SHARC^70^. Natural transition orbitals^72^ were computed using the TheoDORE program^73^. Molecular volumes were calculated via a marching tetrahedron model^74^.

### Electrochemical measurements

Cyclic voltammetry was carried out using a PalmSens EmStat4S at a scan rate of 100 mV s^−1^. Solutions were prepared in dry, degassed THF with 0.1 M *n*-Bu_4_NPF_6_ as the supporting electrolyte. All the experiments were run under argon with a glassy carbon working electrode, Ag/AgCl as the wire quasi-reference and a Pt wire as the counter electrode. The potentials were internally referenced to the half-potential of an fcH/fcH^+^ redox couple.

### Photophysical measurements

Solution absorption spectra were measured using a Shimadzu UV-1800 instrument. Molar extinction coefficients were determined from triplicate runs using a method intended to minimize weighing and dilution error. For each run, >3 mg of the compound was accurately weighed into a 25 ml volumetric flask to make a stock solution. Then, 100 µl of the stock solution was titrated five times into a 1-cm-path-length cuvette (2 ml starting blank solvent volume), measuring the absorption spectra after each addition. Fluorescence and time-correlated single-photon-counting experiments were carried out on Edinburgh Instruments FS5. Solution PL quantum yield measurements were carried out in an integrating sphere on Edinburgh Instruments FS5.

For organic films for photophysics, glass substrates (steady-state PL and absorption, PL quantum efficiency and transient PL) and quartz substrates (TA) were prepared, and they were cleaned with acetone and isopropyl alcohol with sonification for 10 min before loading them into an evaporator (Angstrom Engineering). Then, 50-nm- and 100-nm-thick films were formed on glass and quartz substrates, respectively. The thermal evaporating process was conducted in a vacuum chamber under <5 × 10^−7^ mbar. Steady-state PL spectra were measured by an Edinburgh Instruments fluorescence spectrometer (FLS980) with a monochromated xenon arc lamp at *λ*_Ex_ = 330 nm under nitrogen flow. FLS980 with an integrating sphere under nitrogen flow was used to measure the PL quantum efficiency, and the films were excited by a 330 nm laser. Transient PL was recorded by using an Andor electrically gated intensified charge-coupled device (iCCD) with 330 nm laser excitation; the decay kinetics were obtained from the integration of the total spectrum at each time.

To determine the emitter triplet energies, the samples were prepared via drop casting onto a sapphire substrate from a mixture of the compound at 1 wt% to ZEONEX in toluene. The films were then dried in a vacuum oven at room temperature for 1 h to remove any trace solvent. The time-resolved PL spectra were recorded using nanosecond gated luminescence and lifetime measurement setup (from 400 ps to 1 s). The sample was loaded into a Janis Research VNF-100 cryostat, which was placed under a vacuum and kept at room temperature. The excitation pulses were provided by an Ekspla Nd:YAG laser at the third harmonic of 355 nm and the emission was collected after passing through a spectrograph on a Stanford Computer Optics iCCD camera to produce the time-resolved emission spectra.

For photostability measurements, the samples were prepared in degassed toluene and irradiated with a 400 nm upconverted pump laser. The PL intensity was monitored as a function of time with an iCCD camera.

TA spectroscopy was performed on a setup powered using a commercially available Ti:sapphire amplifier (Spectra Physics Solstice Ace). The amplifier operates at 1 kHz and generates 100 fs pulses centred at 800 nm with an output of 7 W. For the ultrafast TA measurements, a portion of the laser fundamental was frequency doubled using a 1-mm-thick β-barium borate crystal for sample excitation at 400 nm, whereas the third harmonic (355 nm) of an electronically triggered, Q-switched Nd:YVO_4_ laser (Innolas Picolo 25) provided the ~1 ns pump pulses for the nanosecond–microsecond (1 ns to 100 μs) TA measurements. The probe was provided by a broadband visible (525–775 nm) non-collinear optical parametric amplifier. The probe pulses are collected with a Si dual-line array detector (Hamamatsu S8381-1024Q), driven and read by a custom-built board from Entwicklungsbüro Stresing. For determining the triplet-excited-state absorption of the terminal emitters, the probe was instead generated by a LEUKOS Disco 1 ultraviolet low-timing-jitter supercontinuum laser (STM-1-UV), which was then electronically delayed relative to the femtosecond 400 nm excitation by an electronic delay generator (Stanford Research Systems DG645).

### Organic light-emitting devices

For the fabrication of OLED devices, indium-tin-oxide-coated substrates (~15 Ω cm^–2^) were cleaned with acetone and isopropyl alcohol, and then O_2_ plasma treatment was applied to align the energy level with a hole-transporting layer. All the layers, including the organic layers and a LiF/aluminium cathode, were thermally deposited in a high vacuum (~10^−7^ torr). The performance of the OLED devices was measured by a Keithley 2635 source meter and a calibrated Si photodiode. The EL spectra were recorded by an Ocean Optics Flame spectrometer.

## Online content

Any methods, additional references, Nature Portfolio reporting summaries, source data, extended data, supplementary information, acknowledgements, peer review information; details of author contributions and competing interests; and statements of data and code availability are available at 10.1038/s41563-024-01812-4.

### Supplementary information

Supplementary Information Supplementary Figs. 1–138, Notes 1–7, Tables 1–22 and Discussion.

## Figures and Tables

**Extended Data Fig. 1:**
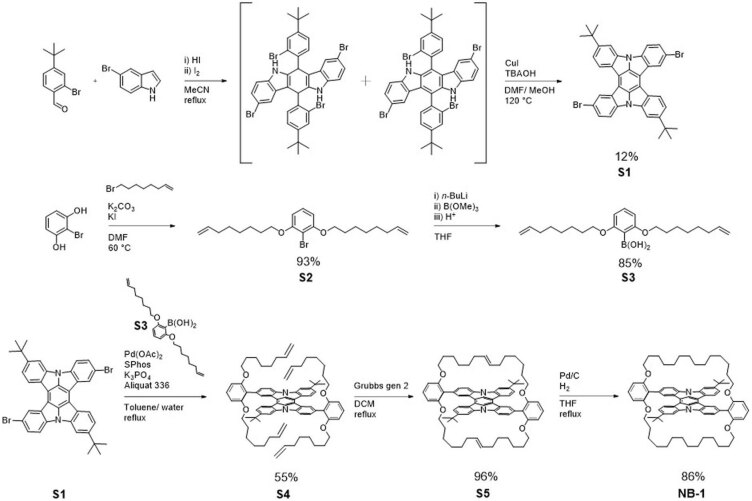
Synthesis of NB-1. Scheme for the synthesis of NB-1 via sequential Suzuki cross-coupling, Grubbs metathesis and catalytic hydrogenation chemistry.

**Extended Data Fig. 2:**
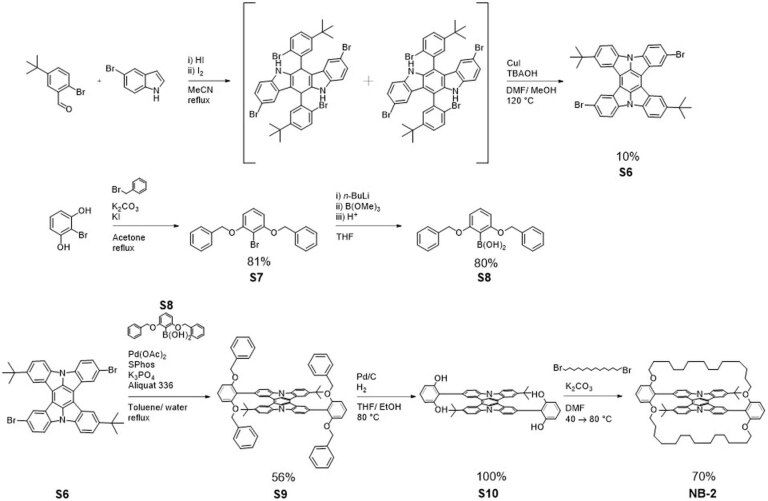
Synthesis of NB-2. Scheme for the synthesis of NB-2 via sequential Suzuki cross-coupling, de-benzylation and nucleophilic substitution chemistry.

**Extended Data Fig. 3:**
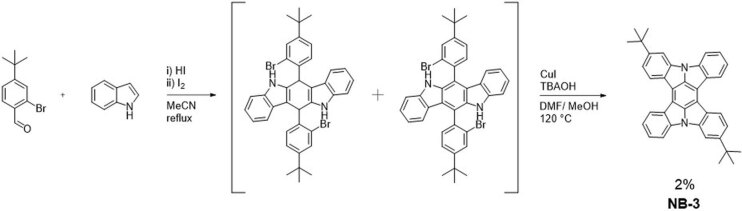
Synthesis of NB-3. Scheme for the synthesis of NB-2 via acid-catalysed condensation and Cu-catalysed intramolecular Ullmann chemistry.

**Extended Data Fig. 4:**
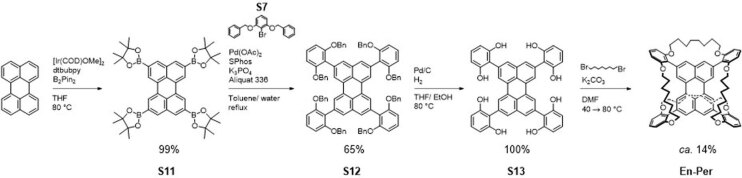
Synthesis of En-Per. Scheme for the synthesis of En-Per via chemistry analogous to that employed in the synthesis of NB-2.complete.

## Data Availability

The main data supporting the findings of this study are available within this Article and its Supplementary Information. Further data are available from the corresponding author on reasonable request. The X-ray crystallographic coordinates for structures reported in this study have been deposited at the Cambridge Crystallographic Data Centre (CCDC) under deposition numbers 2236336, 2236337, 2236338, 2236339 and 2282946.
