# Supplementary material for: Suppression of Dexter transfer by covalent encapsulation for efficient matrix-free narrowband deep blue hyperfluorescent OLEDs
Source: Nat Mater. 2024 Mar 13;23(4):519–26. doi: 10.1038/s41563-024-01812-4 (PMC10990937; doi:10.1038/s41563-024-01812-4)
Supplement: Supplementary file 1 — Supplementary Figs. 1–138, Notes 1–7, Tables 1–22 and Discussion. [file 41563_2024_1812_MOESM1_ESM.pdf]

nature materials

Article

https://doi.org/10.1038/s41563-024-01812-4

Suppression of Dexter transfer by covalent encapsulation for efficient matrix-free narrowband deep blue hyperfluorescent OLEDs

In the format provided by the
authors and unedited

Table of Contents

1. **Supplementary Note 1** - Synthesis S2
2. **Supplementary Note 2** - X-Ray crystallography S31
3. **Supplementary Note 3** - Theoretical calculations S39
4. **Supplementary Note 4** - Thermal analysis S46
5. **Supplementary Note 5** - Absorption, photoluminescence and electrochemistry S47
6. **Supplementary Note 6** - Transient absorption spectroscopy S76
7. **Supplementary Note 7** - Electroluminescence S87
8. **Supplementary References** S114

Supplementary Note 1 - Synthesis

General

^{1}H NMR spectra were recorded on a 400 MHz Avance III HD Spectrometer, 400 MHz Smart Probe Spectrometer or a 500 MHz DCH Cryoprobe Spectrometer in the stated solvent using residual protic solvent as the internal standard. ^{1}H NMR chemical shifts are reported to the nearest 0.01 ppm. The coupling constants (J) are measured in Hertz. ^{13}C NMR spectra were recorded on the 500 MHz DCH Cryoprobe Spectrometer in the stated solvent using the residual protic solvent as the internal standard. ^{13}C NMR chemical shifts are reported to the nearest 0.1 ppm. Mass spectra were obtained using a Waters LCT, Finnigan MAT 900XP or Waters MALDI micro MX spectrometer at the Department of Chemistry, University of Cambridge. Elemental analyses were obtained on an Exeter Analytical Inc. CE-440 elemental analyser. Thermal gravimetric analysis was run under a nitrogen atmosphere at a rate of 10 °C min^{-1} using a Mettler Toledo TGA/DSC 2 instrument at a gas flow of 125 $cm^{3}min^{-1}$. Differential scanning calorimetry was run on a Mettler Toledo DSC822e under the following cycling conditions: 25–380 °C at 10 K min^{-1}; 10 min hold at 380 °C; 380–25 °C at 50 K min^{-1}; 10 min hold at 25 °C; 25–380 °C at 10 K min^{-1}; 10 min hold at 380 °C; 380–25 °C at 50 K min^{-1}; 10 min hold at 25 °C. Reactions requiring an inert atmosphere were carried out under argon. Thin layer chromatography (TLC) was carried out on silica gel and visualized using UV light (254, 365 nm). Flash chromatography was carried out on a Biotage® Isolera automated flash chromatography machine on 60 micron silica gel cartridges purchased from Biotage®.

All commercial chemicals were of ≥95% purity and were used as received without further purification. Anhydrous solvents were purchased from Sigma Aldrich or Acros Organics and used as received.

2-Bromo-4-*tert*-butylbenzaldehyde was prepared starting from 4-*tert*-butyltoluene according to literature procedures. 4-*tert*-butyltoluene was sequentially brominated to 2-bromo-4-*tert*-butyl toluene[1] and then 2-bromo-4-*tert*-butylbromomethylbenzene.[2] Subsequent Hass-Bender oxidation afforded 2-Bromo-4-*tert*-butylbenzaldehyde.[2]

2-Bromo-5-*tert*-butylbenzaldehyde was prepared starting from 4-*tert*-butylbromobenzene according to literature procedures. 4-*tert*-butylbromobenzene was nitrated to afford 4-*tert*-butyl-2-nitrobromobenzene. Reduction and diazotisation followed by treatment with KI afforded 4-*tert*-butyl-2-iodobromobenzene,[3] which was converted to 2-bromo-5-*tert*-butylbenzaldehyde after chemoselective metal-halogen exchange and treatment with DMF.[4]

En-DPA was prepared according to the literature procedure.[5]

Discussion

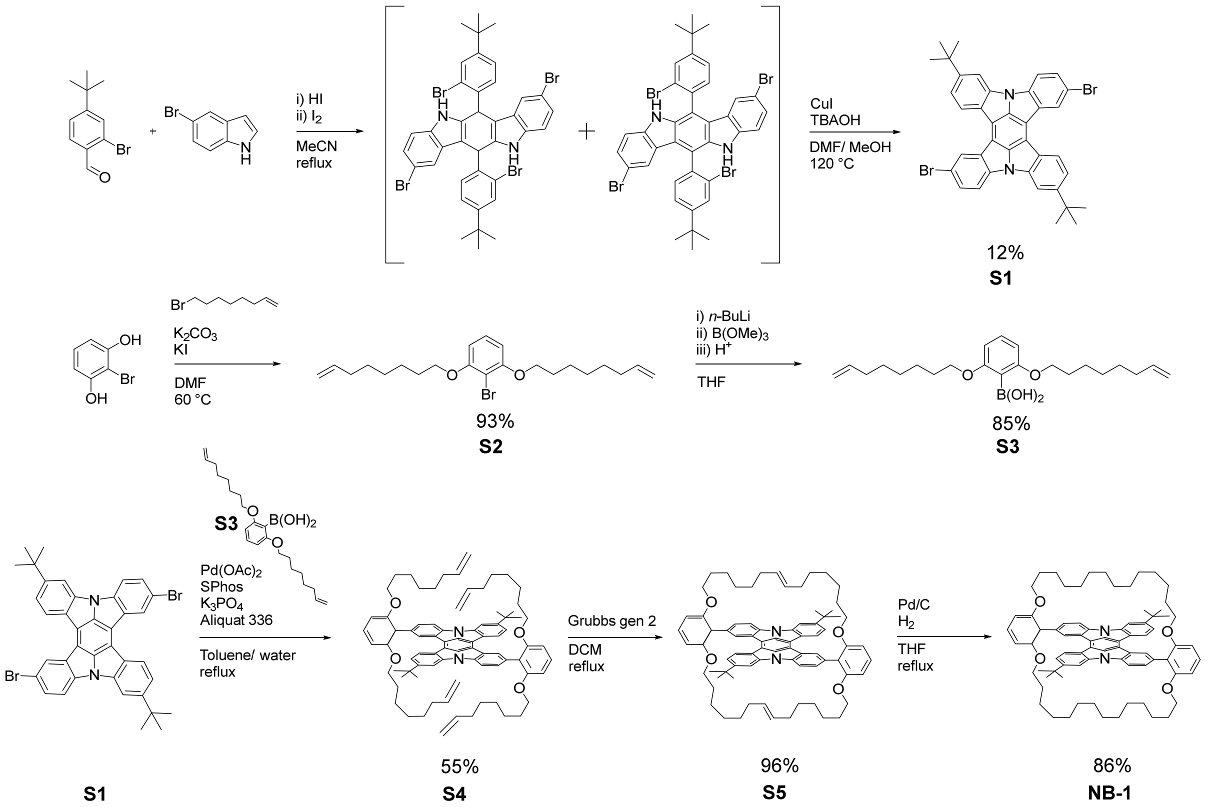

Supplementary Fig. 1: Synthetic scheme for **NB-1**.

The encapsulated emitter **NB-1** was synthesised starting from the brominated mDICz intermediate **S1**. As the ring closing intramolecular Ullmann reaction affording **S1** operates at a comparatively low temperature of 120 °C, the peripheral bromine atoms are retained. **S1** is too poorly soluble to characterise by NMR, but the structure and purity were confirmed by HRMS and elemental analysis, respectively. Fortunately, under dilute conditions in refluxing toluene **S1** was sufficiently soluble to undergo Suzuki coupling with the boronic acid **S3**, affording the soluble intermediate **S4** which could be obtained analytically pure after flash chromatography and recrystallization. Ring closing metathesis (RCM) selectively afforded **S5** in quantitative yield, which was transformed to **NB-1** via hydrogenation over Pd/C.

NB-2 could not be obtained via an analogous route. Despite screening multiple metathesis catalysts (including ones optimised for RCM of sterically encumbered substrates such as Grubbs Catalyst® M205 CAS: 927429-60-5) and different length alkene straps (octene, nonene and decene), it was not possible to observe clean RCM without appreciable polymeric impurities. This highlights the sensitivity of RCM to small changes in substrate molecular structure. The polymeric impurities could not be removed via flash chromatography or through recrystallization, and the authors did not have access to preparative GPC/SEC, prompting a different synthetic route.

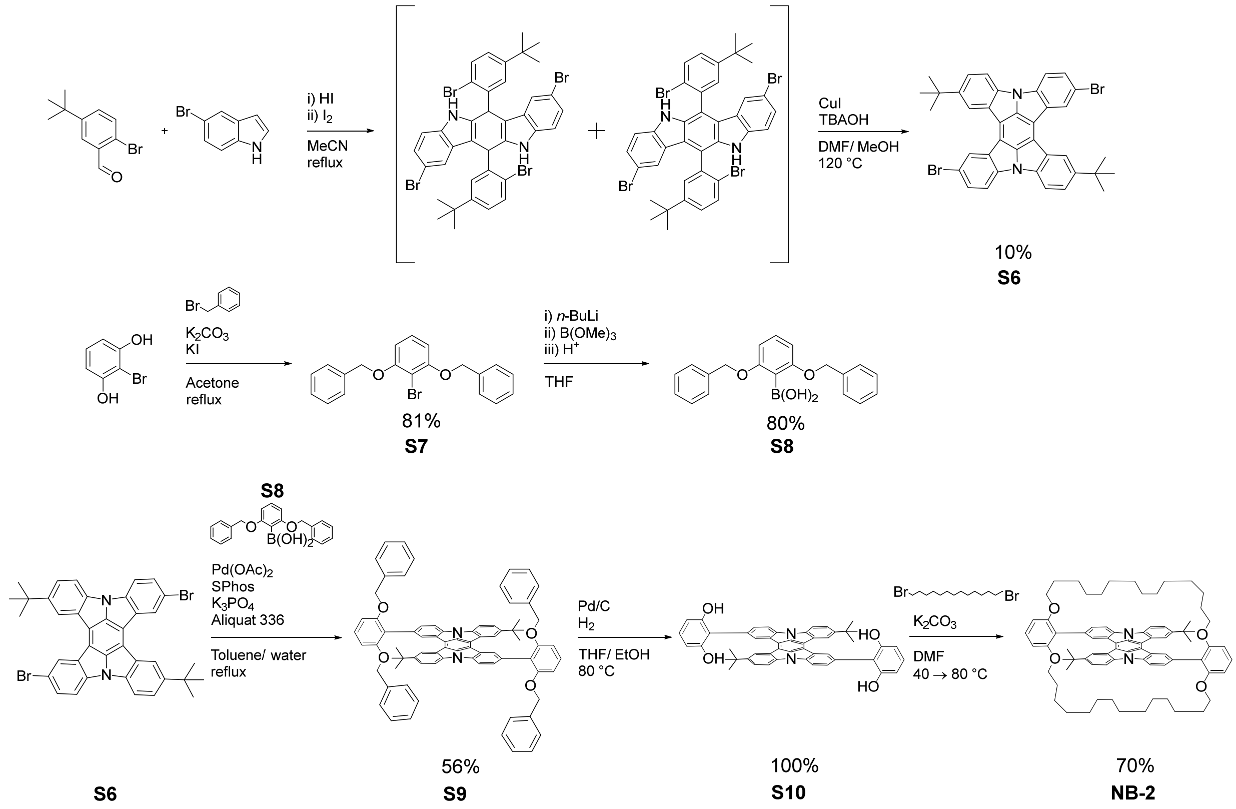

Supplementary Fig. 2: Synthetic scheme for **NB-2**

The adopted route for **NB-2** avoids the need for RCM, and starts with the Suzuki coupling of **S6** with the bis(benzyl)-protected boronic acid **S8**. **S9** was obtained in a satisfactory yield of 56%. The bulky benzyl groups afford sufficient solubility for purification by sequential flash chromatography and recrystallization. Removal of the benzyl protecting groups was carried out quantitatively through hydrogenation in a THF/ethanol mixture at 80 °C in a sealed tube – conditions under which both **S9** and the product **S10** are soluble. Fortunately, **S10** is soluble in warm DMF under dilute conditions, enabling **NB-2** to be obtained in a good yield of 70% after alkylation with 1,14-dibromotetradecane.

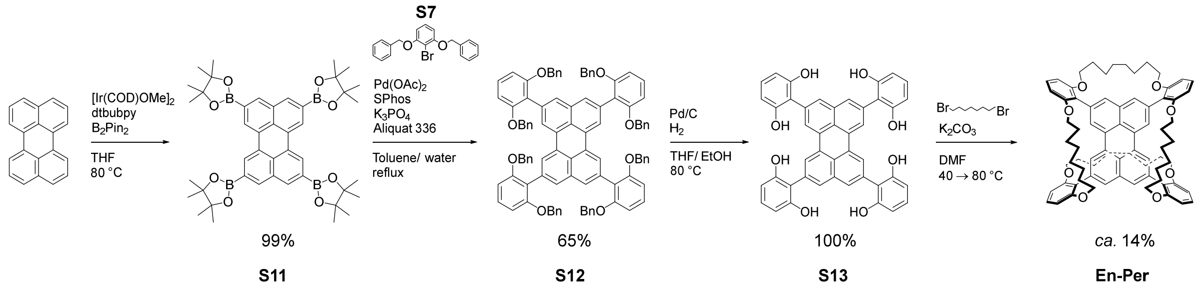

Supplementary Fig. 3: Synthetic scheme for **En-Per**

En-Per was synthesised similarly to **NB-2** through adopting benzyl protection. However, as 2,5,8,11-tetrabromoperylene is challenging to obtain pure on an appreciable scale,[6] the route was based on the tetra boronic ester **S11**. Four-fold Suzuki coupling with 10 molar equivalents of **S7** afforded **S12** in 65% isolated yield, limited by the requirement to remove incompletely coupled/ deborylated materials. A larger excess of **S7** may foreseeably improve this yield by simplifying purification,[7] but we were limited by materials availability. Deprotection via hydrogenation over Pd/C followed by alkylation with 1,8-dibromooctane afforded **En-Per** after preparative HPLC purification. It is

noteworthy that **En-Per** has a different arrangement of encapsulating straps over each face of the perylene core - a different isomer to our previously reported PDI analogue.[7]

In summary, an RCM-centred synthesis like that used for **NB-1** benefits from the good solubility of all intermediates, high late-stage yields and the commercial availability of various terminal-bromoalkenes, making **NB-1** obtainable on gram-scale. However, the RCM conditions are very sensitive to steric hinderance, which may make this strategy less versatile if polymeric side-products cannot be avoided or removed. The **NB-2** route based on alkylation with a dihalogenoalkane does not require bulky Grubbs catalysts, and so should be more tolerant of structural variation of the substrate, particularly if bulky shielding groups are desired. The versatility of the **NB-2** route was proven on the doubly-encapsulated **En-Per**. However, the synthesis of larger luminophores may be restricted by the availability of longer dihalogenoalkanes, and the poor solubility of poly-alcohol intermediates.

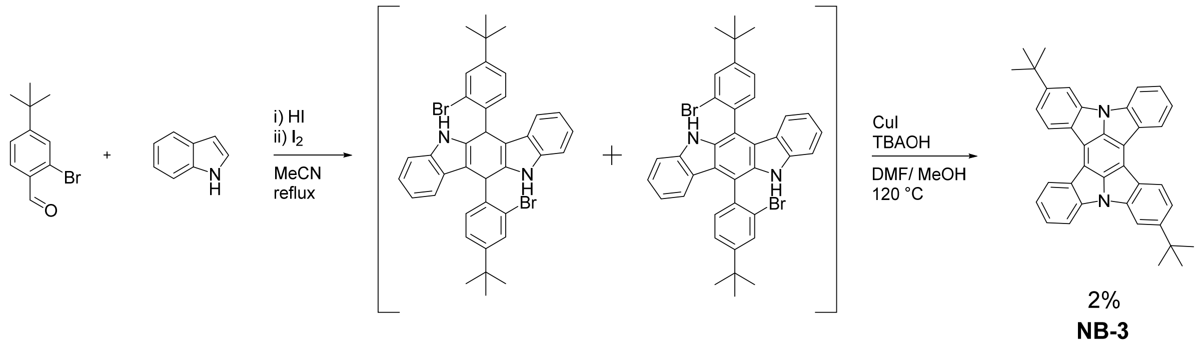

Supplementary Fig. 4: Synthetic scheme for **NB-3**

Spectra

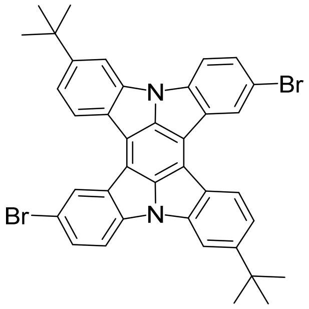

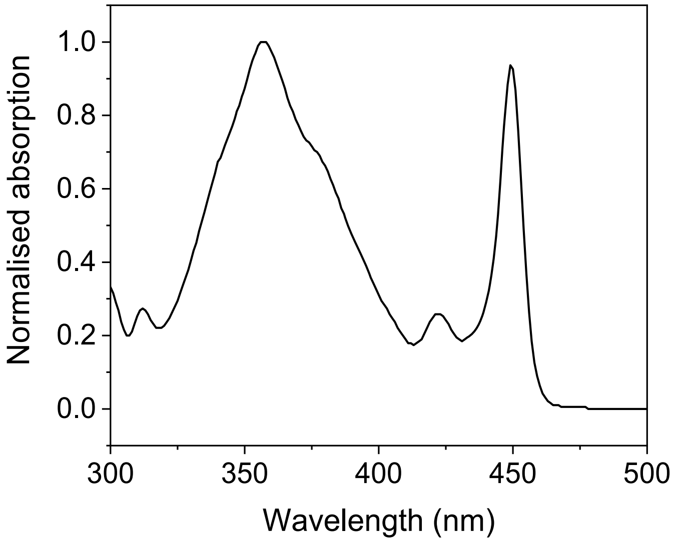

Supplementary Fig. 5: Absorption spectrum of in **S1** toluene.

Elemental Composition Report **Page 1**

Single Mass Analysis
Tolerance = 100.0 PPM / DBE: min = -1.5, max = 50.0
Element prediction: Off
Number of isotope peaks used for i-FIT = 3

Monoisotopic Mass, Even Electron Ions
7 formula(e) evaluated with 1 results within limits (up to 50 closest results for each mass)
Elements Used:
C: 17-38 H: 0-31 N: 0-2 Br: 0-2

HAB_51221 D Congrave DGC-8-17
HAB_51221 D Congrave DGC-8-17 2320 (4.968) Cm (2237:2322)

1: TOF MS ASAP+
7.13e+007

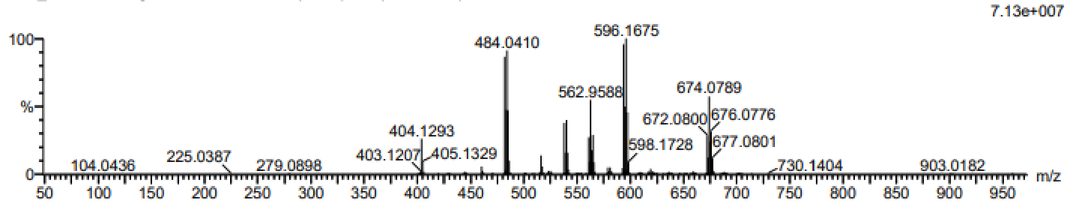

Minimum:				-1.5				
Maximum:		100.0	100.0	50.0				
Mass	Calc. Mass	mDa	PPM	DBE	i-FIT	Norm	Conf(%)	Formula
673.0832	673.0854	-2.2	-3.3	23.5	1115.4	n/a	n/a	C38 H31 N2 Br2

Supplementary Fig. 6: Mass spectrum of **S1**

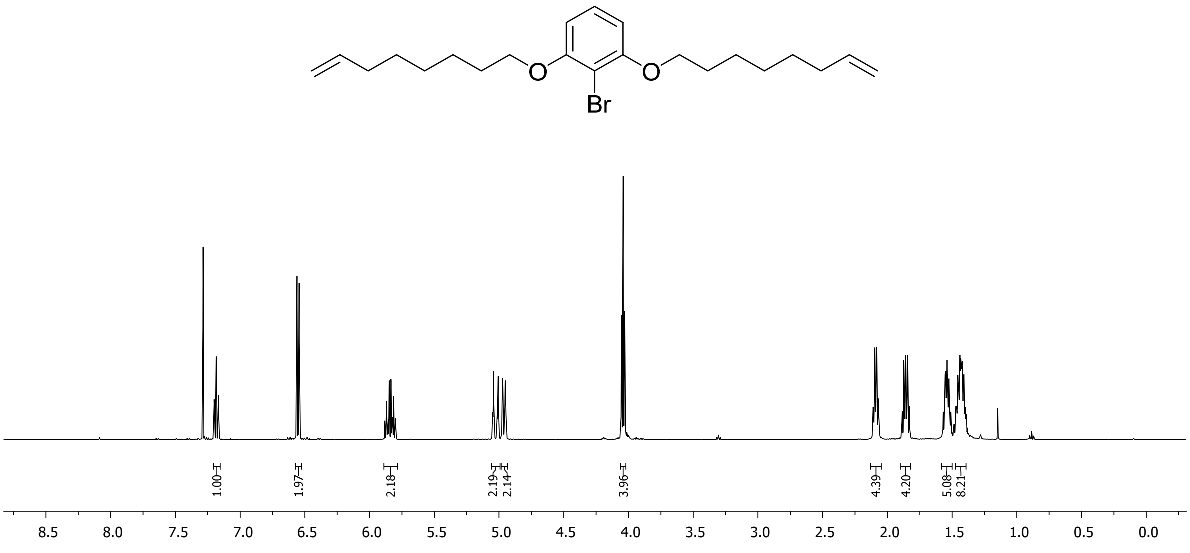

Supplementary Fig. 7: ^{1}H NMR spectrum of **S2**.

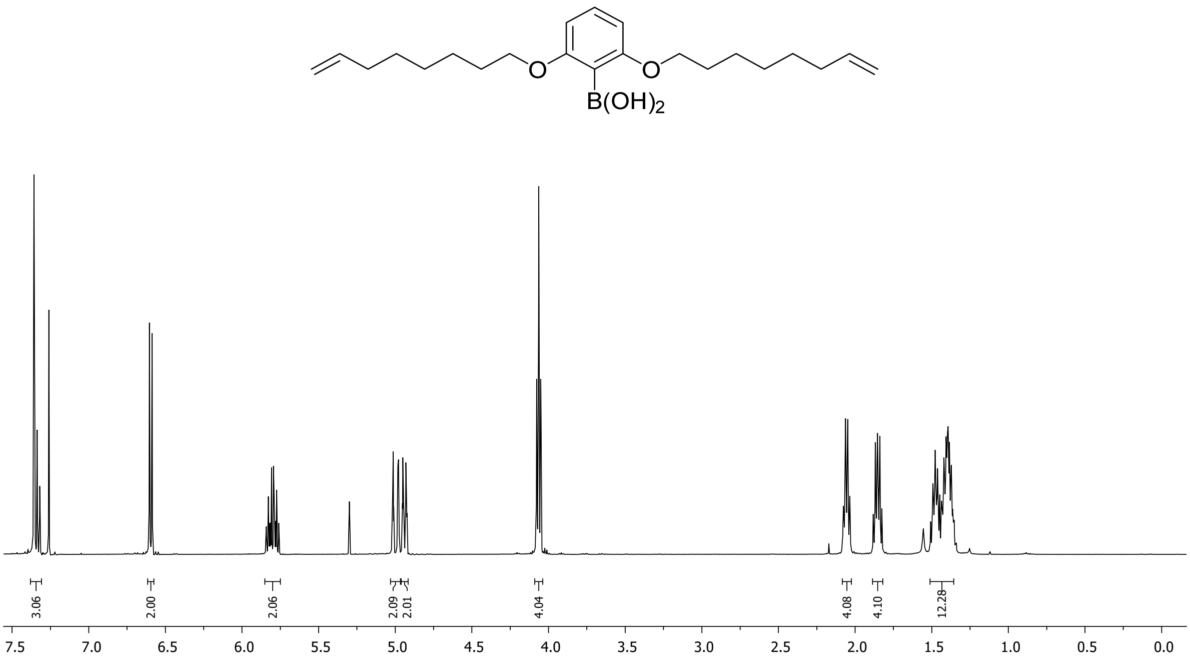

Supplementary Fig. 8: ^{1}H NMR spectrum of **S3**.

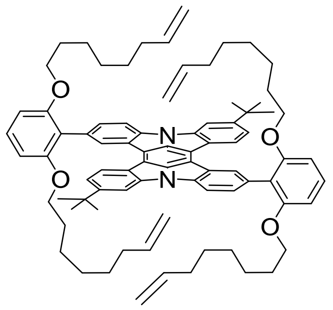

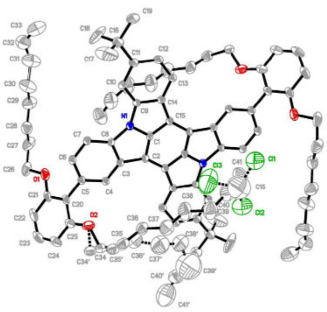

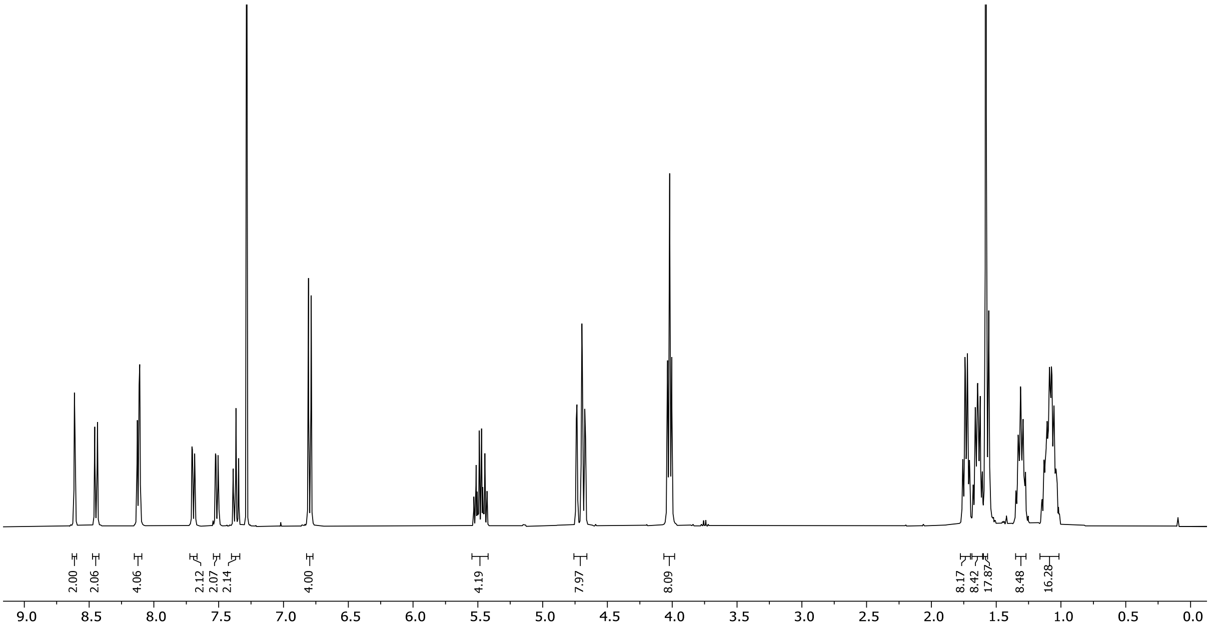

Supplementary Fig. 9: ^{1}H NMR spectrum of **S4**.

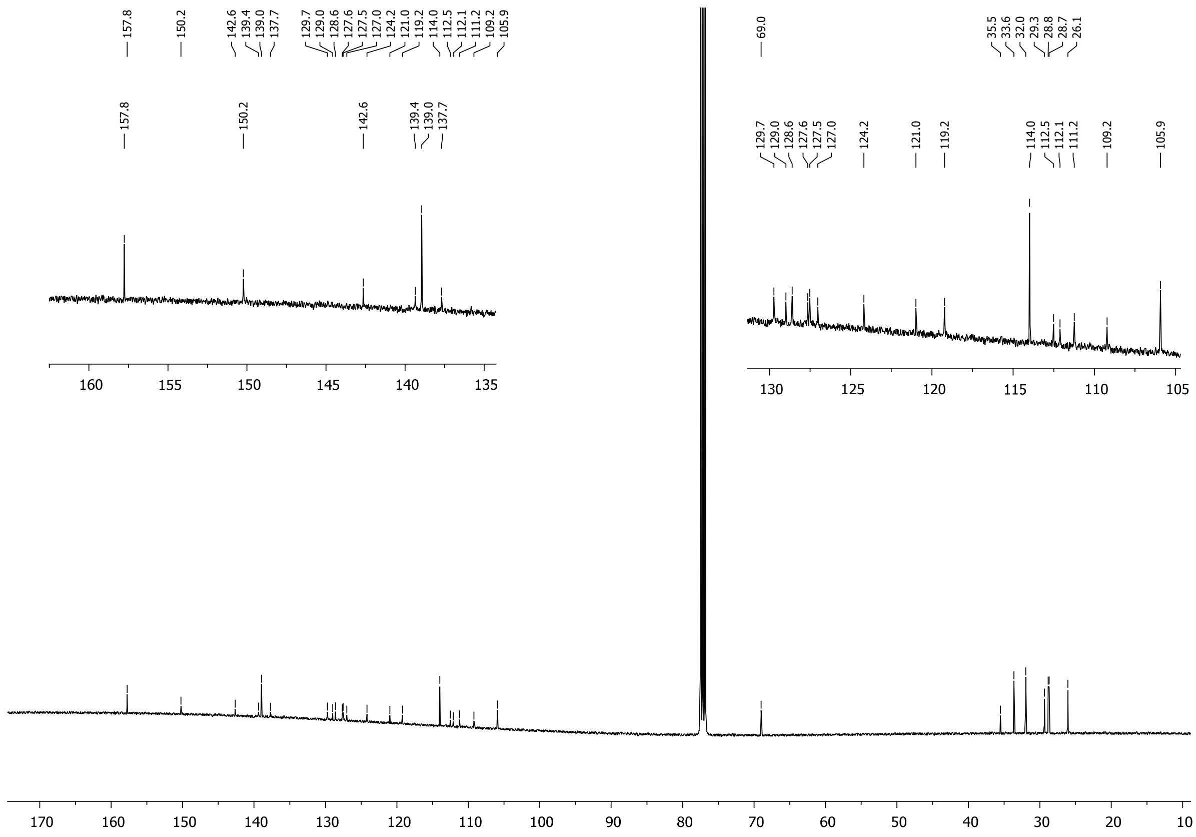

Supplementary Fig. 10: ^{13}C NMR spectrum of **S4**.

Elemental Composition Report

Page 1

Single Mass Analysis

Tolerance = 100.0 PPM / DBE: min = -1.5, max = 50.0
Element prediction: Off
Number of isotope peaks used for i-FIT = 3

Monoisotopic Mass, Even Electron Ions
2 formula(e) evaluated with 1 results within limits (up to 50 closest results for each mass)
Elements Used:
C: 66-82 H: 0-97 N: 0-2 O: 0-4
HAB_51359 DCONGRAVE DGC-8-57
HAB_51359 DCONGRAVE DGC-8-57 2122 (4.552) Cm (2087:2128)

1: TOF MS ASAP+
2.06e+005

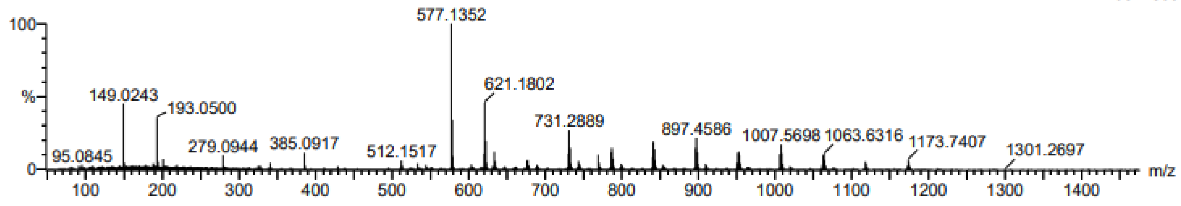

Minimum:				-1.5				
Maximum:		5.0	100.0	50.0				
Mass	Calc. Mass	mDa	PPM	DBE	i-FIT	Norm	Conf(%)	Formula
1173.7407	1173.7448	-4.1	-3.5	35.5	258.3	n/a	n/a	C82 H97 N2 O4

Supplementary Fig. 11: Mass spectrum of **S4**.

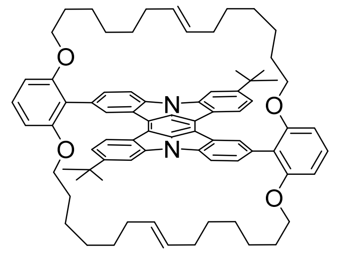

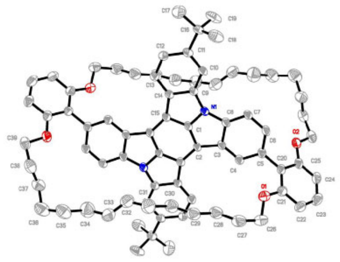

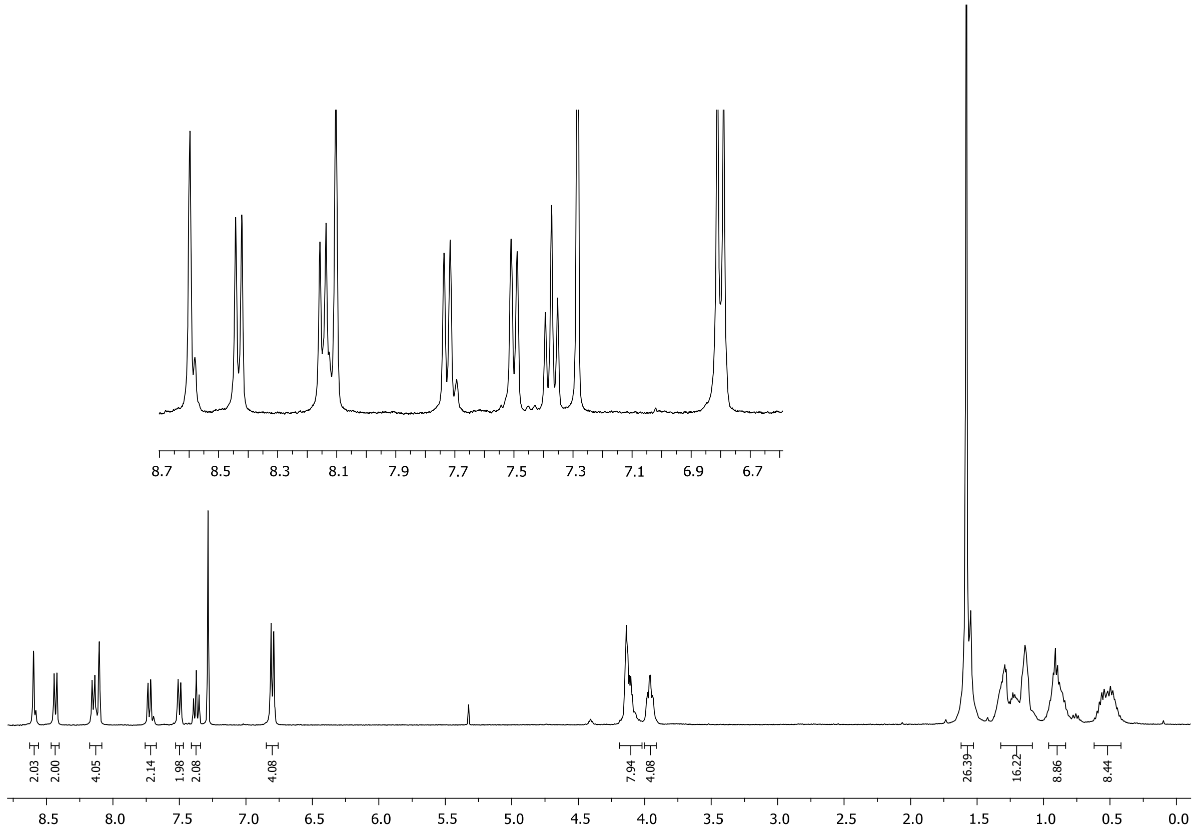

Supplementary Fig. 12: ^{1}H spectrum of **S5**.

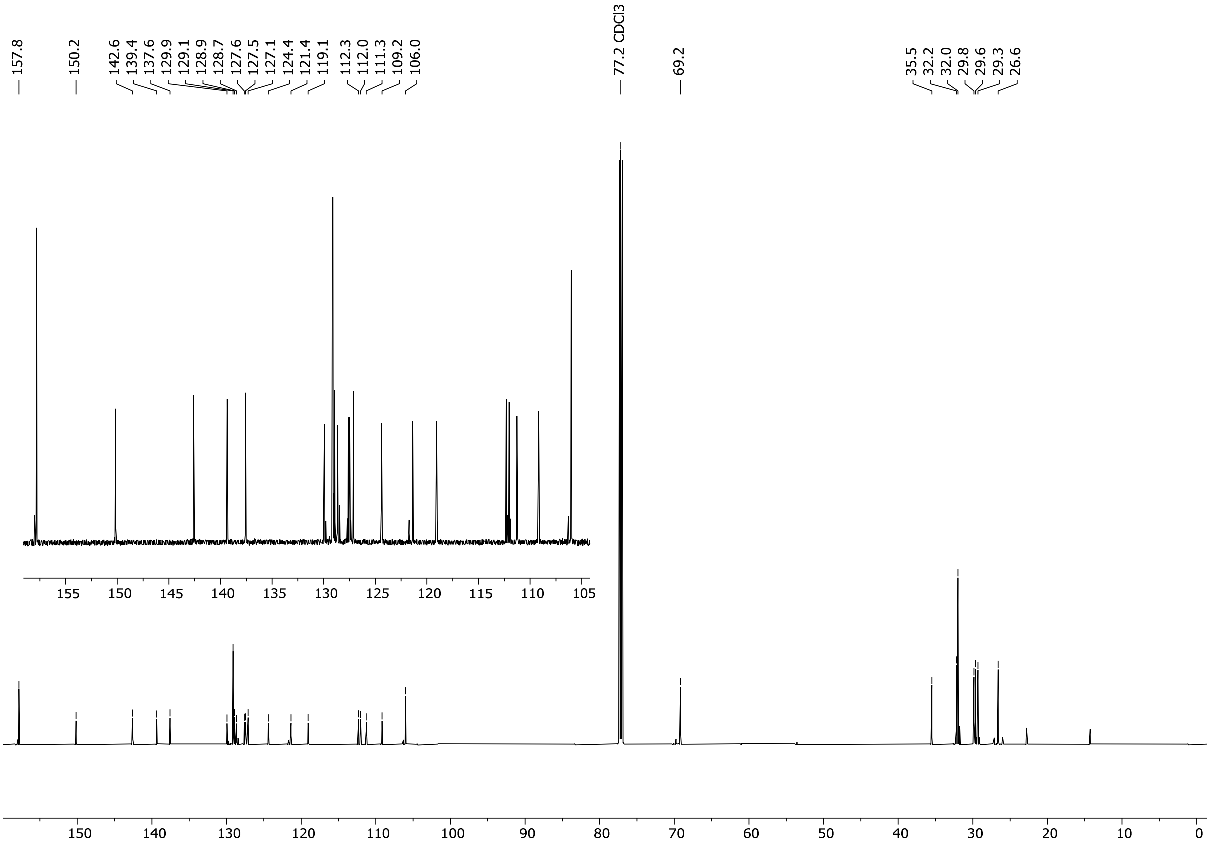

Elemental Composition Report

Page 1

Single Mass Analysis

Tolerance = 100.0 PPM / DBE: min = -1.5, max = 50.0
Element prediction: Off
Number of isotope peaks used for i-FIT = 3

Monoisotopic Mass, Even Electron Ions
2 formula(e) evaluated with 1 results within limits (up to 50 closest results for each mass)
Elements Used:
C: 0-78 H: 0-89 N: 0-2 O: 0-4
HAB_51362 D Congrave DGC-8-59
HAB_51362 D Congrave DGC-8-59 1951 (4.187) Cm (1925:2064)

1: TOF MS ASAP+
3.24e+006

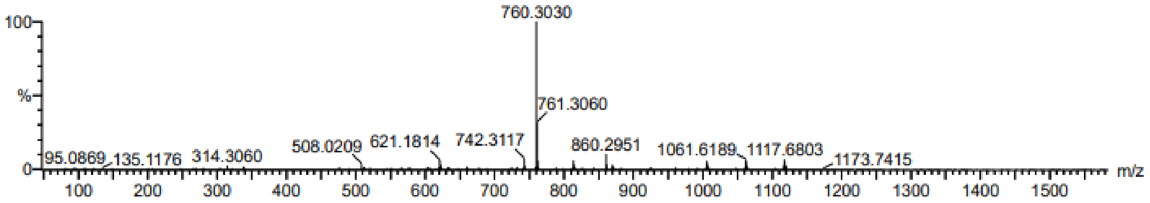

Minimum:				-1.5				
Maximum:		100.0	100.0	50.0				
Mass	Calc. Mass	mDa	PPM	DBE	i-FIT	Norm	Conf(%)	Formula
1117.6803	1117.6822	-1.9	-1.7	35.5	402.0	n/a	n/a	C78 H89 N2 O4

Supplementary Fig. 13: Mass spectrum of **S5**

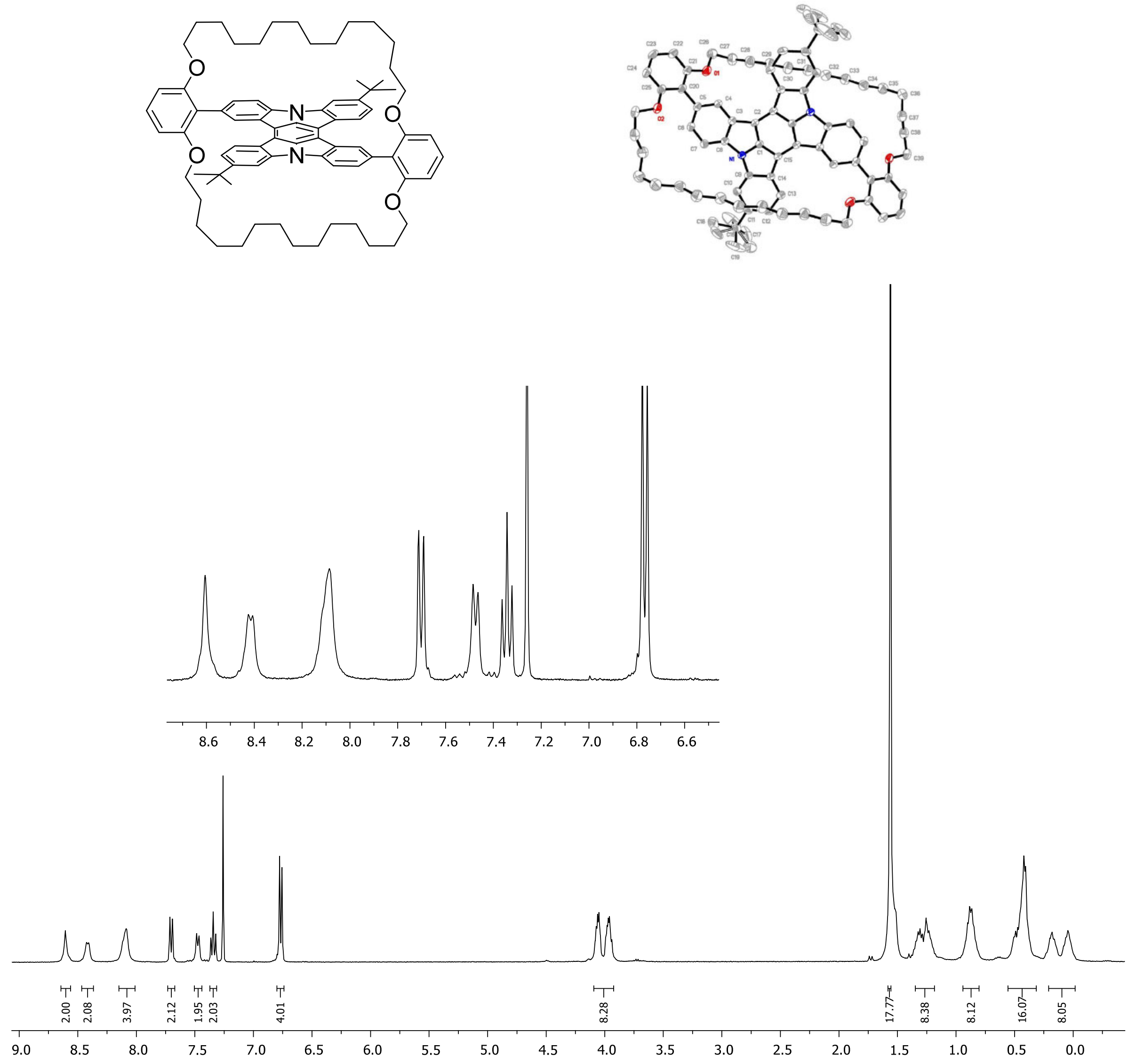

Supplementary Fig. 14: ^{1}H spectrum of **NB-1**.

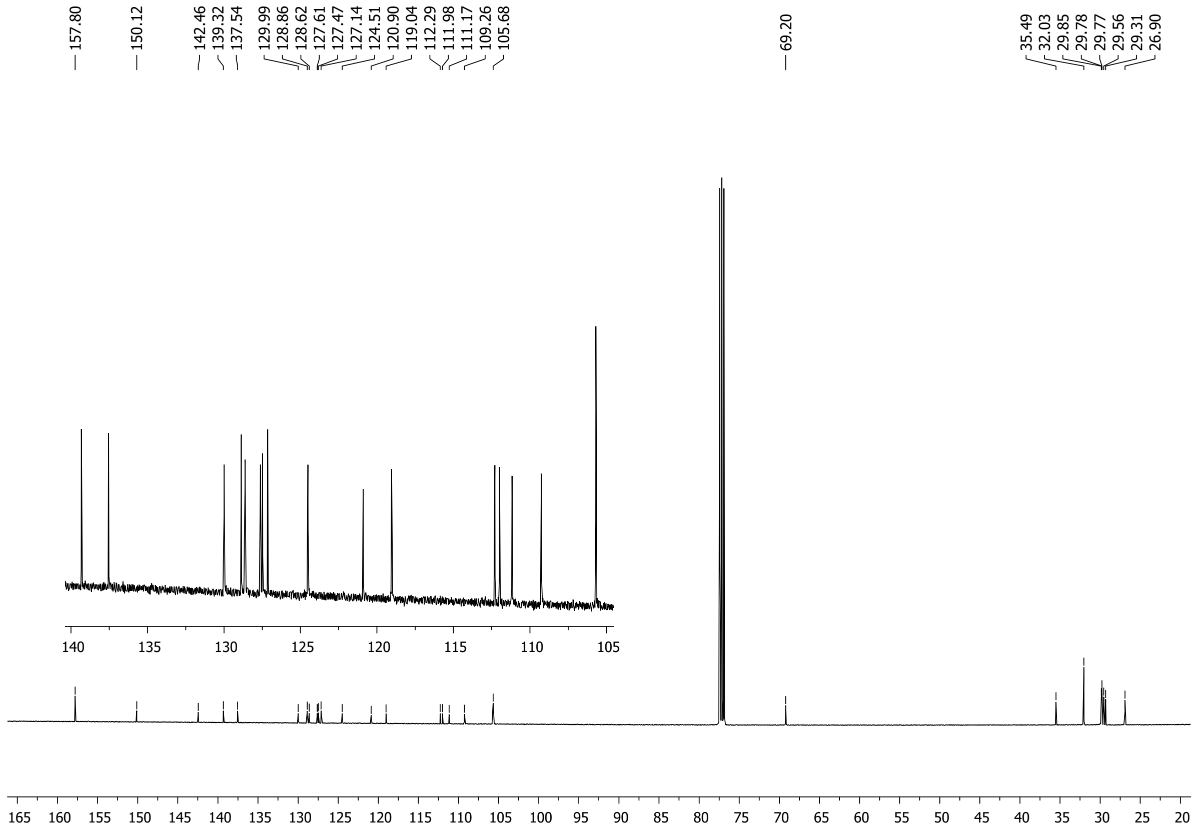

Supplementary Fig. 15: ^{13}C spectrum of **NB-1**.

Elemental Composition Report

Page 1

Single Mass Analysis

Tolerance = 100.0 PPM / DBE: min = -1.5, max = 50.0
Element prediction: Off
Number of isotope peaks used for i-FIT = 3

Monoisotopic Mass, Even Electron Ions
4 formula(e) evaluated with 1 results within limits (up to 50 closest results for each mass)
Elements Used:
C: 0-78 H: 0-93 N: 0-2 O: 0-4
HAB_51374 D Congrave DGC-8-61
HAB_51374 D Congrave DGC-8-61 2229 (4.775) Cm (2165:2231)

1: TOF MS ASAP+
2.18e+006

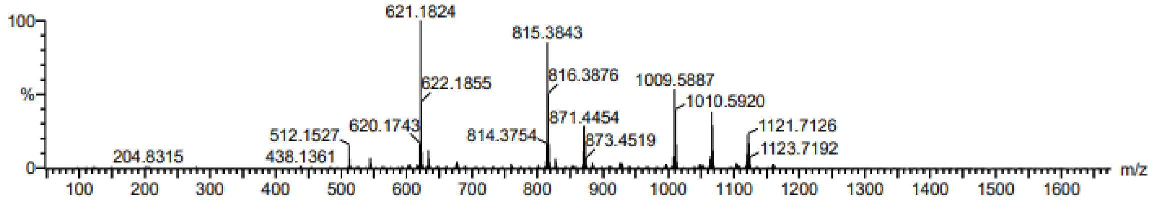

Minimum:				-1.5				
Maximum:		100.0	100.0	50.0				
Mass	Calc. Mass	mDa	PPM	DBE	i-FIT	Norm	Conf(%)	Formula
1121.7126	1121.7135	-0.9	-0.8	33.5	461.9	n/a	n/a	C78 H93 N2 O4

Supplementary Fig. 16: Mass spectrum of **NB-1**.

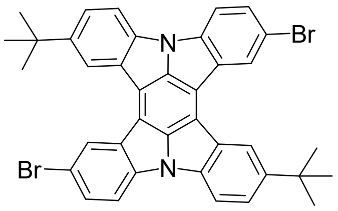

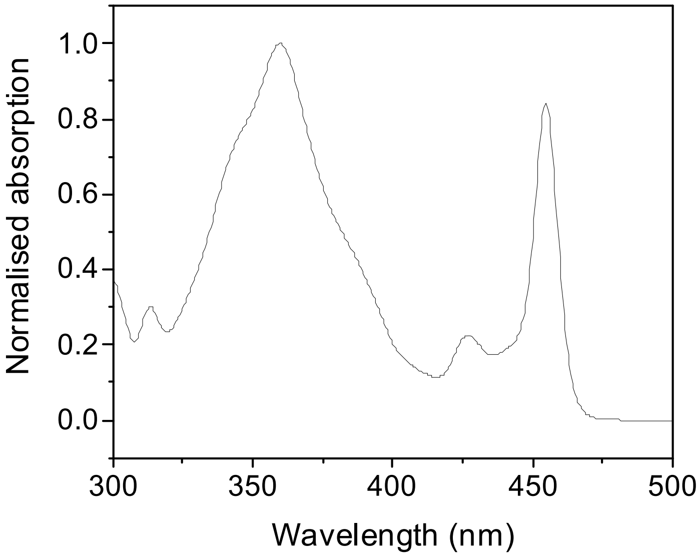

Supplementary Fig. 17: Absorption spectrum of in **S6** toluene.

Elemental Composition Report **Page 1**

Single Mass Analysis
Tolerance = 50.0 PPM / DBE: min = -1.5, max = 50.0
Element prediction: Off
Number of isotope peaks used for i-FIT = 3

Monoisotopic Mass, Even Electron Ions
7 formula(e) evaluated with 1 results within limits (up to 50 closest results for each mass)
Elements Used:
C: 0-38 H: 0-31 N: 0-2 Br: 0-2

HAB_52025 D Congrave DGC-10-82 REPEAT 2032 (4.361) Cm (2031:2033) 1: TOF MS ASAP+ 1.39e+002

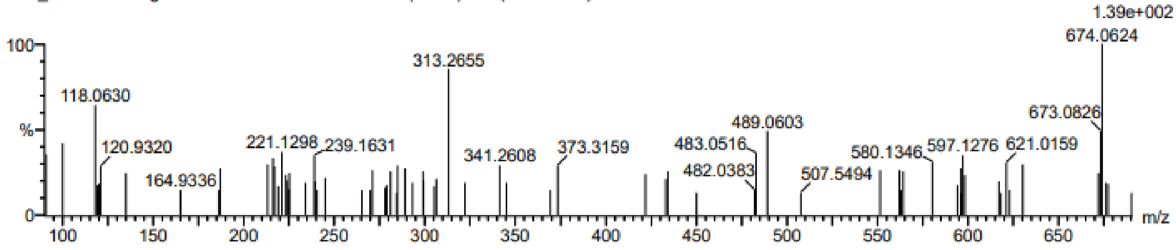

Mass	Calc. Mass	mDa	PPM	DBE	i-FIT	Norm	Conf(%)	Formula
Minimum:				-1.5				
Maximum:		100.0	50.0	50.0				
673.0826	673.0854	-2.8	-4.2	23.5	24.5	n/a	n/a	C38 H31 N2 Br2

Supplementary Fig. 18: Mass spectrum of **S6**.

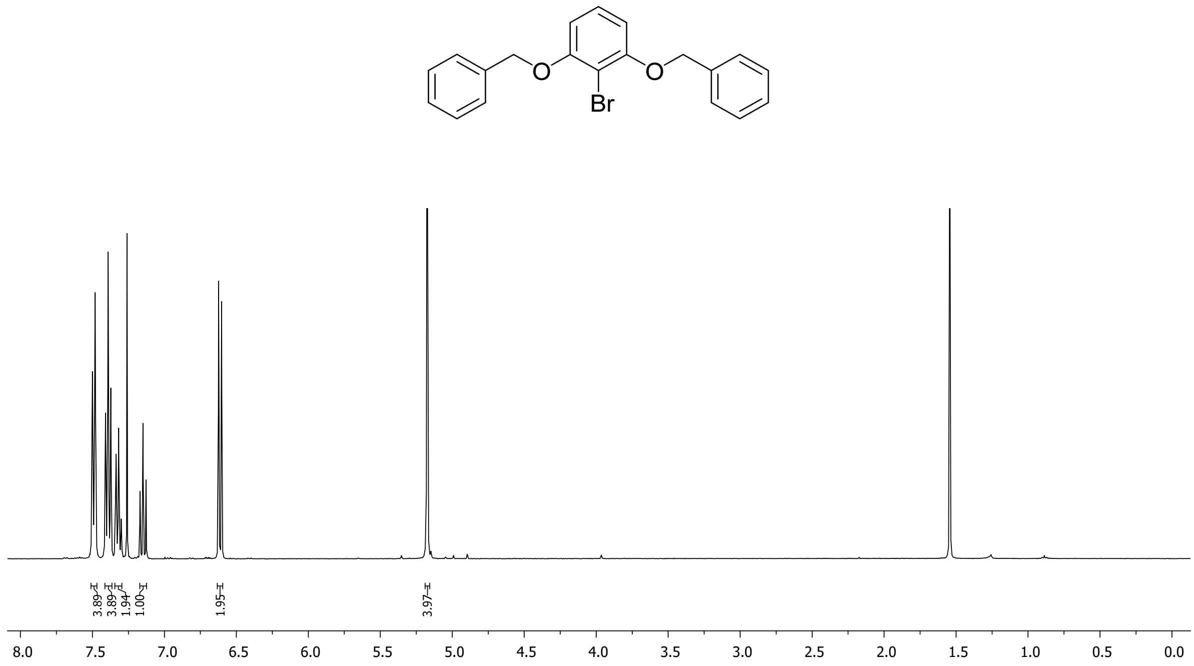

Supplementary Fig. 19: ^{1}H spectrum of **S7**.

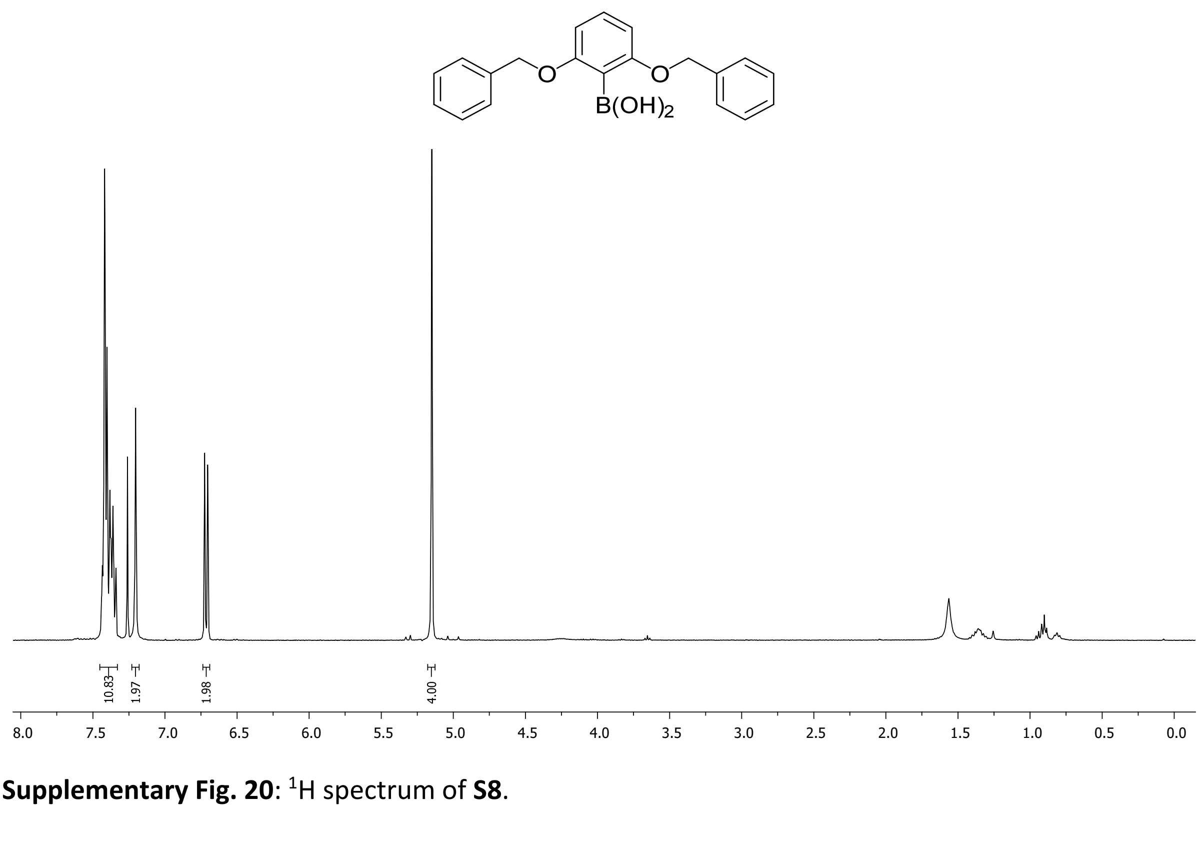

Supplementary Fig. 20: ^{1}H spectrum of **S8**.

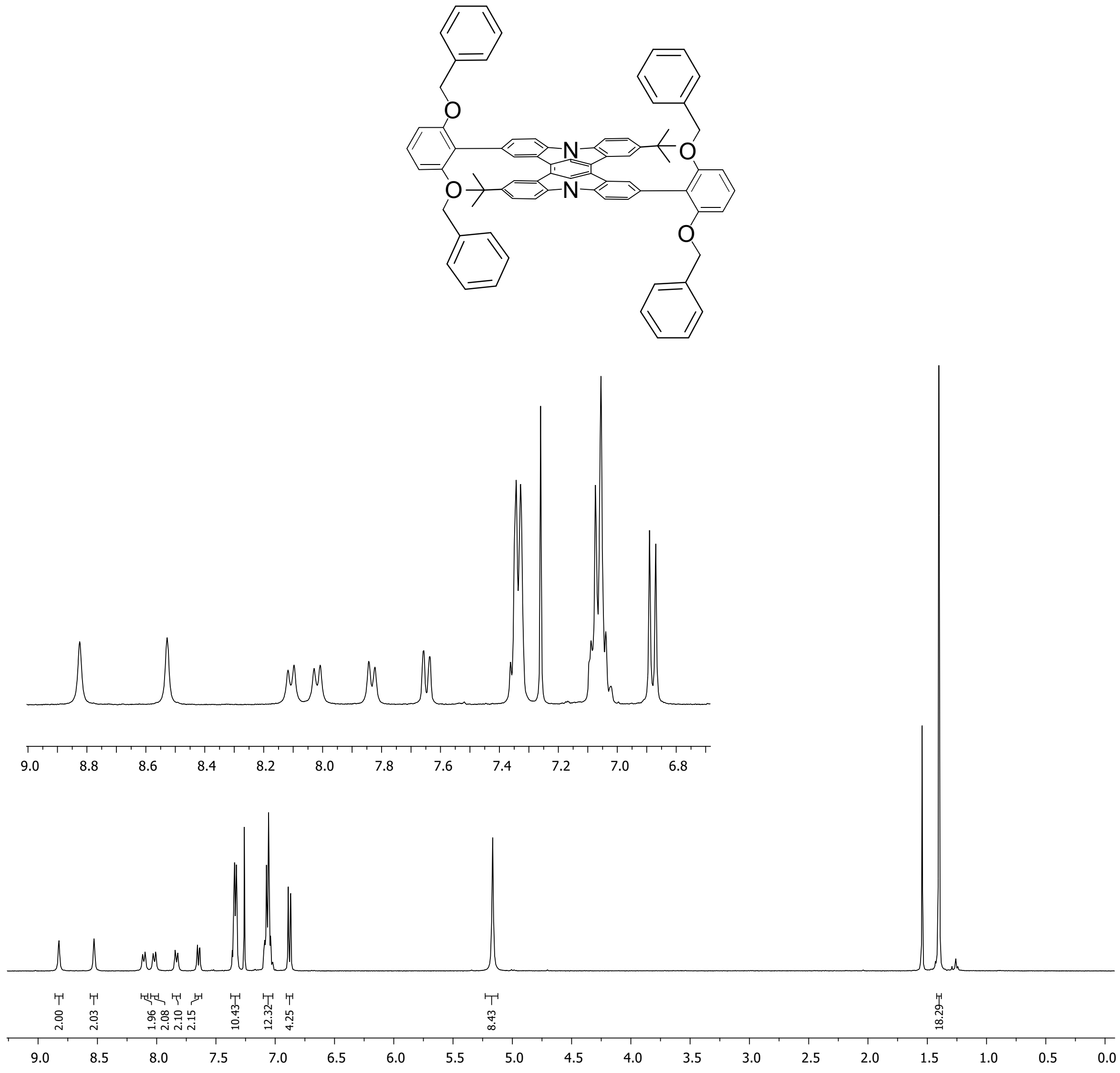

Supplementary Fig. 21: ^{1}H spectrum of **S9**.

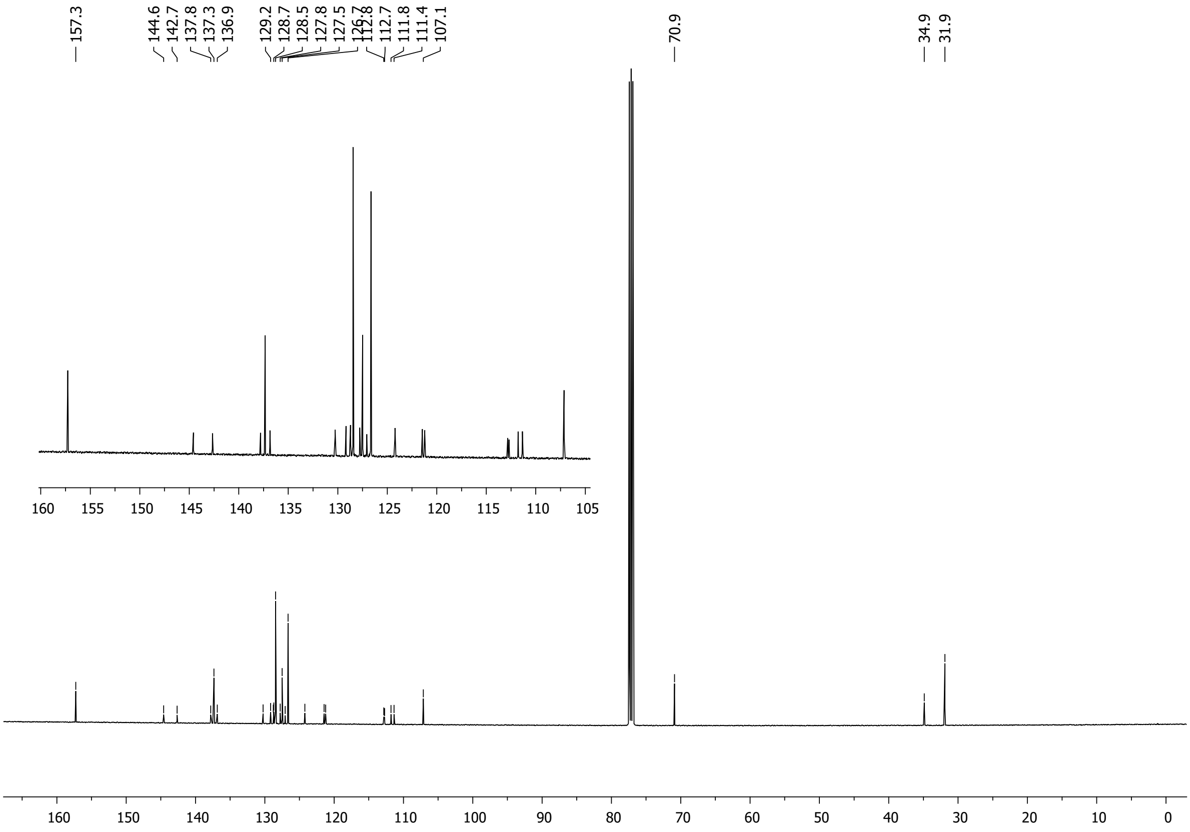

Supplementary Fig. 22: ^{13}C spectrum of **S9**.

Elemental Composition Report

Page 1

Single Mass Analysis

Tolerance = 100.0 PPM / DBE: min = -1.5, max = 50.0
Element prediction: Off
Number of isotope peaks used for i-FIT = 3

Monoisotopic Mass, Even Electron Ions
1 formula(e) evaluated with 1 results within limits (up to 50 closest results for each mass)
Elements Used:
C: 0-78 H: 0-65 N: 0-2 O: 0-4
HAB_D CONGRAVE DGC-10-91repeat
HAB_D CONGRAVE DGC-10-91repeat 528 (1.161) Cm (468:692)

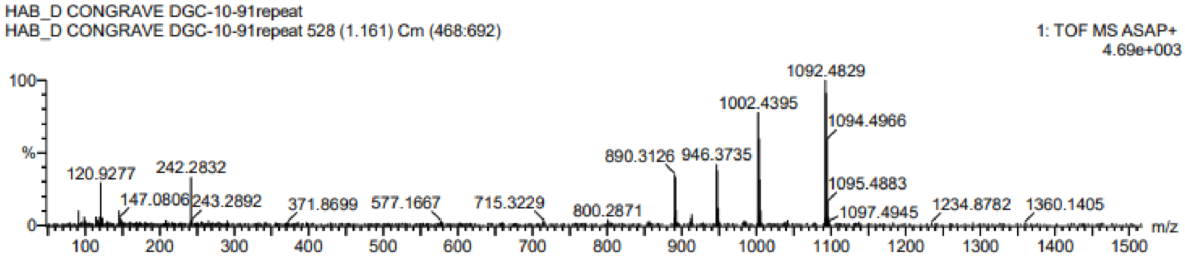

Minimum:				-1.5				
Maximum:		5.0	100.0	50.0				
Mass	Calc. Mass	mDa	PPM	DBE	i-FIT	Norm	Conf(%)	Formula
1093.4896	1093.4944	-4.8	-4.4	47.5	90.1	n/a	n/a	C78 H65 N2 O4

Supplementary Fig. 23: Mass spectrum of **S9**.

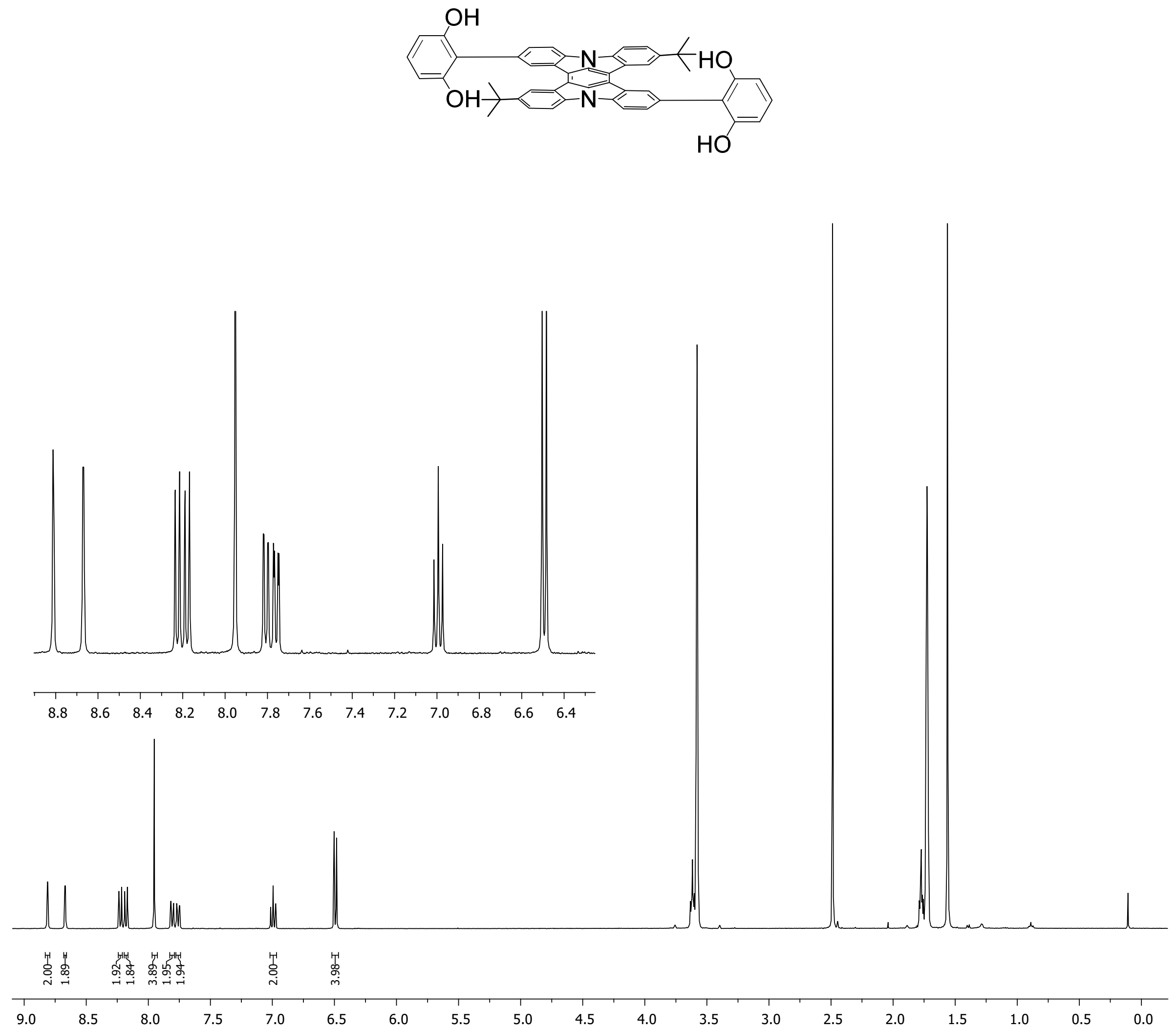

Supplementary Fig. 24: ^{1}H spectrum of **S10**.

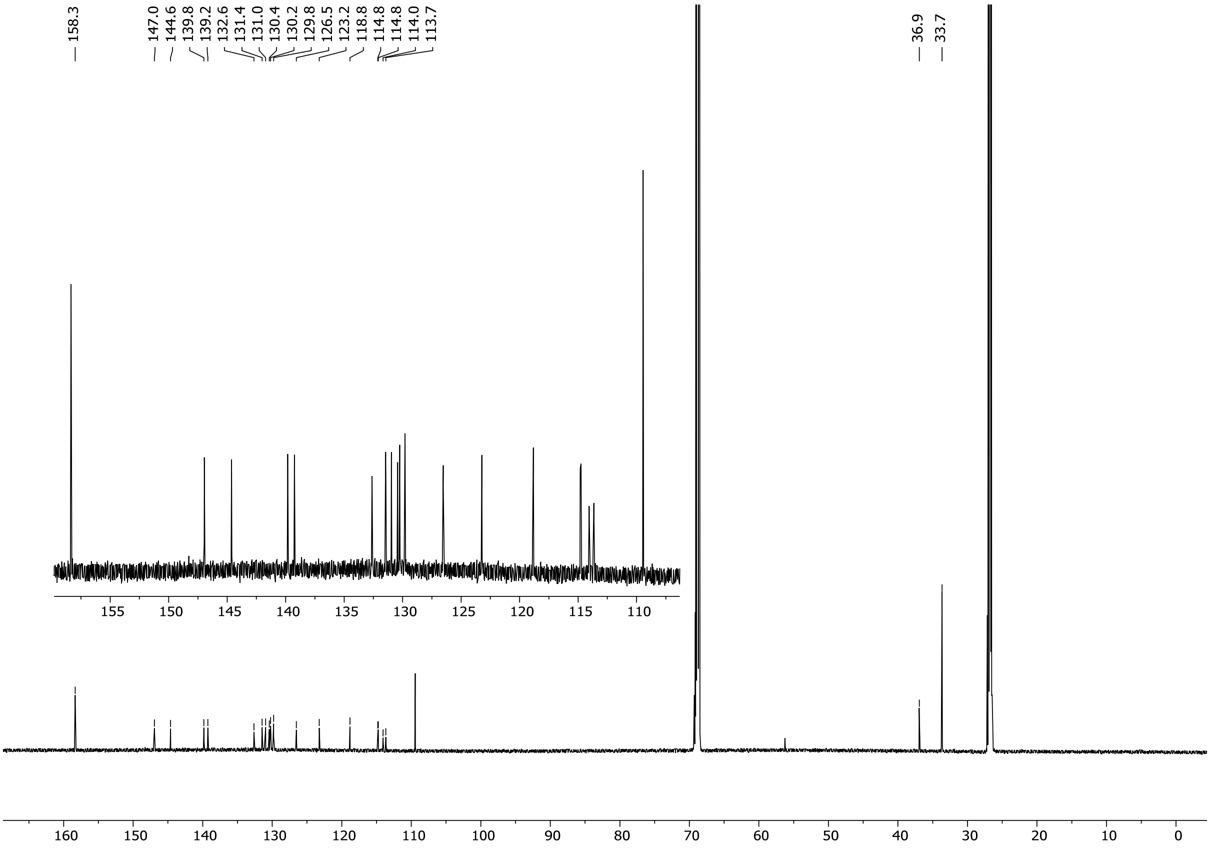

Elemental Composition Report

Page 1

Single Mass Analysis

Tolerance = 100.0 PPM / DBE: min = -1.5, max = 50.0
Element prediction: Off
Number of isotope peaks used for i-FIT = 3

Monoisotopic Mass, Even Electron Ions
4 formula(e) evaluated with 1 results within limits (up to 50 closest results for each mass)
Elements Used:
C: 0-50 H: 0-41 N: 0-2 O: 0-4
HAB_D CONGRAVE DGC-10-92
HAB_D CONGRAVE DGC-10-92 529 (1.163) Cm (521:536)

1: TOF MS ASAP+
8.84e+003

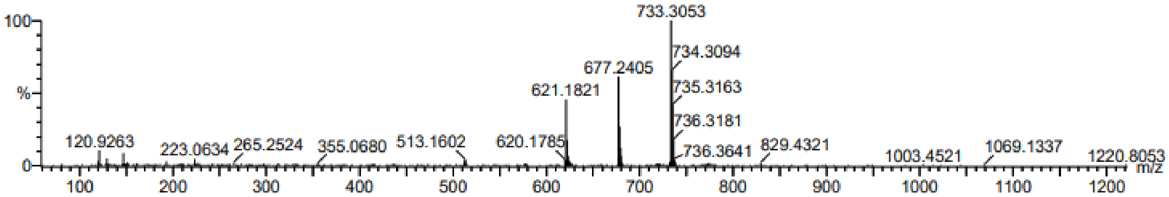

Mass	Calc. Mass	mDa	PPM	DBE	i-FIT	Norm	Conf(%)	Formula
Minimum:				-1.5				
Maximum:		5.0	100.0	50.0				
733.3053	733.3066	-1.3	-1.8	31.5	108.6	n/a	n/a	C50 H41 N2 O4

Supplementary Fig. 25: Mass spectrum of **S10**.

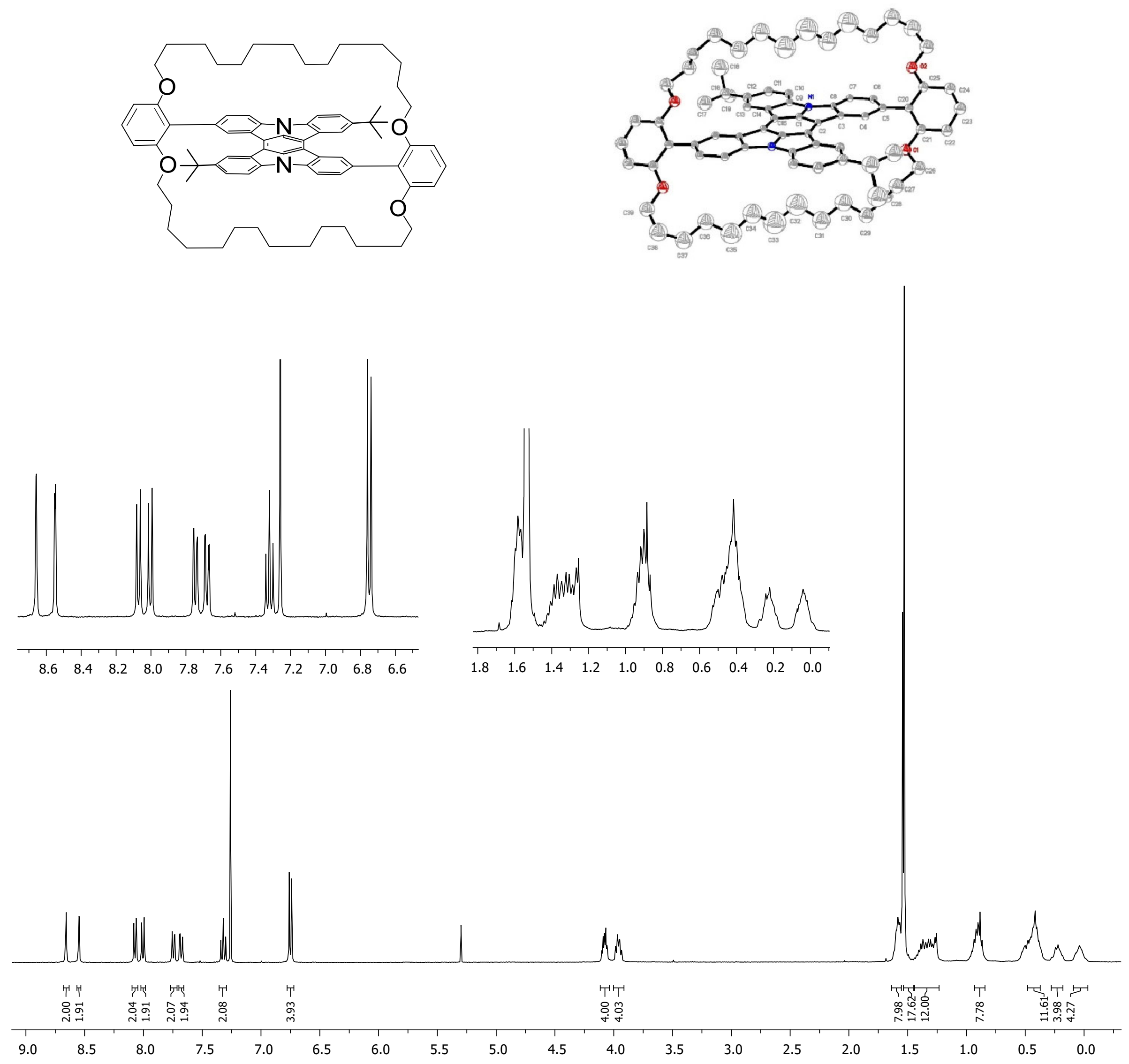

Supplementary Fig. 26: ^{1}H spectrum of **NB-2**.

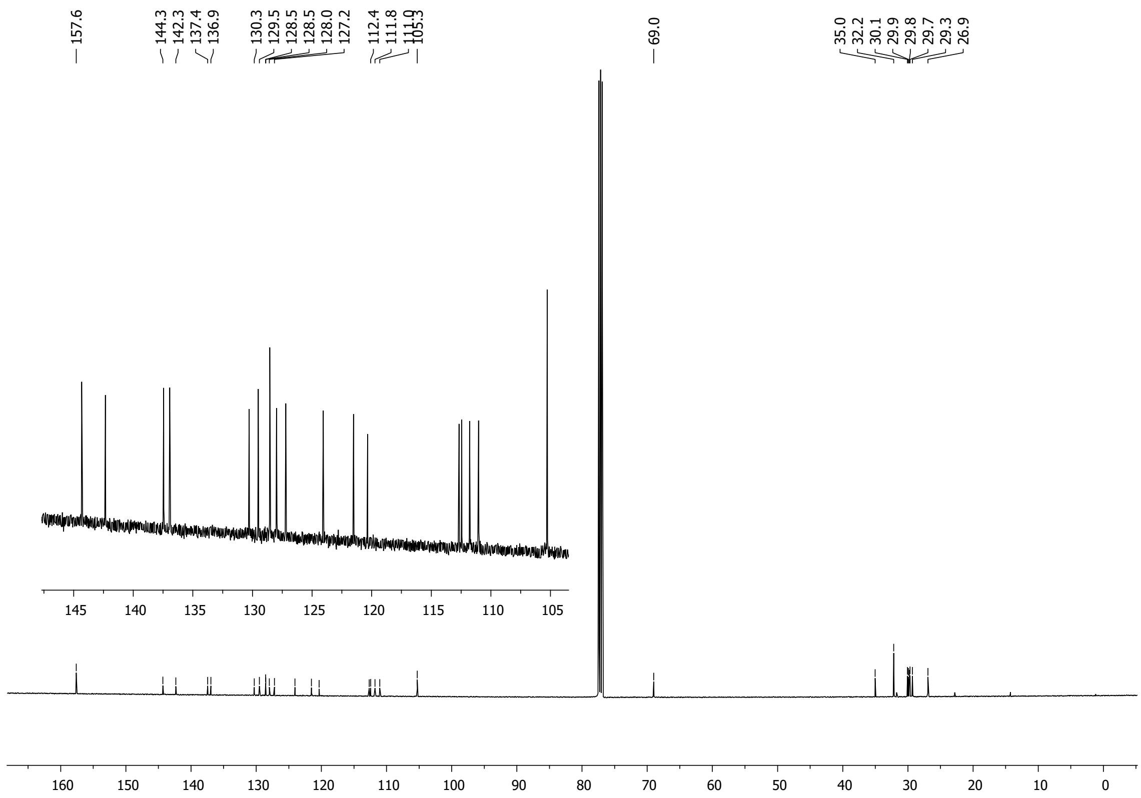

Supplementary Fig. 27: ^{13}C spectrum of **NB-2**.

Elemental Composition Report **Page 1**

Single Mass Analysis
Tolerance = 100.0 PPM / DBE: min = -1.5, max = 50.0
Element prediction: Off
Number of isotope peaks used for i-FIT = 3

Monoisotopic Mass, Even Electron Ions
4 formula(e) evaluated with 1 results within limits (up to 50 closest results for each mass)
Elements Used:
C: 0-78 H: 0-93 N: 0-2 O: 0-4
HAB_D CONGRAVE DGC-10-93
HAB_D CONGRAVE DGC-10-93 290 (0.648) Cm (257:355) 1: TOF MS ASAP+ 4.08e+004

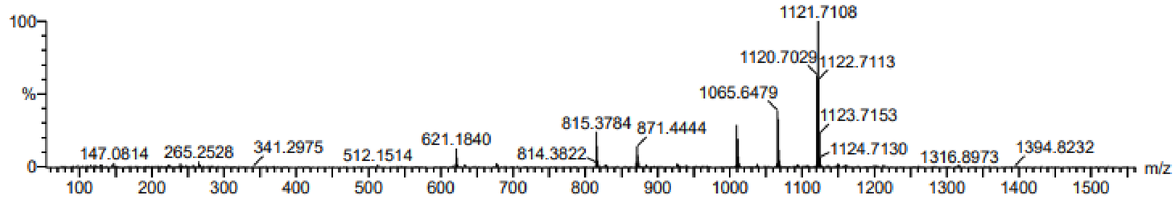

Minimum:				-1.5				
Maximum:		5.0	100.0	50.0				
Mass	Calc. Mass	mDa	PPM	DBE	i-FIT	Norm	Conf(%)	Formula
1121.7108	1121.7135	-2.7	-2.4	33.5	210.2	n/a	n/a	C78 H93 N2 O4

Supplementary Fig. 28: Mass spectrum of **NB-2**.

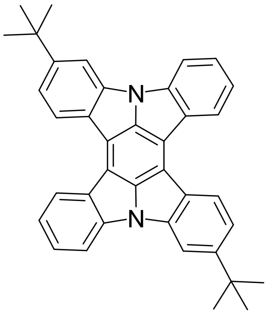

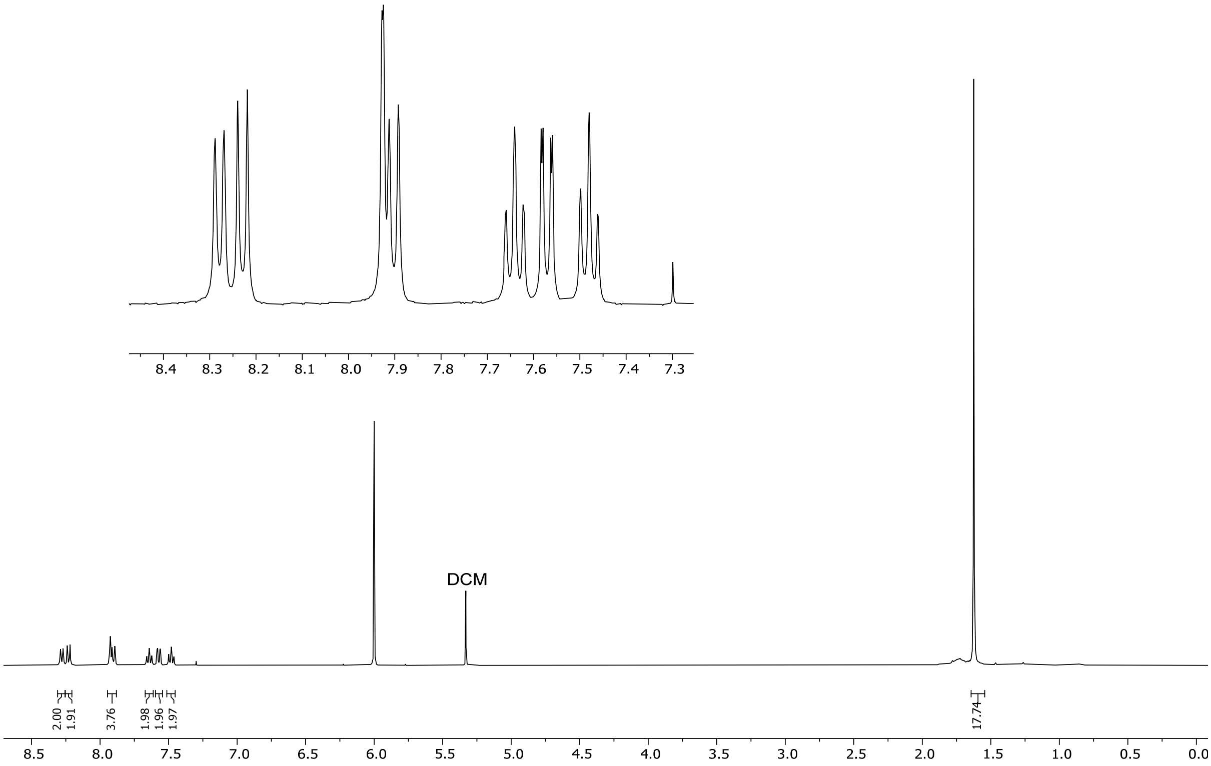

Supplementary Fig. 29: ^{1}H spectrum of **NB-3**.

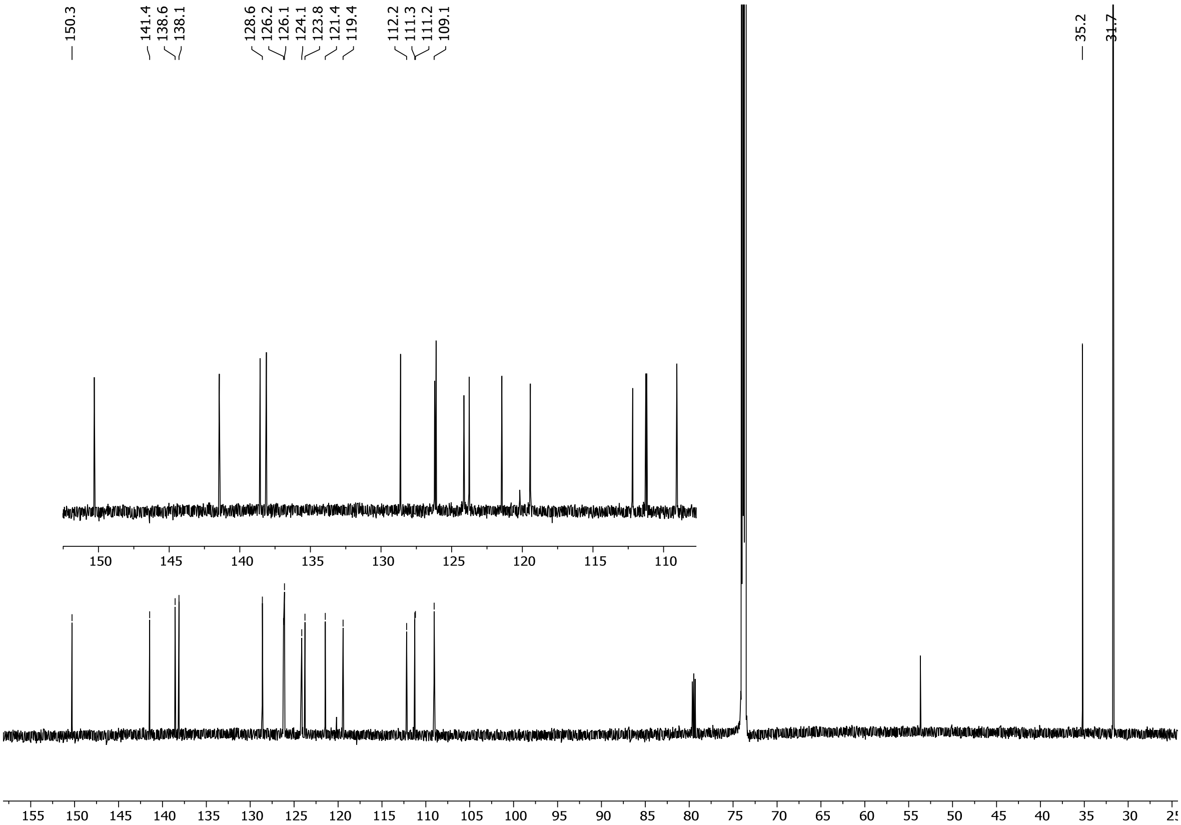

Supplementary Fig. 30: ^{13}C spectrum of **NB-3**.

Elemental Composition Report **Page 1**

Single Mass Analysis
Tolerance = 1500.0 PPM / DBE: min = -1.5, max = 50.0
Element prediction: Off
Number of isotope peaks used for i-FIT = 3

Monoisotopic Mass, Even Electron Ions
1 formula(e) evaluated with 1 results within limits (all results (up to 1000) for each mass)
Elements Used:
C: 0-38 H: 0-33 N: 0-2
HAB_51449 D Congrave DGC-8-16 repeat
HAB_51449 D Congrave DGC-8-16 repeat 2051 (4.397) Cm (1958:2052)

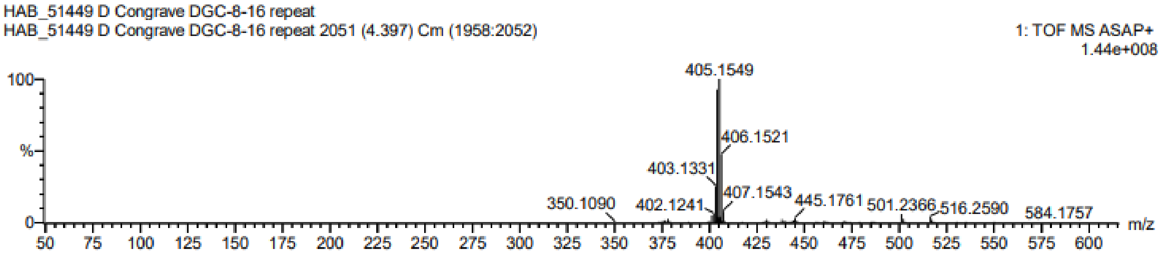

Minimum:				-1.5				
Maximum:		5.0	1500.0	50.0				
Mass	Calc. Mass	mDa	PPM	DBE	i-FIT	Norm	Conf(%)	Formula
517.2625	517.2644	-1.9	-3.7	23.5	855.6	n/a	n/a	C38 H33 N2

Supplementary Fig. 31: Mass spectrum of **NB-3.**

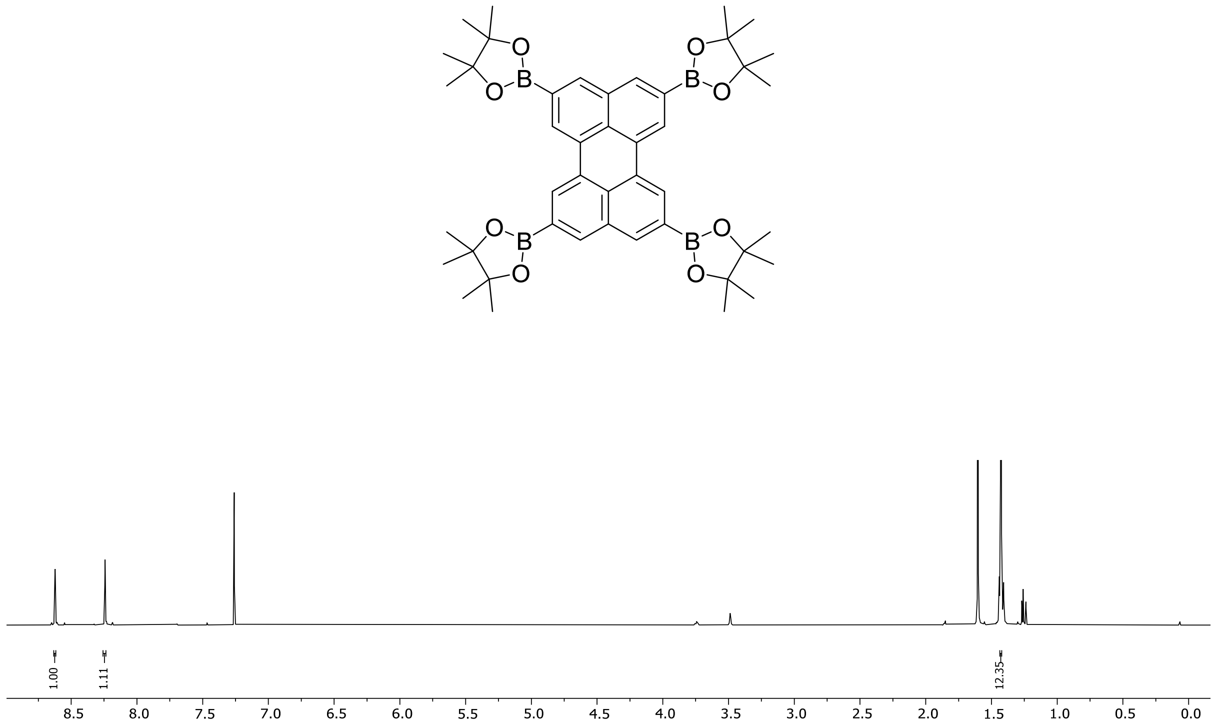

Supplementary Fig. 32: ^{1}H spectrum of **S11**.

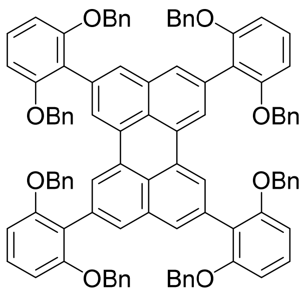

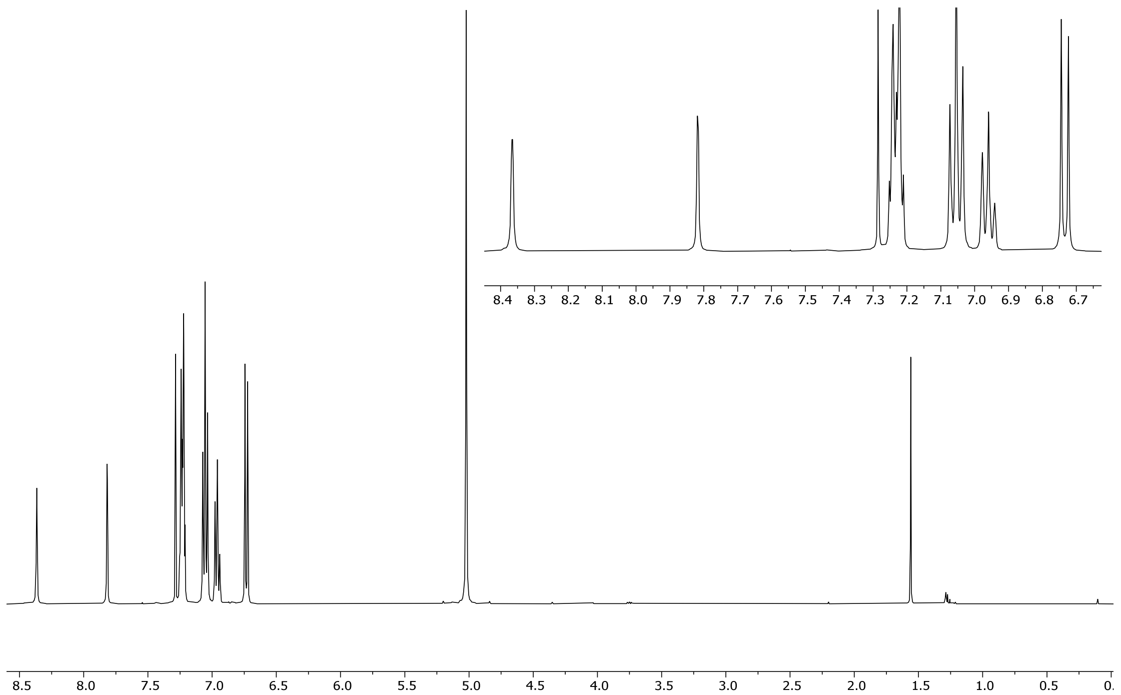

Supplementary Fig. 33: ^{1}H spectrum of **S12**.

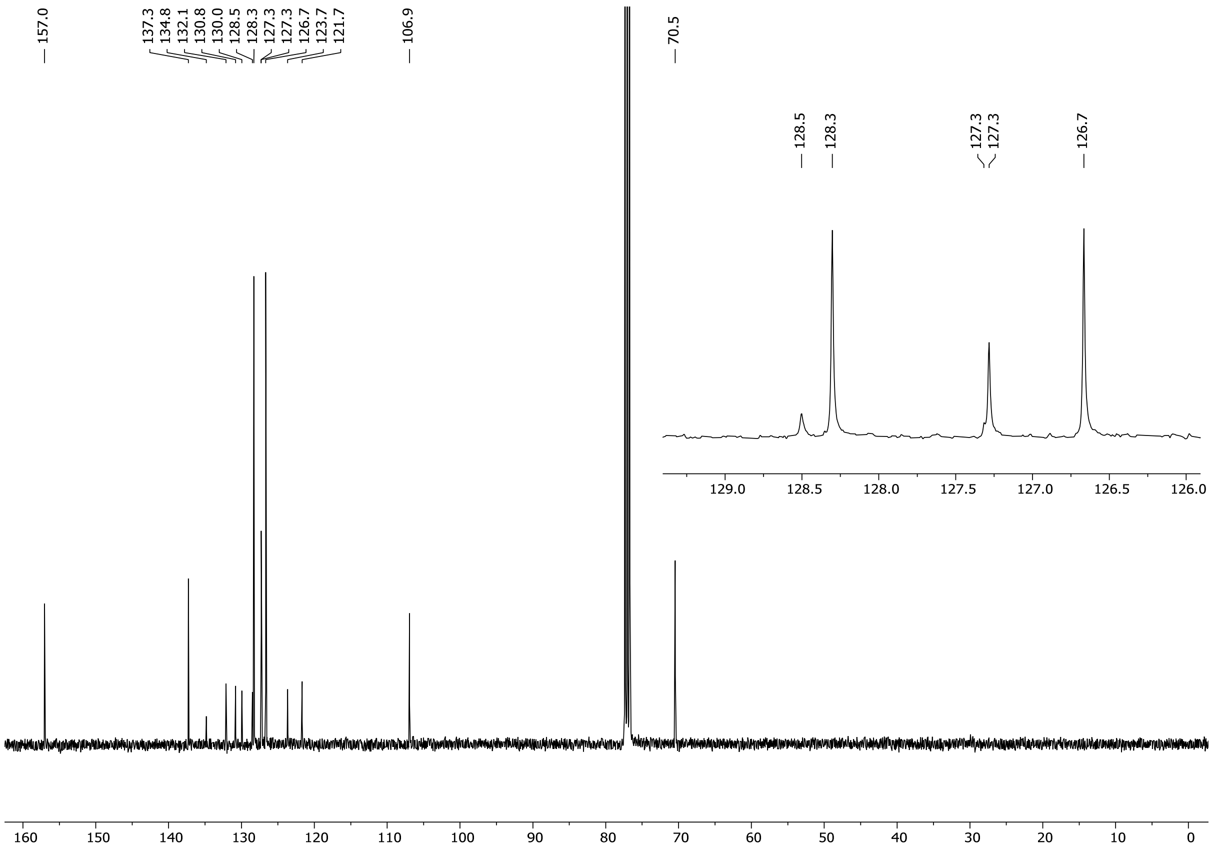

Supplementary Fig. 34: ^{13}C spectrum of **S12**.

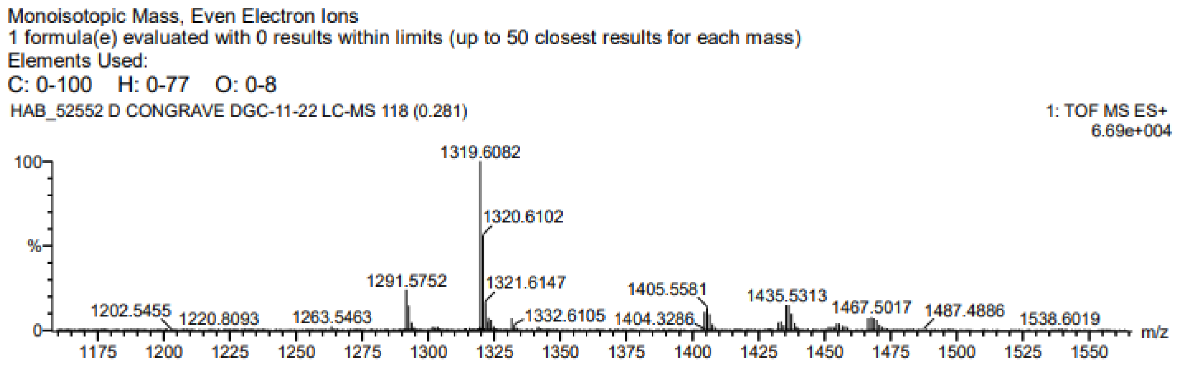

Supplementary Fig. 35: Mass spectrum **S12**.

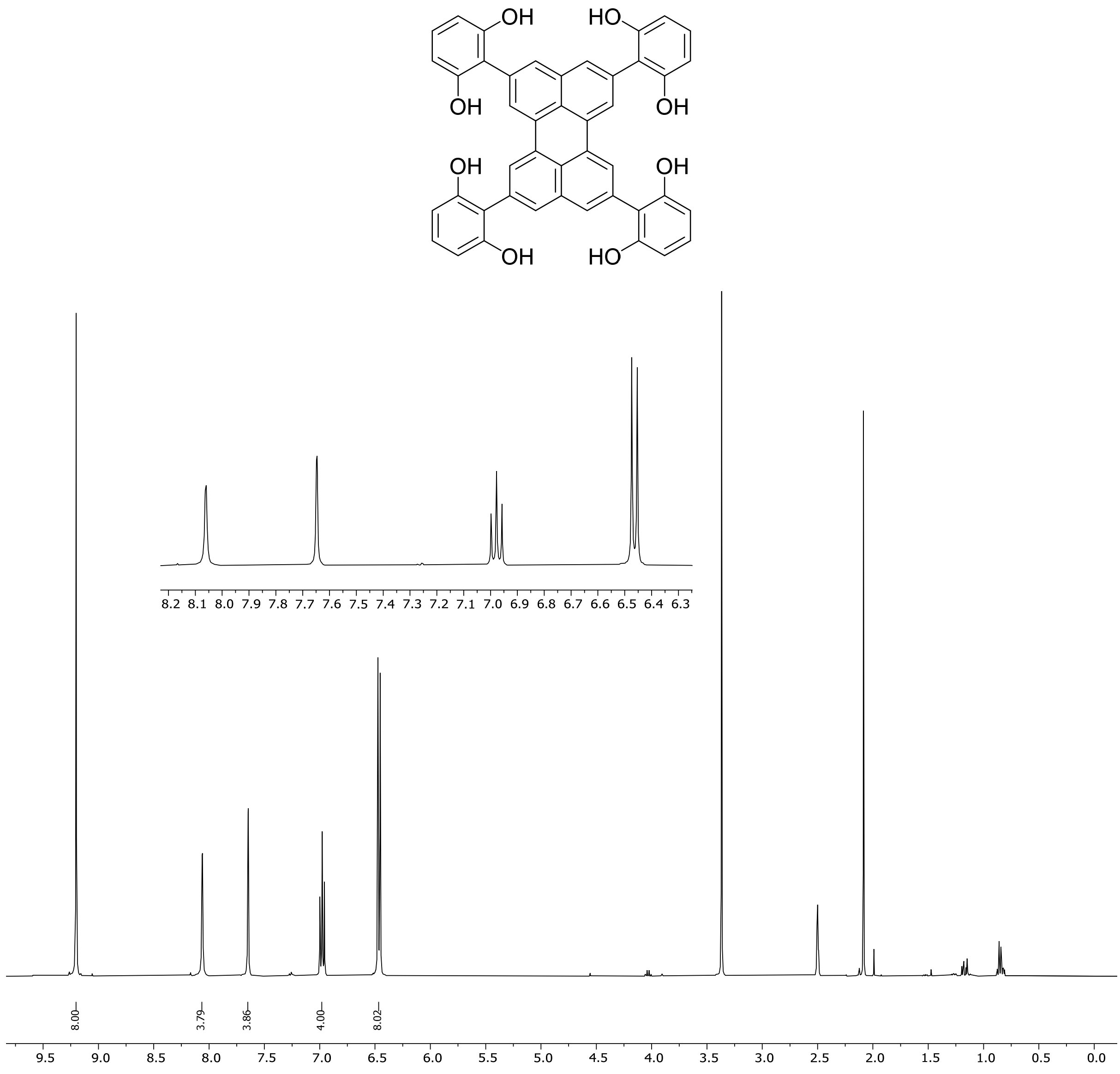

Supplementary Fig. 36: ^{1}H spectrum of **S13**.

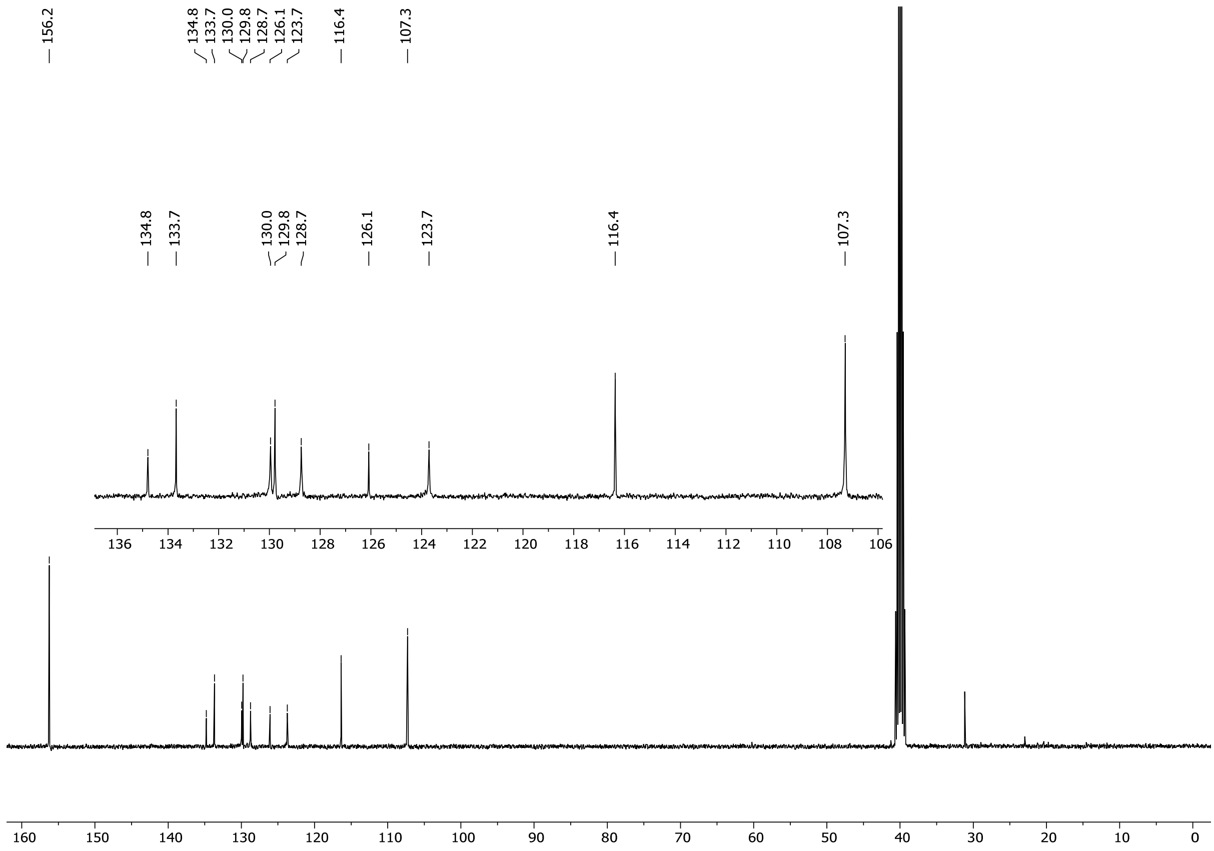

Supplementary Fig. 37: ^{13}C spectrum of **S13**.

Monoisotopic Mass, Even Electron Ions
5 formula(e) evaluated with 1 results within limits (up to 50 closest results for each mass)
Elements Used:
C: 0-44 H: 0-27 O: 0-8

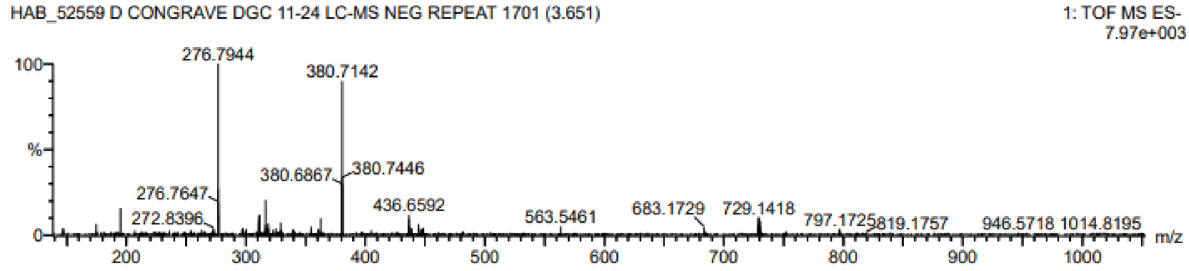

Minimum:				-1.5				
Maximum:		5.0	500.0	50.0				
Mass	Calc. Mass	mDa	PPM	DBE	i-FIT	Norm	Conf(%)	Formula
683.1729	683.1706	2.3	3.4	31.5	77.5	n/a	n/a	C44 H27 O8

Supplementary Fig. 38: Mass spectrum of **S13**.

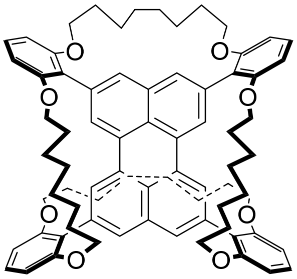

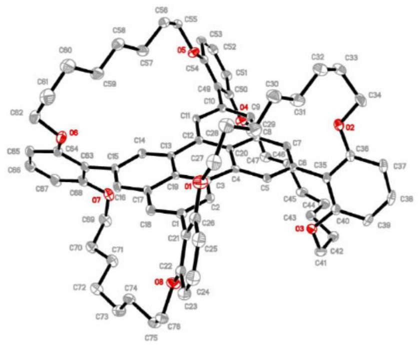

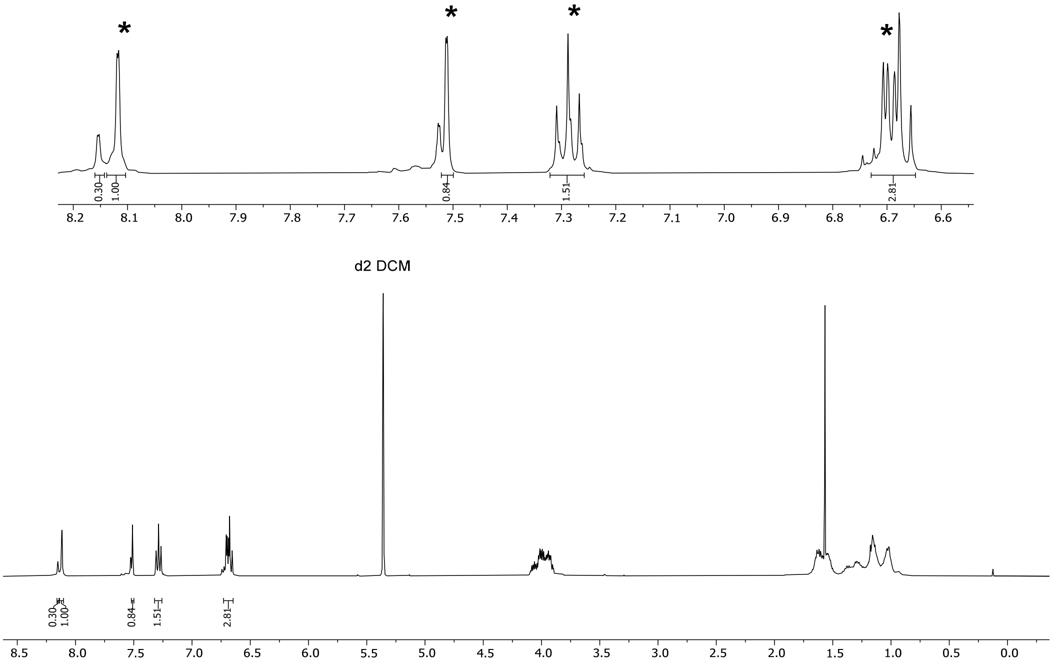

Supplementary Fig. 39: ^{1}H spectrum of **En-Per** mixture after flash chromatography. Product peaks are marked with an asterisk.

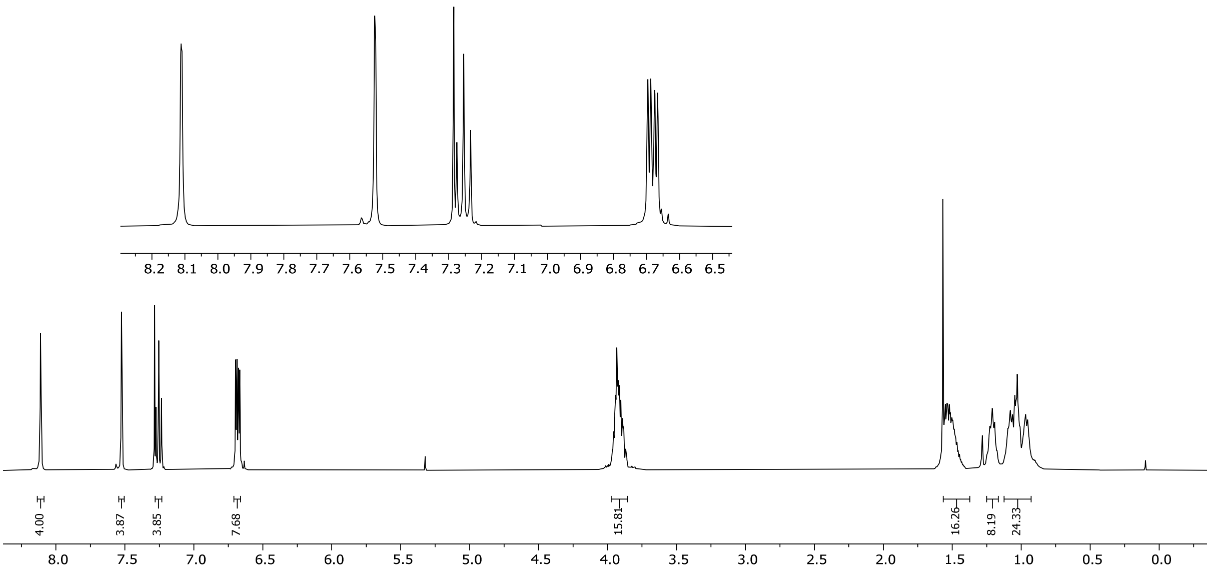

Supplementary Fig. 40: ^{1}H spectrum of **En-Per.**

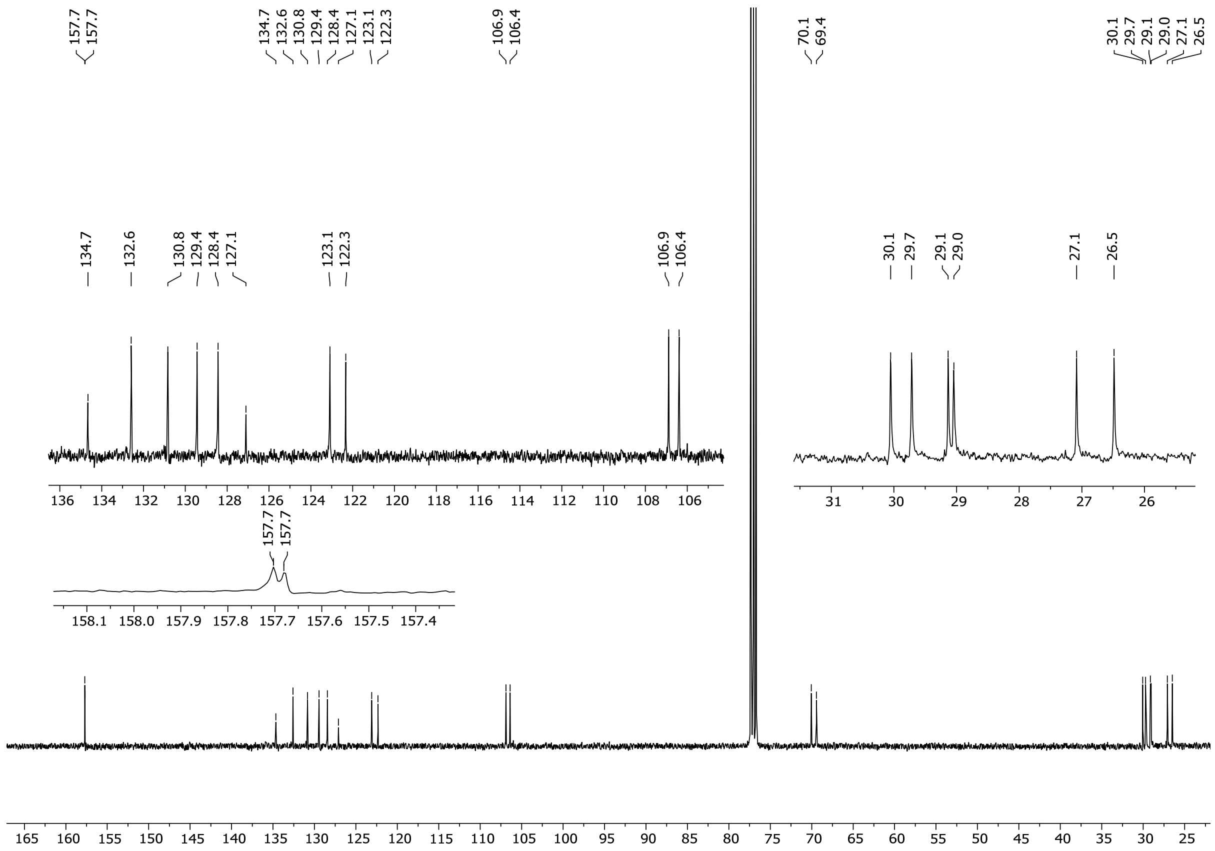

Supplementary Fig. 41: ^{13}C spectrum of **En-Per.**

Monoisotopic Mass, Even Electron Ions
5 formula(e) evaluated with 1 results within limits (up to 50 closest results for each mass)
Elements Used:
C: 0-76 H: 0-85 O: 0-8

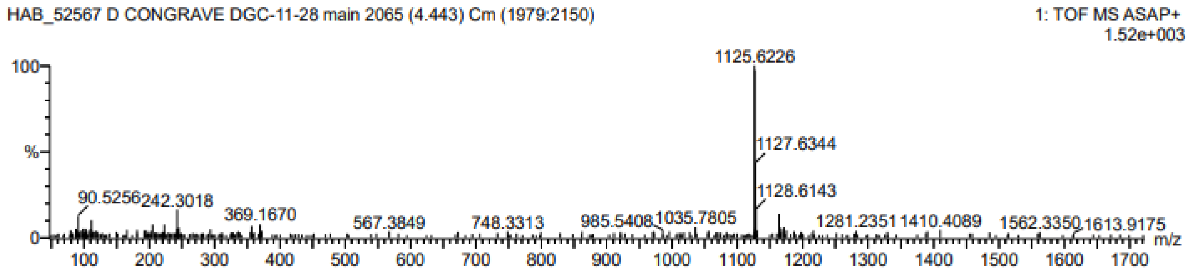

Minimum:				-1.5				
Maximum:		5.0	500.0	50.0				
Mass	Calc. Mass	mDa	PPM	DBE	i-FIT	Norm	Conf(%)	Formula
1125.6226	1125.6244	-1.8	-1.6	34.5	76.5	n/a	n/a	C76 H85 O8

Supplementary Fig. 42: Mass spectrum of **En-Per.**

Supplementary Note 2 - X-Ray crystallography

General

X-ray data were collected on a Bruker D8-QUEST diffractometer, equipped with an Incoatec IμS Cu microsource (λ = 1.5418 Å) and a PHOTON-III detector operating in shutterless mode. Crystals were mounted on a MiTeGen crystal mount using inert polyfluoroether oil and the analysis was carried out under an Oxford Cryosystems open-flow N_2 Cryostream operating at 180(2) K. The control and processing software was Bruker *APEX4*. The diffraction images were integrated using *SAINT* in *APEX4*, and a multi-scan correction was applied using *SADABS*. The final unit-cell parameters were refined against all reflections. Structures were solved using *SHELXT*[13] and refined using *SHELX*.[14] All of the crystal structures include $CHCl_3$ solvent molecules, and the crystals were generally prone to solvent loss and degradation on removal from the mother liquour. In some cases (see below), the *SQUEEZE* algorithm within *PLATON* was applied to complete the refinement.[15] Summary details of the data collections and structure/refinement parameters are given in Supplementary Table 1.

Discussion

S4: The core of the **S4** molecule and one substituent chain (C26-C33) are ordered, except for the penultimate C atom in the chain (C32). The other substituent chain shows disorder. The first component of the disordered chain (C34-C41) has an extended conformation. If this were the only component, voids would exist within the structure in the region between these chains in neighbouring molecules. Instead, it appears that the chain on one molecule adopts the extended conformation, and its neighbour adopts the bent conformation (C34'-C41'), with a $CHCl_3$ molecule filling the remaining space. Hence, the two refined disorder components are grouped as {C34-C41} and {C34'-C41;$CHCl_3$}. A single site occupancy factor (sof) is refined, with the sofs for the two groups constrained to sum to unity. The molecule is situated (on average) on an inversion centre in the applied space group ($P2_1/c$), but it could be that a given molecule has two extended chains or two bent chains, or one of each. The modelled disorder is the best attempt to define this unclear intermolecular region.

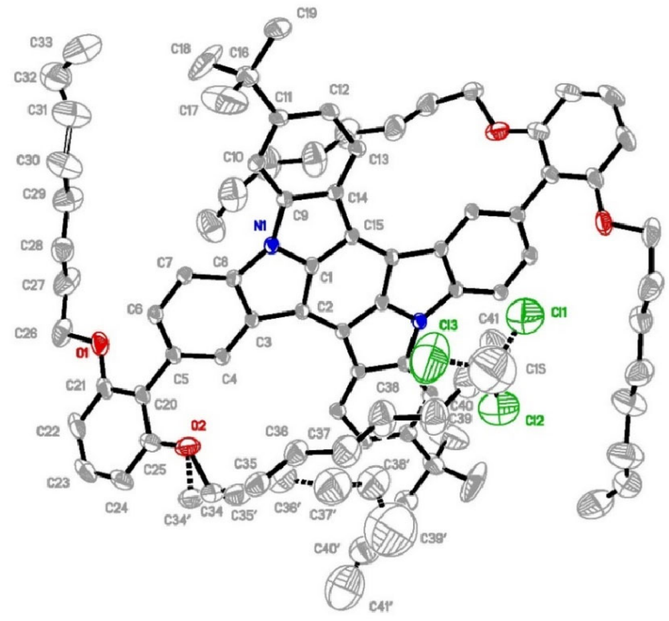

Supplementary Fig. 43: Molecular unit in **S4** showing displacement ellipsoids at 50% probability (H atoms omitted)

S5: The **S5** molecule is situated on an inversion centre with one molecule per unit cell in space group *P*–1. The $CHCl_3$ molecule included in the structure is reasonably well resolved, although the C atom is modelled as split between two sites (*i.e.* the $CHCl_3$ molecule adopts two alternative orientations) and the largest residual peak suggests that further orientations might be modelled. The alternative approach of omitting the $CHCl_3$ molecule from the model and applying *SQUEEZE* improves the R-factors quite significantly (to R1 ca 6%, wR2 ca 17%), which demonstrates that the relatively high reported R-factors can be attributed principally to difficulties with modelling of the solvent molecules. The explicit $CHCl_3$ model was retained to maximise chemical clarity. The structure does not contain any further significant voids, so the reported empirical formula is quite clear.

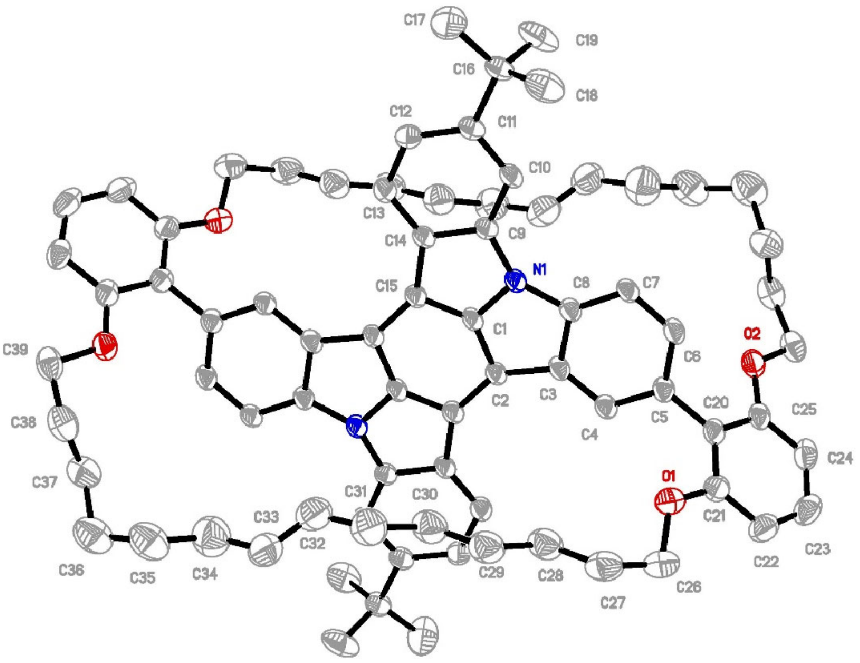

Supplementary Fig. 44: Molecular unit in **S5** showing displacement ellipsoids at 50% probability (H atoms omitted).

<u>NB-1</u>: The **NB-1** molecule is situated on an inversion centre with 9 molecules per unit cell in space group *R*–3. The structure contains two $CHCl_3$ molecules per **NB-1** molecule that are clearly resolved and included in the refinement model. Significant further voids in the structure (ca 10% of the unit-cell volume) must contain further $CHCl_3$, which is difficult to model as discrete molecules. *SQUEEZE* was applied to handle this part of the structure. *SQUEEZE* corrects for 648 electrons per unit cell, which suggests up to 12 further $CHCl_3$ molecules per unit cell. This would give a total empirical formula of 3.3 $CHCl_3$ per **NB-1** molecule. After application of *SQUEEZE*, the largest residual peak in the structure is associated with the $CHCl_3$ molecule that is defined in the model.

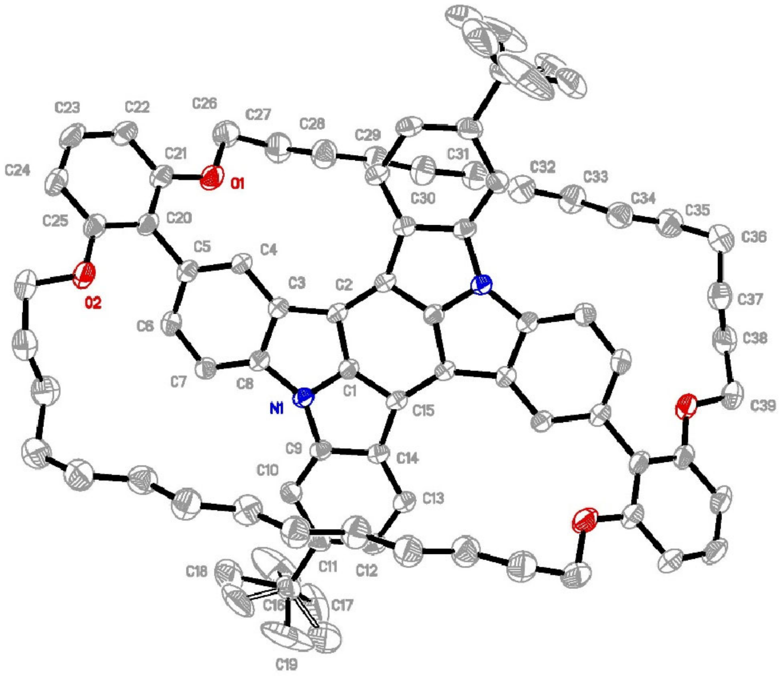

Supplementary Fig. 45: Molecular unit in **NB-1** showing displacement ellipsoids at 50% probability (H atoms omitted)

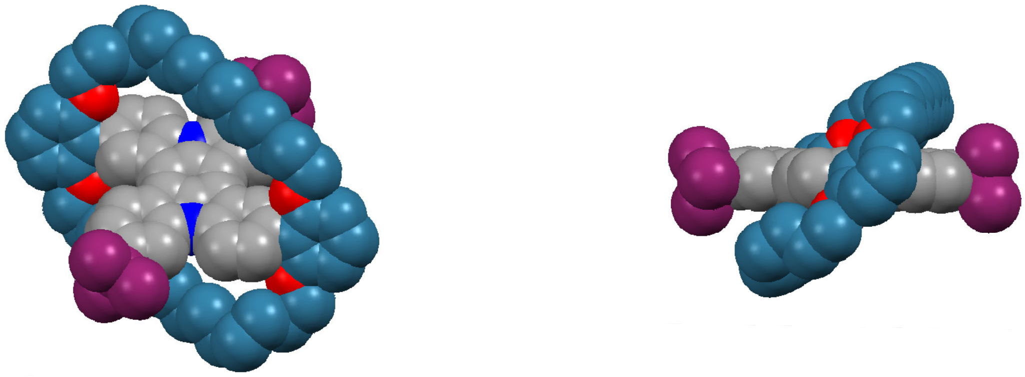

Supplementary Fig. 46: Additional space-fill plots of **NB-1**.

<u>NB-2</u>: The **NB-2** molecule is situated on an inversion centre with 4 molecules per unit cell in space group *P*4/*n*. The core of the **NB-2** molecule is well ordered, while the encapsulating alkyl chain shows larger displacement ellipsoids, particularly at the centre of the chain. Residual peaks in the difference Fourier map suggest likely disorder, but efforts to model that became cumbersome without significantly improving the R-factors; hence the region was left as a single component. The structure contains large channels running along the 4-fold axis (the *c* axis), which must contain $CHCl_3$ solvent. There are two channels per unit cell, summing to ca 25% of the unit-cell volume. It was not possible to identify discrete molecules within the voids, so the *SQUEEZE* algorithm was applied to model the entire solvent region. *SQUEEZE* corrects for 606 electrons per unit cell, which corresponds to ca 20 $CHCl_3$ molecules (*i.e. ca* 5 $CHCl_3$ per **NB-2** molecule). The continuous 1-D nature of the channels makes these crystals particularly prone to solvent loss, and rapid crystal degradation was evident on removal of the crystals from the mother liquor. Several crystals were analysed from two different batches and the reported result is the best obtained. The crystallographic R-factors are high, but the overall packing arrangement of the **NB-2** molecules and the channel-like nature of the structure is clear.

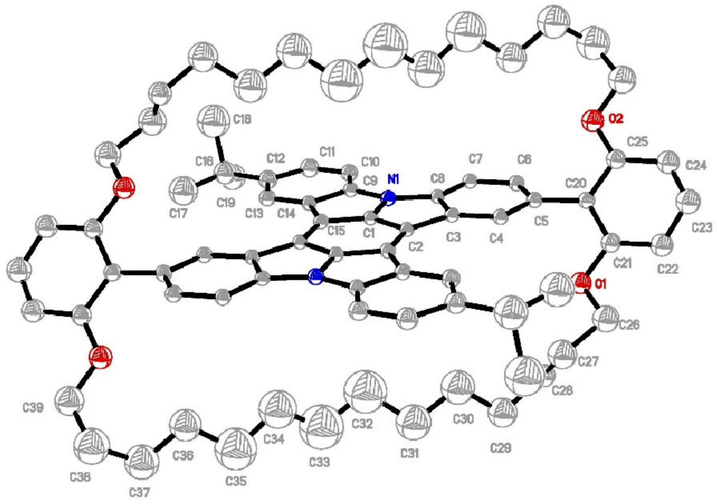

Supplementary Fig. 47: Molecular unit in **NB-2** showing displacement ellipsoids at 30% probability (H atoms omitted)

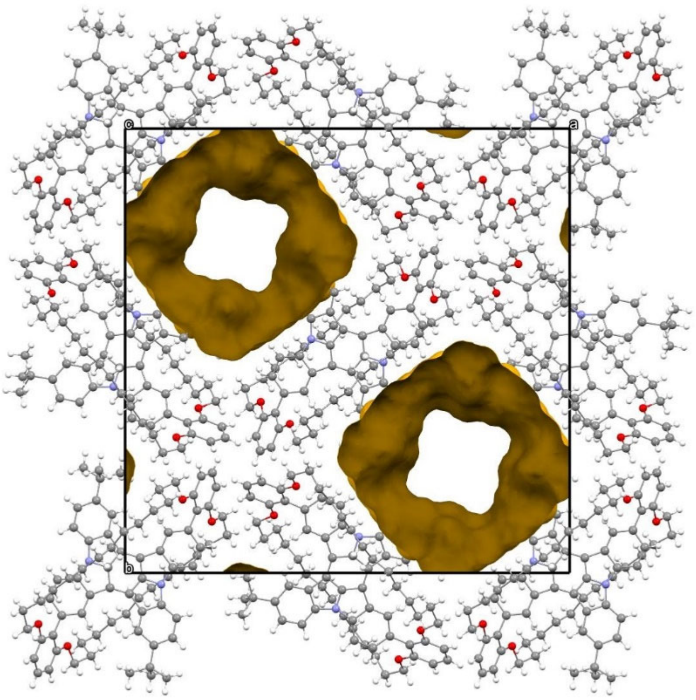

Supplementary Fig. 48: View along channels in crystal structure of **NB-2**. Voids are visualised using *CCDC Mercury*.

En-Per: The molecule of **En-Per** is well ordered in the crystal, including the alkyl chains. The perylene core is twisted quite substantially, with an angle of 12.7° between the planes defined by the two naphthalene rings. The crystal structure contains four DCM molecules per formula unit. The positions of these molecules are clear, but conventional refinement of the C and Cl atoms does not proceed especially well, yielding $wR2 \approx 0.25$. An alternative approach of omitting the DCM molecules and applying *SQUEEZE* improves $wR2$ to 0.15, adding confidence that the moderately high *R*-factors can be attributed principally to inadequate modelling of the solvent molecules. The conventional refinement of the DCM molecules is ultimately preferred, however, since it shows the interaction between the DCM molecules and the perylene core.

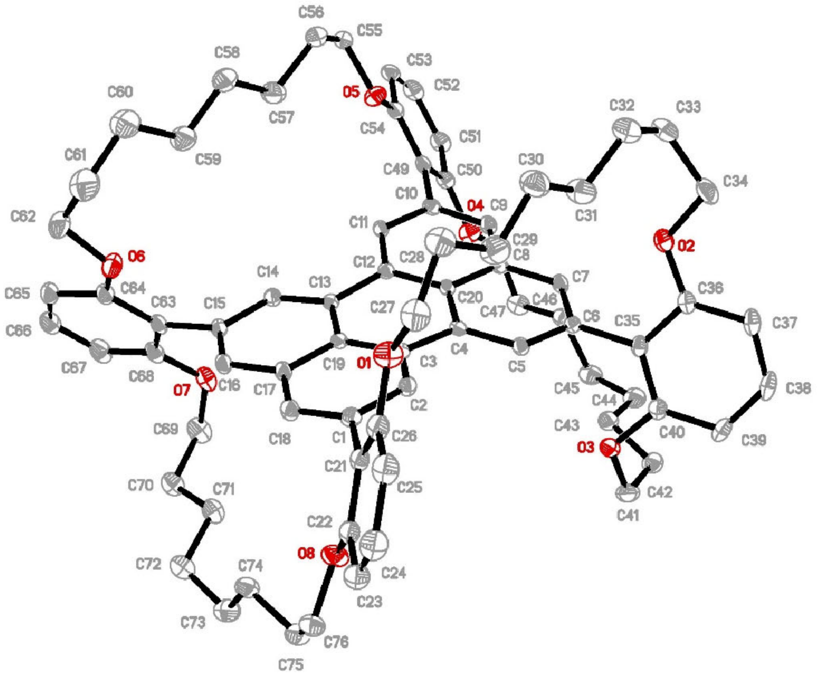

Supplementary Fig. 49: Molecular unit in **En-Per** showing displacement ellipsoids at 50% probability (H atoms omitted).

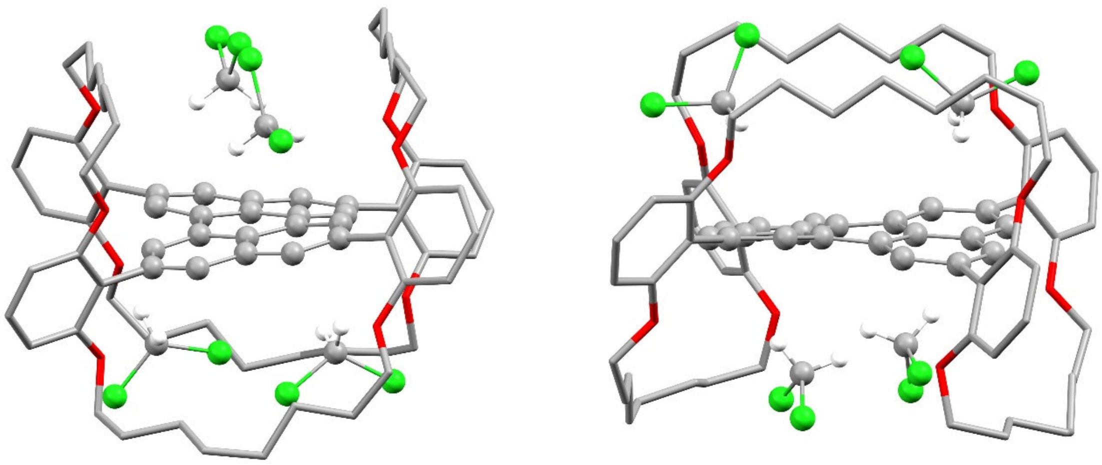

Supplementary Fig. 50: Interaction between DCM molecules and the twisted perylene core in the crystal structure of **En-Per**.

Supplementary Table 1: Summary of crystal and refinement data

	S4	S5	NB1_sq**	NB2_sq**	En-Per
CCDC number	2236338	2236336	2236337	2236339	2282946
Cambridge data number	HB_B1_0044	HB_B1_0045	HB_B2_0024	HB_B1_0055	HB_B2_0042
Chemical formula	$C_{82.83}H_{96.83}Cl_{2.48}N_2O_4$	$C_{80}H_{90}Cl_6N_2O_4$	$C_{80}H_{94}Cl_6N_2O_4$	$C_{78}H_{92}N_2O_4$	$C_{80}H_{92}Cl_8O_8$
Moiety formula	$C_{82}H_{96}N_2O_4$ + 0.83 $CHCl_3$	$C_{78}H_{88}N_2O_4$ + $2CHCl_3$	$C_{78}H_{92}N_2O_4$ + *x* $CHCl_3$	$C_{78}H_{92}N_2O_4$ + *x* $CHCl_3$	$C_{76}H_{84}O_8$ + $4CH_2Cl_2$
Formula weight	1272.08	1356.23	1360.27	1121.53	1465.13
Temperature / K	180(2)	180(2)	180(2)	180(2)	180(2)
Crystal system	monoclinic	triclinic	trigonal	tetragonal	triclinic
Space group	$P\ 2_1/c$	$P\ –1$	$R\ –3$	$P\ 4/n$	$P\ –1$
a / Å	14.2866(5)	10.6437(5)	34.5344(14)	30.2147(7)	12.4929(7)
b / Å	34.2877(13)	12.5447(6)	34.5344(14)	30.2147(7)	12.7370(7)
c / Å	7.5834(3)	15.1157(7)	17.3470(8)	8.2131(4)	24.8820(13)
alpha / °	90	101.774(2)	90	90	79.782(3)
beta / °	93.380(2)	92.373(2)	90	90	85.461(3)
gamma / °	90	110.794(2)	120	90	78.677(3)
Unit-cell volume / $Å^3$	3708.3(2)	1832.77(15)	17916.7(17)	7498.0(5)	3816.6(4)
Z	2	1	9	4	2
Calc. density / g cm^{-3}	1.139	1.229	1.135	0.994	1.275
F(000)	1364	718	6498	2424	1544
Radiation type	Cu Kα	Cu Kα	Cu Kα	Cu Kα	Cu Kα
Absorption coefficient / mm^{-1}	1.322	2.524	2.324	0.461	3.123
Crystal size / mm^3	0.20 x 0.20 x 0.10	0.30 x 0.15 x 0.10	0.32 x 0.32 x 0.14	0.16 x 0.08 x 0.08	0.20 x 0.16 x 0.12
2-Theta range / °	5.15–133.23	6.02–133.55	5.89–133.23	4.14–133.37	7.18–133.58
Completeness to max 2θ	0.973	0.976	0.999	0.998	0.980
No. of refl. Measured	39977	20627	85158	37903	58996
No. of independent refl.	6361	6342	7035	6636	13272
R(int)	0.0422	0.0306	0.0420	0.0888	0.0471
No. parameters / restraints	516 / 200	419 / 6	449 / 54	379 / 283	871 / 86
Final *R*1 values ($I > 2\sigma(I)$)	0.0883	0.0909	0.0670	0.1728	0.0936
Final wR(F^2) values (all data)	0.2695	0.2539	0.1771	0.5074	0.2555
Goodness-of-fit on F^2	1.031	1.085	1.078	1.024	1.045
Largest diff. peak & hole / e $Å^{-3}$	0.708, –0.404	1.431, –1.221	1.357, –0.974	1.340, –0.630	1.473, –1.164

** The *SQUEEZE* algorithm has been applied to account for un-modelled solvent content in these structures (see descriptions above).

Supplementary Note 3 - Theoretical calculations

Computational details

Computations were performed using (time-dependent) density functional theory (TD-DFT) as implemented in Orca 5.0.[16] Ground- and excited-state structure optimizations, Hessians, and vibronic coupling parameters were computed at the ωB97X-D3/def2-SVP level of theory.[17,18] Vertical excitations are reported at the LC-BLYP/def2-TZVP level using a range separation parameter of μ = 0.1 a.u. chosen to reproduce experimentally observed singlet and triplet excitation energies.[19]

Absorption and emission spectra were computed using a path integral approach[20] as implemented in the ORCA ESD module, employing ωB97X-D3/def2-SVP Hessians along with LC-BLYP/def2-TZVP excitation energies.

A linear vibronic coupling model (LVC)[21] was used for interpreting spectral line shapes. Here, the Huang-Rhys factor for normal mode *i* was computed as

$$S_i = \frac{\kappa_i^2}{\omega_i^2}$$

where κ_i is the vibronic coupling parameter and ω_i is the wavenumber for mode i, both inserted in atomic units and computed with respect to ground-state modes. These computations were carried out using the LVC functionality of SHARC.[18,19]

Natural transition orbitals[24] were computed using the TheoDORE program.[25]

Molecular volume

Supplementary Table 2: Calculated molecular volumes

Molecule	Volume / Å^3 [(a)]	Extra volume compared to mDICz / Å^3
**mDICz**	476.3	-
**NB-1**	1496.1	1019.8
**NB-3**	647.2	170.9

[(a)]Calculated on B3LYP/6-31G* optimised structures via a marching tetrahedron model.[26]

Narrowband emission

Within this section we present a discussion of the molecular structure, vertical excitations and computed spectra of mDICz. Results on mDICz are contrasted to perylene-diimide (PDI) to highlight the unique properties of the former.

In its ground-state structure mDICz is distinguished by a strongly distorted benzene ring that "pre-relaxes" the molecule for the S_1 state. An analysis of the vertical excitations shows that these are mostly of locally excited character, but some charge transfer character is present due to the involvement of the nitrogen atoms.

The computed emission spectrum reflects the narrow shape found in experiment. An analysis of the emission spectrum shows that the second peak seen in the spectrum is a vibronic feature related to breathing and distortion of the central benzene ring. The Franck-Condon activity of this breathing mode is strongly reduced when compared to the analogous mode in PDI.

Molecular structure of mDICz

The ground-state optimized molecular structure of mDICz is shown in Supplementary Fig. 51 (a). The crucial feature of this structure is the strongly strained central benzene ring. Bond angles around this ring are 133.9° and 113.1° strongly deviating from the idealized value of 120° found for an isolated benzene molecule. The S_1-optimized structure of mDICz is shown in Supplementary Fig. 51 (b). Its bond angles are even more strongly distorted (135.1° and 112.5°) away from the idealized values for benzene. A superposition of ground- and excited state is shown in Supplementary Fig. 51 (c). This representation highlights the structural rigidity of the system. However, structural rigidity alone is not sufficient to explain the narrow line width as seen for the PDI case, below.

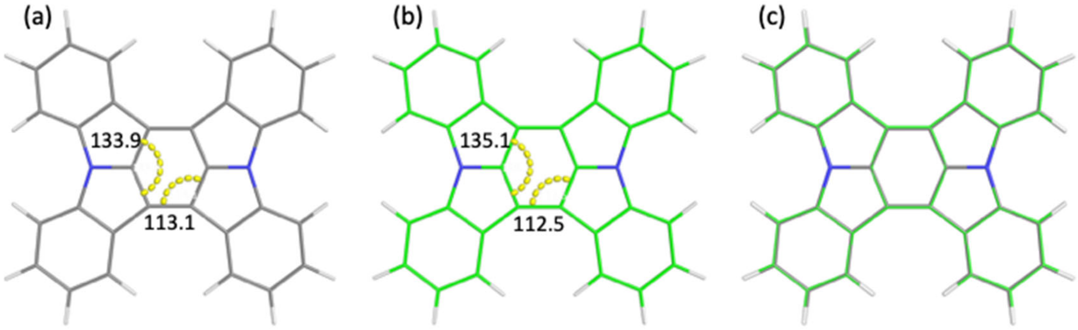

Supplementary Fig. 51: Molecular structures of mDICz optimized for (a) the ground state and (b) the S_1 state. A superposition of the structures is shown in (c). Bond angles around the central benzene ring are shown in (a) and (b).

Vertical excitations of mDICz

Time-dependent density functional theory (TD-DFT) computations were performed to elucidate the excited states of mDICz. Vertical excitation energies of singlet and triplet states computed at the S_0 and S_1 minimum geometries are presented in Supplementary Table 3. The T_1 and T_2 states at the S_0 geometry lie at 2.317 and 2.570 eV respectively. These are followed by the S_1 state with a vertical absorption energy of 2.891 eV. Relaxation of S_1 lowers its energy only slightly and the vertical emission is at 2.794 eV.

Supplementary Table 3: Vertical excitation energies (ΔE, eV) and oscillator strengths for selected excited states of meta-dicz computed at the S_0 and S_1 geometries using the LC-BLYP level of theory.

Geom.	State	ΔE	f
S_0 min.	T_1	2.317	-
S_0 min.	T_2	2.570	-
S_0 min.	S_1	2.891	0.128
S_1 min.	S_1	2.794	0.203

For a graphical representation of the electronic transition we computed the natural transition orbitals (NTO) for the S_1 and T_1 states (Supplementary Fig. 52). The NTOs are distributed fairly evenly over the molecule with the exception that the two nitrogen atoms only contribute to the hole (i.e. they act as donors). Thus, the excited state possesses a partial charge transfer character. When comparing the S_1 and T_1 states one finds that the hole for S_1 is more strongly localized on the nitrogen atoms whereas enhanced contribution for the central benzene ring is found for T_1. This produces enhanced charge transfer character for S_1 and enhanced local character for T_1. These variations in excited-state character may be responsible for the comparatively small singlet-triplet gap despite large orbital overlap.

	Hole	Electron
S_1 (94%)	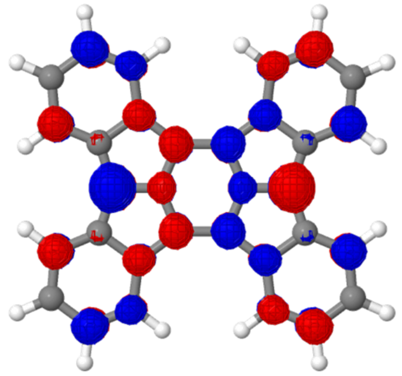	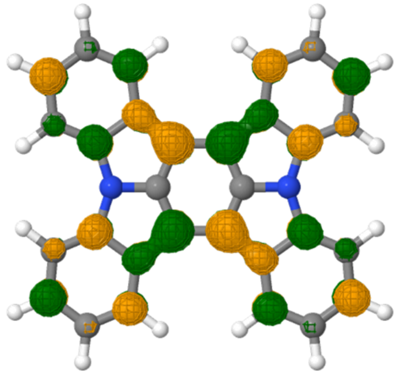
T_1 (99%)	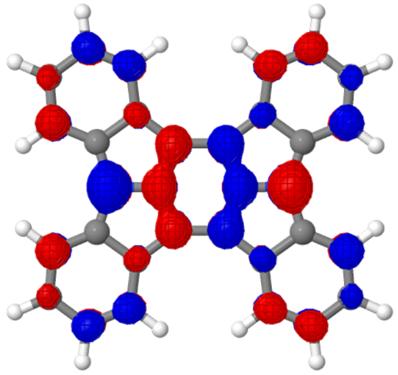	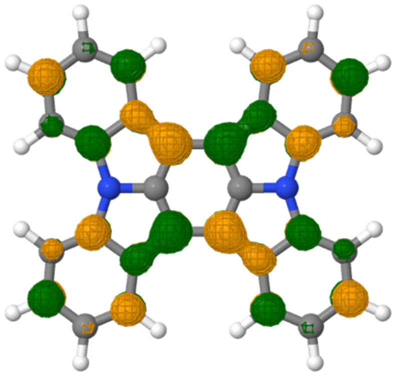

Supplementary Fig. 52: Dominant pair of natural transition orbitals for the S_1 and T_1 states of mDICz computed at the LC-BLYP level of theory.

Absorption and emission spectra of mDICz

Absorption and emission spectra were computed as described in the Computational Details. The simulated spectra for mDICz are presented in Supplementary Fig. 53. The simulated spectra resemble the experimental ones showing one narrow peak with substantial overlap between absorption and emission. The absorption maximum is computed at 430 nm (23,269 cm^{-1}), the emission maximum at 433 nm (23,101 cm^{-1}) featuring a Stokes shift of only 168 cm^{-1}.

In line with experiment, the emission spectrum features a second smaller peak. This peak is located at 463 nm (21,593 cm^{-1}) shifted from the main peak by 1508 cm^{-1}.

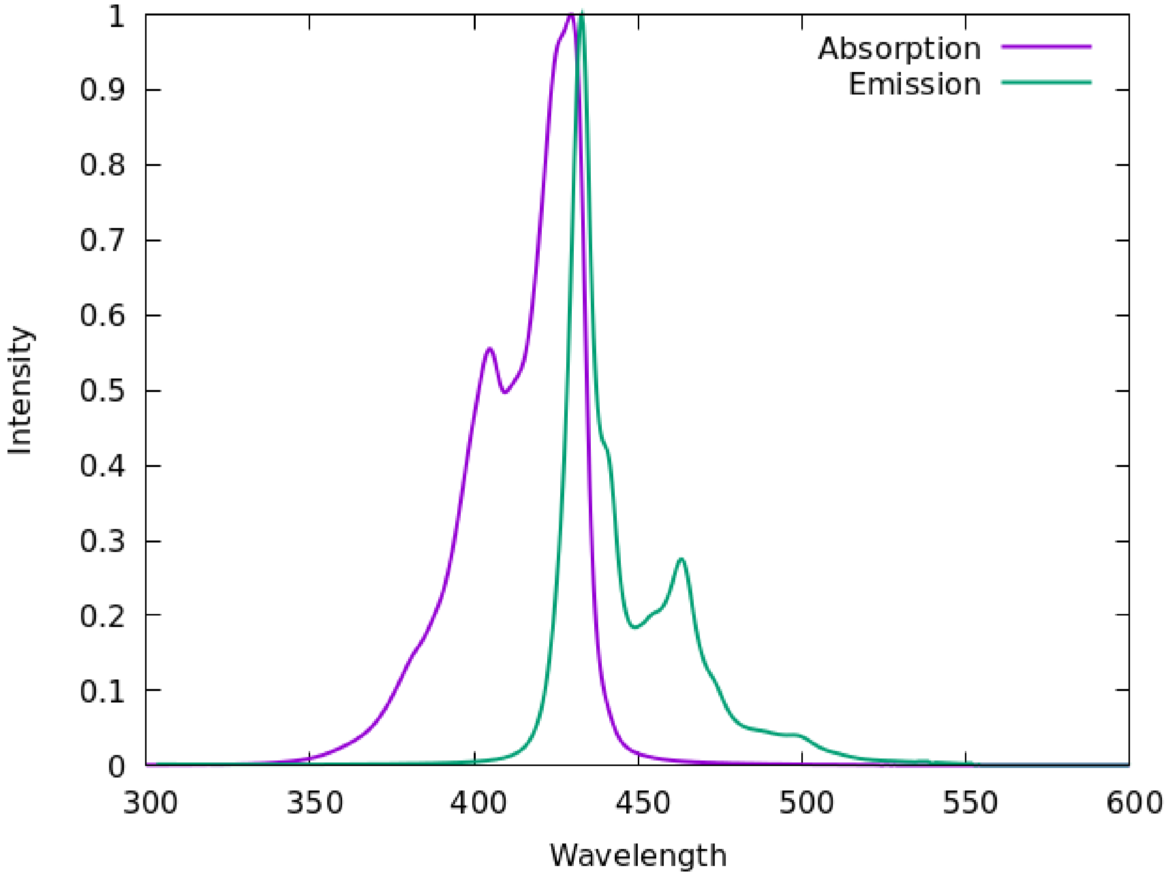

Supplementary Fig. 53: Computed absorption and emission spectra for mDICz.

To obtain more insight into the spectral broadening we computed Huang-Rhys factors as described in the Computational Details (above). Larger Huang-Rhys factors are generally associated with Franck-Condon active modes producing enhanced spectral broadening. Specifically, the overlap of the ground-state vibrational wavefunctions is given as[27]

$$\left|\langle \chi_{0,i}' | \chi_{0,i} \rangle\right|^2 = \exp(-S_i) \qquad (1)$$

where S_i is the Huang-Rhys factor for mode i. Thus, the intensity of the 0-0 transition decays exponentially with the Huang-Rhys factor.

The computed Huang-Rhys factors are presented in Supplementary Fig. 54. The largest Huang-Rhys factor (0.285) is observed for a mode lying at 139 cm^{-1} involving in-plane motion of the four phenyl rings. Note, however, that this mode is hidden within the overall width of the main peak and does not contribute to any noticeable vibronic progression. For all Huang-Rhys factors corresponding to modes above 250 cm^{-1} values below 0.15 are obtained meaning that according to Eq. (1) these contribute to the main 0-0 peak with over 86%. We can now proceed to assign the second peak shown in the emission spectrum (Fig. S3.3), which appears 1508 cm^{-1} below the main peak. Two modes with substantial Huang-Rhys factors are found around this area (at 1469 cm^{-1} and 1553 cm^{-1}) and we suggest that both contribute to this peak. Both modes involve breathing of the central benzene ring as well shortening of the adjacent CN bonds. In addition, they feature further distortion of the central benzene ring from an ideal hexagon (see also Supplementary Fig. 51).

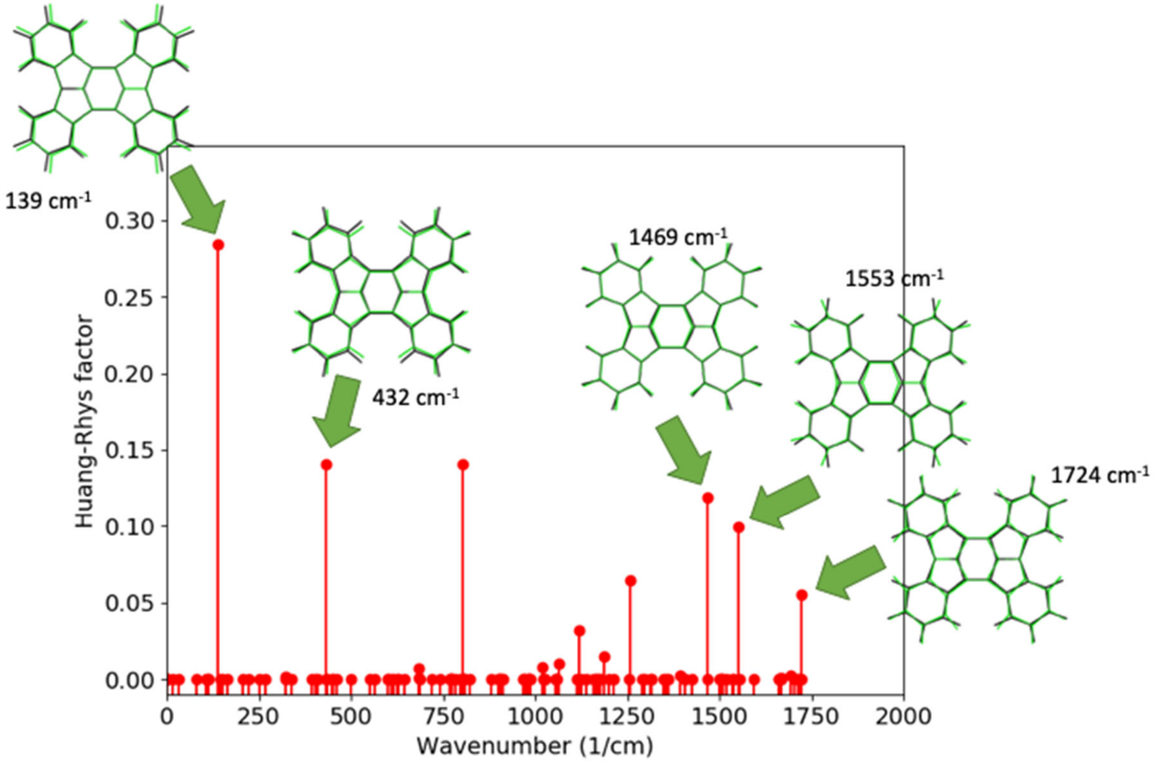

Supplementary Fig. 54: Huang-Rhys factors computed for mDICz. Selected modes are shown as inset.

Comparison to perylene-diimide

Having discussed the spectra of mDICz, we will now proceed to a discussion of perylene-diimide (PDI). On the one hand, PDI resembles mDICz in being a heteroaromatic polycyclic system. On the other hand, it is crucially different through its broad vibronic progression.

We start by a presentation of the molecular structures optimized for the ground state and S_1 state (Supplementary Fig. 55). This representation resembles mDICz, as shown in Supplementary Fig. 51 c), in the sense that there is almost no apparent difference between the structures. They have the same symmetry, with a central benzene ring fused into a polycyclic structure. This indicates that structural rigidity alone is not enough to explain the narrow emission of mDICz.

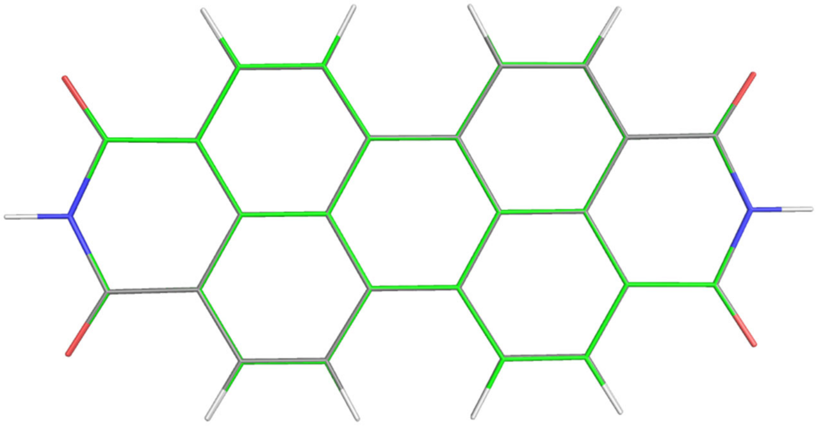

Supplementary Fig. 55: Superposition of optimized structures for PDI in the ground state (black) and S_1 state (green).

The computed absorption and emission spectra of PDI are shown in Supplementary Fig. 56. A clear vibronic progression is seen with peaks separated by about 1600 cm^{-1}. This vibronic progression is reflected by enhanced Huang-Rhys factors as shown in Supplementary Fig. 57. Importantly, the dominant modes (at 1454 and 1700 cm^{-1}) have a similar appearance to the dominant modes of mDICz, i.e. breathing of the central ring and “squeezing” of the adjacent bonds. The associated Huang-Rhys factors are significantly enhanced causing the difference in spectral shape.

In summary, we conclude that the mechanism for spectral broadening in mDICz and PDI is very similar involving related breathing modes. But mDICz is distinguished in suppressing the activity of these modes.

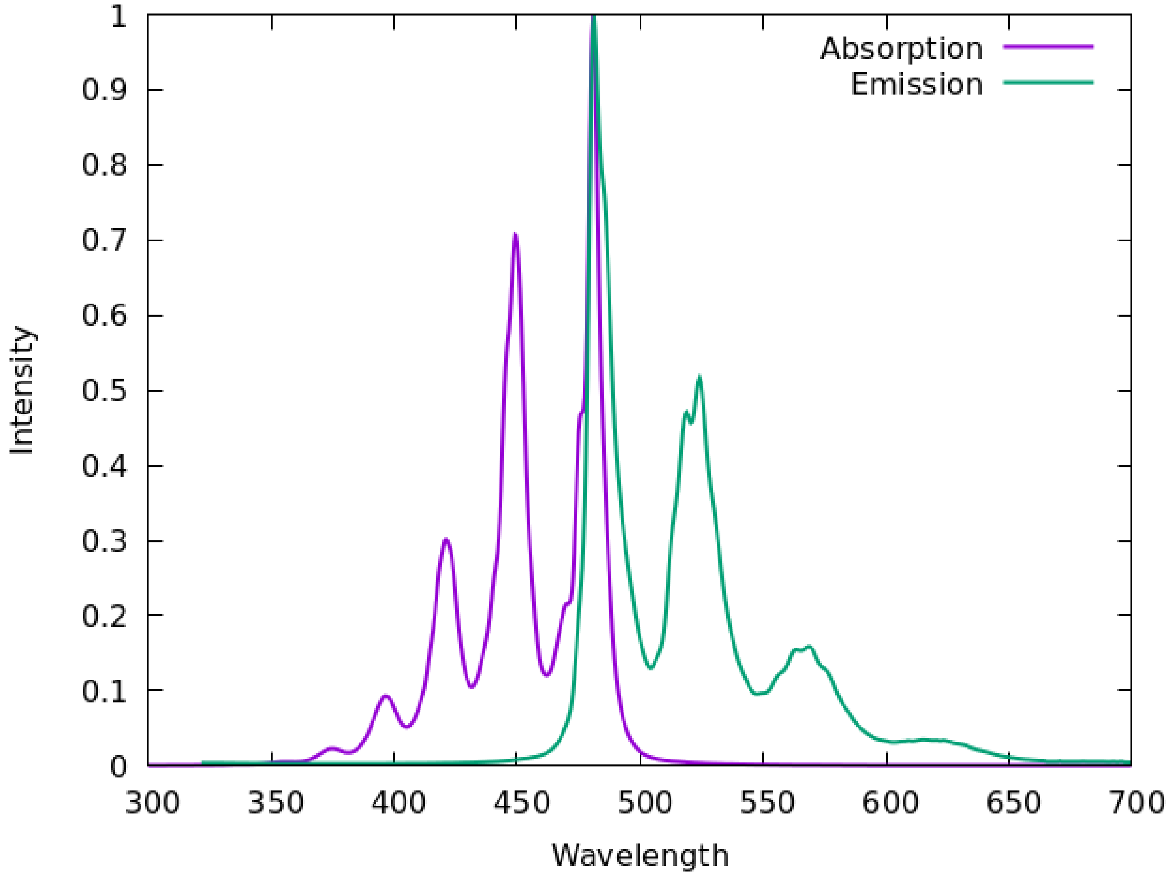

Supplementary Fig. 56: Computed absorption and emission spectra for PDI.

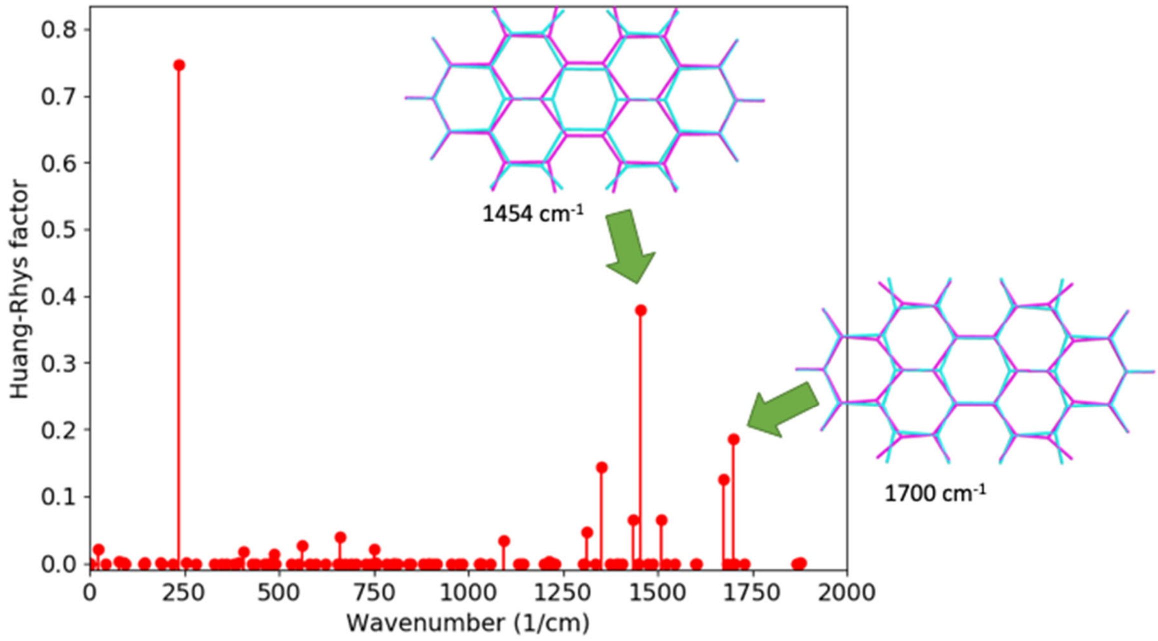

Supplementary Fig. 57: Huang-Rhys factors for PDI. Selected modes are shown as inset.

Supplementary Note 4 - Thermal analysis

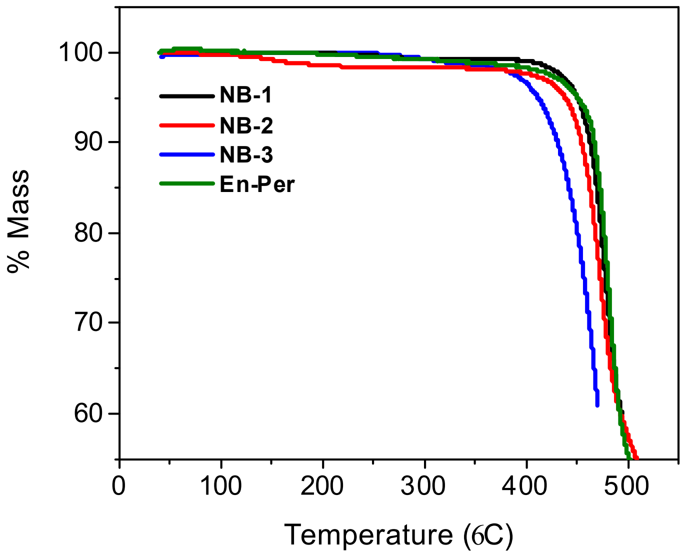

Supplementary Fig. 58: TGA traces for **NB-1, NB-2** and **NB-3**. 5% Mass loss (T_d) occurs at and 450 °C, 440 °C, 410 °C and 450 °C for **NB-1, NB-2**, **NB-3** and **En-Per**, respectively. The initial mass loss below 100 °C for **NB-2** is ascribed to trapped solvent. OLED films were fabricated in temperature ranges of 200–220 °C and 220–240 °C for **NB-3** and **NB-1/ NB-2**, respectively.

Supplementary Note 5 - Absorption, photoluminescence and electrochemistry

General

Solution absorption spectra were measured using a Shimadzu UV-1800. Molar extinction coefficients were determined from triplicate runs using a method intended to minimise weighing and dilution error. For each run >3 mg of the compound was accurately weighed into a 25 mL volumetric flask to make a stock solution. 100 uL of the stock solution was titrated 5 times into a 1 cm path length cuvette (2 mL starting blank solvent volume) measuring absorption spectra after each addition. Fluorescence and time correlated single photon counting experiments were carried out on an Edinburgh instruments FS5. Solution photoluminescence quantum yield measurements were carried out in an integrating sphere on an Edinburgh instruments FS5.

For organic films for photophysics, glass substrates (steady-state PL and absorption, PLQE, and transient PL) and quartz substrates (TA) were prepared, and they were cleaned with acetone and isopropyl alcohol with sonification for ten minutes before loading them into an evaporator (manufactured by Angstrom Engineering). 50 nm and 100 nm thick films were formed on glass and quartz substrates, respectively. The thermal evaporating process was conducted in a vacuum chamber under $< 5 \times 10^{-7}$ mBar.

Steady-state PL spectra were measured by an Edinburgh Instruments fluorescence spectrometer (FLS980) with a monochromated xenon arc lamp at λ_{Ex} = 330 nm under a nitrogen flow. FLS980 with an integrating sphere under a nitrogen flow was used to measure PLQE, and the films were excited by 330 nm laser. Transient PL was recorded by using an Andor electrically gated intensified charge-coupled device (ICCD) with 330 nm laser excitation; the decay kinetics were obtained from the integration of the total spectrum at each time.

Cyclic voltammetry was carried out using a PalmSens EmStat4S at a scan rate of 100 mV s^{-1}. Solutions were prepared in dry, degassed THF with 0.1 M n-Bu_4NPF_6 as the supporting electrolyte. All experiments were run under argon with a glassy carbon working electrode, Ag/AgCl wire quasi-reference and a Pt wire counter electrode. The potentials were referenced internally to the half potential of the fcH/ fcH^+ redox couple.

Absorption and photoluminescence in solution

Supplementary Table 4: PL data for **NB-1**, **NB-2**, **NB-3** and **En-Per** in toluene

	Abs 0-0 (nm)	PL 0-0 (nm)[a]	Stokes shift (nm)	FWHM (nm)	PLQY raw (%)[a]	PLQY tail fit (%)[b]	τ (ns)[c]	k_r (s^{-1})	k_{nr} (s^{-1})
**NB-1**	445	447	2	11	81	96	5.8	1.7×10^8	6.9×10^6
**NB-2**	451	454	3	11	75	93	6.2	1.5×10^8	1.1×10^7
**NB-3**	440	443	2.5	10	75	100	5.6	1.8×10^8	-
**En-Per**	453.5	453.5	0	44	80	100	4.1	2.4×10^8	-

[a]exc 370 nm for **NB**, 420 nm for **En-Per**; [b]Corrected for self-reabsorption; [c] exc 360 nm

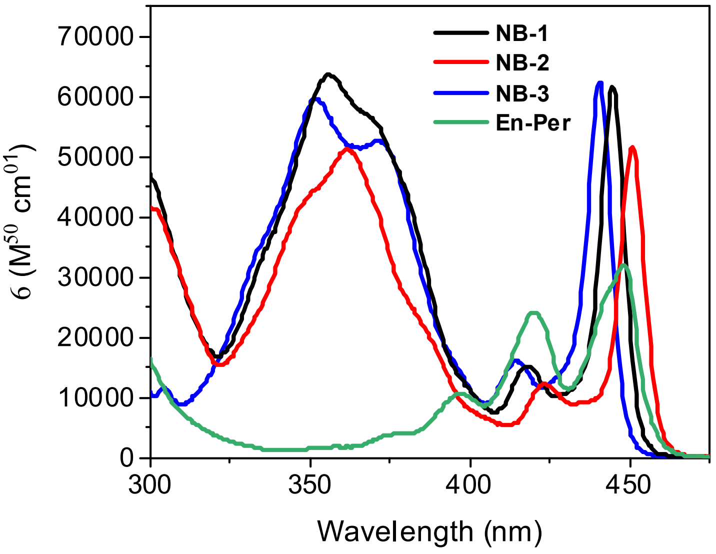

Supplementary Fig. 59: Extinction coefficient spectra of **NB-1**, **NB-2**, **NB-3** and **En-Per** in toluene. For reference, the molar extinction coefficient for the perylene 0–0 band has been reported at 38,500 M^{-1} cm^{-1}.[28]

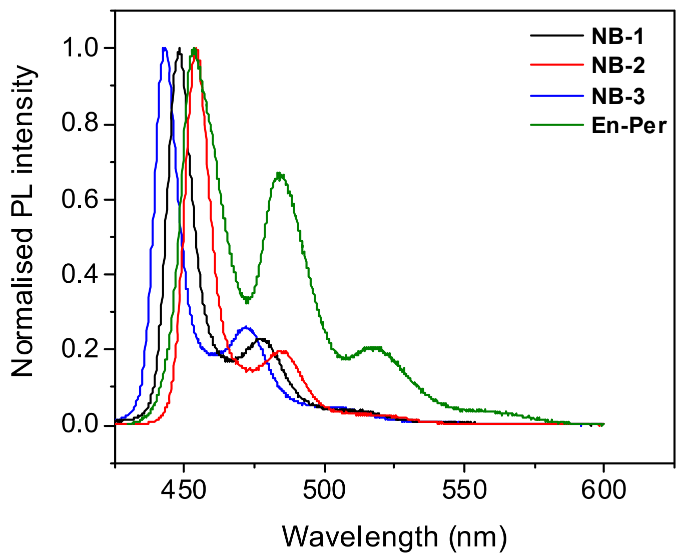

Supplementary Fig. 60: PL spectra of **NB-1**, **NB-2**, **NB-3** (exc 370 nm) and **En-Per** (420 nm) in toluene.

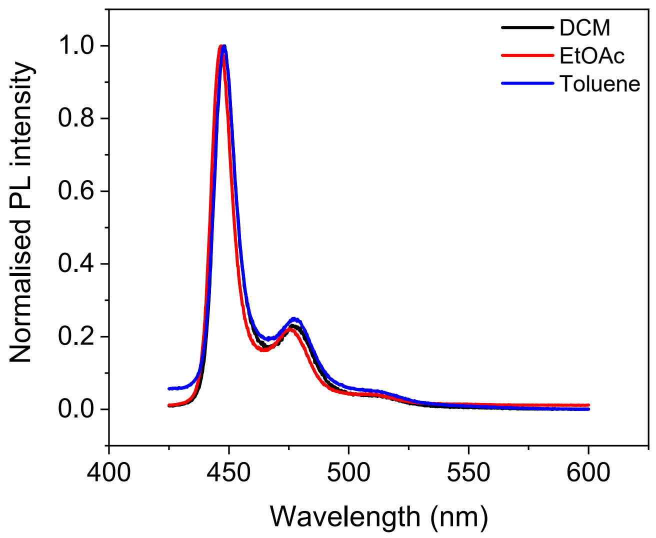

Supplementary Fig. 61: PL spectra (exc 370 nm) of **NB-1** in different solvents.

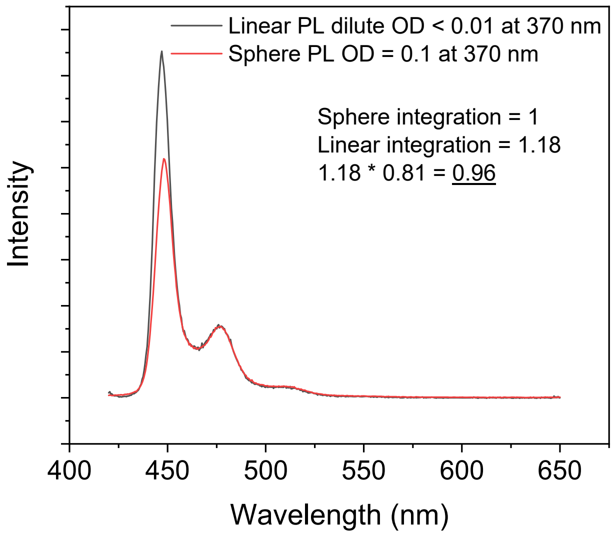

Supplementary Fig. 62: Example tail-fit for determining the PLQY of **NB-1** corrected for self-reabsorption.

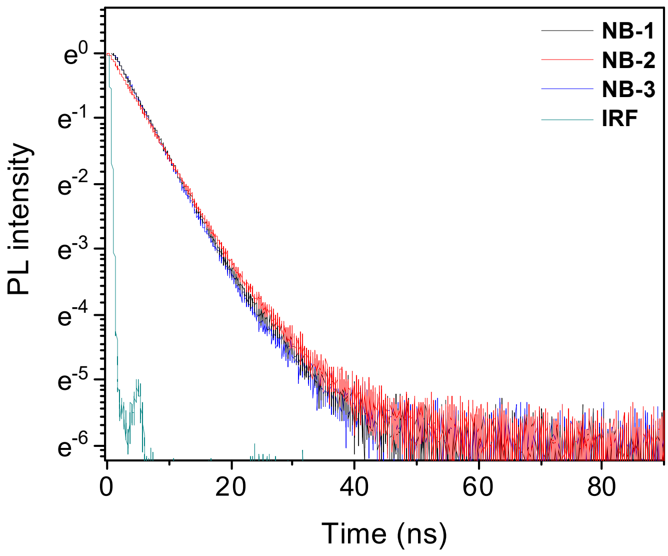

Supplementary Fig. 63: PL decays (exc 360 nm) of **NB-1**, **NB-2** and **NB-3** in toluene, measured using TCSPC.

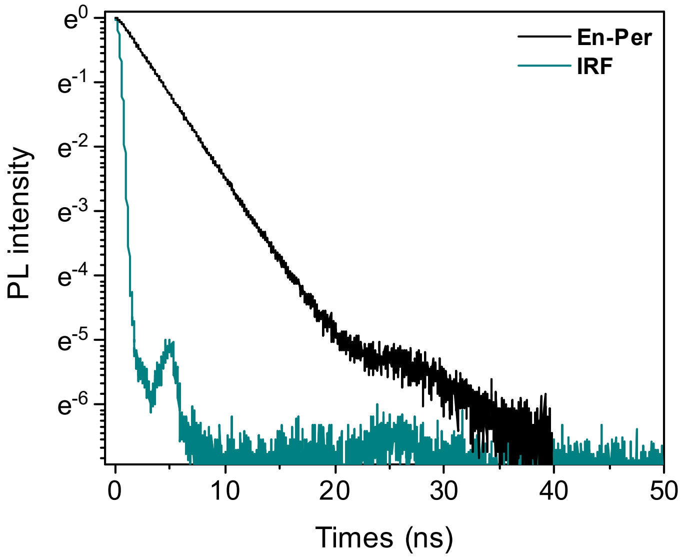

Supplementary Fig. 64: PL decay (exc 360 nm) of **En-Per** in toluene, measured using TCSPC.

Electrochemistry

The oxidation processes for **NB-1**, **NB-2** and **En-Per** are electrochemically reversible (based on the equal intensity of the corresponding anodic and cathodic peaks). A clearly reversible oxidation could not be observed for **NB-3**, potentially due to its very low solubility (which also adds greater error to the HOMO estimation). Therefore, for consistency the oxidation potential for all emitters was determined from the oxidation onset (E_{onset}^{ox}) rather than the half wave potentials. Convincing reduction processed could not be observed for all emitters despite scanning to potentials where the reduction was expected for the **NB** molecules.[29] Therefore, for consistency across the series the LUMO energy was extrapolated from the optical gap.

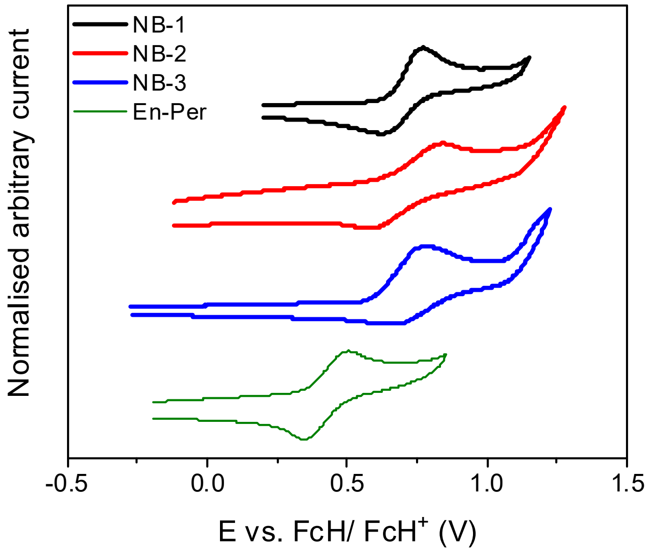

Supplementary Fig. 65: Cyclic voltammograms.

Supplementary Table 5: Electrochemical data.

**Compound**	E_{onset}^{ox} [E_{pa}] / V[a]	**HOMO / eV**[b]	**LUMO / eV**[c]	E_g^{opt} / eV[d]
**NB-1**	0.65 [0.77]	−5.75	−3.01	2.74
**NB-2**	0.64 [0.84]	−5.74	−3.04	2.70
**NB-3**	0.59 [0.79]	−5.69	−2.92	2.77
**En-Per**	0.35 [0.50]	−5.45	−2.74	2.71

[a] Referenced to $E_{1/2}$ of the fcH/ fcH$^+$ redox couple; [b]HOMO level calculated from CV potentials using the HOMO of ferrocene (–5.10 eV) as the standard, HOMO = −5.10 + ($-E_{onset}^{ox}$); [c]LUMO = HOMO + E_g^{opt}; [d]Onset of the UV-Vis spectrum in toluene

Photostability in solution

Samples were prepared in degassed toluene and irradiated 400 nm upconverted pump laser. PL intensity was monitored as a function of time with an iCCD camera. Below a fluence of *ca.* 4000 μW/cm^2 it was not possible to observe any appreciable decrease in PL intensity over hours of exposure.

To observe degradation a high fluence of 8000 μW/cm^2 was necessary. Both **NB-1** and **NB-3** are highly photostable, retaining over 90% PL intensity over an experiment time of 500 seconds.

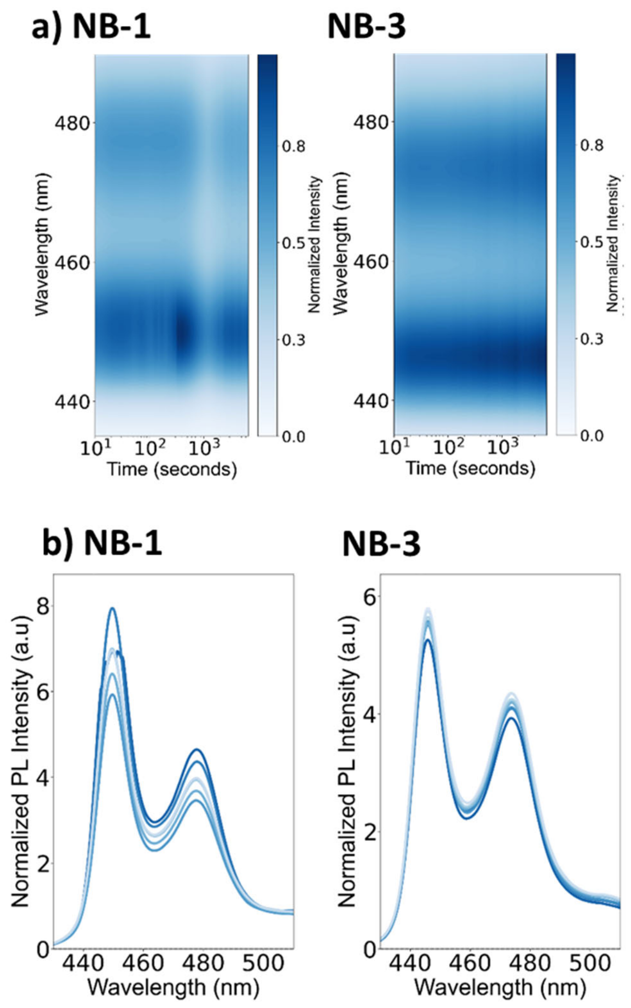

Supplementary Fig. 66: a), b) Plots of PL intensity over time for **NB-1** and **NB-3** in photostability experiments.

Data in Zeonex

The samples were prepared via drop-casting onto a sapphire substrate from a mixture of the compound at 1 wt.% to zeonex in toluene. The films were then dried in a vacuum oven at room temperature for 1 hour to remove any trace solvent.

The time-resolved photoluminescence spectra were recorded using nanosecond gated luminescence and lifetime measurement setup (from 400 ps to 1 s). The sample was loaded into a Janis Research VNF-100 cryostat which was placed under vacuum and kept at room temperature. The excitation pulses were provided by an Ekspla Nd:YAG laser at the third harmonic of 355 nm and the emission was collected after passing through a spectrograph on a Stanford Computer Optics iCCD camera to produce the time-resolved emission spectra.

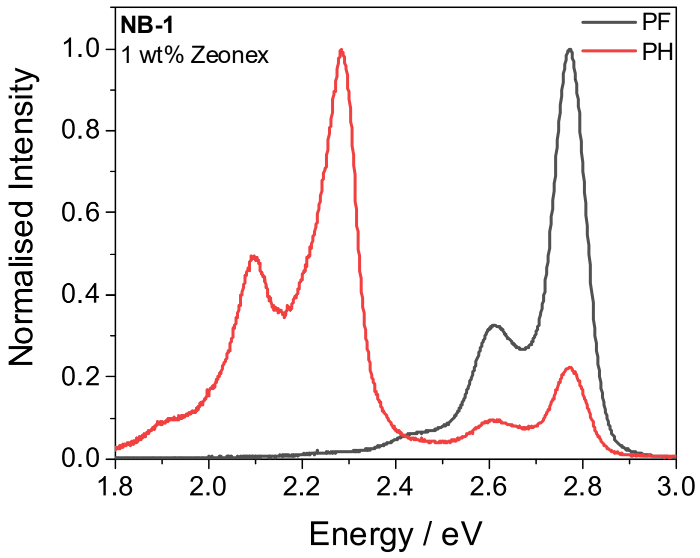

Supplementary Fig. 67: Prompt fluorescence and phosphorescence spectra of **NB-1** in 1 wt.% Zeonex film. ΔE_{ST} *ca.* 0.5 eV. The higher energy emission in the PH spectra is delayed fluorescence, hence the very similar onset to the PF spectra.

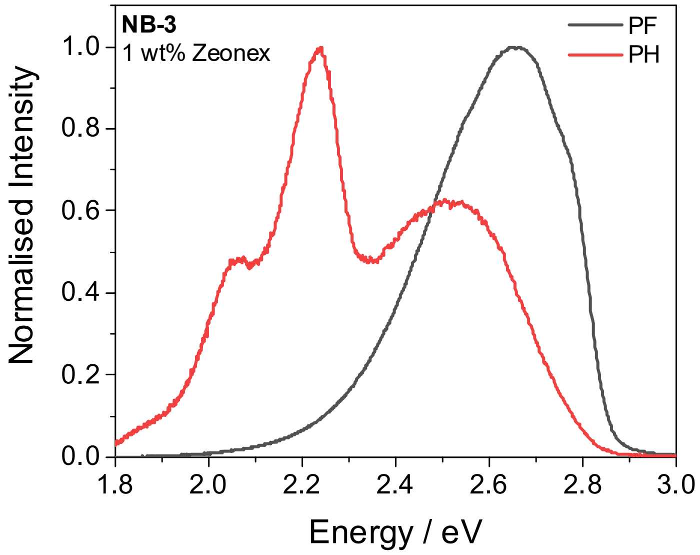

Supplementary Fig. 68: Prompt fluorescence and phosphorescence spectra of **NB-3** in 1 wt.% Zeonex film. ΔE_{ST} *ca.* 0.5 eV. The higher energy emission in the PH spectra is delayed fluorescence, hence the very similar onset to the PF spectra.

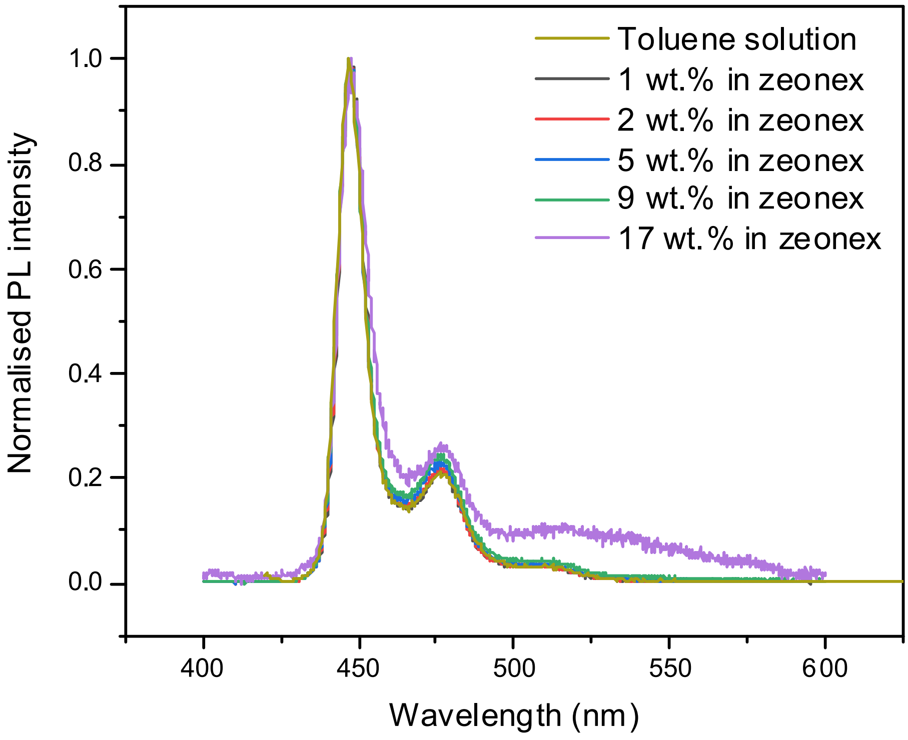

Supplementary Fig. 69: Steady state PL spectra for a concentration series of **NB-1** in zeonex.

Data in pristine samples

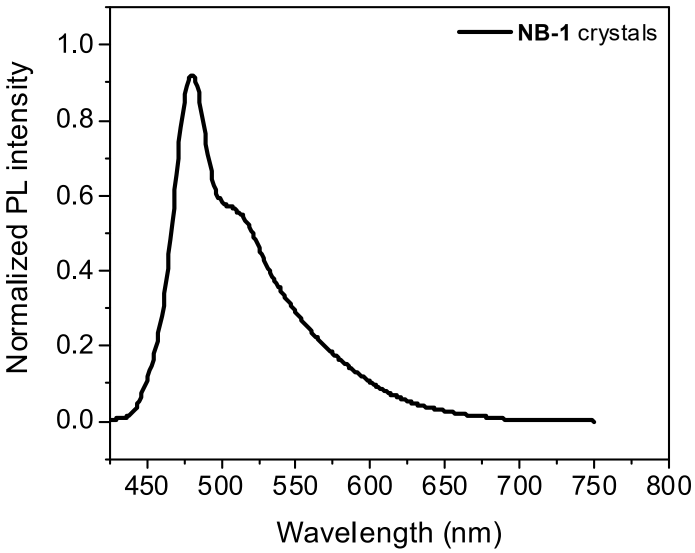

Supplementary Fig. 70: Steady-state PL spectrum for single crystals of **NB-1**.

Supplementary Table 6: Single crystal PL data **NB-1**.

PLQY (%)	$\tau_{average}$ (ns)	τ_1 (ns)	A_1	τ_2 (ns)	A_2	τ_3 (ns)	A_3
6.2	5.6	2.4	0.6	6.3	0.3	16.2	0.02

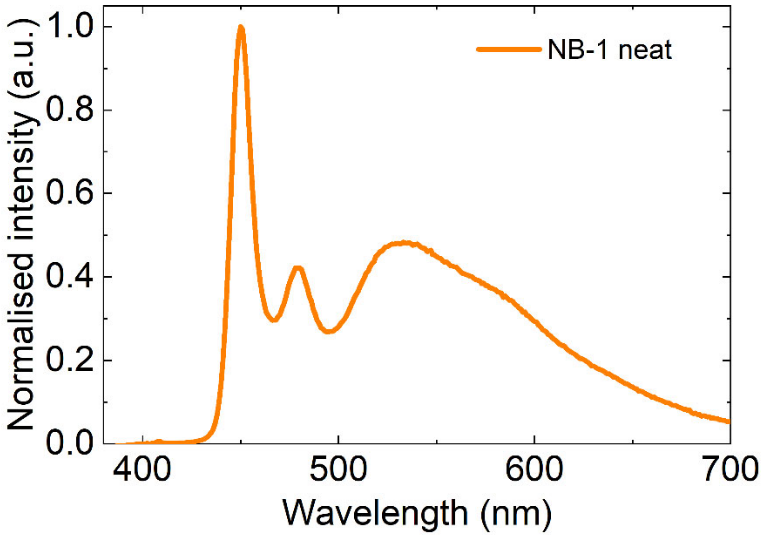

Supplementary Fig. 71: Steady-state PL spectrum for an evaporated film of **NB-1**.

Data in wide-gap hosts

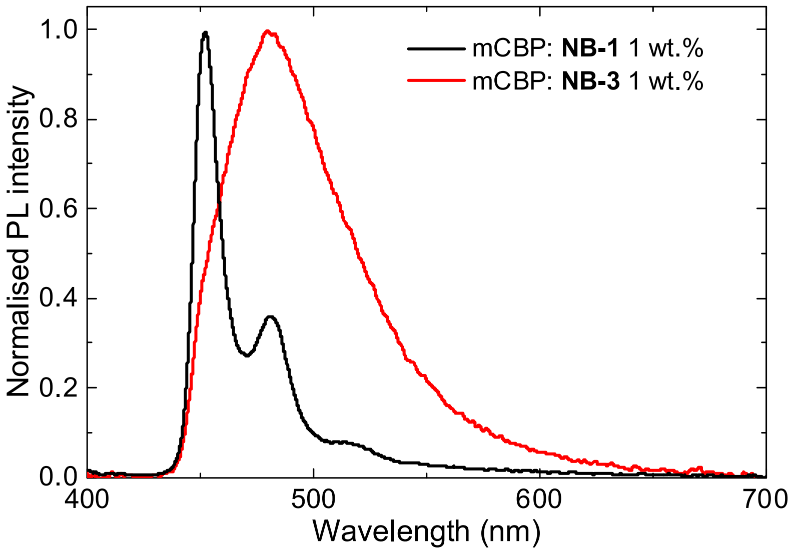

Supplementary Fig. 72: Steady-state PL spectra for **NB-1** and **NB-3** doped into mCBP at 1 wt.% (exc = 330 nm).

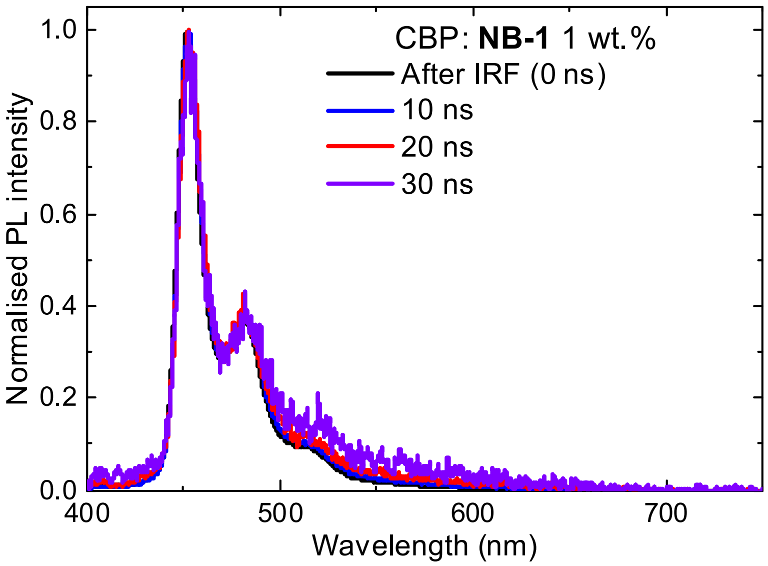

Supplementary Fig. 73: Time-resolved PL spectra for **NB-1** doped into CBP at 1 wt.% (exc = 330 nm).

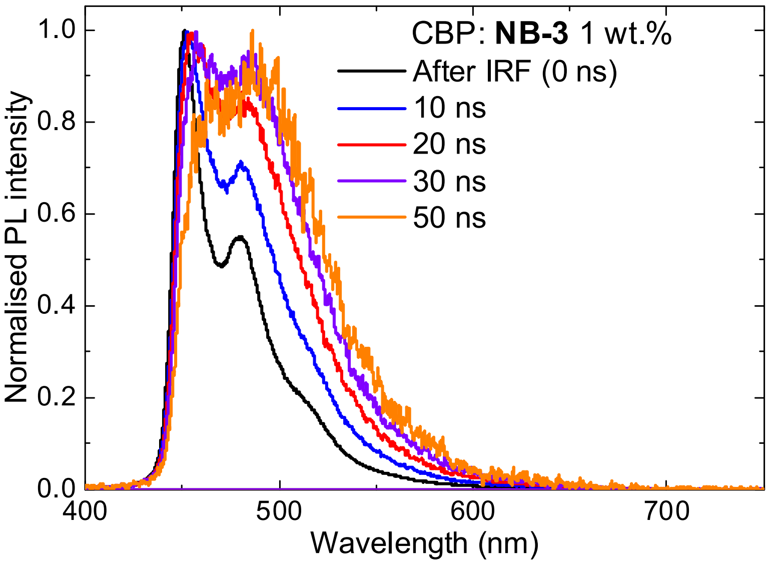

Supplementary Fig. 74: Time-resolved PL spectra for **NB-3** doped into CBP at 1 wt.% (exc = 330 nm).

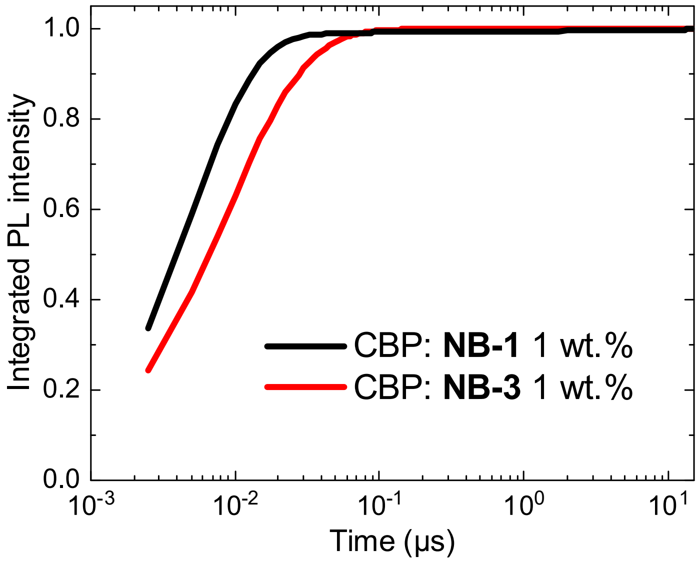

Supplementary Fig. 75: Plots of integrated PL intensity over time for **NB-1** and **NB-3** doped into CBP at 1 wt.% (exc = 330 nm). The slower decay of **NB-3** is ascribed to energy transfer to aggregates, in-line with Fig. S5.10.

Data in TADF and HF systems

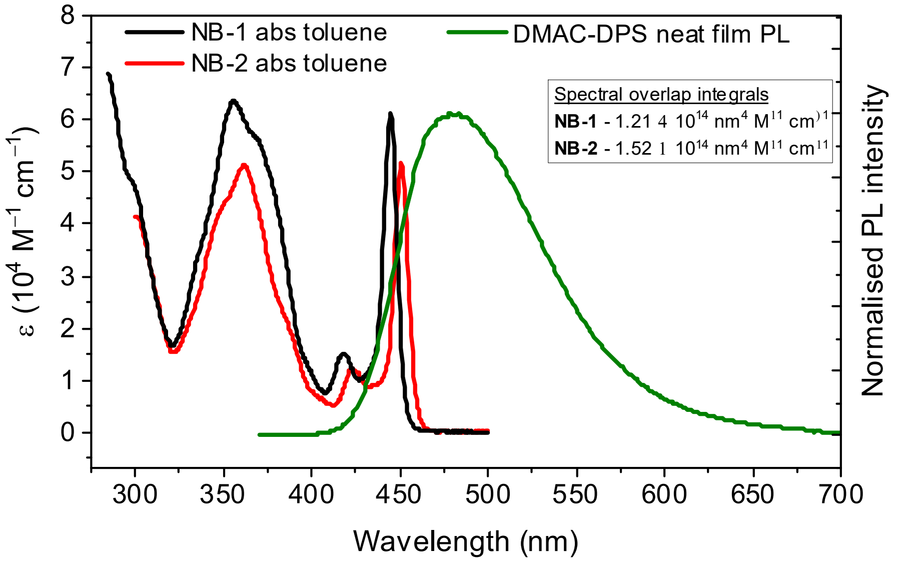

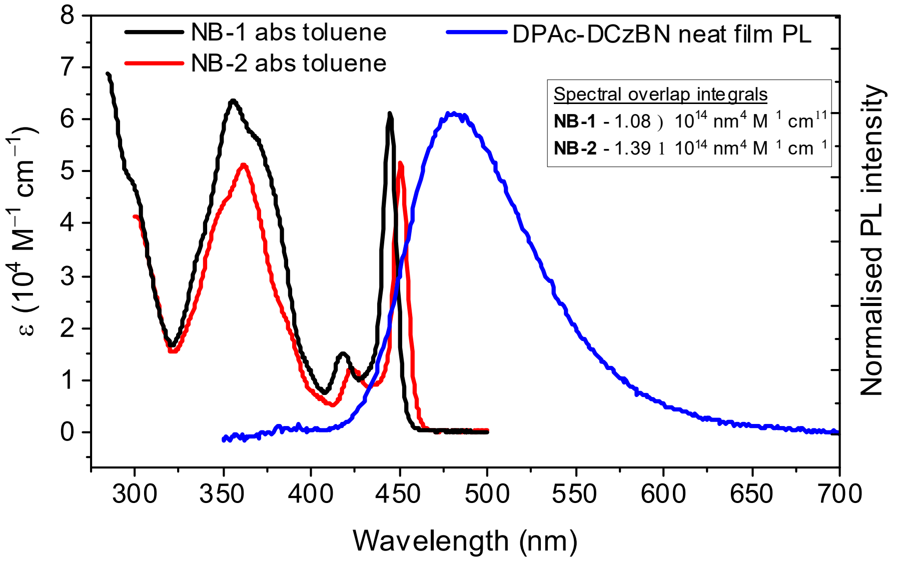

Host	Emitter	Normalised overlap integral
**DMAC-DPS**	**NB-1**	1.12
	**NB-2**	1.41
**DPAc-DCzBN**	**NB-1**	1
	**NB-2**	1.29

Supplementary Fig. 76: PL spectra of the neat film hosts (exc = 330 nm) plotted alongside the solution extinction coefficient spectra of **NB-1** and **NB-2** to show spectral overlap for energy transfer.

1) Exciton decay kinetics

To study exciton decay kinetics in this hyperfluorescence system, transient PL measurements were conducted with 330 nm laser excitation. Supplementary Fig. 77 shows the transient PL data for the **NB-1** and **NB-3** doped films and the DMAC-DPS neat film. The radiative decay rate constants of prompt (k_p) and delayed (k_d) fluorescence for the films can be calculated by,

$$k_{\mathrm{p}} = \frac{\Phi_{\mathrm{p}}}{\tau_{\mathrm{p}}} \tag{S5-1}$$

$$k_{\mathrm{d}} = \frac{\Phi_{\mathrm{d}}}{\tau_{\mathrm{d}}} \tag{S5-2}$$

where Φ_p and Φ_{d} are the PLQEs of prompt and delayed emission, and τ_{p} and τ_{d} are the decay lifetime of prompt and delayed emission. The total PLQE (Φ_{total}) is expressed by the sum of Φ_{p} and Φ_{d}, expressed by the following equations,

$$\Phi_{\mathrm{total}} = \Phi_{\mathrm{p}} + \Phi_{\mathrm{d}} \tag{S5-3}$$

$$\Phi_{\mathrm{p}} = r_1 \Phi_{\mathrm{total}} \tag{S5-4}$$

$$\Phi_{\mathrm{d}} = r_2 \Phi_{\mathrm{total}} \tag{S5-5}$$

where r_1 and r_2 are the intensity ratio of prompt and delayed components, defined by

$$r_1 = \frac{A_1 \tau_{\mathrm{p}}}{A_1 \tau_{\mathrm{p}} + A_2 \tau_{\mathrm{d}}} \tag{S5-6}$$

$$r_2 = \frac{A_2 \tau_{\mathrm{d}}}{A_1 \tau_{\mathrm{p}} + A_2 \tau_{\mathrm{d}}} \tag{S5-7}$$

where A_1 and A_2 are the fitting parameters in the double exponential equation ($A_1 e^{-t/\tau_{\mathrm{p}}} + A_2 e^{-t/\tau_{\mathrm{d}}}$). Supplementary Figs. 77 and 78 show the biexponential fittings for the transient PL profiles of the films. Based on these results, the decay time and rate constants for the films are calculated,[30] as summarised in Supplementary Tables 7 and 8. Similar analysis was also carried out for the DPAc-DCzBN:**NB-2** system (Supplementary Figs. 79 and 80, Supplementary Tables 9 and 10)

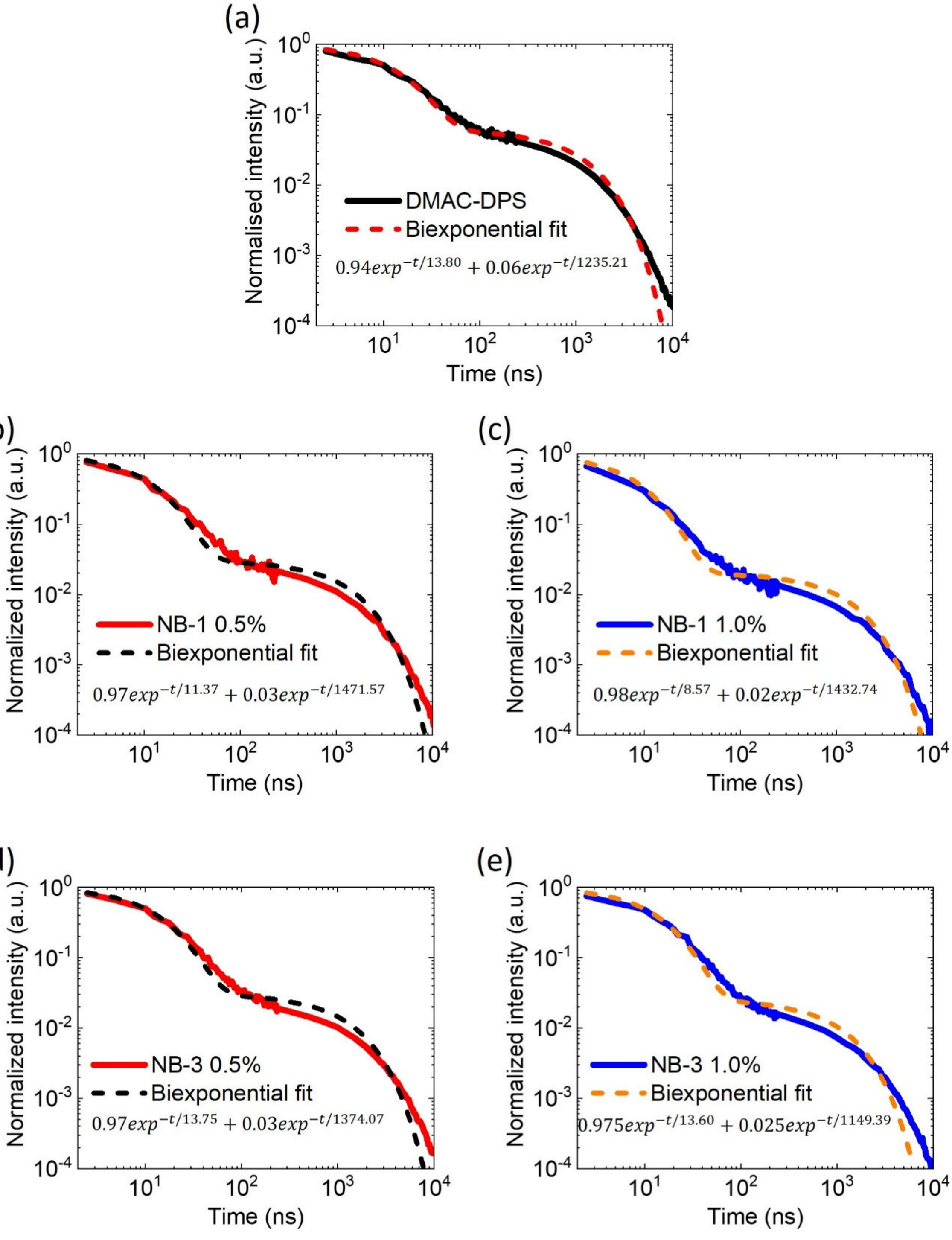

Supplementary Fig. 77: Transient PL profiles of DMAC-DPS-based films. (a) DMAC-DPS. (b),(c) **NB-1** 0.5% and 1.0%. (d),(e) **NB-3** 0.5% and 1.0%.

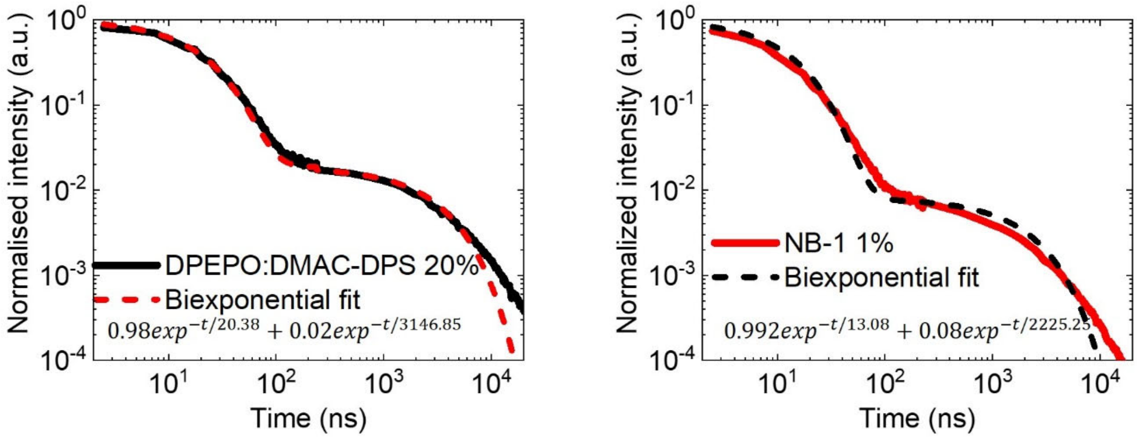

Supplementary Fig. 78: Transient PL profiles of DPEPO:DMAC-DPS 20% and DPEPO:DMAC-DPS 20%: NB-1 1% films.

Supplementary Table 7: The summary of PLQEs (total, prompt, and delayed), decay time, and decay rate for DMAC-DPS neat and **NB-1** and **NB-3** doped in DMAC-DPS.

Host	DMAC-DPS				
Dopant	**Non-doped**	**NB-1**		**NB-3**	
Doping (%)		0.5	1	0.5	1
Φ_{total}	0.96	0.75	0.63	0.43	0.35
r_1	0.15	0.15	0.23	0.24	0.32
r_2	0.85	0.60	0.77	0.76	0.68
Φ_p	0.14	0.20	0.14	0.11	0.11
Φ_d	0.82	0.55	0.49	0.32	0.24
τ_p (10^{-9} s)	13.80	11.37	8.57	13.75	13.60
τ_d (10^{-6} s)	1.24	1.47	1.43	1.37	1.15
k_p (10^7 /s)	1.04	1.32	1.67	0.76	0.81
k_d (10^5 /s)	6.61	4.08	3.40	2.36	2.08

Supplementary Table 8: The summary of PLQEs (total, prompt, and delayed), decay time, and decay rate for DPEPO:DMAC-DPS 20% neat and **NB-1** doped in DMAC-DPS.

Host	DPEPO:DMAC-DPS 20%	
Dopant	**Non-doped**	**NB-1**
Doping (%)		1
Φ_{total}	1.00	0.83
r_1	0.24	0.42
r_2	0.76	0.58
Φ_p	0.24	0.35
Φ_d	0.76	0.48
τ_p (10^{-9} s)	20.38	13.08
τ_d (10^{-6} s)	3.15	2.23
k_p (10^7 /s)	1.18	2.68
k_d (10^5 /s)	2.4	2.16

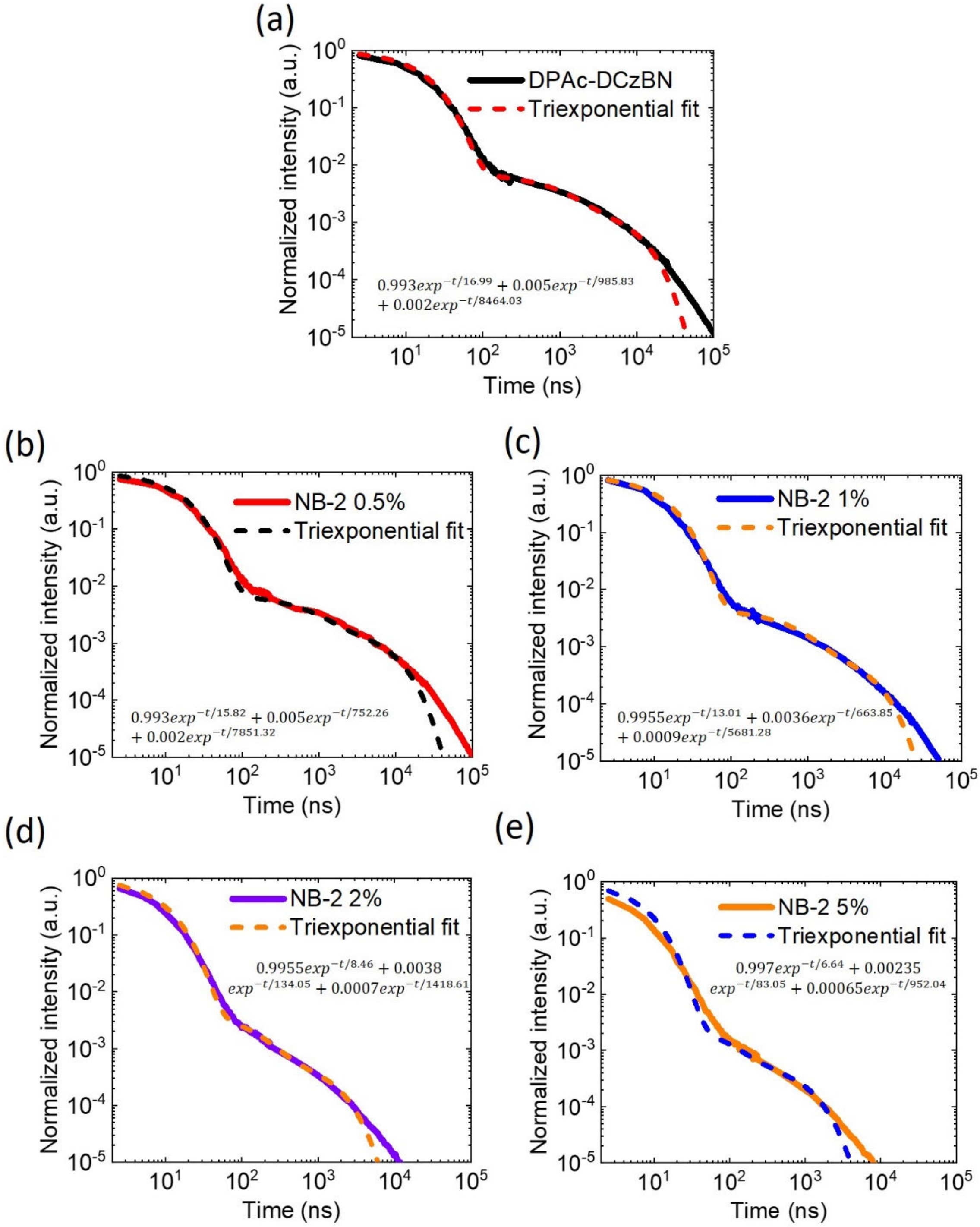

Supplementary Fig. 79: Transient PL profiles of the DPAc-DCzBN and DPAc-DCzBN: **NB-2** system. (a) DPAc-DCzBN. (b),(c) **NB-2** 0.5% and 1.0%. (d),(e) **NB-2** 0.5% and 1.0%.

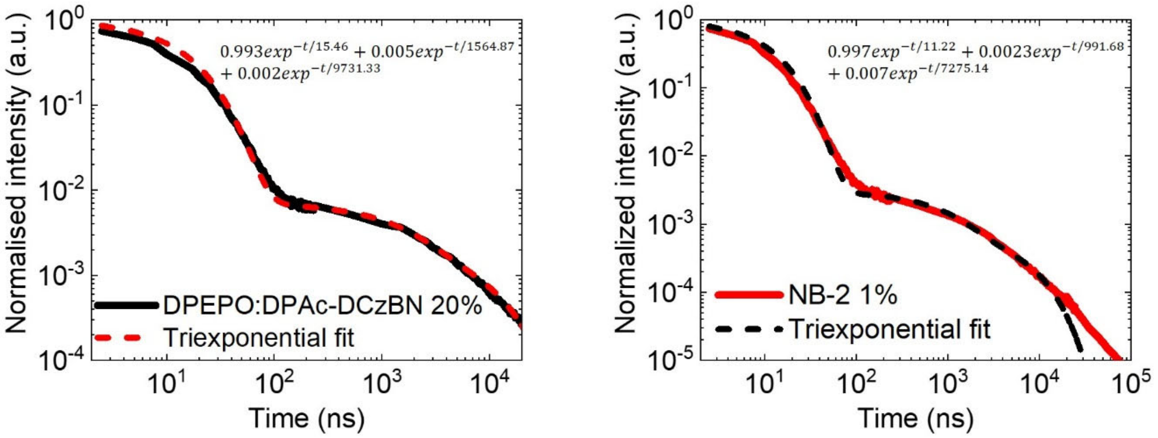

Supplementary Fig. 80: Transient PL profiles of DPEPO:DPAc-DCzBN 20% and DPEPO:DPAc-DCzBN 20%: **NB-2** 1% films.

Supplementary Table 9: The summary of PLQEs (total, prompt, and delayed), decay time, and decay rate for DPAc-DCzBN neat and **NB-2** doped in DPAc-DCzBN.

Host	DPAc-DCzBN				
Dopant	**Non-doped**	**NB-2**			
Doping (%)		0.5	1	2.5	5.0
Φ_{total}	0.91	0.87	0.69	0.45	0.36
r_1	0.44	0.45	0.63	0.85	0.89
r_2	0.56	0.55	0.37	0.15	0.11
Φ_p	0.40	0.39	0.44	0.38	0.32
Φ_d	0.51	0.48	0.25	0.07	0.04
τ_p (10^{-9} s)	16.99	15.82	13.01	8.46	6.64
τ_d (10^{-6} s)	6.78	6.48	4.08	0.98	0.74
k_p (10^7/s)	2.33	2.46	3.36	5.21	4.8
k_d (10^5/s)	0.76	0.74	0.62	0.69	0.53

Supplementary Table 10: The summary of PLQEs (total, prompt, and delayed), decay time, and decay rate for DPEPO:DPAc-DCzBN 20% neat and **NB-2** doped in DPAc-DCzBN.

Host	DPEPO:DPAc-DCzBN 20%	
Dopant	**Non-doped**	**NB-2**
Doping (%)		1
Φ_{total}	0.96	0.79
r_1	0.36	0.60
r_2	0.64	0.40
Φ_p	0.35	0.48
Φ_d	0.61	0.31
τ_p (10^{-9} s)	15.46	11.22
τ_d (10^{-6} s)	7.39	5.33
k_p (10^7/s)	2.24	4.24
k_d (10^5/s)	0.83	0.59

2) Exciton dynamics of TADF hosts

Assuming that the T_1-to-S_0 radiative and nonradiative transition in DMAC-DPS ($k_{\mathrm{r,T}}$ and $k_{\mathrm{nr,T}}$) is negligible, the decay rates and PLQEs of the prompt and delayed emission for DMAC-DPS ($k_{\mathrm{p,D}}$, $\Phi_{\mathrm{p,D}}$, $k_{\mathrm{d,D}}$, $\Phi_{\mathrm{d,D}}$) can be approximated as

$$k_{\mathrm{p,D}} = k_{\mathrm{r,S}} + k_{\mathrm{nr,S}} + k_{\mathrm{ISC}} \quad \text{(S5-8)}$$

$$\Phi_{\mathrm{p,D}} = \frac{k_{\mathrm{r,S}}}{k_{\mathrm{r,S}}+k_{\mathrm{nr,S}}+k_{\mathrm{ISC}}} = \frac{k_{\mathrm{r,S}}}{k_{\mathrm{p,D}}} \quad \text{(S5-9)}$$

$$k_{\mathrm{d,D}} = \left(1 - \frac{k_{\mathrm{r,S}}}{k_{\mathrm{r,S}}+k_{\mathrm{nr,S}}+k_{\mathrm{ISC}}}\right) k_{\mathrm{RISC}} \quad \text{(S5-10)}$$

$$\Phi_{\mathrm{d,D}} = \sum_{k=1}^{\infty}(\Phi_{\mathrm{ISC}}\Phi_{\mathrm{RISC}})^k \Phi_{\mathrm{p,D}} = \frac{\Phi_{\mathrm{ISC}}\Phi_{\mathrm{RISC}}}{1-\Phi_{\mathrm{ISC}}\Phi_{\mathrm{RISC}}}\Phi_{\mathrm{p,D}} \quad \text{(S5-11)}$$

where $k_{\mathrm{r,S}}$ and $k_{\mathrm{nr,S}}$ are the radiative and nonradiative decay rates of the singlet state, k_{ISC} and k_{RISC} are the rate constants of ISC and RISC, and Φ_{ISC} and Φ_{RISC} are the efficiencies of ISC and RISC, respectively. From the above equations, k_{ISC} and k_{RISC} are described by the following equations,

$$k_{\mathrm{ISC}} = \frac{\Phi_{\mathrm{d,D}}}{\Phi_{\mathrm{p,D}}+\Phi_{\mathrm{d,D}}} k_{\mathrm{p,D}} \quad \text{(S5-12)}$$

$$k_{\mathrm{RISC}} = \frac{k_{\mathrm{d,D}}\Phi_{\mathrm{RISC}}}{1-\Phi_{\mathrm{ISC}}\Phi_{\mathrm{RISC}}} = \frac{k_{\mathrm{p,D}}k_{\mathrm{d,D}}}{k_{\mathrm{ISC}}}\frac{\Phi_{\mathrm{d,D}}}{\Phi_{\mathrm{p,D}}} \quad \text{(S5-13)}$$

Accordingly, k_{ISC} and k_{RISC} are obtained as 8.82 × 10^6 s^{-1} and 4.44 × 10^6 s^{-1} , respectively. From the same method, for DPAc-DCzBN, k_{ISC} and k_{RISC} are calculated as 1.32 × 10^7 s^{-1} and 1.74 × 10^5 s^{-1}, respectively. Also, for DPEPO:DMAC-DPS 20 wt% k_{ISC} and k_{RISC} are calculated as 8.85 × 10^6 s^{-1} and 1.01 × 10^6 s^{-1}, respectively, and for DPEPO:DPAc-DCzBN 20 wt%, k_{ISC} and k_{RISC} are calculated as 1.43 × 10^7 s^{-1} and 2.31 × 10^5 s^{-1}.

3) Förster resonance energy transfer (FRET)[31]

The FRET between DMAC-DPS and the emitters are explored. The fluorescent emission from the DMAC-DPS hosted films consists of the emitter emission by excitons transferred from DMAC-DPS via FRET in addition to DMAC-DPS emission by the remaining excitons not transferred to the emitter molecules. To understand FRET between DMAC-DPS and the emitters, Forster radius (R_0) was calculated by,

$$R_0^6 = \frac{9000 \ln 10 \eta_D \kappa^2}{128\pi^5 N_A n^4} \int \lambda^4 F_{\mathrm{D}}(\lambda) \epsilon_{\mathrm{A}}(\lambda) d\lambda \qquad \text{(S5-14)}$$

where N_A is Avogadro's number, n is the refractive index, η_{D} is the PLQE of the donor, κ is the dipole orientation factor, λ is the wavelength, F_D is the PL spectrum of the donor normalised to unity, and ϵ_{A} is the molar decadic absorption coefficient of the acceptor. n is set as 1.7 and κ in a randomly orientated amorphous film is $0.845\sqrt{2/3}$, which is used for the calculation of R_0.[32] The values of R_0 are 2.73 nm for **NB-3** and 2.88 nm for **NB-1**, respectively.

Assuming that the fluorescent emission of the emitter-doped films originates from the FRET of excitons initially formed in DMAC-DPS, the characteristic FRET efficiency and rate (E_{FRET} and k_{FRET}) can be described as

$$E_{\mathrm{FRET}} = \frac{k_{\mathrm{FRET}}}{k_{\mathrm{p,D}} + k_{\mathrm{FRET}}} = \frac{1/\Phi_{\mathrm{total}} \int P_{\mathrm{A}}(\lambda) d\lambda}{\int P_{\mathrm{D}}(\lambda) d\lambda + 1/\Phi_{\mathrm{total}} \int P_{\mathrm{A}}(\lambda) d\lambda} \qquad \text{(S5-15)}$$

$$k_{\mathrm{FRET}} = \frac{E_{\mathrm{FRET}} k_{\mathrm{p,D}}}{1 - E_{\mathrm{FRET}}} = \frac{1}{\tau_{\mathrm{D}}} \left(\frac{R_0}{R_{\mathrm{avr}}} \right)^6 \qquad \text{(S5-16)}$$

where P_D and P_A are the decomposed donor and acceptor emission spectrum, Φ_{total} is the total PLQE of the films, τ_{D} is the radiative decay lifetime of DMAC-DPS, and R_{avr} is the average intermolecular distance between donor and acceptor molecules. P_D and P_A are obtained by subtracting a linearly-scaled donor-only spectrum from the total PL spectrum (Supplementary Fig. 81-84). E_{FRET} for **NB-3** is 0.58 for 0.5% and 0.93 for 1.0%, respectively. In contrast, E_{FRET} for **NB-1** is 0.31 for 0.5% and 0.64 for 1.0%, respectively, which is lower than that for **NB-3**. Also, k_{FRET} and R_{avr} can be calculated from Equation S5-16, as shown in Supplementary Table 11. These energy transfer parameters for **NB-2** in DPAc-DCzBN are obtained by the same equations above and shown in Supplementary Table 12.

4) Aggregation and Dexter energy transfer (DET)

Next, the energy loss mechanisms are examined. Dexter triplet transfer does not effect any loss in prompt emission as triplets are only present on delayed timescales in optically pumped experiments. Therefore, losses in prompt emission (e.g. aggregation-caused quenching and self-reabsorption) are simply referred to as 'quenching', while it is approximated that any efficiency loss in delayed emission results from Dexter triplet transfer. Based on these assumptions, the efficiency and rate of quenching (E_{quench} and k_{quench}) can be approximated by

$$E_{\mathrm{quench}} = 1 - \Phi_{\mathrm{total}} = \frac{k_{\mathrm{quench}}}{k_{\mathrm{p}} + k_{\mathrm{quench}}} \quad \text{(S5-17)}$$

$$k_{\mathrm{quench}} = \frac{E_{\mathrm{quench}} k_{\mathrm{p}}}{1 - E_{\mathrm{quench}}} \quad \text{(S5-18)}$$

Also, assuming that $k_{\mathrm{nr,T}}$ is negligible (k_{RISC} and $k_{\mathrm{DET}} \gg k_{\mathrm{nr,T}}$), the efficiency and rate of DET (E_{DET} and k_{DET}) can be expressed by

$$E_{\mathrm{DET}} = 1 - \Phi_{\mathrm{total}} = \frac{k_{\mathrm{DET}}}{k_{\mathrm{RISC}} + k_{\mathrm{DET}}} \quad \text{(S5-19)}$$

$$k_{\mathrm{DET}} = \frac{E_{\mathrm{DET}} k_{\mathrm{RISC}}}{1 - E_{\mathrm{DET}}} \quad \text{(S5-20)}$$

From the above equations, k_{quench} and k_{DET} can be extracted. The rate constants of energy transfer and other important parameters are summarised in Supplementary Table 11. As expected, the k_{quench} and k_{DET} of **NB-1** are much slower than those of **NB-3**, which can lead to higher efficiency in devices. In addition, the k_{quench} and k_{DET} for **NB-2** are also calculated and shown in Supplementary Table 12.

Supplementary Table 11: The summary of E_{FRET}, k_{FRET}, R_0, R_{avr}, k_{quench}, and k_{DET} for the **NB-1** and **NB-3** doped in DMAC-DPS and **NB-1** doped in DPEPO:DMAC-DPS 20%.

Host	DMAC-DPS			
Dopant	**NB-1**		**NB-3**	
Doping (wt%)	0.5	1	0.5	1
E_{FRET}	0.31	0.64	0.58	0.93
k_{FRET} (10^7/s)	0.47	1.84	1.43	13.77
R_0 (nm)	2.88		2.73	
R_{avr} (nm)	3.29	2.62	2.59	1.77
k_{quench} (10^7/s)	0.44	0.98	1.01	1.51
k_{DET} (10^6/s)	1.48	2.61	5.89	8.25

Host	DPEPO:DMAC-DPS 20%
Dopant	**NB-1**
Doping (wt%)	1
E_{FRET}	0.75
k_{FRET} (10^7/s)	3.48
R_0 (nm)	3.10
R_{avr} (nm)	2.59
k_{quench} (10^7/s)	0.54
k_{DET} (10^6/s)	2.08

Supplementary Table 12: The summary of E_{FRET}, k_{FRET}, R_0, R_{avr}, k_{quench}, and k_{DET} for the **NB-2** in DPAc-DCzBN and DPEPO:DPAc-DCzBN 20%.

Host	DPAc-DCzBN			
Dopant	**NB-2**			
Doping (wt%)	0.5	1	2.5	5.0
E_{FRET}	0.16	0.60	0.76	0.95
k_{FRET} (10^7/s)	0.45	3.50	7.55	42.31
R_0 (nm)	2.93			
R_{avr} (nm)	3.85	3.20	2.41	1.81
k_{quench} (10^7/s)	0.37	1.51	5.42	8.58
k_{DET} (10^5/s)	0.26	0.78	2.04	3.09

Host	DPEPO:DPAc-DCzBN 20%
Dopant	**NB-2**
Doping (wt%)	1
E_{FRET}	0.51
k_{FRET} (10^7/s)	2.29
R_0 (nm)	3.30
R_{avr} (nm)	3.29
k_{quench} (10^7/s)	1.13
k_{DET} (10^5/s)	0.61

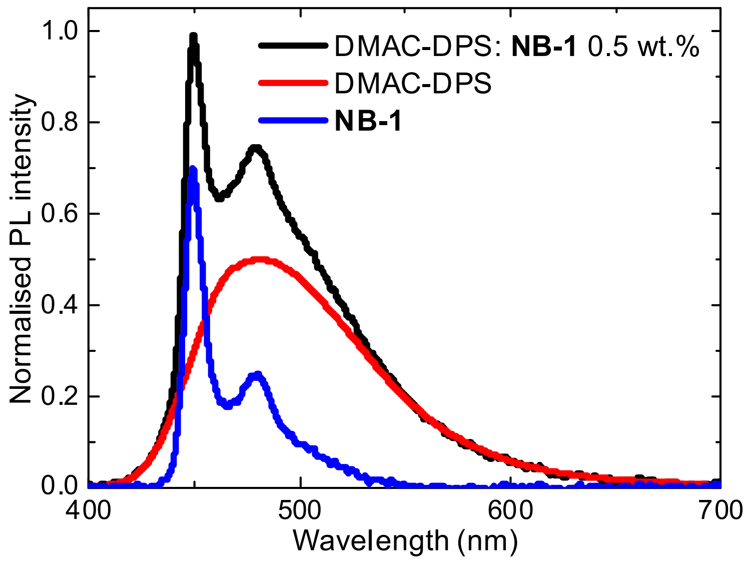

Supplementary Fig. 81: Spectral deconvolution for the PL spectrum of **NB-1** doped into DMAC-DPS at 0.5 wt.% (exc = 330 nm).

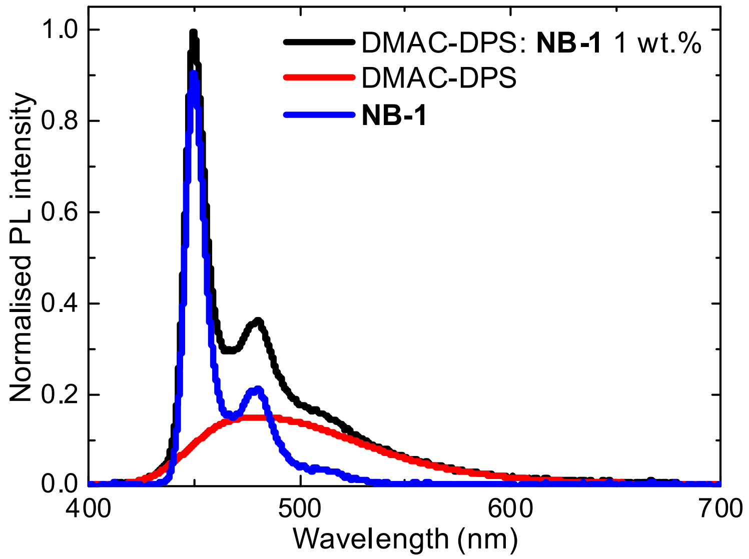

Supplementary Fig. 82: Spectral deconvolution for the PL spectrum of **NB-1** doped into DMAC-DPS at 1 wt.% (exc = 330 nm).

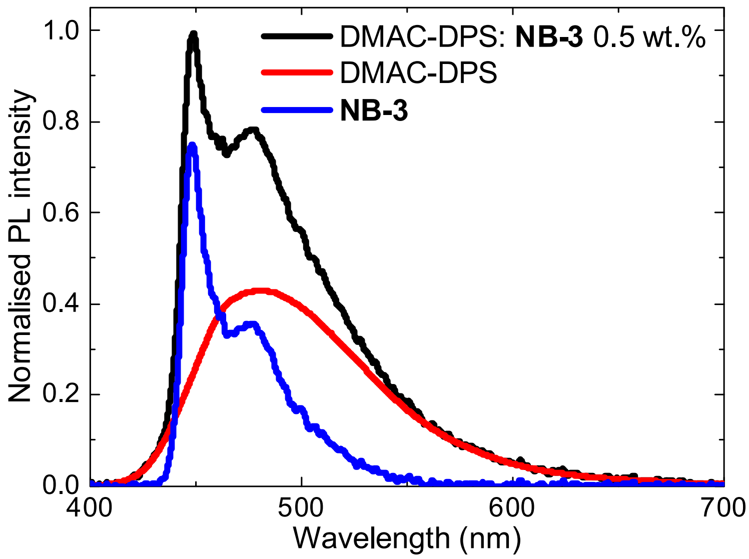

Supplementary Fig. 83: Spectral deconvolution for the PL spectrum of **NB-3** doped into DMAC-DPS at 0.5 wt.% (exc = 330 nm).

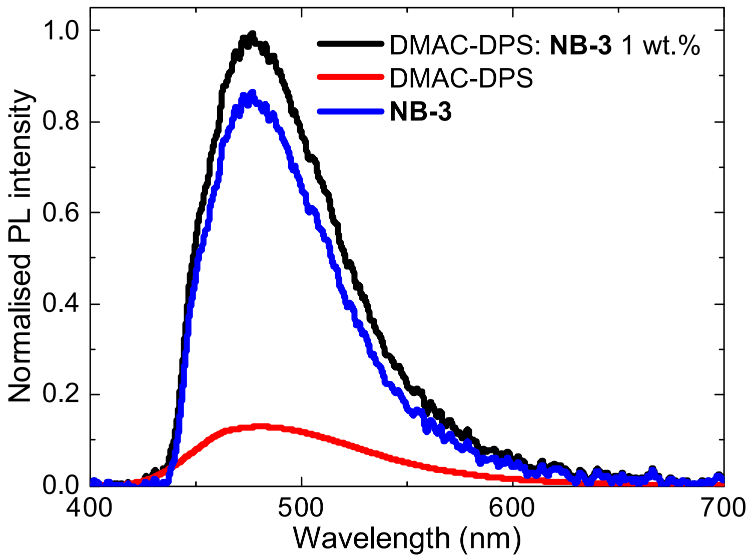

Supplementary Fig. 84: Spectral deconvolution for the PL spectrum of **NB-3** doped into DMAC-DPS at 1 wt.% (exc = 330 nm).

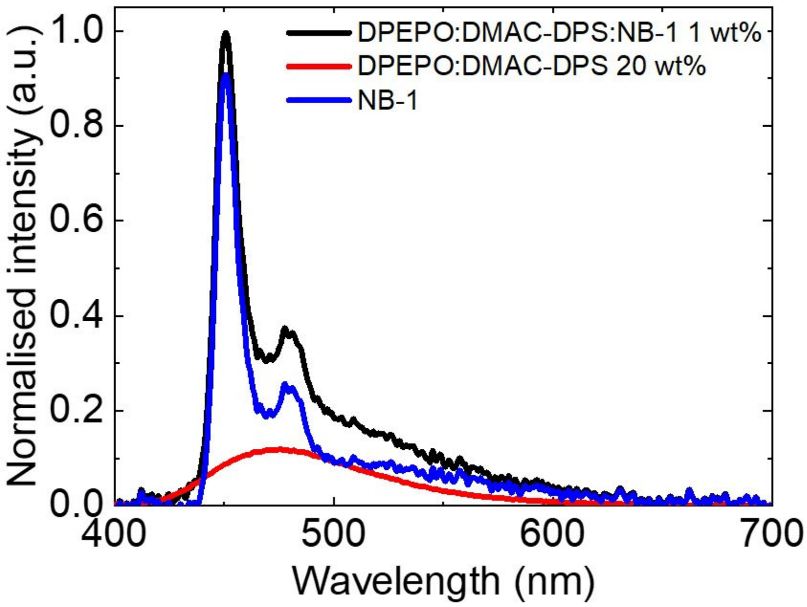

Supplementary Fig. 85: Spectral deconvolution for the PL spectrum of **NB-1** doped into DPEPO:DMAC-DPS 20 wt.% at 1 wt.% (exc = 330 nm).

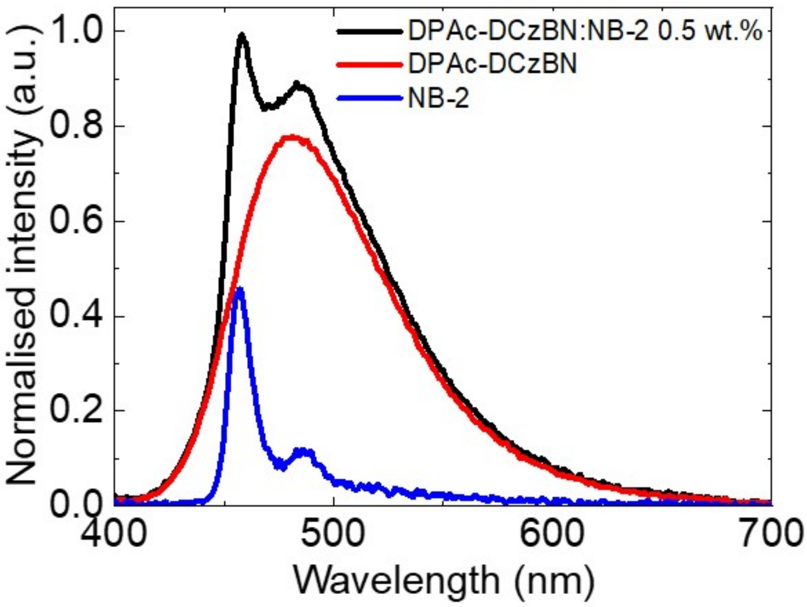

Supplementary Fig. 86: Spectral deconvolution for the PL spectrum of **NB-2** doped into DPAc-DCzBN at 0.5 wt.% (exc = 330 nm).

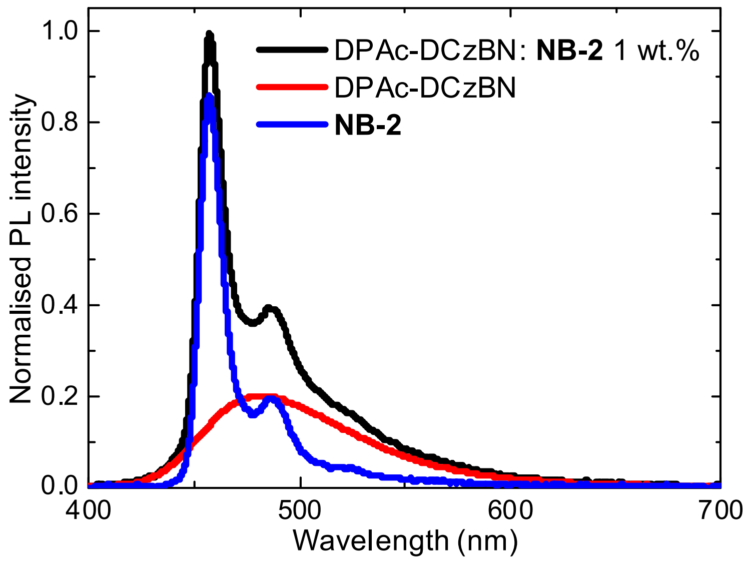

Supplementary Fig. 87: Spectral deconvolution for the PL spectrum of **NB-2** doped into DPAc-DCzBN at 1 wt.% (exc = 330 nm).

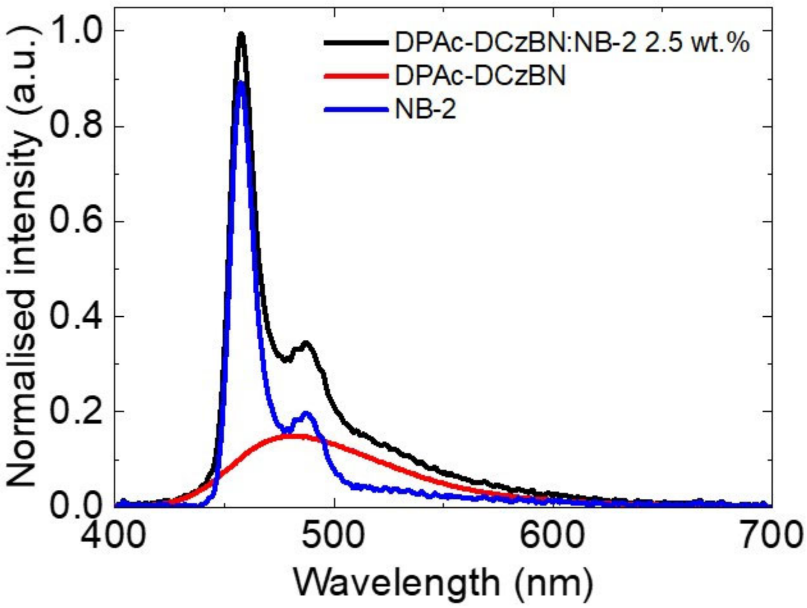

Supplementary Fig. 88: Spectral deconvolution for the PL spectrum of **NB-2** doped into DPAc-DCzBN at 2.5 wt.% (exc = 330 nm).

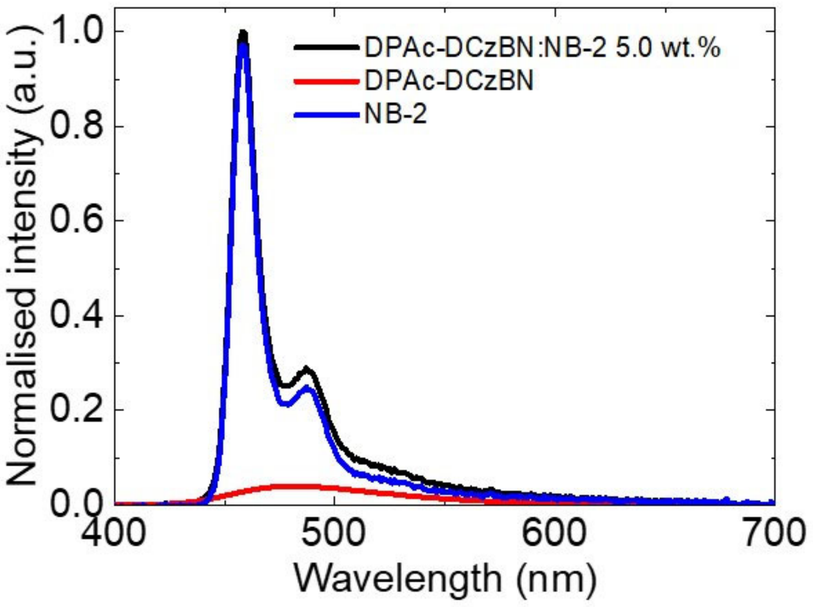

Supplementary Fig. 89: Spectral deconvolution for the PL spectrum of **NB-2** doped into DPAc-DCzBN at 5.0 wt.% (exc = 330 nm).

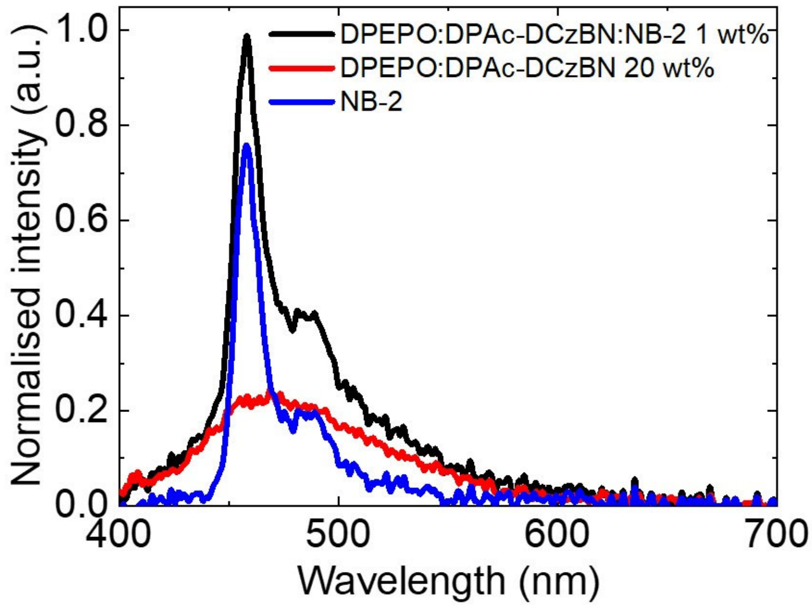

Supplementary Fig. 90: Spectral deconvolution for the PL spectrum of **NB-2** doped into DPEPE:DPAc-DCzBN 20 wt% at 1.0 wt.% (exc = 330 nm).

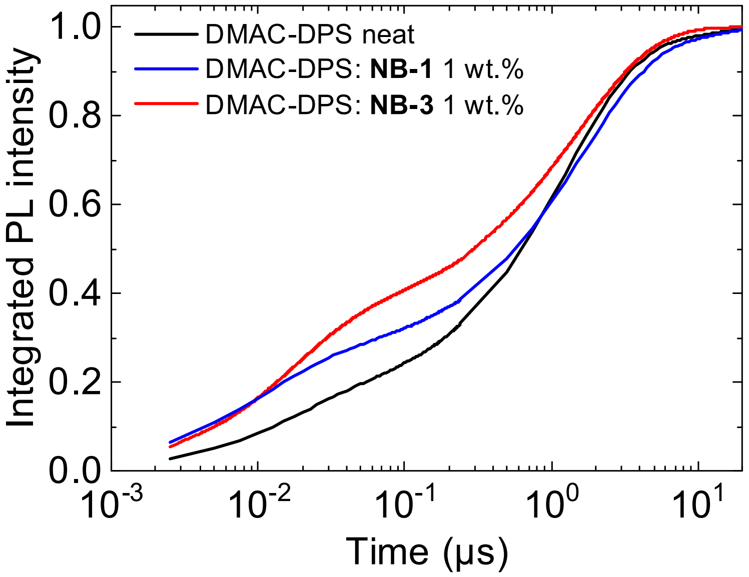

Supplementary Fig. 91: Plots of integrated PL intensity over time for **NB-1** and **NB-3** doped into DMAC-DPS at 1 wt.% and DMAC-DPS (exc = 330 nm). The slower decay of the **NB-1** doped film compared to **NB-3** is in agreement with the higher delayed PLQY of **NB-1** in DMAC-DPS at 1 wt.%.

Supplementary Note 6 - Transient absorption spectroscopy

General

TA was performed on a setup powered using a commercially available Ti:sapphire amplifier (Spectra Physics Solstice Ace). The amplifier operates at 1 kHz and generates 100 fs pulses centred at 800 nm with an output of 7 W. For the ultrafast TA measurements, a portion of the laser fundamental was frequency doubled using a 1 mm thick BBO crystal for sample excitation at 400 nm, whilst the third harmonic (355 nm) of an electronically triggered, Q-switched Nd:YVO_4 laser (Innolas Picolo 25) provided the ~1 ns pump pulses for the nanosecond-microsecond (1 ns–100 µs) TA measurements. The probe was provided by a broadband visible (525–775 nm) non-colinear optical parametric amplifier (NOPA). The probe pulses are collected with a Si dual-line array detector (Hamamatsu S8381-1024Q), driven and read out by a custom-built board from Stresing Entwicklungsbüro. For determining the triplet excited state absorption of the terminal emitters, the probe was instead generated by a LEUKOS Disco 1 UV low timing jitter supercontinuum laser (STM-1-UV), which was then electronically delayed relative to the femtosecond 400 nm excitation by an electronic delay generator (Stanford Research Systems DG645).

Data in solution

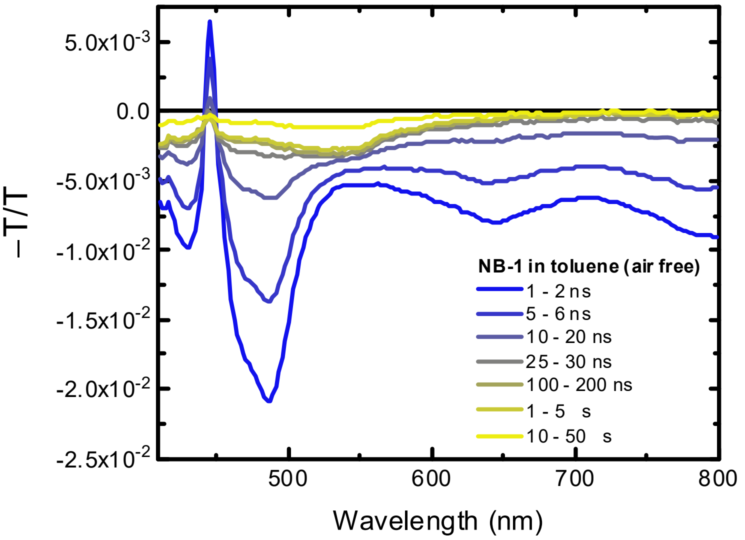

Supplementary Fig. 92: Transient absorption spectra recorded for **NB-1** in toluene (exc. 400 nm).

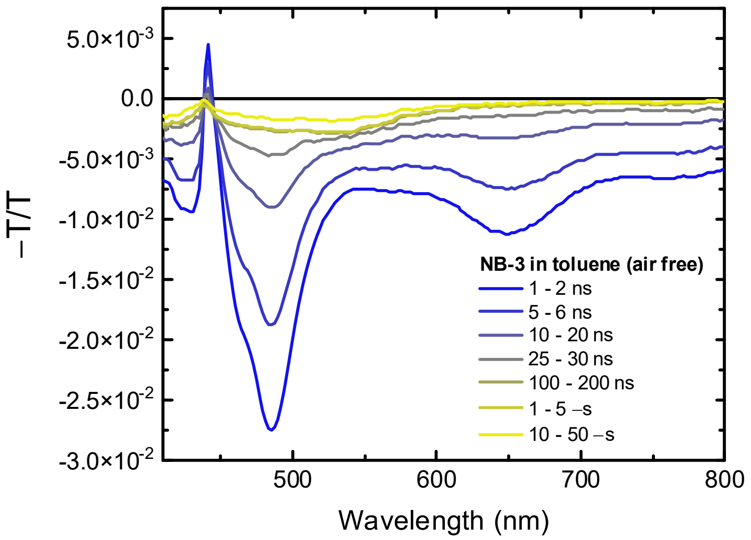

Supplementary Fig. 93: Transient absorption spectra recorded for **NB-3** in toluene (exc. 400 nm).

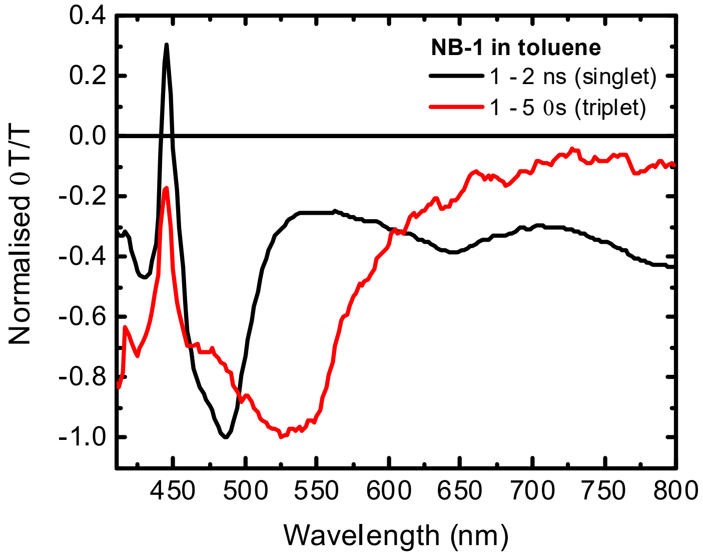

Supplementary Fig. 94: Decays of the (490 – 560 nm) PIA for **NB-1** in toluene (exc. 400 nm) in the presence and absence of air.

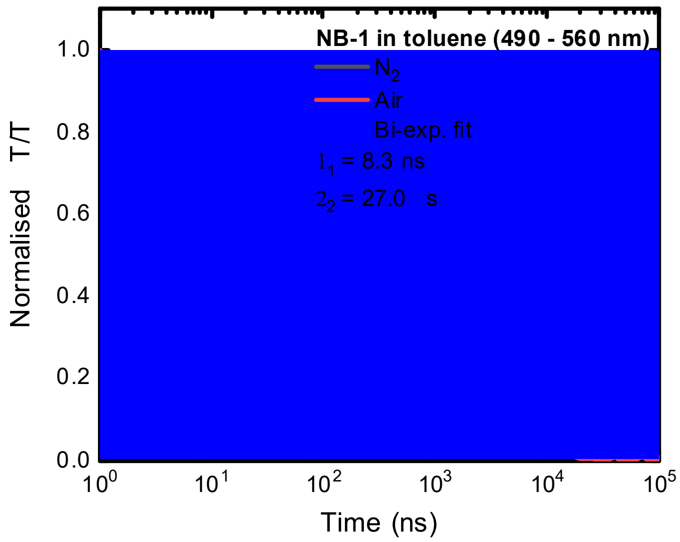

Supplementary Fig. 95: Decays of the (490 – 560 nm) PIA for **NB-1** in toluene (exc. 400 nm) in the presence and absence of air.

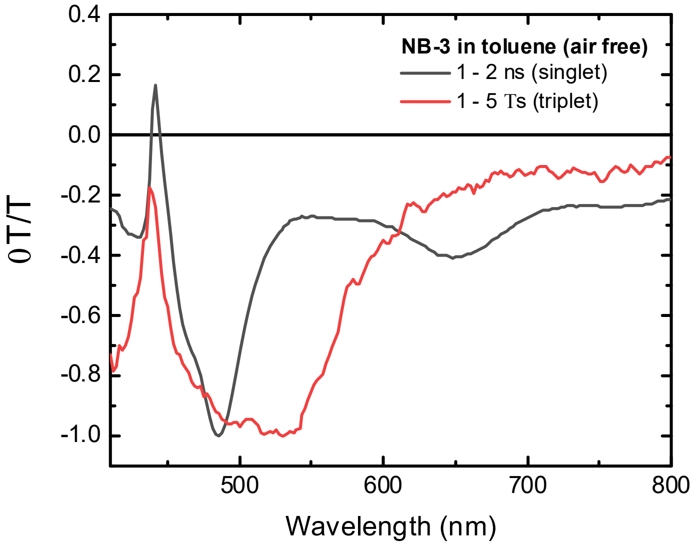

Supplementary Fig. 96: Transient absorption spectra recorded for **NB-3** in toluene (exc. 400 nm) at short (ns) and long (µs) timescales to assign S_1 and T_1 PIAs.

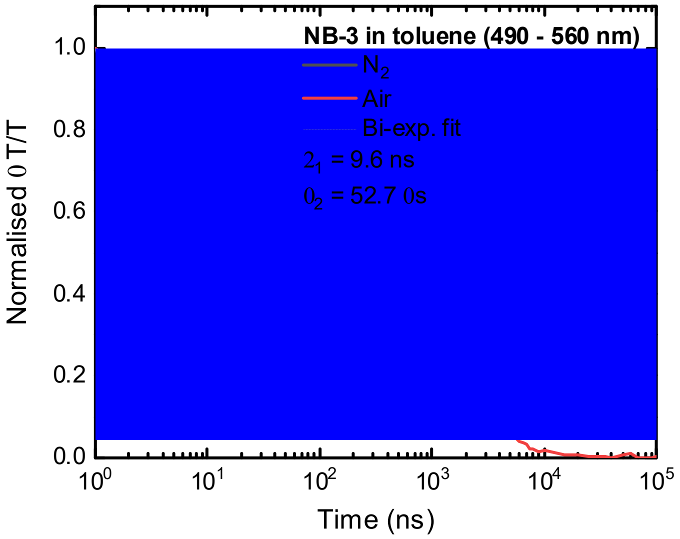

Supplementary Fig. 97: Decays of the (490 – 560 nm) PIA for **NB-3** in toluene (exc. 400 nm) in the presence and absence of air.

Data in wide-gap host

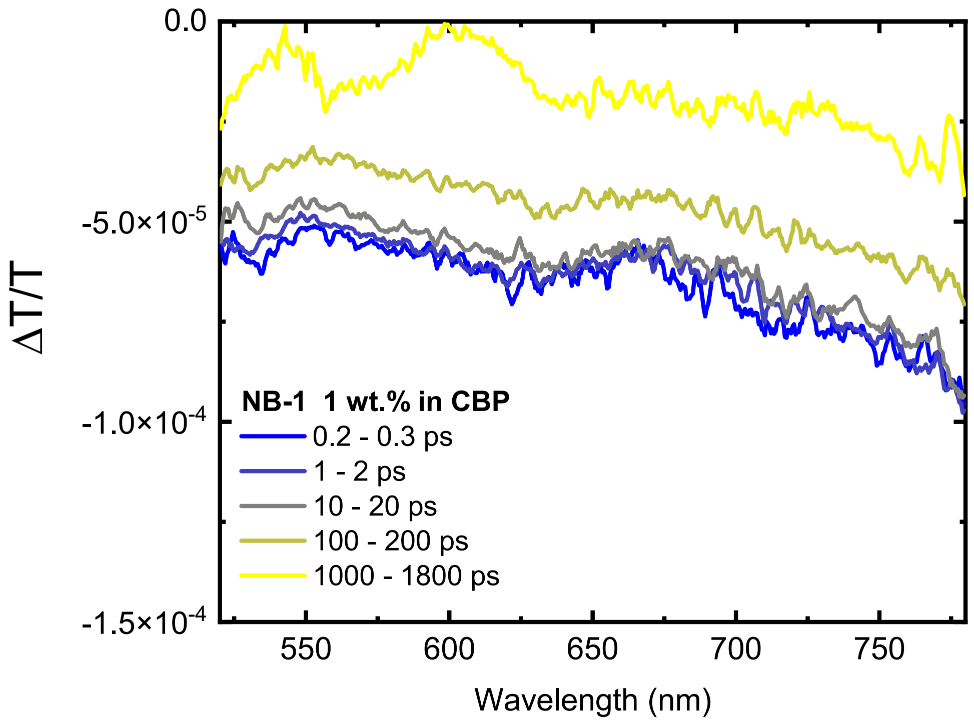

Supplementary Fig. 98: Transient absorption spectra recorded for **NB-1** in CBP (exc. 400 nm).

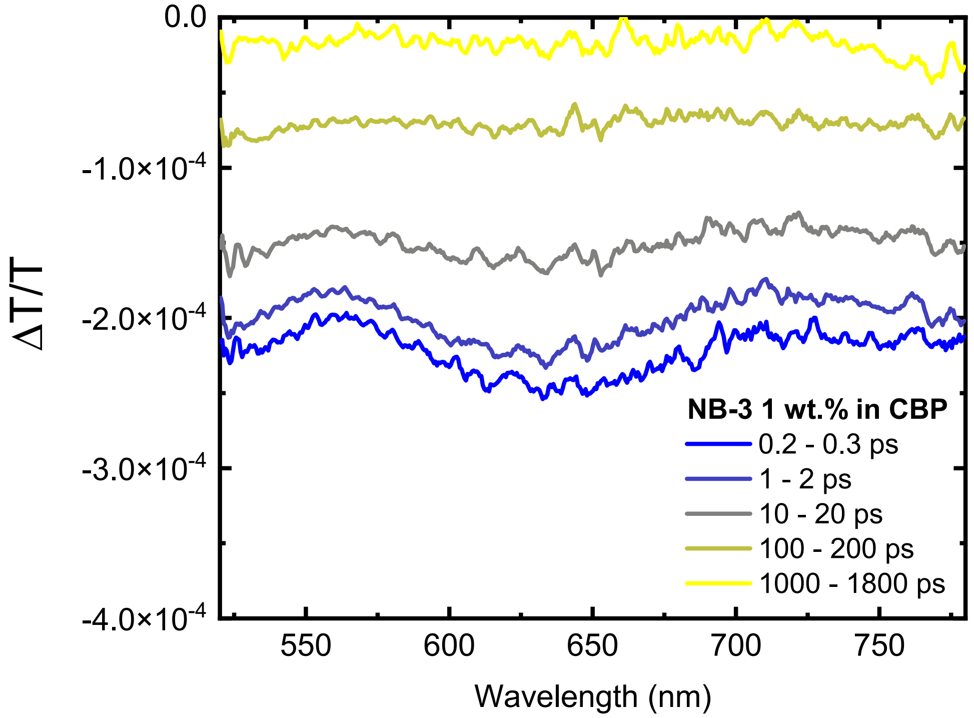

Supplementary Fig. 99: Transient absorption spectra recorded for **NB-3** in CBP (exc. 400 nm).

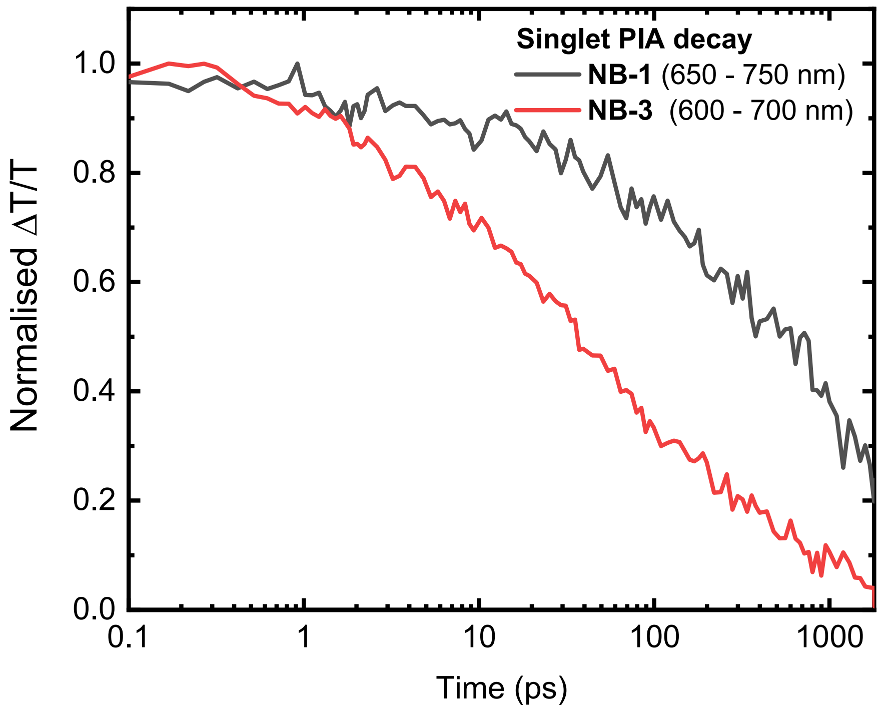

Supplementary Fig. 100: Decays for the S_1 PIAs of **NB-1** and **NB-3** in CBP (exc. 400 nm).

Data in DMAC-DPS

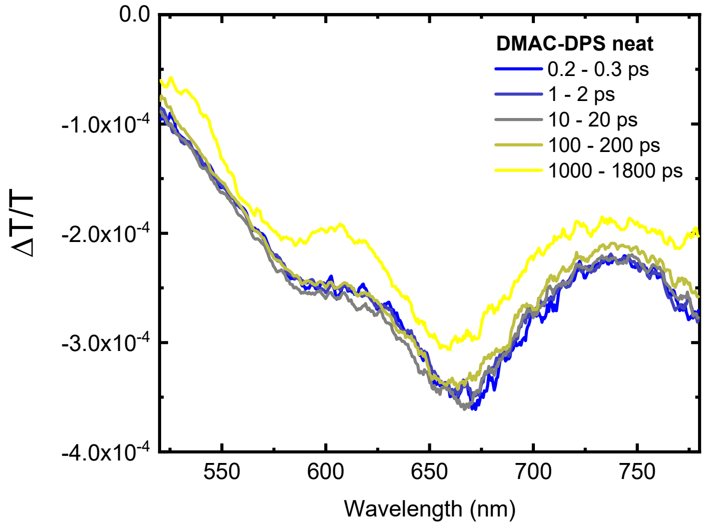

Supplementary Fig. t: Short-time transient absorption spectra recorded for DMAC-DPS (exc. 400 nm).

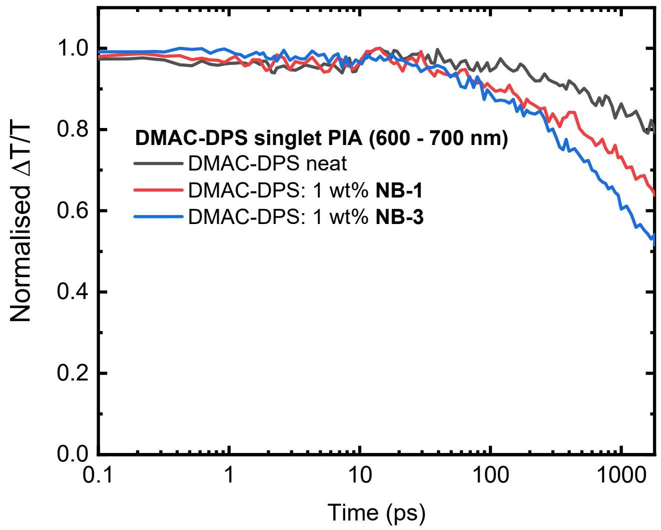

Supplementary Fig. 102: Decays for the short-time S_1 PIA of DMAC-DPS, pristine and doped with 1 wt.% **NB-1** and **NB-3** (exc. 400 nm).

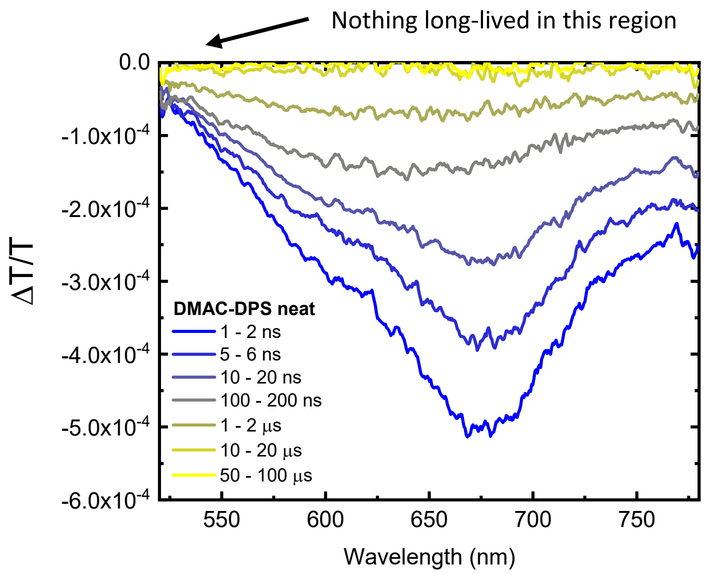

Supplementary Fig. 103: Long-time transient absorption spectra recorded for DMAC-DPS (exc. 355 nm).

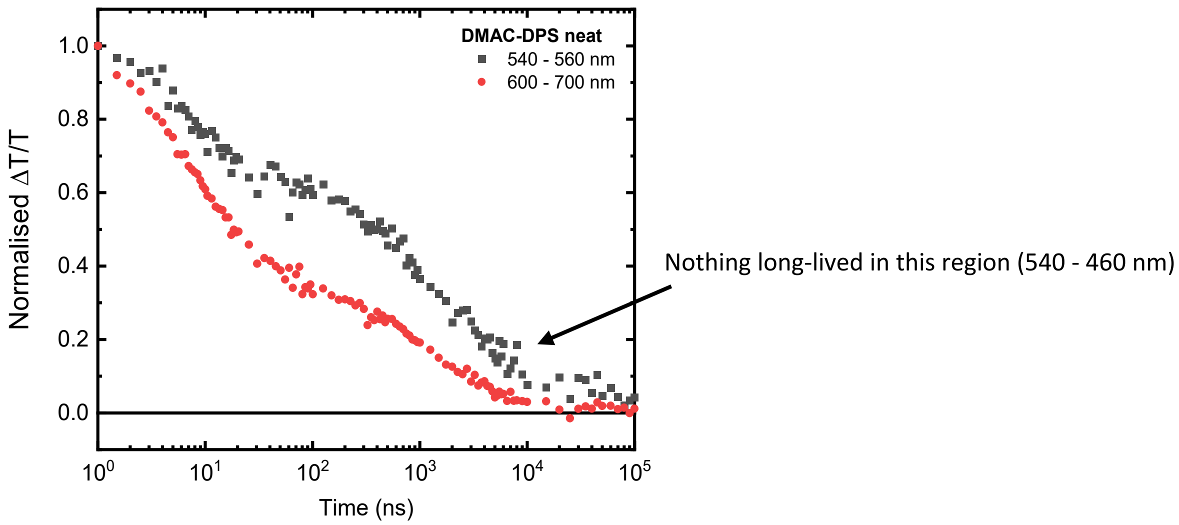

Supplementary Fig. 104: Decays for different regions of the PIA of DMAC-DPS (exc. 355 nm).

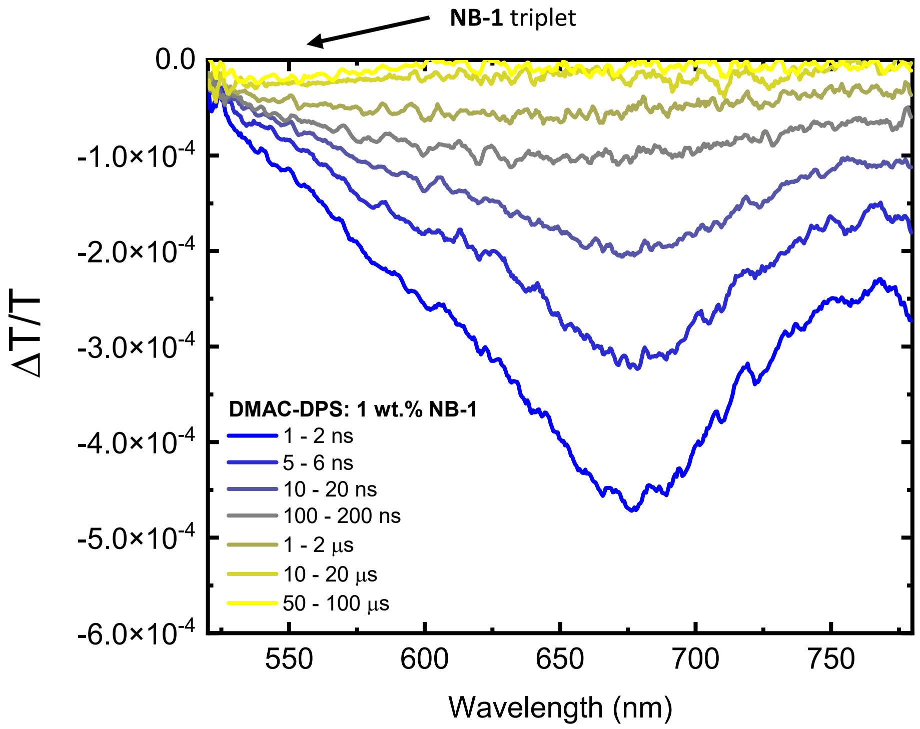

Supplementary Fig. 105: Long-time transient absorption spectra recorded for 1 wt.% **NB-1** in DMAC-DPS (exc. 355 nm).

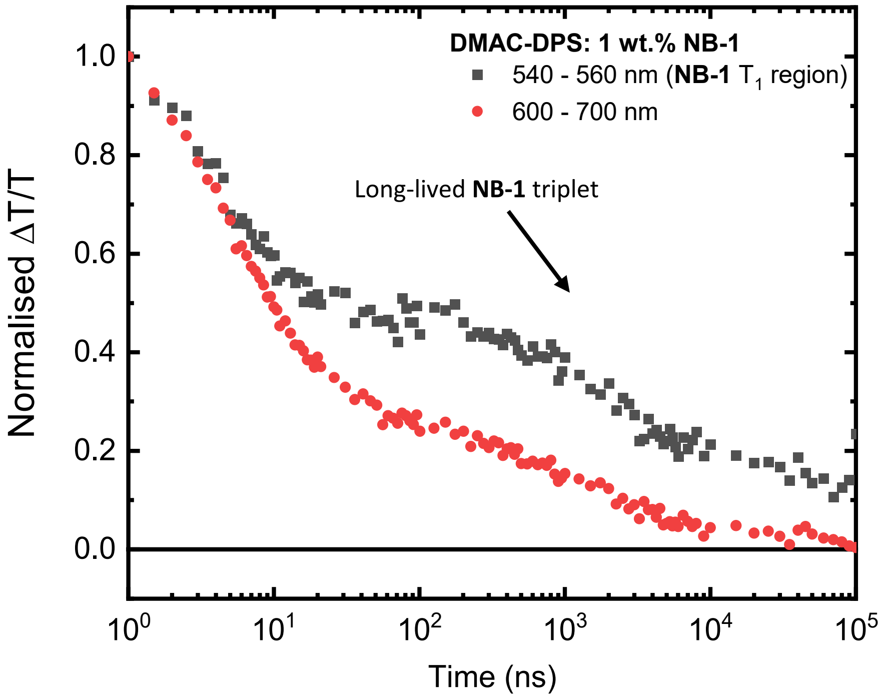

Supplementary Fig. 106: Decays for different regions of the PIA of DMAC-DPS with 1 wt.% **NB-1** (exc. 355 nm).

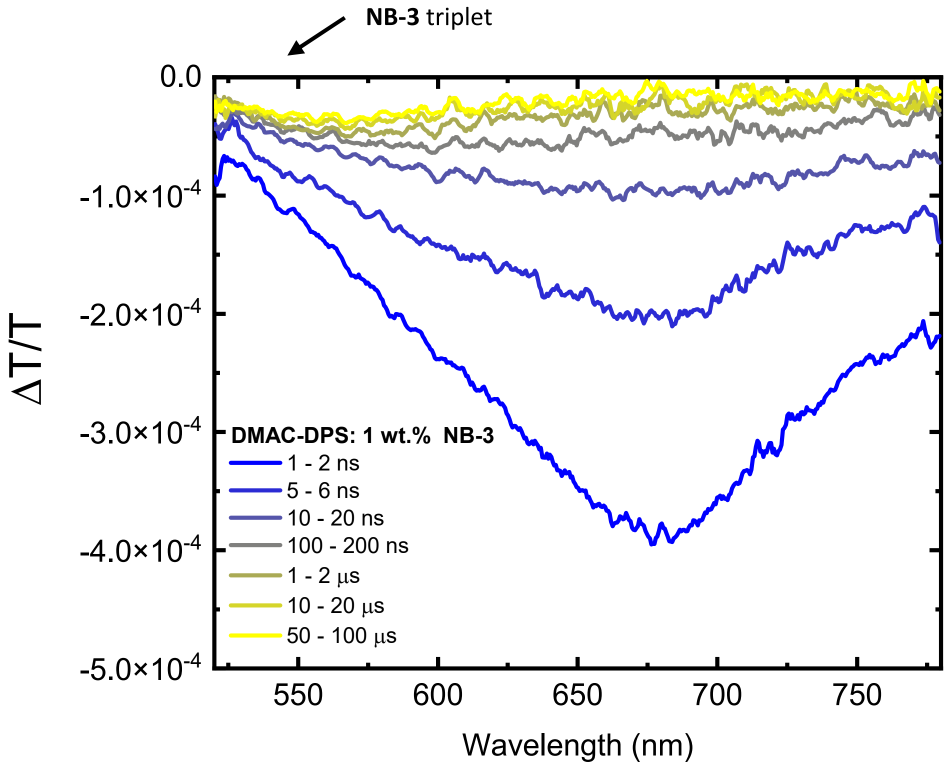

Supplementary Fig. 107: Long-time transient absorption spectra recorded for 1 wt.% **NB-3** in DMAC-DPS (exc. 355 nm).

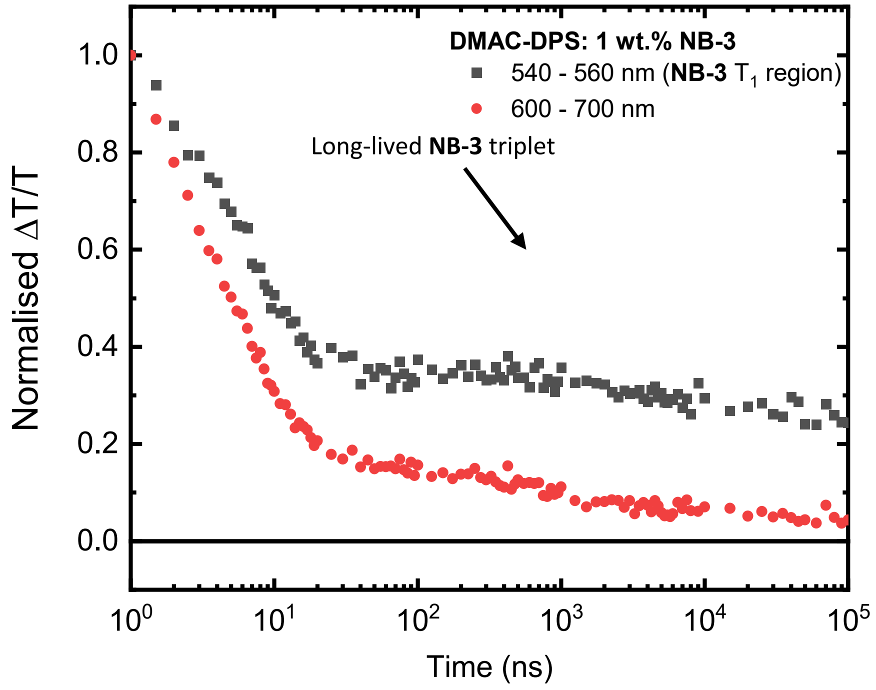

Supplementary Fig. 108: Decays for different regions of the PIA of DMAC-DPS with 1 wt.% **NB-3** (exc. 355 nm).

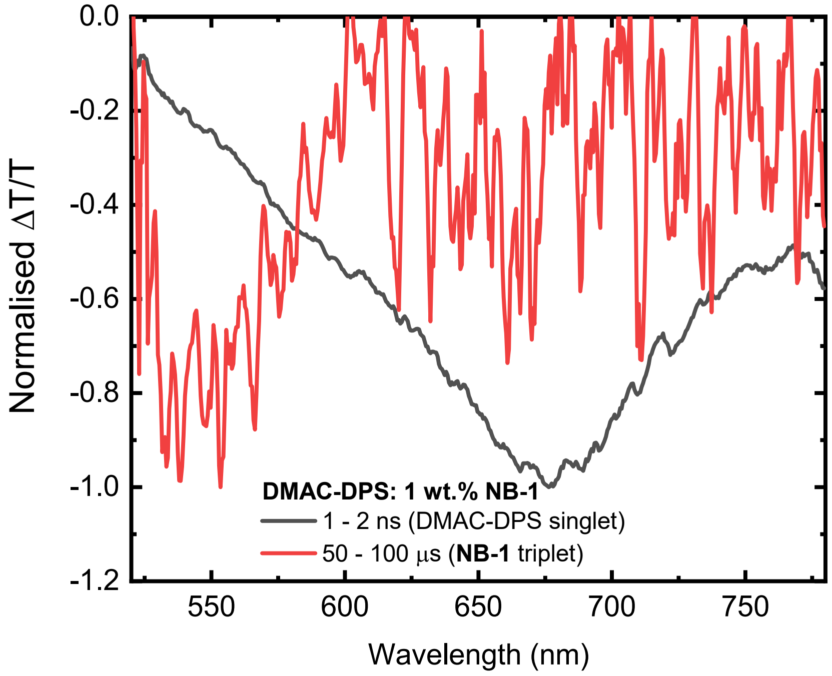

Supplementary Fig. 109: Normalised transient absorption spectra recorded for 1 wt.% **NB-1** in DMAC-DPS (exc. 355 nm) at short (ns) and long (µs) timescales.

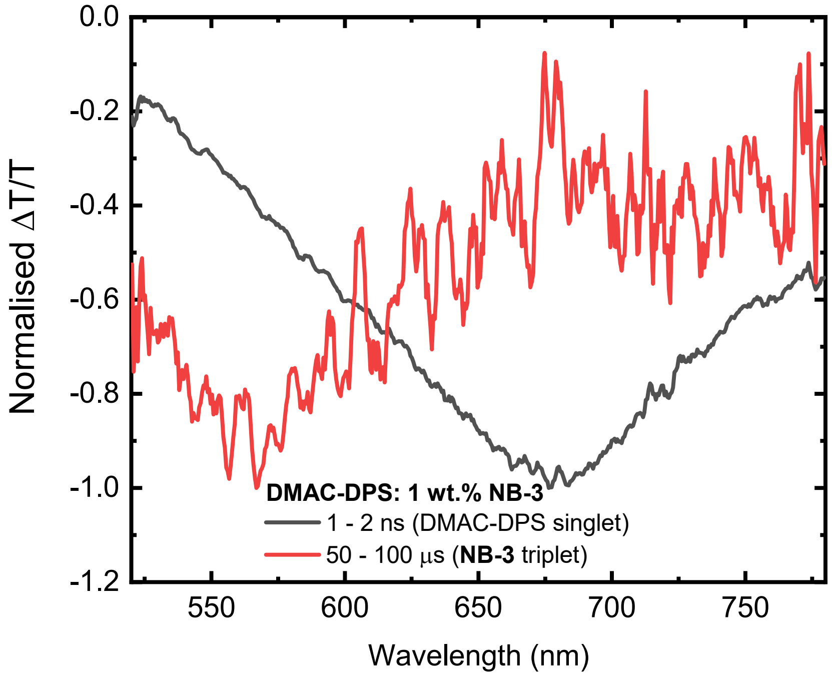

Supplementary Fig. 110: Normalised transient absorption spectra recorded for 1 wt.% **NB-3** in DMAC-DPS (exc. 355 nm) at short (ns) and long (µs) timescales.

Supplementary Note 7 - Electroluminescence

Experimental

For the fabrication of OLED devices, ITO coated substrates (~15 Ω/cm^2) were cleaned with acetone and isopropyl alcohol, and then O_2 plasma treatment was applied to align the energy level with a hole transporting layer. All layers, including organic layers and a LiF/aluminium cathode, were thermally deposited in high vacuum (~10^{-7} torr). The performance of the OLED devices was measured by a Keithley 2635 source-meter and a calibrated Si photodiode. The EL spectra were recorded by an Ocean Optics Flame spectrometer.

Device characterisation: DMAC-DPS:tbu-pyrene

The simple device structure displayed in Supplementary Fig. 111 was used in this study. 1,1-Bis[(di-4-tolylamino)phenyl]cyclohexane (TAPC) and 2,2′-di(9H-carbazol-9-yl)-1,1′-biphenyl (oCBP) were employed in an HTL part, and diphenyl-4-triphenylsilylphenyl phosphine oxide (TSPO1) was employed as an ETL. One of the conventional blue emitters, tbu-pyrene, is used as a dopant in DMAC-DPS-based devices as shown in Supplementary Fig. 111. Device performance is shown in Supplementary Fig. 112, showing the maximum EQEs of only 5-7%.

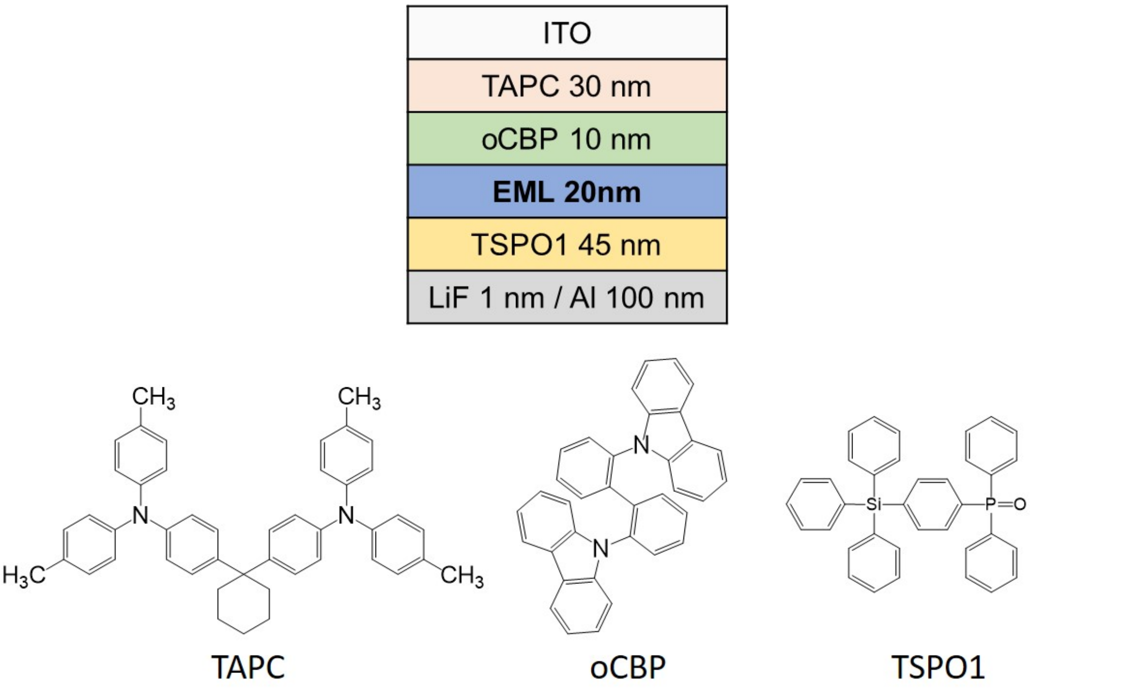

Supplementary Fig. 111: Schematics of the device stack and chemical structures of charge transporting layers employed in matrix-free OLEDs.

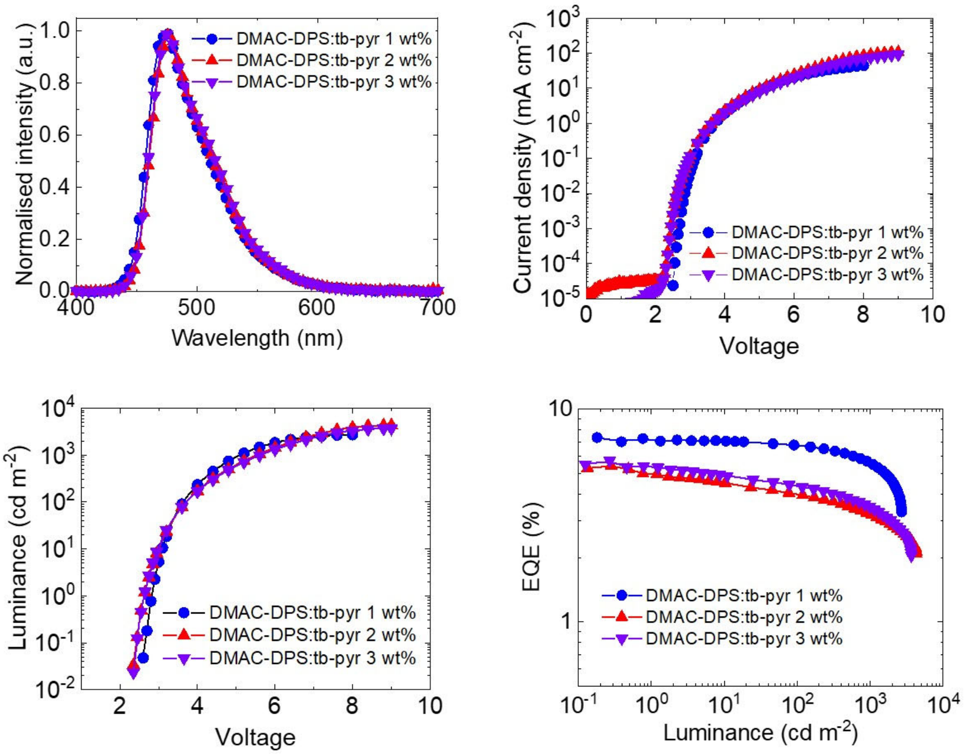

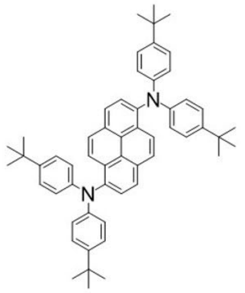

tBu-pyr

	V_{on} (V)	EQE_{Max} (%)	EQE_{1000}[a)] (%)	$Luminance_{Max}$ (cd m^{-2})	λ_{max} (nm)	CIE (x,y)
Device 2	2.5	7.4	5.6	2698.8	473	0.144, 0.281
Device 3	2.2	5.4	3.2	4294.9	476	0.144, 0.314
Device 4	2.2	5.7	3.4	3672.0	476	0.145, 0.314

a) EQE at 1000 cd m^{-2}

Supplementary Fig. 112: Data for matrix-free hyperfluorescent OLEDs based on tBu-pyr with DMAC-DPS host.

Device characterisation of MFHF: DMAC-DPS: NB-1, NB-2, and NB-3

Next, NB-1 and NB-3 emitters were employed in the same device structure shown in Supplemtarry Fig. 111, and DMAC-DPS was used as a TADF host. Supplementary Fig.114-116 show the device characteristics of spectra, EQE, current density, and luminance, and they are summarised in Supplementary Table 13. DMAC-DPS:NB-3 devices show a substantial efficiency drop (≤ 5% from 1 wt%) as doping concentration increases, while DMAC-DPS:NB-1 and NB-2 devices keep high efficiency (> 15%) at 1 wt% doping and higher. Also, NB-3 shows broad spectra due to aggregation, whereas NB-1 and NB-2 show narrow emission (FWHM ≤ 15 nm) at 1 wt% doping and higher.

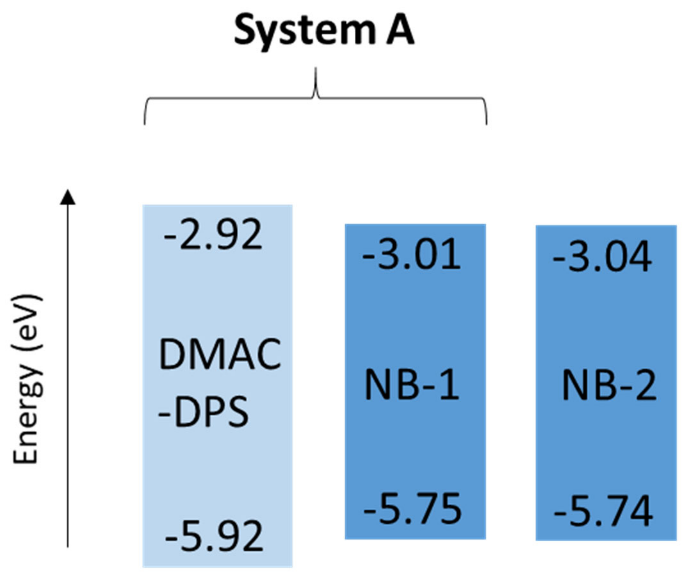

Supplementary Fig. 113: Energy levels of the encapsulated emitters vs. DMAC-DPS.

Supplementary Table 13: DMAC-DPS-based non-doped TADF and MFHF OLED data

Emitter	Host	Emitter wt.%	V_{on}[a)] (V)	EQE_{Max} (%)	V_{100}[b)] (V)	EQE_{100}[b)] (%)	$Luminance_{Max}$ ($cd{\cdot}m^{-2}$)	λ_{peak} (nm)	FWHM (nm)	CIE (x,y)
**nondoped**	**DMAC-DPS**	-	2.7	17.0	3.3	15.8	2261.7	476	88.3	0.185, 0.315
**NB-1**	**DMAC-DPS**	0.5	2.6	18.8	3.4	16.7	4531.8	449	60.3	0.176, 0.247
		0.75	2.6	21.1	3.4	19.3	4285.1	449	47.8	0.174, 0.225
		1.0	2.7	16.4	3.5	14.6	2103.0	449	14.5	0.169, 0.184
		2.0	2.7	13.5	3.6	11.4	1485.5	449	12.6	0.166, 0.154
**NB-2**	**DMAC-DPS**	0.5	2.6	19.1	3.4	18.4	2448.4	457	49.9	0.175, 0.242
		0.75	2.6	19.0	3.4	18.4	2190.5	456	19.8	0.168, 0.212
		1.0	2.6	15.6	3.5	14.5	1754.5	456	15.2	0.172, 0.191
		2.0	2.6	12.6	3.5	12.2	1454.7	456	14.1	0.161, 0.161
**NB-3**	**DMAC-DPS**	0.5	2.6	14.1	3.7	12.9	2284.2	449	60.3	0.173, 0.234
		0.75	2.6	10.0	4.2	9.1	1699.7	449	61.3	0.165, 0.221
		1.0	2.6	5.2	4.4	4.2	1202.7	479	77.8	0.170, 0.293
		2.0	2.6	4.2	4.8	3.5	928.4	476	62.5	0.158, 0.266

[a)] Voltage at 0.1 cd/m^2; [b)] Values at 100 cd/m^2

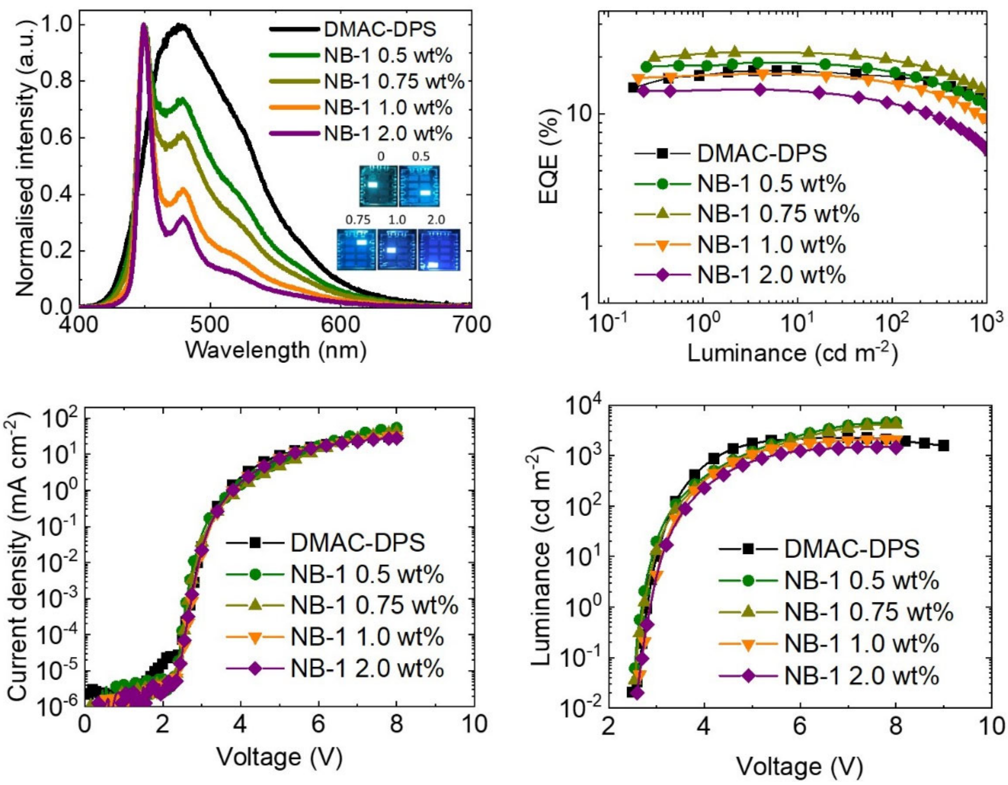

Supplementary Fig. 114: OLED data for DMAC-DPS matrix-free devices with **NB-1** as the terminal emitter.

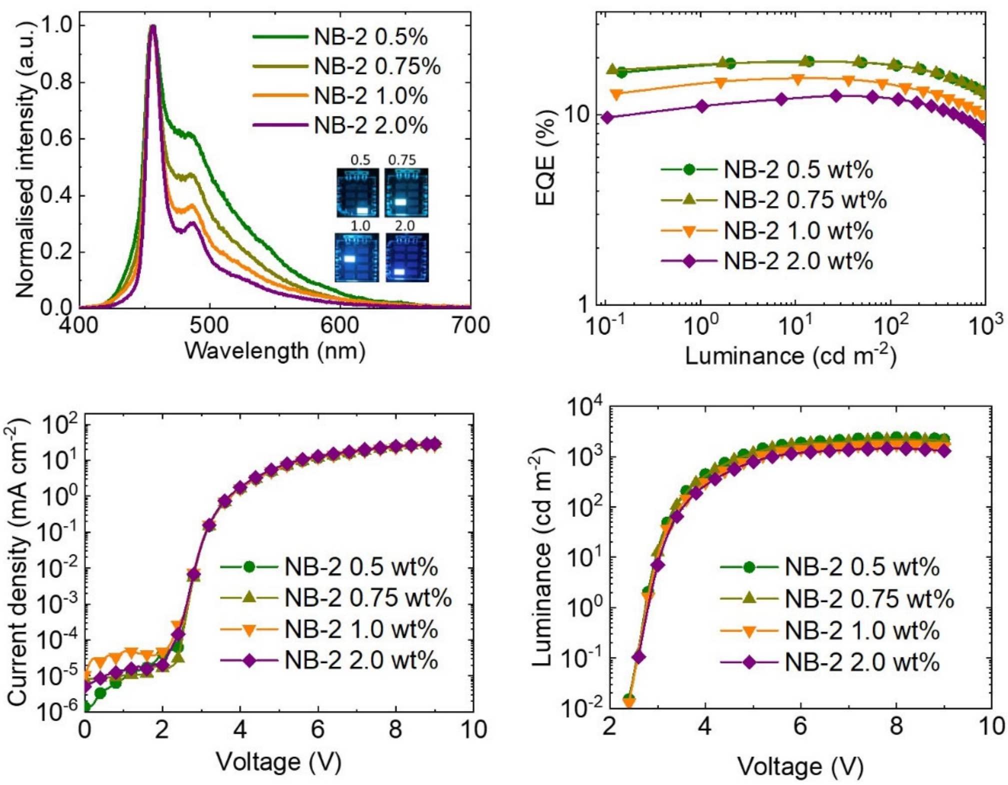

Supplementary Fig. 115: OLED data for DMAC-DPS matrix-free devices with **NB-2** as the terminal emitter.

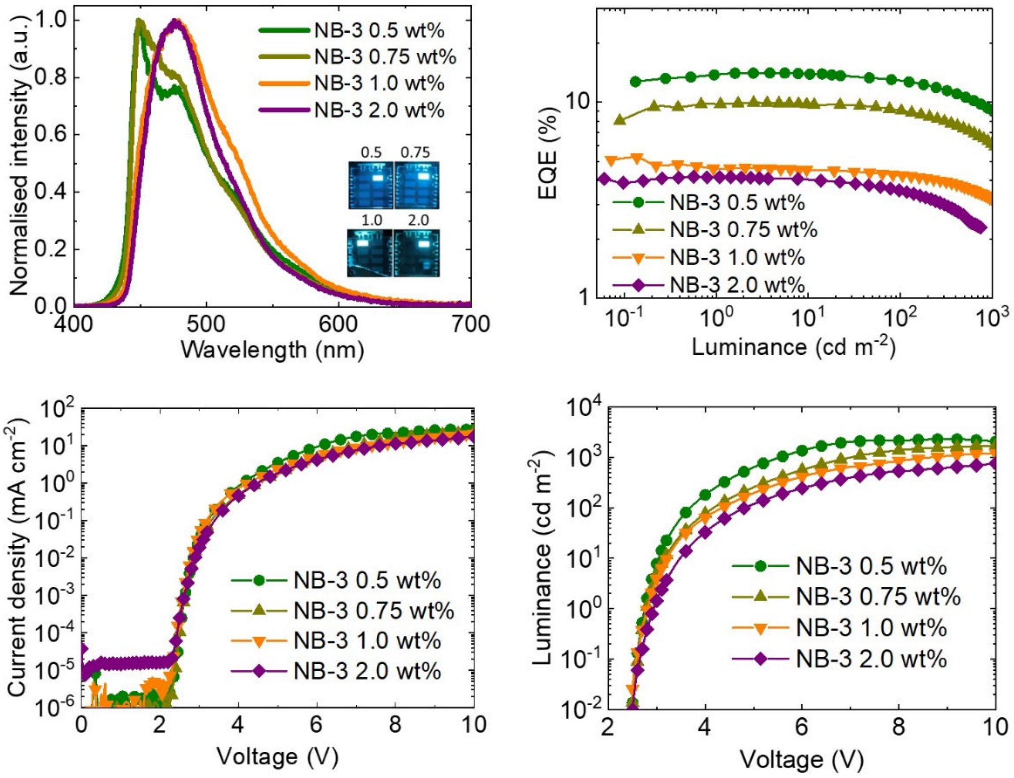

Supplementary Fig. 116: OLED data for DMAC-DPS matrix-free devices with **NB-3** as the terminal emitter.

Device characterisation of MFHF: DPAc-DCzBN: NB-1, NB-2 and NB-3

Next, DPAc-DCzBN was selected as an exciton donor for NB-1, NB-2 and NB-3, and these MFHF systems are applied in the device structure shown in Supplementary Fig. 111. These device characteristics such as spectra, EQE, current density, and luminance are presented in Supplementary Fig.119-121, and they are summarised in Supplementary Table 14. As the nondoped DPAc-DCzBN device show higher than 20% EQE, The DPAc-DCzBN MFHF device also shows higher maximum EQEs than the DMAC-DPS devices above. Therefore, with NB-2, DPAc-DCzBN MFHF devices show higher than 20% EQEs at ≤1 wt% while maintaining excellent deep blue CIEx,y and narrow emission FWHM of ~15 nm.

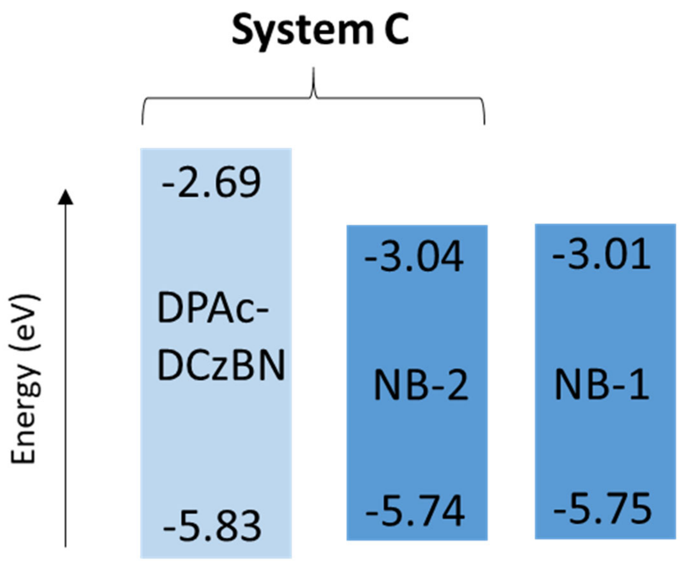

Supplementary Fig. 117: Energy levels of the encapsulated emitters vs. DPAc-DCzBN.

Supplementary Table 14: DPAc-DCzBN-based nondoped TADF and MFHF OLED data

Emitter	Host	Emitter wt.%	V_{on}[a)] (V)	EQE_{Max} (%)	V_{100}[b)] (V)	EQE_{100}[b)] (%)	$Luminance_{Max}$ ($cd{\cdot}m^{-2}$)	λ_{peak} (nm)	FWHM (nm)	CIE (x,y)
**nondoped**	**DPAc-DCzBN**	-	2.9	20.3	4.4	18.7	2563.2	476	86.3	0.186, 0.313
**NB-1**	**DPAc-DCzBN**	0.5	2.9	18.2	4.3	16.2	4259.0	450	73.4	0.175, 0.242
		0.75	2.9	15.9	4.7	13.8	3644.3	450	51.6	0.168, 0.212
		1.0	3.0	14.3	4.2	11.6	1297.0	450	18.5	0.172, 0.192
		2.5	2.9	10.4	5.3	5.6	1681.4	450	13.7	0.161, 0.161
**NB-2**	**DPAc-DCzBN**	0.5	3.1	22.2	4.4	18.7	2577.6	458	64.0	0.177, 0.265
		0.75	3.0	21.6	4.3	18.6	2140.8	458	44.6	0.172, 0.236
		1.0	3.0	21.5	4.4	17.8	2494.0	458	15.4	0.161, 0.186
		1.5	3.1	18.5	4.6	15.6	1904.8	457	15.2	0.158, 0.168
		2.5	3.2	15.3	4.8	11.4	1386.3	457	14.6	0.159, 0.156
**NB-3**	**DPAc-DCzBN**	0.5	3.1	18.3	4.2	16.6	1290.9	469	75.5	0.173, 0.264
		1.0	3.2	11.2	4.5	9.7	1400.0	451	61.2	0.164, 0.212
		2.5	3.4	6.8	5.5	4.6	887.4	464	58.9	0.155, 0.213

[a)] Voltage at 0.1 cd/m^2; [b)] Values at 100 cd/m^2

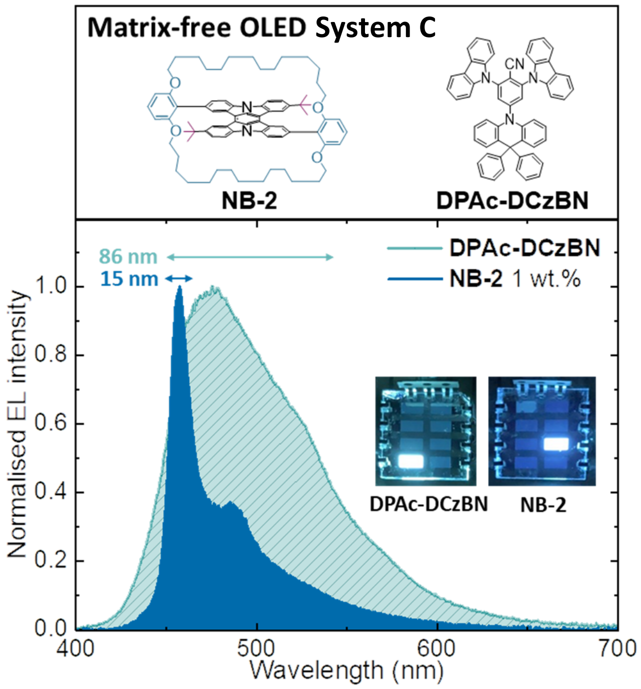

Supplementary Fig. 118: EL spectra for matrix-free OLED system C, insets are photographs of OLED emission.

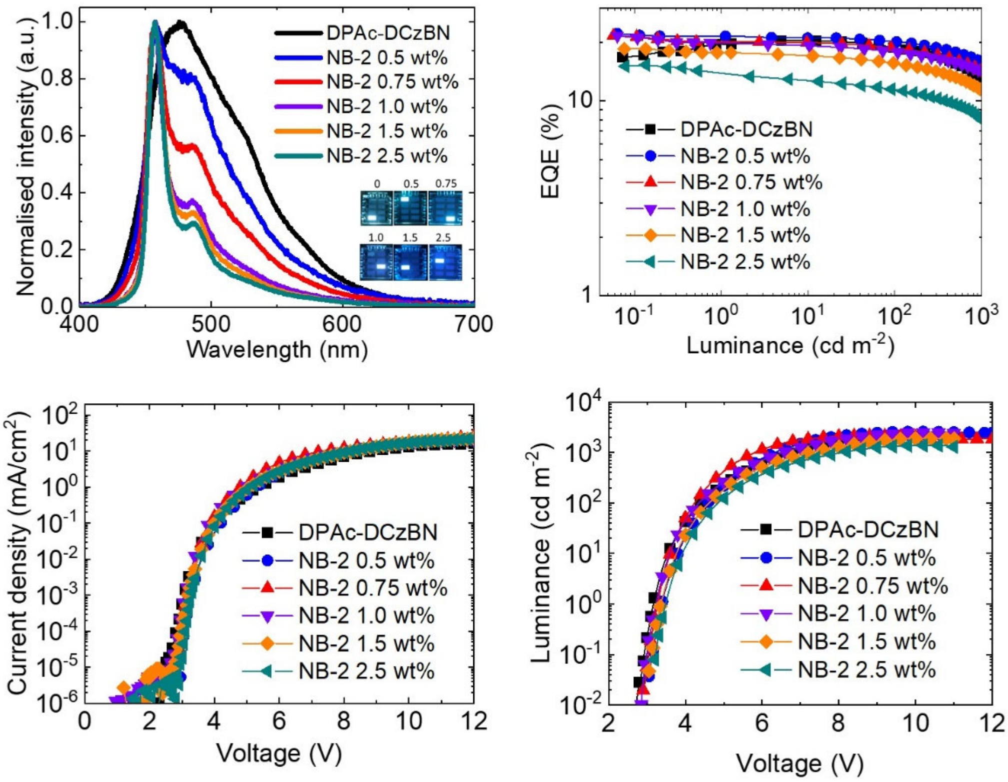

Supplementary Fig. 119: OLED data for DPAc-DCzBN matrix-free devices with **NB-2** as the terminal emitter.

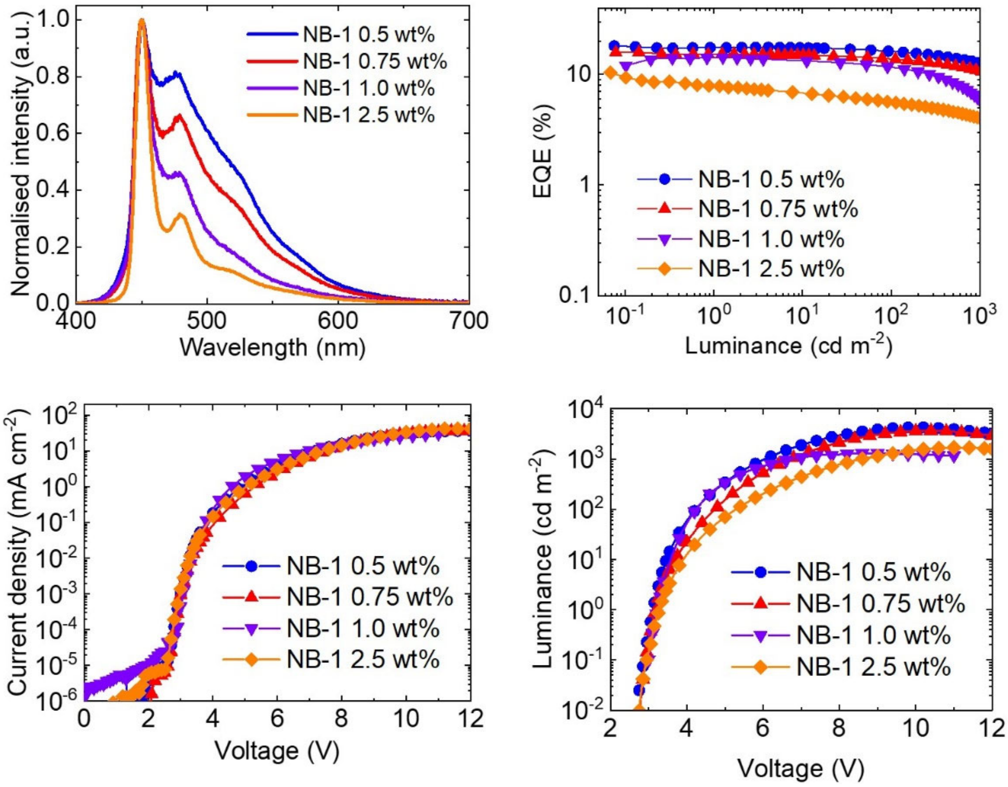

Supplementary Fig. 120: OLED data for DPAc-DCzBN matrix-free devices with **NB-1** as the terminal emitter.

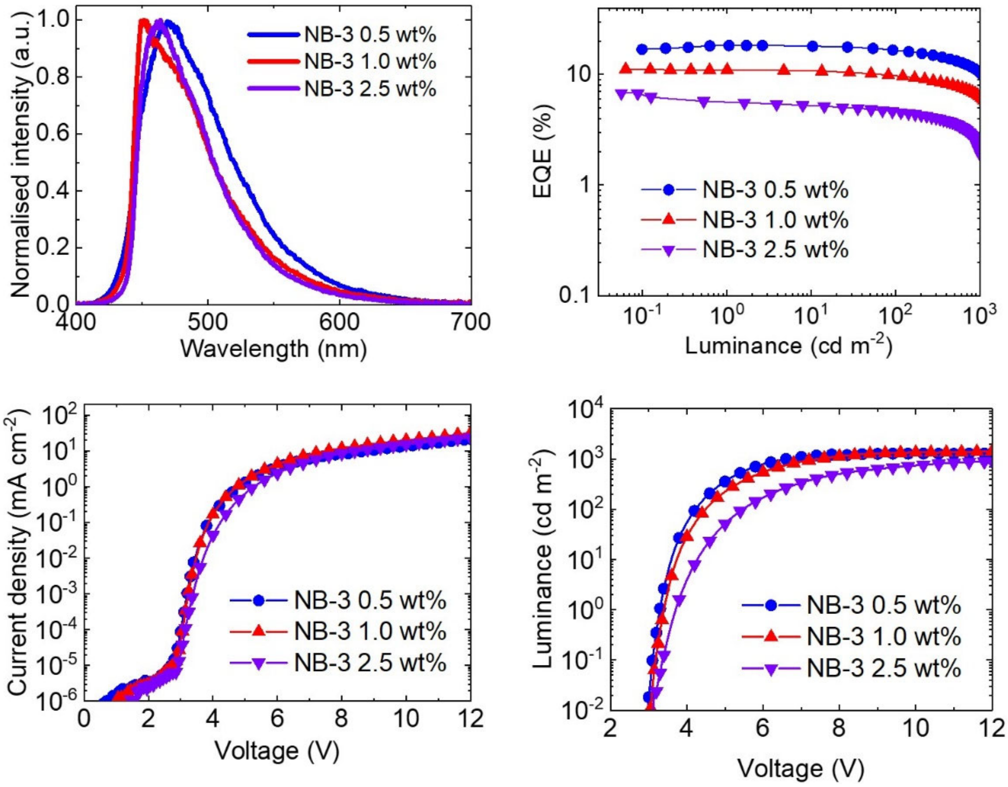

Supplementary Fig. 121: OLED data for DPAc-DCzBN matrix-free devices with **NB-3** as the terminal emitter.

Device characterisation of MFHF: high doping (5, 10 wt%)

For exploring the device characteristics at high doping concentrations, the devices with 5 and 10 wt% doping concentrations were fabricated using the device structure shown in Supplementary Fig. 111, and their performance is plotted in Supplementary Fig. 122 and 123 and summarised in Supplementary Table 15.

Supplementary Table 15: MFHF OLED data for high doping

Emitter	Host	Emitter wt.%	V_{on} [a] (V)	EQE_{Max} (%)	V_{100} [b] (V)	EQE_{100} [b] (%)	$Luminance_{Max}$ ($cd \cdot m^{-2}$)	λ_{peak} (nm)	FWHM (nm)	CIE (x,y)
NB-2	DPAc-DCzBN	5.0	3.2	8.5	6.0	4.1	550.6	457	12.5	0.153, 0.119
		10.0	3.3	5.0	7.9	1.9	261.4	457	12.6	0.154, 0.111
NB-1	DMAC-DPS	5.0	2.9	7.8	4.5	6.7	748.5	449	12.3	0.165, 0.121
		10.0	3.0	4.6	5.1	3.3	330.3	449	11.4	0.170, 0.111

[a] Voltage at 0.1 cd/m^2; [b] Values at 100 cd/m^2

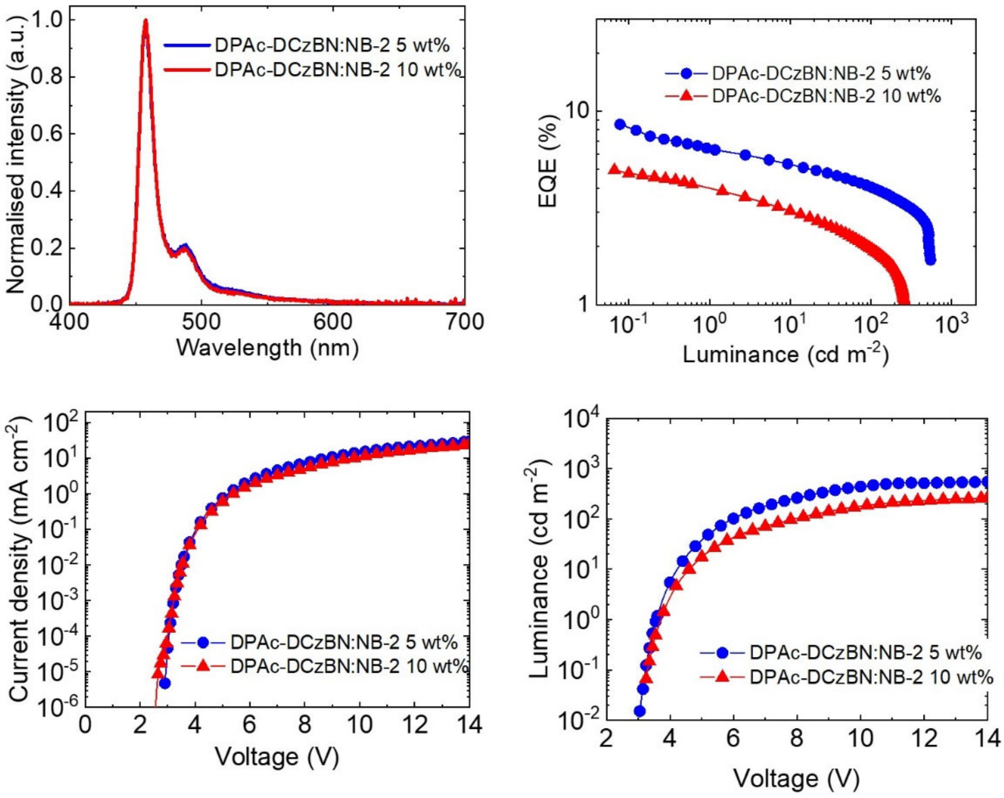

Supplementary Fig. 122: OLED data for DPAc-DCzBN matrix-free devices with **NB-2** as the terminal emitter.

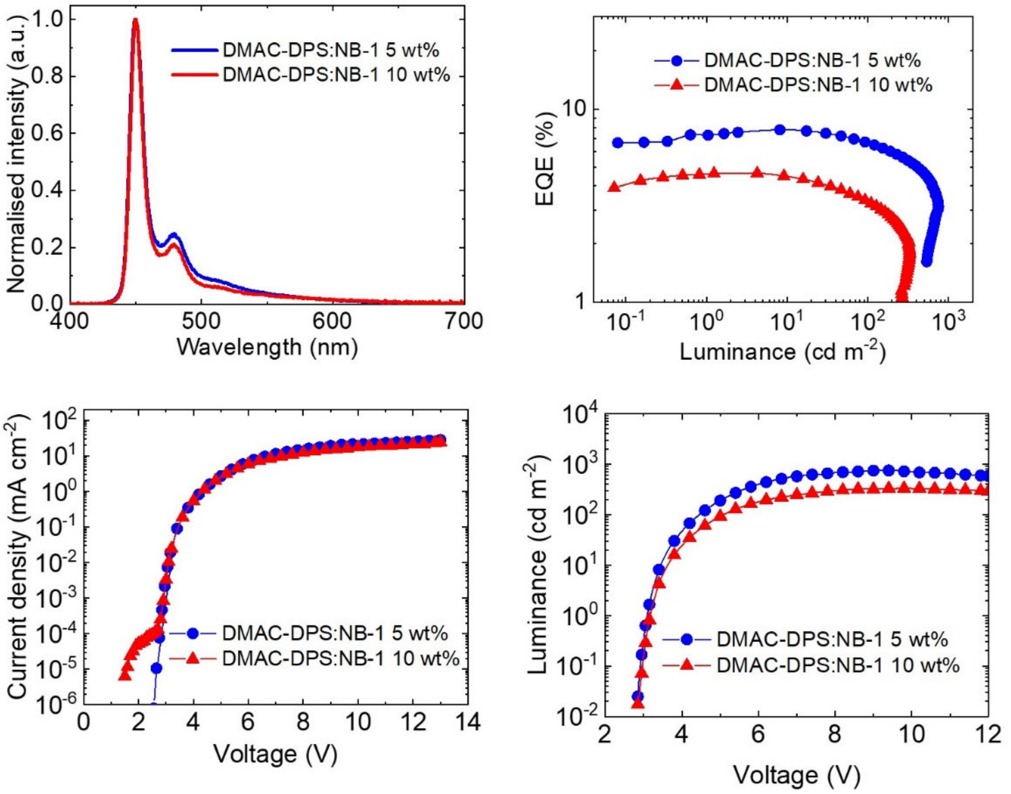

Supplementary Fig. 123: OLED data for DMAC-DPS matrix-free devices with **NB-2** as the terminal emitter.

Device characterisation: TTA OLEDs: NB-1 and NB-3

For triplet-triplet annihilation (TTA) OLEDs, one of the conventional anthracene derivatives, 2-Methyl-9,10-bis(naphthalen-2-yl)anthracene (MADN), was used as a host and SF3-TRz / 4,6-bis(3,5-di(pyridin-3-yl)phenyl)-2-methylpyrimidine (B3PYMPM) layers are deposited in the ETL part. TAPC/ 4,4',4"-Tris(carbazol-9-yl)triphenylamine (TCTA) /mCP (1,3-di(9H-carbazol-9-yl)benzene) layers are deposited for the HTL part. This device structure is shown in Supplementary Fig. 124.

MADN:NB-1 TTA OLED results are shown in Supplementary Fig. 125 and summarised in Supplementary Table 16. Pure blue emission with FWHM of <12 nm and a peak wavelength of 450 nm is recorded.

MADN:NB-3 TTA OLED results are shown in Supplementary Fig. 126 and summarised in Supplementary Table 17. As expected from NB-3-based MFHF results above, the broad EL spectra are recorded, indicating the severe aggregation effect.

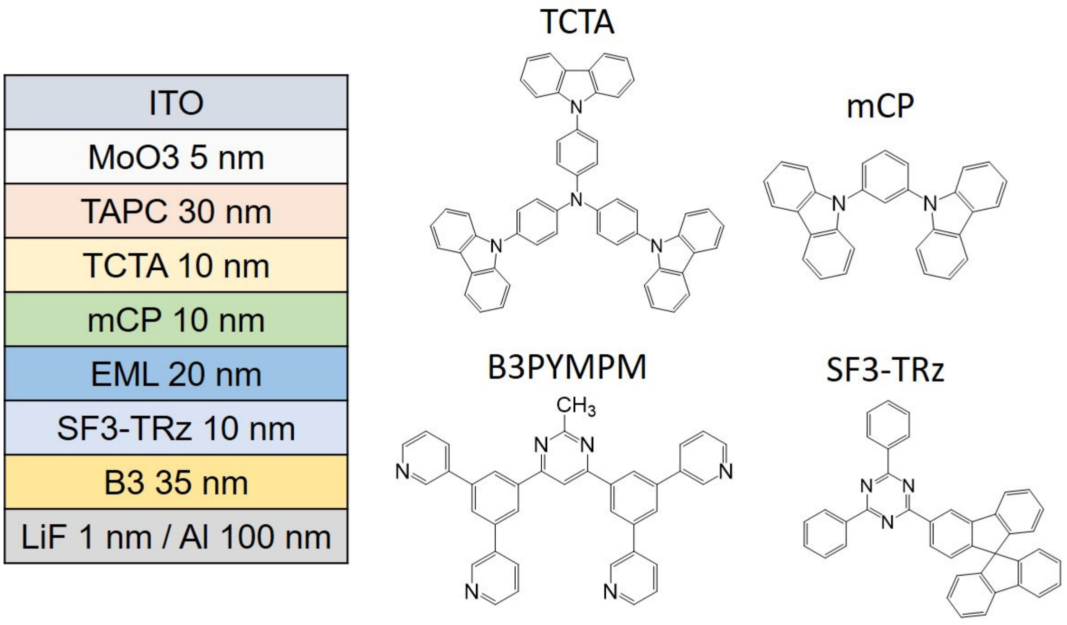

Supplementary Fig. 124: Device layout and chemical structures for TTA OLEDs.

Supplementary Table 16: MADN:**NB-1** TTA OLED data

Emitter	Host	Emitter wt.%	V_{on}[a)] (V)	EQE_{Max} (%)	V_{100}[b)] (V)	EQE_{100}[b)] (%)	$Luminance_{Max}$ ($cd \cdot m^{-2}$)	λ_{peak} (nm)	FWHM (nm)	CIE (x,y)
**NB-1**	**MADN**	0.5	2.9	7.0	5.1	5.1	8545.5	450	13.0	0.151, 0.060
		0.75	2.9	7.3	5.1	5.6	9028.8	450	11.8	0.149, 0.054
		1.0	2.9	8.3	5.0	6.2	10125.5	450	11.3	0.149, 0.049
		2.0	2.9	8.5	4.9	6.5	10908.6	450	11.9	0.149, 0.050
		3.0	2.9	7.8	5.1	6.1	9330.6	451	11.9	0.150, 0.051
		5.0	2.9	7.5	4.9	5.9	8694.0	451	12.6	0.150, 0.056

a) Voltage at 0.1 cd/m^2; b) Values at 100 cd/m^2

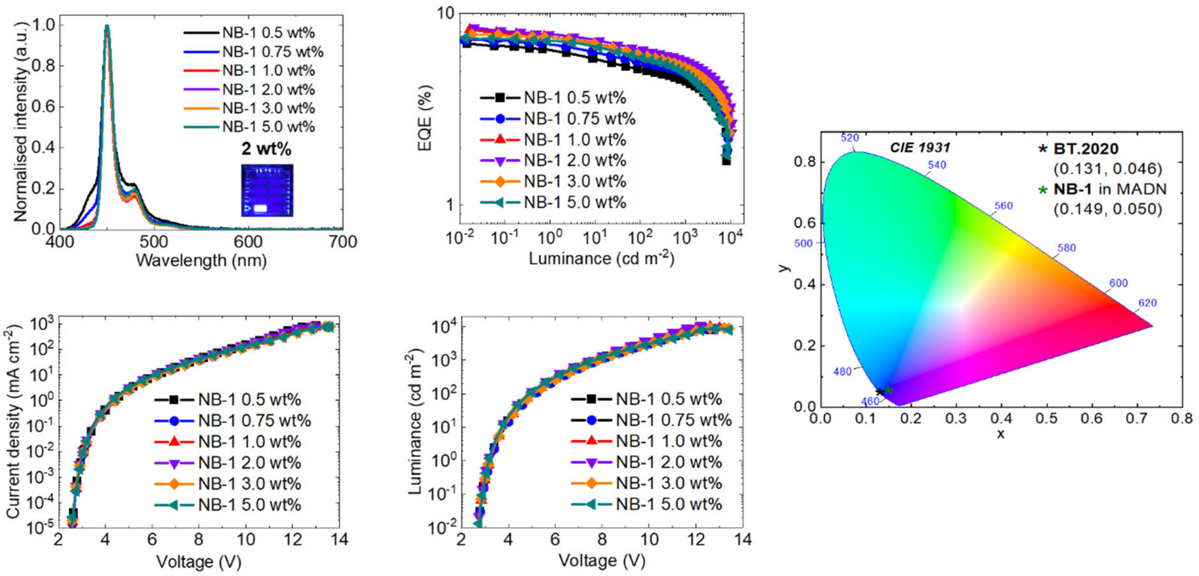

Supplementary Fig. 125: OLED data for MADN matrix-free devices with **NB-1** as the terminal emitter.

Supplementary Table 17: MADN:**NB-3** TTA OLED data

Emitter	Host	Emitter wt.%	V_{on} [a)] (V)	EQE_{Max} (%)	V_{100} [b)] (V)	EQE_{100} [b)] (%)	$Luminance_{Max}$ ($cd \cdot m^{-2}$)	λ_{peak} (nm)	FWHM (nm)	CIE (x,y)
**NB-3**	**MADN**	0.5	3.1	5.8	5.2	5.6	14268.9	449	43.0	0.145, 0.114
		1.0	3.3	5.4	5.4	5.4	20413.3	470	53.6	0.137, 0.195
		2.0	3.1	5.2	4.9	5.1	33102.4	477	58.3	0.152, 0.312
		5.0	3.1	4.9	4.9	4.8	31125.2	477	61.6	0.156, 0.330

[a)] Voltage at 0.1 cd/m^2; [b)] Values at 100 cd/m^2

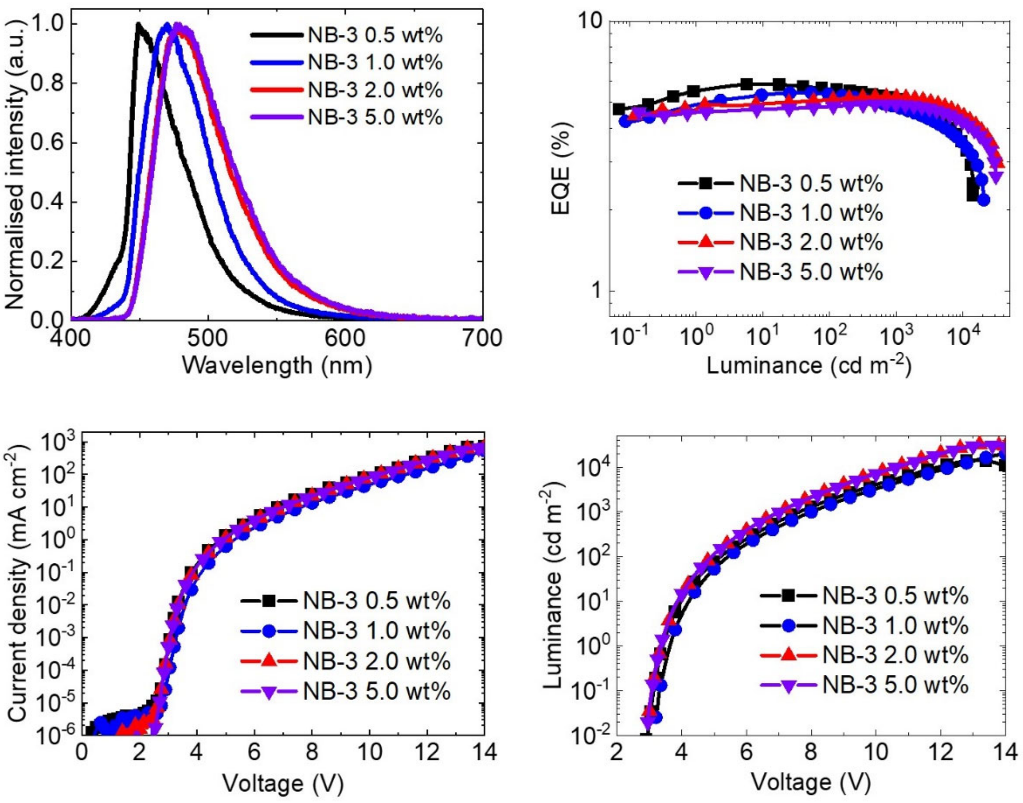

Supplementary Fig. 126: OLED data for MADN matrix-free devices with **NB-3** as the terminal emitter.

Device characterisation: MCHF, Device engineering of MFHF, TTA OLEDs and their stability

DMAC-DPS-based MCHF devices were fabricated by using the device structure shown in Supplementary Fig. 111, and their device performance is displayed in Supplementary Fig. 127 and summarised in Supplementary Table 18.

DPAc-DCzBN-based MCHF, MFHF, and TTA OLEDs were explored in terms of luminance and stability. The detailed device structures for OLEDs studied here are shown in Supplementary Fig. 128, and their optoelectronic performances are summarised in Tables S7.6 and S7.7. From the simple device structure shown in Supplementary Fig. 111, The ETL part was engineered by using 1,3,5-tri(m-pyridin-3-ylphenyl)benzene (TmPyPB), 2-(9,9'-spirobi[fluoren]-3-yl)-4,6-diphenyl-1,3,5-triazine (SF3-TRz), and Lithium quinolate (Liq).

As the widegap host, bis[2-(diphenylphosphino)phenyl]ether oxide (DPEPO), was used for MCHF, turn-on and driving voltage were considerably increased. Also, their EQEs are even lower than MFHF devices at the same doping, probably due to direct trapping of the charges at the NB-2 emitters, considering shallower J-V characteristics as doping concentrations increase.

Based on the device engineering of MFHF OLEDs (their device performance is shown in Supplementary Fig. 130-132), a very high maximum luminance of >20,000 cd m^{-2} and more than 150 times longer device stability was attained compared to MCHF OLEDs (Supplementary Table 19 and Fig. 134), and this is comparable to TTA OLEDs. This shows that with device engineering, device performance can be further improved. Also, these results are based on a lab-scale experiment (organic layers are exposed to nitrogen during evaporation steps, and lifetime was measured without encapsulation), so if the fabrication and measurement conditions are optimised, much more enhanced device stability may be obtained.

Supplementary Table 18: Summary of **NB-1**-based MCHF OLED data

No.	Host	NB-1 wt.%	V_{on} [a] (V)	EQE_{Max} (%)	V_{100} [b] (V)	EQE_{100} [b] (%)	$Luminance_{Max}$ ($cd{\cdot}m^{-2}$)	λ_{peak} (nm)	FWHM (nm)	CIE (x,y)
1	DPEPO:DMAc-DPS 20 wt.%	-	2.8	28.5	3.7	25.8	5420.5	461	83.0	0.173, 0.242
2		0.5	2.8	29.2	3.8	24.1	3809.0	450	40.5	0.165, 0.189
3		1	2.9	25.6	3.9	20.3	3618.7	450	12.8	0.162, 0.159
4		2.0	2.9	19.9	4.4	14.1	2327.5	450	12.3	0.159, 0.115

[a] Voltage at 0.1 cd/m^2; [b] Values at 100 cd/m^2

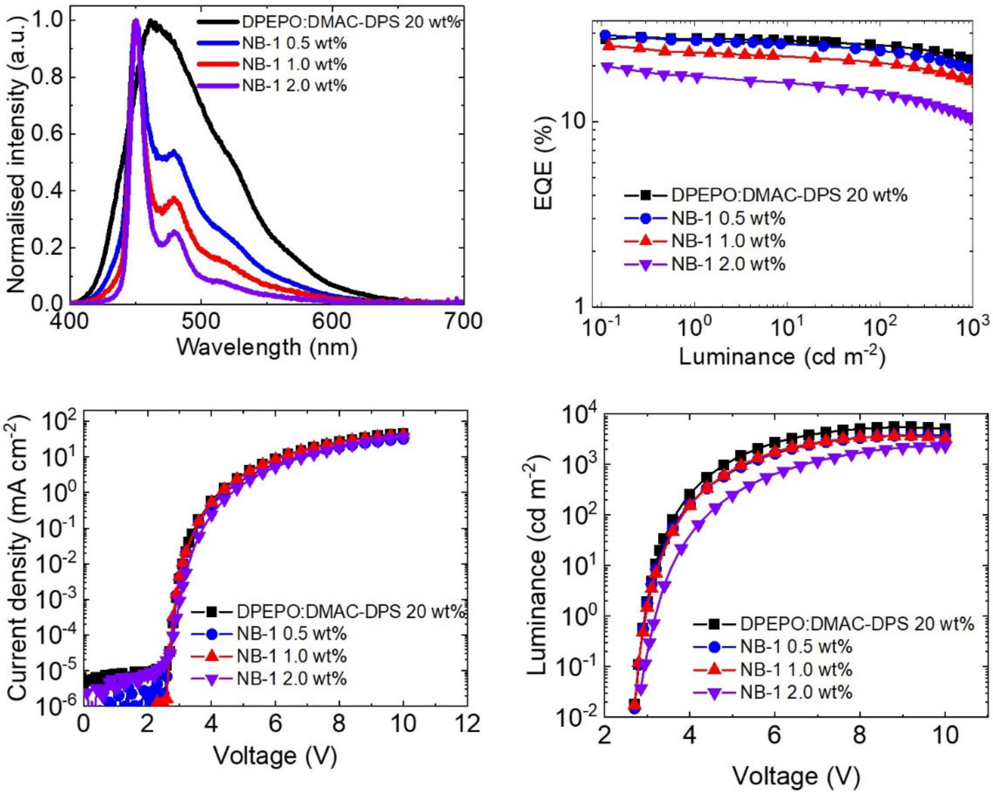

Supplementary Fig. 127: OLED data for DMAC-DPS-based MCHF devices.

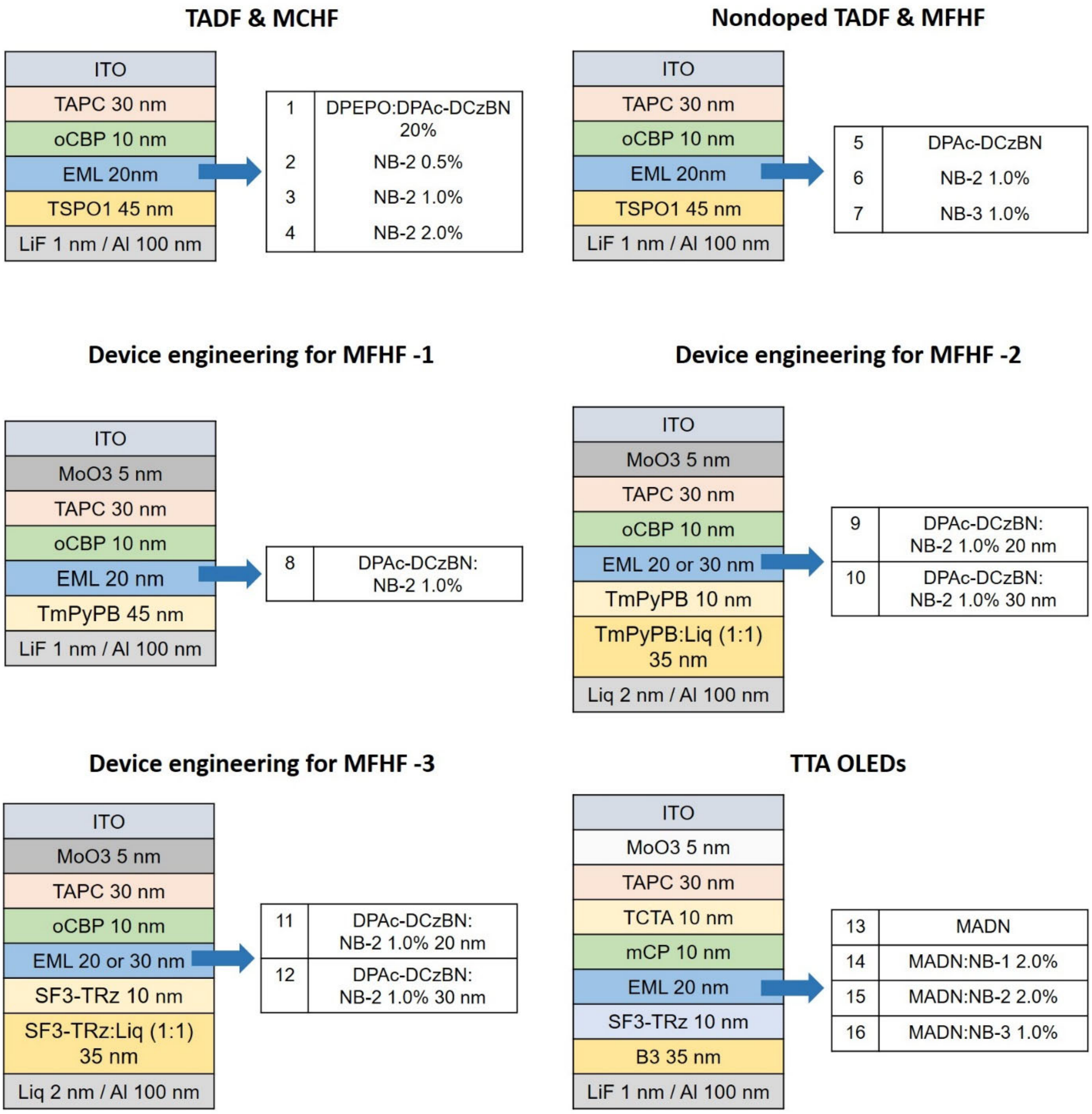

Supplementary Fig. 128: Device structures for OLEDs with device engineering for different emissive systems

Supplementary Table 19: Summary of DPAc-DCzBN-based MCHF and MFHF OLED data

No.	Host	NB-2 (NB-3) wt.%	V_{on}[a)] (V)	EQE_{Max} (%)	V_{100}[b)] (V)	EQE_{100}[b)] (%)	$Luminance_{Max}$ ($cd \cdot m^{-2}$)	λ_{peak} (nm)	FWHM (nm)	CIE (x,y)	Relative Lifetime[c)]
1	DPEPO:DPAc-DCzBN 20 wt%	-	4.3	23.5	7.3	16.9	1054.5	461	71.1	0.163, 0.184	1
2		0.5	4.4	18.8	7.9	14.0	950.9	458	48.2	0.161, 0.175	1.2
3		1.0	5.0	14.3	9.2	10.0	872.2	457	14.8	0.155, 0.142	1.4
4		2.5	5.2	7.7	10.4	4.0	835.4	457	13.6	0.150, 0.119	4.8
5	DPAc-DCzBN	-	2.9	20.3	4.4	18.7	2563.2	476	86.3	0.186, 0.313	45.9
6		1.0	3.0	21.5	4.4	17.8	2494.0	458	15.4	0.161, 0.186	66.9
7		1.0 (NB-3)	3.2	11.2	4.5	9.7	1400.0	451	61.2	0.164, 0.212	3.8
8	DPAc-DCzBN	1.0	2.9	16.0	4.0	15.2	17519.0	458	15.3	0.161, 0.169	179.2
9		1.0	2.9	17.9	4.8	13.7	17563.1	458	15.4	0.160, 0.183	159.1
10		1.0	2.9	19.4	5.2	15.4	21467.7	458	15.3	0.158, 0.179	154.3
11		1.0	2.8	15.7	4.1	12.1	18734.6	458	15.9	0.160, 0.181	151.1
12		1.0	2.8	18.6	4.5	14.1	23211.5	458	15.4	0.158, 0.184	196.6

[a)] Voltage at 0.1 cd/m^2; [b)] Values at 100 cd/m^2 ; [c)] EL intensity decay to 50% measured at 1 mA cm^{-2}

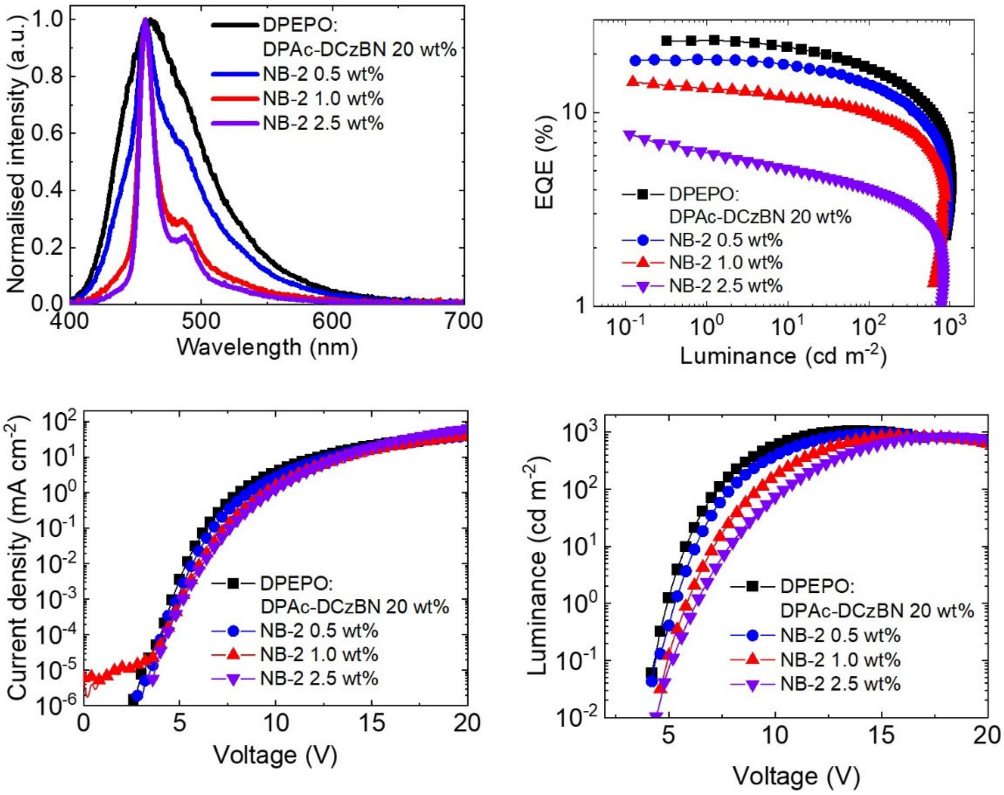

Supplementary Fig. 129: OLED data for DPAc-DCzBN-based MCHF devices (Device 1-4)

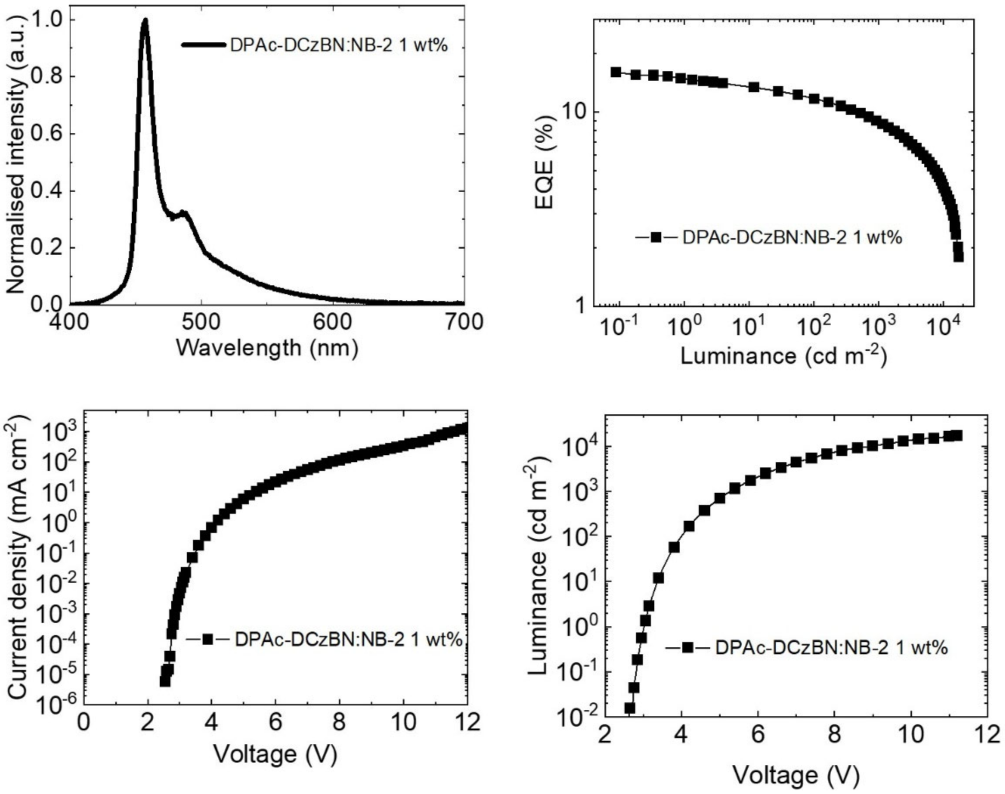

Supplementary Fig. 130: OLED data for DPAc-DCzBN-based MFHF devices (Device 8)

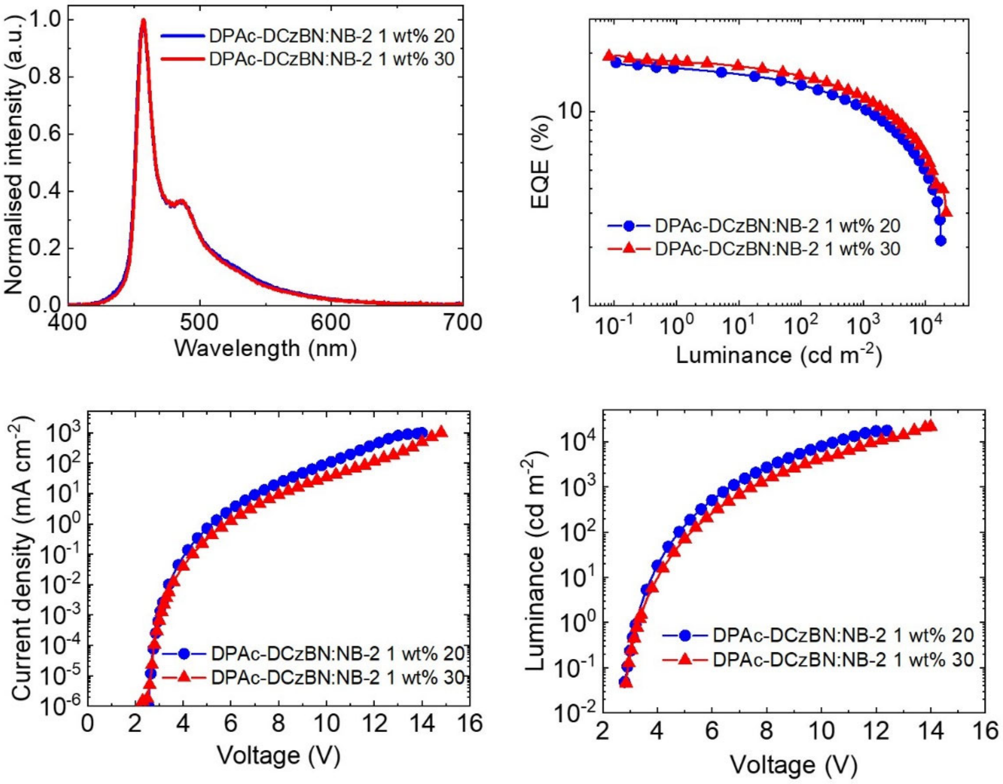

Supplementary Fig. 131: OLED data for DPAc-DCzBN-based MFHF devices (Device 9-10)

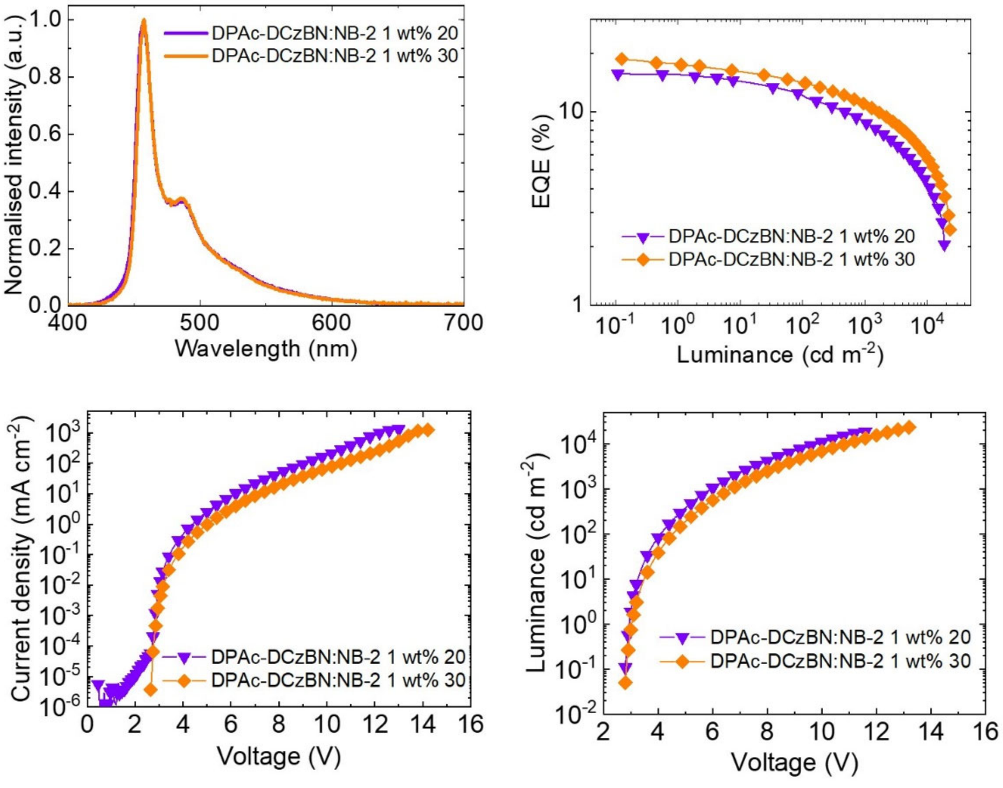

Supplementary Fig. 132: OLED data for DPAc-DCzBN-based MFHF devices (Device 11-12)

Supplementary Table 20: Summary of MADN-based TTA OLED data

No.	Host	Doping wt.%	V_{on}[a] (V)	EQE_{Max} (%)	V_{100}[b] (V)	EQE_{100}[b] (%)	$Luminance_{Max}$ ($cd \cdot m^{-2}$)	λ_{peak} (nm)	FWHM (nm)	CIE (x,y)	Relative Lifetime[c]
**13**	**MADN**	-	3.1	6.5	5.1	5.1	7498.3	440	51.0	0.178, 0.047	94.1
**14**	**MADN:NB-1**	2.0	2.9	8.5	4.9	6.5	10908.6	450	11.9	0.149, 0.050	133.1
**15**	**MADN:NB-2**	2.0	3.1	7.9	4.9	7.6	16477.9	457.5	12.2	0.142, 0.064	147.9
**16**	**MADN:NB-3**	1.0	3.3	5.4	5.4	5.4	20413.3	470	53.6	0.137, 0.195	118.5

[a] Voltage at 0.1 cd/m^2; [b] Values at 100 cd/m^2; [c] EL intensity decay to 50% measured at 1 mA cm^{-2}

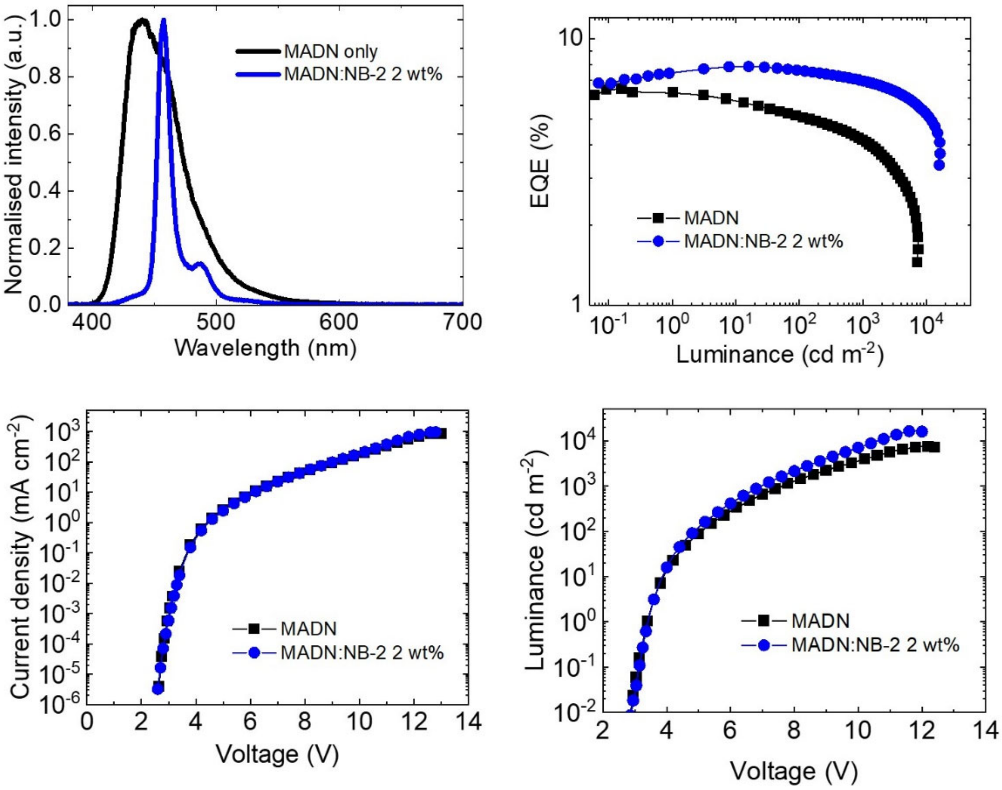

Supplementary Fig. 133: OLED data for MADN-based TTA OLEDs (Device 13, 15)

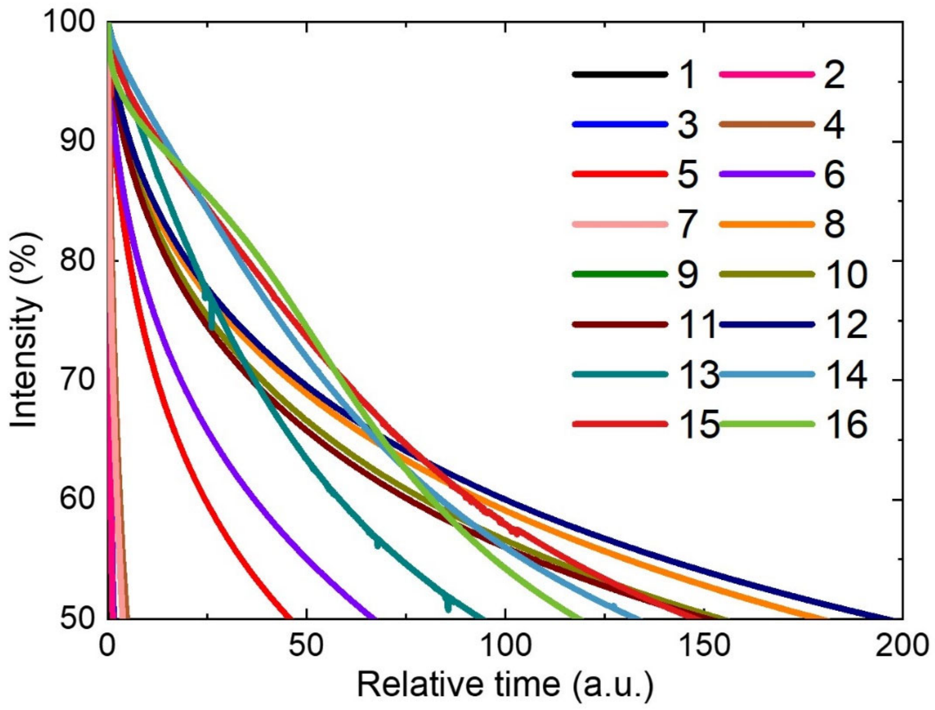

Supplementary Fig. 134: Operational lifetime plots for the devices summarised in Tables S7.7 and S7.8.

Device characterisation: En-DPA and En-Per

We tested other types of encapsulated molecules: encapsulated 9,10-diphenylanthracene (**En-DPA**) and encapsulated perylene (En-perylene). Firstly, **En-DPA** was explored (Supplementary Fig. 135). 9,9'-(4,4'-sulfonylbis(4,1-phenylene))bis(3,6-di-tert-butyl-9H-carbazole) (CZ-PS) is employed as a triplet harvesting exciton donor. **En-DPA**-based device performance is shown in Supplementary Fig. 136 and summarised in Supplementary Table 21. As CZ-PS does not show high efficiency and effective TADF characteristics (~9% EQE),[33] the absolute EQEs are lower than 10%. However, their efficiency is maintained as almost the same even at 10 wt%.

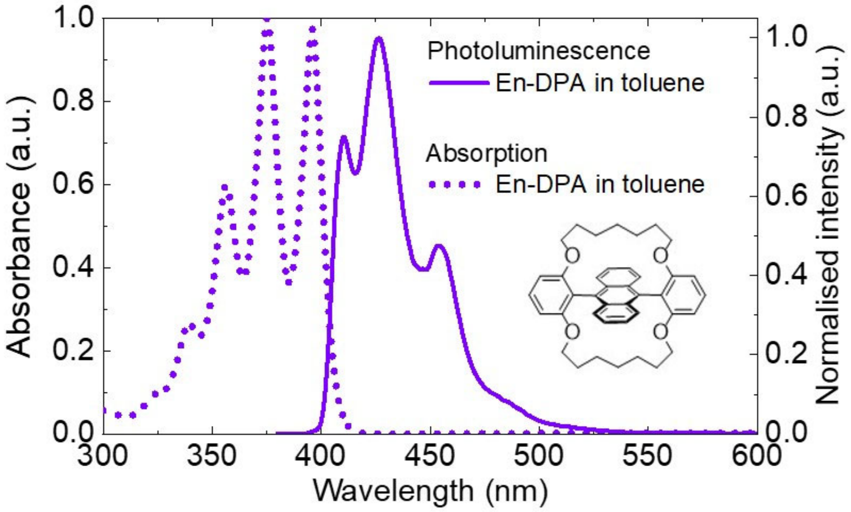

Supplementary Fig. 135: Solution absorption and PL spectra of **En-DPA** and its chemical structure

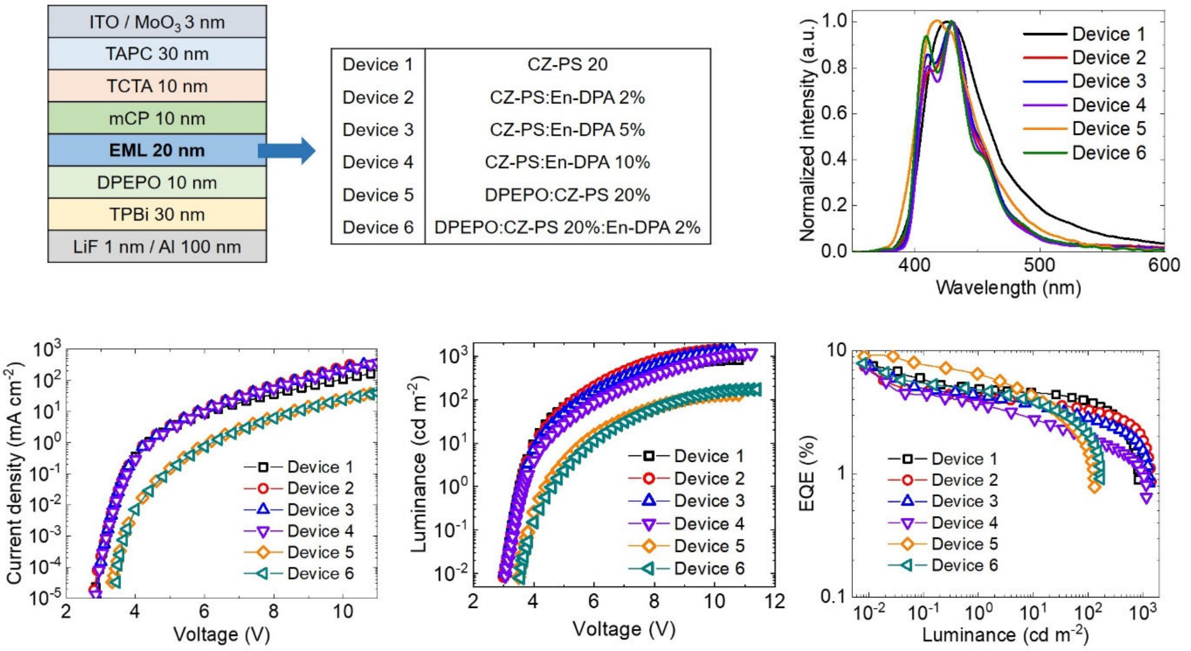

Supplementary Fig. 136: Device structure and device performance of En-DPA-based OLEDs

Supplementary Table 21: En-DPA-based OLED data

Emitter	Host	doping wt.%	V_{on}[a)] (V)	EQE_{Max} (%)	V_{100}[b)] (V)	EQE_{100}[b)] (%)	$Luminance_{Max}$ ($cd \cdot m^{-2}$)	λ_{peak} (nm)	FWHM (nm)	CIE (x,y)
**En-DPA**	**CZ-PS**	-	3.0	7.1	5.4	3.9	832.3	425	57.6	0.174, 0.103
		2.0	2.8	7.5	5.4	3.3	1421.2	410, 430	46.6	0.172, 0.060
		5.0	2.9	7.5	5.6	2.8	1304.1	410, 430	49.0	0.168, 0.055
		10.0	2.9	7.3	6.2	2.0	1168.9	410, 430	44.6	0.167, 0.049
**En-DPA**	**DPEPO:CZ-PS 20 wt%**	-	3.3	9.3	9.0	1.6	132.6	415	53.9	0.164, 0.054
		2.0	3.4	7.9	8.9	2.1	171.9	410, 430	44.6	0.163, 0.049

[a)] Voltage at 0.01 cd/m^2; [b)] Values at 100 cd/m^2

Next, **En-Per** is tested in MFHF systems, As its PL and absorption is red-shifted compared to **En-DPA**, DMAC-DPS can be employed as an exciton donor. The simple device structure shown in Supplementary Fig. 111 is used for MFHF OLEDs, and their device performance are shown and summarised in Supplementary Fig. 138 and Supplementary Table 22. A maximum EQE of 16% is achieved at 1 wt% doping of **En-Per**, which is comparable to the maximum EQE of the DMAC-DPS:**NB-1** 1 wt% MFHF device.

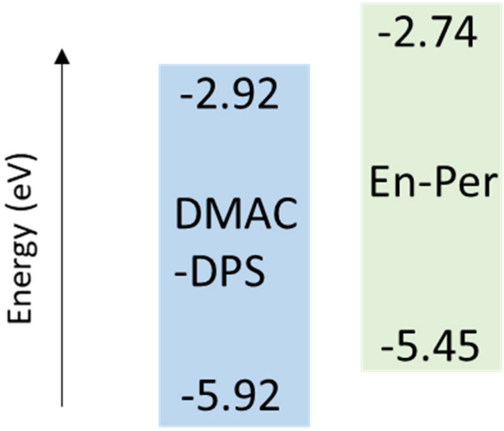

Supplementary Fig. 137: Energy levels of **En-Per** vs. DMAC-DPS.

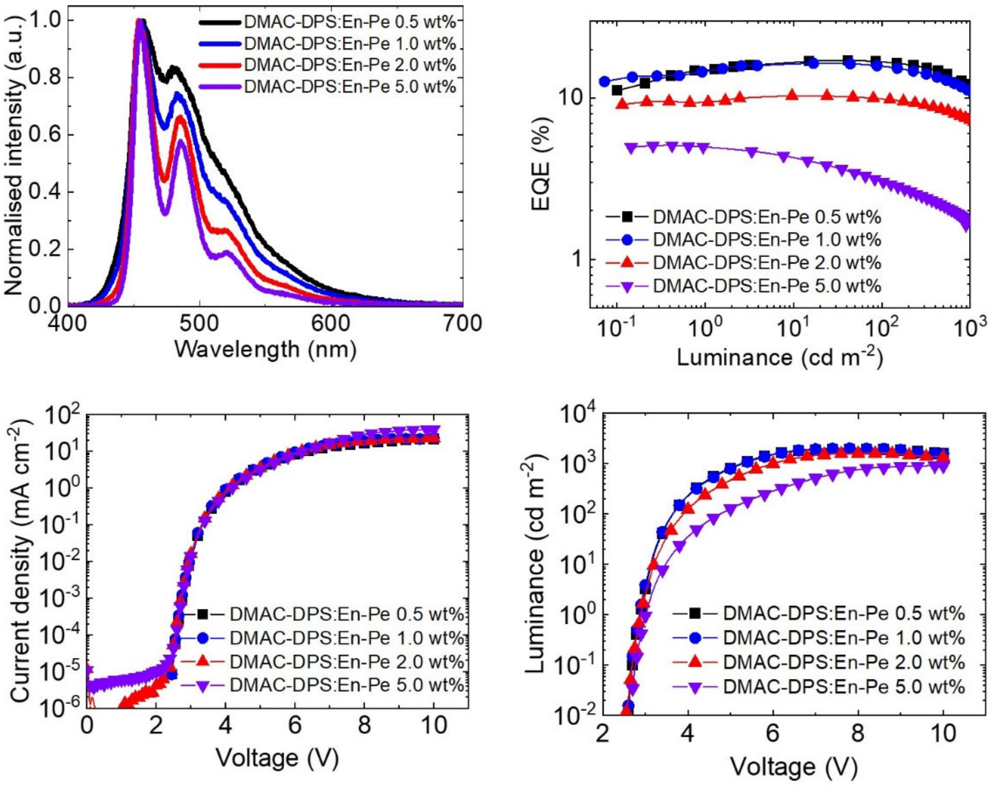

Supplementary Fig. 138: Device performance of **En-Per**-based OLEDs

Supplementary Table 22: En-Per-based OLED data

Emitter	Host	doping wt.%	V_{on} [a)] (V)	EQE_{Max} (%)	V_{100} [b)] (V)	EQE_{100} [b)] (%)	$Luminance_{Max}$ ($cd \cdot m^{-2}$)	λ_{peak} (nm)	FWHM (nm)	CIE (x,y)
En-Per	DMAC-DPS	-	2.7	17.0	3.3	15.8	2261.7	476	88.3	0.185, 0.315
		0.5	2.7	17.0	3.6	16.7	1914.3	455	67.2	0.170, 0.251
		1.0	2.7	16.4	3.6	15.7	1981.9	454	54.2	0.164, 0.231
		2.0	2.7	10.3	3.9	9.9	1582.0	454	47.2	0.155, 0.202
		5.0	2.8	5.1	4.8	3.0	899.9	455	42.6	0.148, 0.175

[a)] Voltage at 0.1 cd/m^2; [b)] Values at 100 cd/m^2

Supplementary References

1. Dressler, J. J., Miller, S. A., Meeuwsen, B. T., Riel, A. M. S. & Dahl, B. J. Synthesis of dilactone bridged terphenyls with crankshaft architectures. *Tetrahedron* **71,** 283–292 (2015).

2. Mallory, F. B. *et al.* Phenacenes: A family of graphite ribbons. Part 3: Iterative strategies for the synthesis of large phenacenes. *Tetrahedron* **57,** 3715–3724 (2001).

3. Wegner, H. A. *et al.* Oligoindenopyrenes: A new class of polycyclic aromatics. *J. Org. Chem.* **71,** 9080–9087 (2006).

4. Ribar, P. *et al.* Donor-Acceptor Molecular Triangles. *Synth.* **49,** 899–909 (2017).

5. Fujiwara, Y. *et al.* Double alkylene-strapped diphenylanthracene as a photostable and intense solid-state blue-emitting material. *J. Org. Chem.* **78,** 2206–2212 (2013).

6. Merz, J. *et al.* Synthesis, photophysical and electronic properties of tetra-donor- or acceptor-substituted: Ortho -perylenes displaying four reversible oxidations or reductions. *Chem. Sci.* **10,** 7516–7534 (2019).

7. Royakkers, J. *et al.* Doubly Encapsulated Perylene Diimides: Effect of Molecular Encapsulation on Photophysical Properties. *J. Org. Chem.* **85,** 207–214 (2020).

8. Gu, R., Van Snick, S., Robeyns, K., Van Meervelt, L. & Dehaen, W. A facile and general method for the synthesis of 6,12-diaryl-5,11- dihydroindolo[3,2-b]carbazoles. *Org. Biomol. Chem.* **7,** 380–385 (2009).

9. Niebel, C. *et al.* Dibenzo[2,3:5,6]pyrrolizino[1,7-bc]indolo[1,2,3-lm]carbazole: A new electron donor. *New J. Chem.* **34,** 1243–1246 (2010).

10. Stoltenberg, D., Lüthje, S., Winkelmann, O., Näther, C. & Lüning, U. Tetraols as templates for the synthesis of large endo-functionalized macrocycles. *European J. Org. Chem.* **4,** 5845–5859 (2011).

11. Reck, L. M., Haberhauer, G. & Lüning, U. Enantiopure Chiral Concave 1,10-Phenanthrolines. *European J. Org. Chem.* 1119–1131 (2016). doi:10.1002/ejoc.201501289

12. Wisser, F. M. *et al.* Molecular Porous Photosystems Tailored for Long-Term Photocatalytic CO2 Reduction. *Angew. Chemie - Int. Ed.* **59,** 5116–5122 (2020).

13. Sheldrick, G. M. SHELXT - Integrated space-group and crystal-structure determination. *Acta Crystallogr. Sect. A Found. Crystallogr.* **71,** 3–8 (2015).

14. Sheldrick, G. M. Crystal structure refinement with SHELXL. *Acta Crystallogr. Sect. C Struct. Chem.* **71,** 3–8 (2015).

15. Spek, A. L. PLATON SQUEEZE: A tool for the calculation of the disordered solvent contribution to the calculated structure factors. *Acta Crystallogr. Sect. C Struct. Chem.* **71,** 9–18 (2015).

16. Neese, F. Software update: The ORCA program system—Version 5.0. *Wiley Interdiscip. Rev. Comput. Mol. Sci.* **12,** e1606 (2022).

17. Weigend, F. & Ahlrichs, R. Balanced basis sets of split valence, triple zeta valence and quadruple zeta valence quality for H to Rn: Design and assessment of accuracy. *Phys. Chem. Chem. Phys.* **7,** 3297–3305 (2005).

18. Chai, J. Da & Head-Gordon, M. Systematic optimization of long-range corrected hybrid

density functionals. *J. Chem. Phys.* **128,** 84106 (2008).

19. Tawada, Y., Tsuneda, T., Yanagisawa, S., Yanai, T. & Hirao, K. A long-range-corrected time-dependent density functional theory. *J. Chem. Phys.* **120,** 8425–8433 (2004).

20. De Souza, B., Neese, F. & Izsák, R. On the theoretical prediction of fluorescence rates from first principles using the path integral approach. *J. Chem. Phys.* **148,** 34104 (2018).

21. Köuppel, H., Domcke, W. & Cederbaum, L. S. Multimode Molecular Dynamics Beyond the Born-Oppenheimer Approximation. *Adv. Chem. Phys.* **57,** 59–246 (1984).

22. Mai, S., Marquetand, P. & González, L. Nonadiabatic dynamics: The SHARC approach. *Wiley Interdiscip. Rev. Comput. Mol. Sci.* **8,** e1370 (2018).

23. Plasser, F., Gómez, S., Menger, M. F. S. J., Mai, S. & González, L. Highly efficient surface hopping dynamics using a linear vibronic coupling model. *Phys. Chem. Chem. Phys.* **21,** 57–69 (2019).

24. Martin, R. L. Natural transition orbitals. *J. Chem. Phys.* **118,** 4775–4777 (2003).

25. Plasser, F. TheoDORE: A toolbox for a detailed and automated analysis of electronic excited state computations. *J. Chem. Phys.* **152,** 84108 (2020).

26. Lu, T. & Chen, F. Quantitative analysis of molecular surface based on improved Marching Tetrahedra algorithm. *J. Mol. Graph. Model.* **38,** 314–323 (2012).

27. Imbush, G. F. in *Luminescence Spectroscopy* (ed. Lumb, M. D.) 93–148 (Academic Press, 1978).

28. Taniguchi, M. & Lindsey, J. S. Database of Absorption and Fluorescence Spectra of >300 Common Compounds for use in PhotochemCAD. *Photochem. Photobiol.* **94,** 290–327 (2018).

29. Liu, T. *et al.* Ultrapure blue organic light-emitting diodes exhibiting 13 nm full width at half-maximum. *J. Mater. Chem. C* **10,** 7735–8128 (2022).

30. Nakagawa, T. *et al.* Electroluminescence based on thermally activated delayed fluorescence generated by a spirobifluorene donor–acceptor structure. *Chem. Commun.* **48,** 9580 (2012).

31. Förster, T. 10th Spiers Memorial Lecture: Transfer Mechanisms of Electronic Excitation. *Discuss. Faraday Soc.* **27,** 7–17 (1959).

32. Baumann, J. & Fayer, M. D. Excitation transfer in disordered two-dimensional and anisotropic three-dimensional systems: Effects of spatial geometry on time-resolved observables. *J. Chem. Phys.* **85,** 4087–4107 (1986).

33. Zhang, Q. *et al.* Design of efficient thermally activated delayed fluorescence materials for pure blue organic light emitting diodes. *J. Am. Chem. Soc.* **134,** 14706–14709 (2012).
